# Stereoretentive enantioconvergent reactions

**DOI:** 10.1038/s41557-024-01504-1

**Published:** 2024-04-17

**Authors:** Steven H. Bennett, Jacob S. Bestwick, Vera P. Demertzidou, David J. Jones, Helen E. Jones, François Richard, Joshua A. Homer, Rosie Street-Jeakings, Andrew F. Tiberia, Andrew L. Lawrence

**Affiliations:** EaStCHEM School of Chemistry, University of Edinburgh, Edinburgh, UK

**Keywords:** Stereochemistry, Synthetic chemistry methodology, Synthetic chemistry methodology

## Abstract

Enantioconvergent reactions are pre-eminent in contemporary asymmetric synthesis as they convert both enantiomers of a racemic starting material into a single enantioenriched product, thus avoiding the maximum 50% yield associated with resolutions. All currently known enantioconvergent processes necessitate the loss or partial loss of the racemic substrate’s stereochemical information, thus limiting the potential substrate scope to molecules that contain labile stereogenic units. Here we present an alternative approach to enantioconvergent reactions that can proceed with full retention of the racemic substrate’s configuration. This uniquely stereo-economic approach is possible if the two enantiomers of a racemic starting material are joined together to form one enantiomer of a non-*meso* product. Experimental validation of this concept is presented using two distinct strategies: (1) a direct asymmetric coupling approach, and (2) a multicomponent approach, which exhibits statistical amplification of enantiopurity. Thus, the established dogma that enantioconvergent reactions require substrates that contain labile stereogenic units is shown to be incorrect.

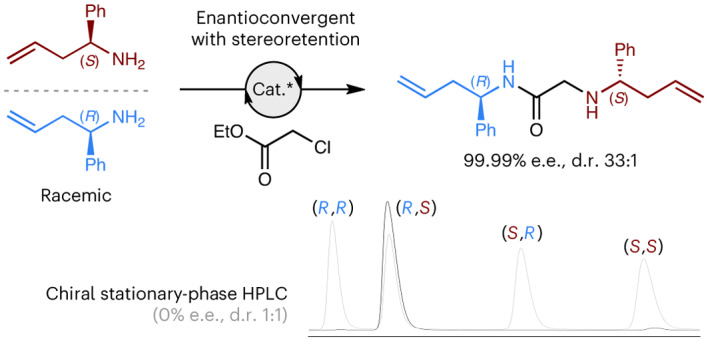

## Abstract (web summary)

Enantioconvergent reactions convert both enantiomers of a racemic starting material into a single enantioenriched product. All currently known enantioconvergent processes necessitate the loss or partial loss of the racemic substrate’s stereochemical information. Now, an alternative approach has been developed that proceeds with full retention of the racemic substrate’s configuration.

## Main

**Fig. 1:**
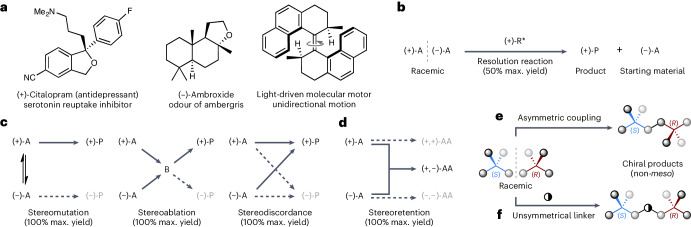
Chiral molecules, resolution reactions and enantioconvergent reactions. **a**, Examples of chiral molecules in which the absolute configuration is critical to their function. **b**, Resolution reactions, which are inherently limited to a maximum 50% yield (A is starting material, R* is a chiral reagent, P is product). **c**, Established approaches to achieving enantioconvergent reactions (A is starting material, B is an intermediate, P is product). **d**, A stereoretentive approach to enantioconvergent reactions (A is starting material, AA is a chiral non-*meso* product). **e**, Stereoretentive enantioconvergent heterochiral dimerization. **f**, Stereoretentive enantioconvergent multicomponent reaction using an unsymmetrical linker molecule (represented as ◑).

From medicine to materials science^1,2^, the ability to control the absolute configuration of chiral molecules is vital to controlling their function (Fig. 1a). Since Pasteur’s seminal work on the chiral resolution of racemic tartrates^3^, scientists have sought out new ways to access chiral molecules in enantioenriched form. The stereoselective synthesis of chiral molecules in enantioenriched form, known as asymmetric synthesis, has been a great success for the discipline of synthetic organic chemistry. The importance of asymmetric synthesis was recognized in 2001 when the Nobel Prize in Chemistry was awarded to Knowles, Noyori and Sharpless for their development of metal-catalysed asymmetric reactions. The field has remained an innovative and vibrant area of research, with the 2021 Nobel Prize in Chemistry awarded to List and MacMillan for their development of organocatalysed asymmetric reactions. Indeed, asymmetric reactions are now routine, both in industrial and academic settings, with a wide variety of catalysts available, from intricate precious-metal complexes to bespoke engineered enzymes.

The approach taken in an asymmetric synthesis is dictated by the stereochemical nature of the starting material. When starting from an achiral substrate an enantioselective reaction can be used to access a new chiral product in up to 100% e.e. and 100% yield. Asymmetric synthesis using racemic substrates can be achieved through resolution reactions (for example, kinetic resolutions^4,5^), wherein one enantiomer of the starting material is converted into a new product (Fig. 1b). This unfortunately results in a maximum achievable yield of 50%, which represents a notable weakness in contemporary asymmetric synthesis. Enantioconvergent reactions, on the other hand, can be used to achieve full conversion of both enantiomers of a racemic starting material into a single enantioenriched product in up to 100% e.e. and 100% yield^6–8^. An increasing number of enantioconvergent reactions have been developed in recent years, but they all rely on just three conceptual approaches for achieving enantioconvergency: stereomutation, stereoablation and stereodiscordance (Fig. 1c). Stereomutation involves the mutation (that is, interconversion) of the configuration of the racemic starting materials’ stereogenic unit(s)^6–8^, and is most commonly associated with ‘dynamic kinetic resolutions’^9–12^. In stereoablative processes^6–8,13^, such as type II dynamic kinetic asymmetric transformations^14^, the stereogenic units in both enantiomers of the racemic starting material are ablated (that is, destroyed), thus generating a common intermediate that undergoes a stereoselective transformation to give an enantioenriched product. In stereodiscordant processes (a terminology we introduce here for the first time), such as ‘enantioconvergent parallel kinetic resolutions’^15,16^, one enantiomer undergoes a stereoinversion process whilst the other enantiomer proceeds with stereoretention.

Although each of these three established approaches have their own particular advantages and challenges (Fig. 1c), they are all limited to racemic substrates that contain labile stereogenic units. This is because they all necessitate the loss, or partial loss, of stereochemical information during the reaction, either by mutation, ablation or partial inversion. Furthermore, substrates containing multiple stereogenic units are not generally amenable to any of these established approaches unless, for example, the substrate is pseudosymmetric^14,17^. Thus, the only currently available option for asymmetric synthesis using racemic substrates that contain robust and/or multiple stereogenic units is to use inherently wasteful resolution reactions (Fig. 1b).

We realized that the configuration of a racemic substrate could be fully retained during an enantioconvergent reaction if the two enantiomers of a racemic substrate were coupled together to form one enantiomer of a non-*meso* product (Fig. 1d). We herein provide experimental validation of this stereoretentive approach to enantioconvergent reactions using two distinct strategies. First, we describe a direct asymmetric coupling approach, wherein a heterochiral dimer of a racemic substrate is produced in enantioenriched form (Fig. 1e). Second, we disclose a multicomponent approach that uses an unsymmetrical linker to ensure the final product is chiral (that is, non-*meso*) (Fig. 1f).

## Results and discussion

### Direct coupling approach

**Fig. 2:**
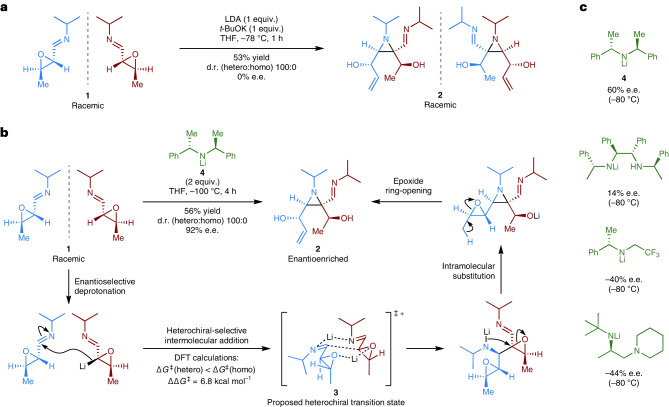
Development of a stereoretentive enantioconvergent reaction. **a**, A known heterochiral selective, but not enantioconvergent, aza-Darzens dimerization of racemic imine epoxide **1**, as reported by Würthwein and co-workers^19^. **b**, An enantioconvergent aza-Darzens reaction achieved using chiral lithium amide **4**. The heterochiral selectivity was rationalized as a result of a bis-lithium, cationic Zimmerman–Traxler-type transition state **3** for the addition step^19^. **c**, Results obtained when using other chiral lithium amides.

Dimerizations of racemic substrates are intrinsically more complex than those involving achiral or enantiopure substrates because of the issue of homochiral–heterochiral selectivity. That is, one enantiomer of a racemic substrate may react with another molecule of the same configuration (homochiral dimerization) or opposite configuration (heterochiral dimerization). If the selectivity of such a dimerization could be controlled to be both heterochiral-selective and enantioselective, then a stereoretentive enantioconvergent reaction would be realized (Fig. 1e). The aza-Darzens reaction was selected as a model dimerization to experimentally validate stereoretention as a viable concept for enantioconvergent reactions^18^ (Fig. 2). In 2001, Würthwein, Fröhlich and Alickmann reported that treatment of racemic imine epoxide **1** with LDA or LDA/KO*t*-Bu resulted in an exquisitely heterochiral-selective aza-Darzens dimerization to give racemic aziridine **2** (Fig. 2a)^19^. The inherent heterochiral selectivity of this reaction was postulated to be a result of a bis-lithium, cationic Zimmerman–Traxler type transition state **3** for the addition step, with density functional theory calculations (B3LYP/6-31G*) revealing a 6.8 kcal mol^–1^ preference for the heterochiral combination^19^. We aimed to render this process enantioconvergent by replacing the achiral base with an enantiopure chiral base. Aziridine **2** was successfully formed in enantioenriched form when using a number of different chiral lithium amides at −80 °C (Fig. 2c; for full details, see Supplementary Fig. 1). The *C*_2_-symmetric base **4** provided the highest enantioselectivity (60% e.e. at −80 °C)^20^, which could be improved by lowering the reaction temperature to −100 °C to give aziridine **2** in 56% isolated yield and 92% e.e. (Fig. 2b). This represents the asymmetric synthesis of a highly substituted aziridine but more importantly constitutes an enantioconvergent reaction that proceeds with stereoretention. The benefits in terms of stereo-economy are clear; three of the four stereogenic units in the racemic imine epoxide **1** are fully retained in the enantioenriched aziridine **2**. A new stereogenic centre is also created during the intermolecular addition step, which compensates for the one lost in the final epoxide ring-opening step.

### Multicomponent approach

**Fig. 3:**
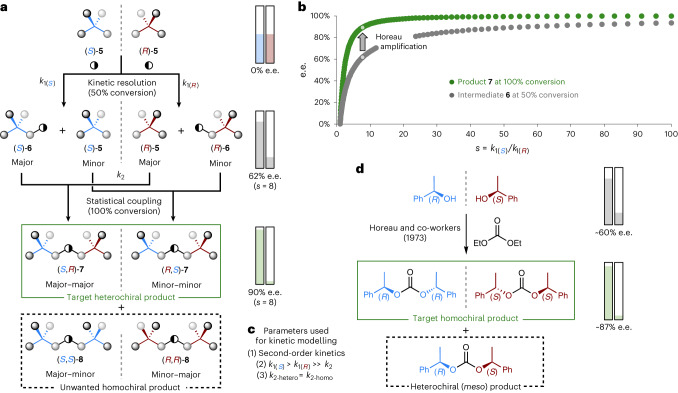
Kinetic modelling for multicomponent stereoretentive enantioconvergent reactions. **a**, Envisaged reaction manifold for a multicomponent stereoretentive enantioconvergent reaction, using an asymmetric linker molecule (◑), with example of statistical amplification of enantiopurity for a poorly selective initial KR (*s* = 8). **b**, Statistical amplification of enantiopurity (that is, Horeau amplification) over a representative range of selectivity factors in the initial KR (1 < *s* < 100). **c**, Parameters used for the kinetic modelling: (1) simple second-order kinetics for both steps; (2) the coupling step is much slower than the initial KR (*k*_1_ ≫ *k*_2_); and (3) there is no asymmetric induction in the second step (*k*_2-hetero_ = *k*_2-homo_). More complex reaction kinetics and/or asymmetric induction in the second step could result in stereoisomeric ratios different from those predicted. Full details of the kinetic modelling are described in section 7 of the Supplementary Information. **d**, One of Horeau’s original reactions exhibiting statistical amplification of enantiopurity.

Having realized our first goal of achieving a direct stereoretentive enantioconvergent coupling of a racemic substrate, we next turned our attention to demonstrating the concept of stereoretention in multicomponent enantioconvergent reactions (Fig. 1f). In this approach (Fig. 3), an unsymmetrical linker molecule (represented as ◑) provides a general and predictable approach to achieving heterochiral selectivity, whilst ensuring the final heterochiral product **7** is chiral (that is, non-*meso*). The envisaged process begins with a kinetic resolution (KR) of the racemic starting material **5** with the linker ◑, giving an enantioenriched intermediate **6** and resolved starting material **5** (Fig. 3a). These are then coupled together to form the target heterochiral species **7** as the major product, alongside minor quantities of the homochiral product **8**. Importantly, this coupling step will lead to statistical amplification of enantiopurity (that is, the Horeau principle^21^), meaning that even when using a moderately selective KR the final product **7** will be formed in exceptionally high enantiopurity^22–24^. Kinetic modelling of this multicomponent process illustrates how impressive this amplification of enantiopurity will be^25^ (Fig. 3a–c). In Fig. 3b, the calculated e.e. of the intermediate **6** (at 50% conversion) and the e.e. of the final product **7** (at 100% conversion) are plotted against the selectivity factor, *s* = *k*_1(*S*)_/*k*_1(*R*)_, of the initial KR (note: *s* factors are often known as *E* values for biocatalytic KR)^26^. The enantioamplification is substantial; for example, in Fig. 3a the expected results are shown for a low *s*-factor of 8 in the initial KR (note: *s* < 15 low; 15 < *s* < 30 acceptable; 30 < *s* < 50 good; *s* > 50 excellent). In a normal KR an *s*-factor of 8 would produce intermediate **6** in just 62% e.e. (at 50% conversion), whereas in this enantioconvergent process the final product **7** will be formed in 90% e.e. (at 100% conversion). Thus, the demands for achieving selectivity in the initial KR are remarkably low, meaning the labour-intensive reaction/catalyst optimization process usually associated with developing new asymmetric transformations can be largely avoided^27–29^. This statistical amplification of enantiopurity has strong parallels to the original studies reported by Horeau and co-workers in 1973^21^ (Fig. 3d), but it is different. Horeau’s seminal work demonstrated that statistical amplification of enantiopurity could be achieved by coupling enantioenriched samples with symmetrical linkers to give homochiral dimers in amplified enantiopurity, which comes at the cost of forming small amounts of the *meso*-heterochiral dimers (see example in Fig. 3d). Whereas, in our multicomponent reactions, unsymmetrical linkers are used in stereoselective processes to give the heterochiral dimers in amplified enantiopurity at the cost of forming small amounts of the unwanted homochiral dimers (Fig. 3a).

**Fig. 4:**
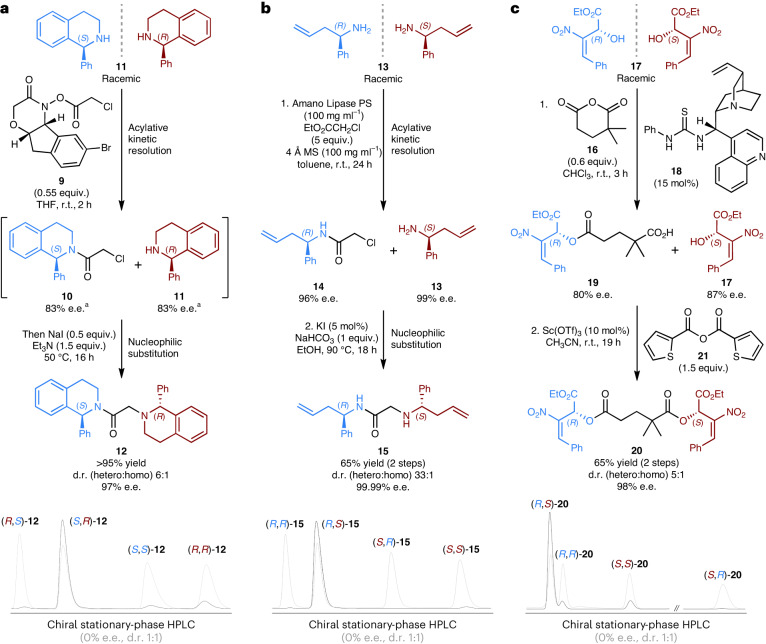
Multicomponent stereoretentive enantioconvergent reactions. **a**, Enantioconvergent reaction starting from racemic tetrahydroisoquinoline **11** using a Bode-type acylating reagent **9**. Et_3_N, triethylamine; THF, tetrahydrofuran. **b**, Enantioconvergent synthesis of α-aminoamide **15** from racemic primary amine **13** using a lipase biocatalyst and ethyl chloroacetate as the linker. EtOH, ethanol; MS, molecular sieve. **c**, Enantioconvergent synthesis starting from racemic secondary alcohol **17** using organocatalyst **18** and 2,2-dimethylglutaric anhydride **16** as the linker. HPLC chromatograms of the products (**12**, **15** and **20**) are shown in black and are overlaid with chromatograms of their statistical stereoisomeric mixtures (that is, d.r. 1:1, 0% e.e.) in grey. ^a^e.e. values are estimates assuming 50% conversion (*s* = 28); for full details, see section 3 of the Supplementary Information.

KR by acylation, which is a common method in asymmetric synthesis^30^, was selected as a model system with which to develop multicomponent stereoretentive enantioconvergent processes (Fig. 4). Bode and co-workers have reported the KR of racemic amines using acylated chiral hydroxamic acid reagents^31,32^. We designed an analogous chloroacetylated reagent **9**, so that that the intermediate from the KR would be a chloroacetamide, **10**, rather than a simple acetamide, which could undergo a final coupling with the remaining amine **11** via nucleophilic substitution. The impact of having the α-chloro substituent on reagent **9** was investigated for the KR of racemic tetrahydroisoquinoline **11**. Thankfully, the α-chloro substituent was found to be well tolerated and resulted in an acceptable *s*-factor of 28, which at a perfect 50% conversion would give chloroacetamide **10** in 83% e.e. A multicomponent stereoretentive enantioconvergent reaction was achieved by first conducting a KR of racemic tetrahydroisoquinoline **11** with our α-chloro-Bode reagent **9** in tetrahydrofuran at room temperature. Once the KR was complete, the reaction temperature was increased to 50 °C and triethylamine and NaI were added to facilitate the final nucleophilic substitution. This gives the target α-aminoamide **12** in an amplified enantiopurity of 97% e.e. (Fig. 4a; for full details, see section 3 of the Supplementary Information). This reaction, alongside the aza-Darzens dimerization (Fig. 2), experimentally validate stereoretention as a viable concept for enantioconvergent reactions. These conceptually ground-breaking reactions were achieved via stoichiometric reagent-controlled stereoselectivity, that is, a super-stoichiometric chiral reagent and a traceless chiral auxiliary. Our attention next turned to demonstrating the concept of stereoretention in enantioconvergent reactions using asymmetric catalysis.

A catalytic stereoretentive enantioconvergent synthesis was achieved using the commercially available Amano Lipase PS from *Burkholderia cepacia*^33^. A biocatalytic KR of racemic amine **13** using ethyl chloroacetate as the linker gives both chloroacetamide **14** and recovered amine **13** in highly enantioenriched form (96% e.e. and 99% e.e., respectively)^34^. Filtration to remove the biocatalyst and molecular sieves followed by evaporative removal of the solvent and excess ethyl chloroacetate gives a clean mixture of chloroacetamide **14** and recovered amine **13**. These are then coupled together through a nucleophilic substitution reaction, using NaHCO_3_ and catalytic KI, to give α-aminoamide **15** in 65% yield over the two steps. Owing to the highly selective nature of the biocatalytic KR, the final product **15** is formed in near enantiopure form (99.99% e.e.), with only trace quantities of the unwanted homochiral product present (d.r. 33:1) (Fig. 4b).

For these multicomponent reactions the design of the linker is very important and must satisfy a number of criteria. Most importantly, it must allow for a highly site-selective KR of the racemic substrate whilst containing an orthogonal reactive site for the subsequent statistical coupling. For reactions based on acylative KR of amines the inclusion of an α-electrophilic site worked very well in terms of reactivity and selectivity (Fig. 4a,b). For reactions based on the acylative KR of alcohols, however, this linker design did not work well owing to transesterification competing with the desired nucleophilic substitution. Therefore, 2,2-dimethylglutaric anhydride **16** was used as a highly site-selective acylating reagent for the organocatalytic KR of racemic secondary alcohol **17**, using the bifunctional cinchona-thiourea catalyst **18**^35^. The free carboxylic acid in intermediate **19** then served very well as an orthogonal reactive site for a subsequent modified-Shiina esterification^36^, to give diester **20** in 65% yield over the two steps in good heterochiral selectivity (d.r. 5:1) and amplified enantiopurity (98% e.e.).

### Diastereoconvergent approach

**Fig. 5:**
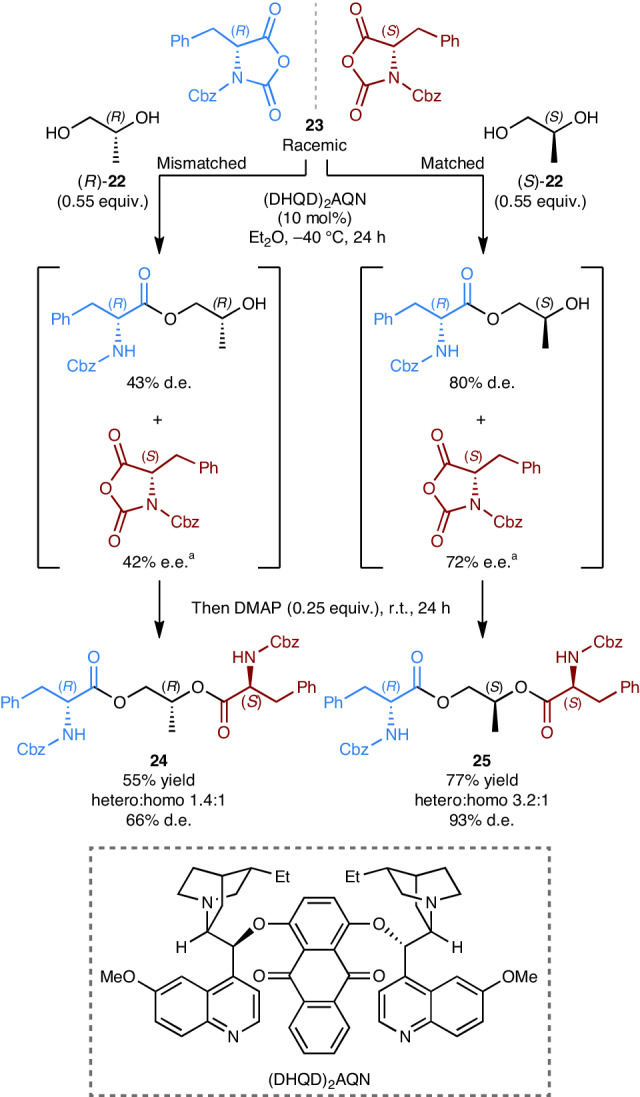
Investigating triple-stereodifferentiation effects in multicomponent diastereoconvergent reactions using enantiopure linkers. Identification of a triple-stereodifferentiation effect using (*R*)/(*S*)-propylene glycol **22** as the asymmetric linker in a diastereoconvergent reaction of racemic phenylalanine *N*-carboxyanhydride **23** using (DHQD)_2_AQN as catalyst. DMAP, 4-(dimethylamino)pyridine; Et_2_O, diethyl ether. ^a^e.e. values were determined from the corresponding methyl esters, after hydrolysis and methylation using KHCO_3_ and CH_3_I; for full details, see section 5 of the Supplementary Information.

We were curious to explore the potential benefits of using chiral enantiopure linkers in multicomponent diastereoconvergent processes. Specifically, we were interested in trying to identify and leverage triple-stereodifferentiation effects to boost selectivity (that is, higher-order matched–mismatched effects)^37^. Traditional KRs are examples of double stereodifferentiating reactions, where the reaction outcome depends on the absolute configuration of the two participants (for example, the substrate and the catalyst)^4,5^. By using a chiral enantiopure linker the outcome of our initial KR will now depend to some extent on the absolute configuration of all three participants. To see if triple stereodifferentiation might be observable, and therefore potentially useful, we selected the KR of racemic amino acid *N*-carboxyanhydrides with alcohols as a suitable reaction manifold^38^ (Fig. 5). Propylene glycol **22**, which is commercially available in both enantiomeric forms, was selected as a model chiral linker as it satisfies the design requirements already outlined above; the primary alcohol can serve as the most reactive site for the KR and the secondary alcohol can then serve as the reactive site for the final nucleophilic acyl substitution coupling (Fig. 5). Thus, two reactions using (DHQD)_2_AQN as catalyst were performed on racemic phenylalanine *N*-carboxyanhydride **23**, with (*S*)-propylene glycol **22** used in one reaction and (*R*)-propylene glycol **22** used in the other. After 24 h at −40 °C we observed a pronounced difference in selectivity between the initial kinetic resolutions, with (*S*)-propylene glycol **22** identified as the ‘matched’ stereodifferentiating linker. Both reactions were then allowed to warm to room temperature and DMAP added to catalyse the final nucleophilic acyl substitution. When (*R*)-propylene glycol **22** was used as the linker, product **24** was obtained in a poor heterochiral–homochiral ratio of 1.4:1 with the desired heterochiral adduct formed in just 66% d.e. In comparison, when using (*S*)-propylene glycol **22**, the final product **25** was formed in a heterochiral–homochiral ratio of 3.2:1 with the heterochiral adduct formed in 93% d.e. (Fig. 5).

## Conclusion and outlook

The results presented in this report experimentally validate stereoretention as a conceptually different and complementary approach to achieving enantioconvergency. This concept can now be exploited by others to develop new stereoconvergent methodology which, for the first time, can utilize racemic substrates with robust and multiple stereogenic units. Controlling the many aspects of reactivity and selectivity needed for these processes stands as an exciting new challenge for the synthetic chemistry community. Addressing these challenges will allow for a substantial expansion of our collective toolbox of stereoconvergent methods, which has the potential to impact all areas of science in which the configuration of chiral molecules is important for their function^1,2^.

## Online content

Any methods, additional references, Nature Portfolio reporting summaries, source data, extended data, supplementary information, acknowledgements, peer review information; details of author contributions and competing interests; and statements of data and code availability are available at 10.1038/s41557-024-01504-1.

### Supplementary information

Supplementary Information Supplementary Figs. 1–5, Tables 1–6, discussion, experimental procedures and data, HPLC chromatograms, NMR spectra, and kinetic modelling.

## Data Availability

All data to support the conclusions are available in the Supplementary Information.
