# Supplementary material for: Stereoretentive enantioconvergent reactions
Source: Nat Chem. 2024 Apr 17;16(7):1177–83. doi: 10.1038/s41557-024-01504-1 (PMC11230902; doi:10.1038/s41557-024-01504-1)
Supplement: Supplementary file 1 — Supplementary Figs. 1–5, Tables 1–6, discussion, experimental procedures and data, HPLC chromatograms, NMR spectra, and kinetic modelling. [file 41557_2024_1504_MOESM1_ESM.pdf]

nature chemistry

Article https://doi.org/10.1038/s41557-024-01504-1

Stereoretentive enantioconvergent reactions

In the format provided by the authors and unedited

Table of Contents

1. Materials and Methods - 4 -

1.1. Thin-Layer Chromatography (TLC) - 4 -

1.2. Chromatography - 4 -

1.3. Solvents - 4 -

1.4. Analysis - 5 -

2. Stereoretentive Enantioconvergent *Aza*-Darzens Dimerization - 6 -

2.1. Synthesis of Racemic Aziridine - 6 -

2.2. General Procedure for Chiral Lithium Amides - 8 -

2.3. Stereoretentive Enantioconvergent Aza-Darzens Reaction - 9 -

3. Stereoretentive Enantioconvergent Reaction with a Traceless Auxiliary - 11 -

3.1. Synthesis of Racemic α-Aminoamides - 11 -

3.2. HPLC Trace Assignment of Dimeric α-Aminoamides - 13 -

3.3. Stereoretentive Enantioconvergent Traceless Auxillary Reaction - 18 -

3. Biocatalytic Stereoretentive Enantioconvergent Reaction - 24 -

3.1. Synthesis of Racemic-Homo/Heterodimer - 24 -

3.2. Synthesis of (*R*,*S*)-Heterodimer - 33 -

3.3. Optimisation of the Biocatalytic Kinetic Resolution and S_N2 Dimerisation - 45 -

4. Organocatalytic Stereoretentive Enantioconvergent Reaction - 49 -

4.1. Synthesis of Starting Materials - 49 -

4.2. Optimisation of the second esterification - 55 -

4.3. Synthesis of Racemic Diester - 59 -

4.4. Kinetic Resolution of (*rac*)-nitroalcohol 17 - 61 -

4.5 HPLC trace assignment of diester 20 isomers - 63 -

4.6. Synthesis of Enantioenriched Diester - 66 -

5. Organocatalytic Stereoretentive Diastereoconvergent Reaction - 68 -

5.1. Preparation of starting materials - 68 -

5.2. Procedure for the Kinetic Resolution.. - 70 -
5.3. Procedure for the Stereoretentive Diastereoconvergent Coupling: .... - 74 -
5.4. Preparation of Authentic Samples .... - 81 -
5.5 HPLC Traces Assignment.. - 89 -
5.6. Calibration.. - 91 -
5.7. Optimisation of the Stereoretentive Diasteroconvergent Reaction............. - 96 -
6. NMR Spectra (with Table of Contents).. - 102 -
7. Kinetic Modelling .... - 139 -
8. References... - 143 -

1. Materials and Methods

Unless otherwise stated, all reactions were carried out using oven-dried glassware under a positive pressure of dry nitrogen using standard Schlenk manifold techniques. All starting materials were bought from commercial suppliers (*e.g.,* Sigma-Aldrich, Fisher Scientific, Alfa Aesar and Acros Organics) and used without further purification unless indicated otherwise.

Room temperature (rt) means 18–25 °C and was regulated using an oil bath. Reactions were stirred magnetically using a temperature-regulated hotplate/stirrer and monitored by thin-layer chromatography (TLC), reverse-phase liquid chromatography–mass spectrometry (LCMS) or nuclear magnetic resonance spectroscopy (NMR) where appropriate.

1.1. Thin-Layer Chromatography (TLC)

Thin-layer chromatography (TLC) was performed using aluminum-backed silica plates (Merck Keiselgel 60 F_{254}) and visualised by UV light (λ = 254–312 nm) and/or by staining with *p*-anisaldehyde, phosphomolybdic acid, potassium permanganate or vanillin solutions, which were subsequently heat-treated.

1.2. Chromatography

Flash column chromatography was carried out using Merck silica gel 60 (40–63 µm, 230–400 mesh). Preparative Thin Layer Chromatography (PTLC) was carried out using Merck Analtech 01012, 20 cm × 20 cm, 500 µm.

1.3. Solvents

The following anhydrous solvents were obtained from the University of Edinburgh School of Chemistry's communal solvent purification system, which were purified by filtration through activated alumina columns: acetonitrile (MeCN), dichloromethane (CH_2Cl_2), diethyl ether (Et_2O), *n*-hexane, tetrahydrofuran (THF) and toluene. These solvents were transferred, stored, and used under a positive pressure of nitrogen in Young's valve-sealed, oven-dried and nitrogen-purged Strauss flasks containing activated 3 Å molecular sieves (~20% w/v). The following anhydrous solvents were purchased and used as received from Acros Organics: 1,4-dioxane [CAS: 123-91-1], *N*,*N*-dimethylformamide (DMF) [CAS: 68-12-2], methanol (MeOH) [CAS: 67-56-1], ethanol [CAS: 64-17-5] and 2-methyltetrahydrofuran (2-MeTHF) [CAS: 96-47-9]. Solvents for non-anhydrous reactions or purifications were used as received.

1.4. Analysis

Nuclear Magnetic Resonance (NMR) data (^1H, ^{13}C, HSQC, HMBC, COSY) were recorded using a Bruker Avance 600 MHz, Bruker Avance 500 MHz or Bruker Pro 500 MHz NMR spectrometer. Data were recorded at 298 K unless specified and all ^{13}C NMR spectra were broadband ^1H decoupled. Deuterated solvents ($CDCl_3$, CD_3OD) were used as obtained from Sigma-Aldrich. Chemical shifts (δ / ppm) are reported relative to the solvent's reference peaks (*e.g.,* ^1H - $CDCl_3$, 7.26 ppm; ^{13}C - $CDCl_3$, 77.16 ppm). Data for ^1H NMR spectra are reported as follows: chemical shift (δ / ppm), peak multiplicity (s, singlet; d, doublet; t, triplet; q, quartet; p, pentet; m, multiplet or unresolved, *br* s, broad singlet), integration, coupling constants (Hz) and peak assignment. Coupling constants (*J*) are quoted to the nearest 0.1 Hz.

High Resolution Mass Spectrometry (HRMS) data were recorded by the University of Edinburgh Mass Spectrometry Services Laboratory using electrospray ionisation (ESI; Bruker Daltonics micrOTOF II) calibrated with sodium formate clusters with data analysis using Data Analysis 4.1 (Bruker Daltonics).

Fourier Transformed Infra-Red (FTIR) spectra were obtained using a Shimadzu IR Affinity-1 FTIR spectrometer loading compounds as thin films or solids. Select peak values were quoted in wave numbers (cm^{-1}); only characteristic functional group absorption maxima (ν_{max}) are reported.

High-Performance Liquid Chromatography (HPLC) was conducted using a modular Shimadzu system using a LC-20AD pump, DGU-20A5R degassing unit, SIL-20A HT autosampler, CTO-20A column oven, SPD-20A UV/Vis detector and CDM-20A communications module.

Optical Rotation ($[\alpha]_D$ values) were recorded using a Bellingham and Stanley Ltd. ADP 450 polarimeter with a Bellingham and Stanley Ltd. 0.5 mL cell (l = 0.25 dm). Concentrations (c) are reported in g/100 mL.

2. Stereoretentive Enantioconvergent *Aza*-Darzens Dimerization

2.1. Synthesis of Racemic Aziridine

Trisubstituted aziridine ((*rac*)-2)[20]

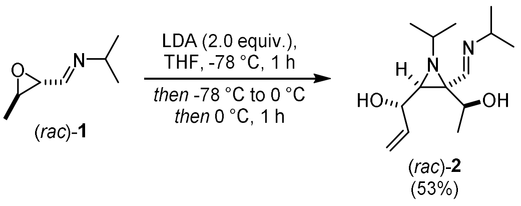

Based on conditions reported by Würthwein and co-workers.[20] To a stirred solution of freshly distilled *N,N*-diisopropylamine (0.22 mL, 1.58 mmol, 2.00 equiv.) in anhydrous THF (1.00 mL) under a N_2 atmosphere was added *n*-butyllithium (0.94 mL, 1.60 M in hexanes, 1.58 mmol, 2.00 equiv.) at −78 °C dropwise over 10 sedconds. The resulting colourless solution was allowed to stir at this temperature for 0.5 h. A solution of imine **1**[20] (100 mg, 0.79 mmol, 1.00 equiv) in anhydrous THF (1.00 mL) was then added dropwise over 15 seconds at −78 °C. The resulting brown solution was stirred at this temperature for 1 h before being allowed to warm gradually to 0 °C (*ca.* 90 mins) and then stirred at this temperature for a further 1 h. The reaction mixture was then quenched with water (5 mL) and extracted into CH_2Cl_2 (3 × 5 mL). The combined organic extracts were washed with brine (10 mL), dried over anhydrous Na_2SO_4, filtered, and concentrated under reduced pressure. The resulting orange oil was then purified by column chromatography on silica gel (75% Et_2O in *n*-hexane), affording allylic alcohol **2** (53 mg, 0.21 mmol, 53%) as a colourless crystalline solid. Spectroscopic data were in agreement with those reported previously.[20]

MP: 127–128 °C (*n*-hexane/Et_2O) (Lit: 128 °C)[20]

***R*f** = 0.35 (75% Et_2O in *n*-hexane), stained with $KMnO_4$;

^{1}H NMR (601 MHz, $CDCl_3$) δ = 7.47 (d, *J* = 1.6 Hz, 1H), 6.04 (ddd, *J* = 17.2, 10.6, 4.9 Hz, 1H), 5.41 (dt, *J* = 17.3, 1.6 Hz, 1H), 5.20 (dt, *J* = 10.6, 1.6 Hz, 1H), 4.59 (d, *J* = 8.4 Hz, 1H), 4.25 – 4.19 (m, 1H), 3.66 (p, *J* = 6.9 Hz, 1H), 3.32 (hept, *J* = 6.3 Hz, 1H), 2.56 (hept, *J* = 6.1 Hz, 1H), 2.45 (d, *J* = 4.2 Hz, 1H), 2.13 (d, *J* = 6.3 Hz, 1H), 1.41 (d, *J* = 6.5 Hz, 3H), 1.19 (d, *J* = 6.4 Hz, 3H), 1.18 (d, *J* = 6.4 Hz, 3H), 1.17 (d, *J* = 6.3 Hz, 3H), 1.05 (d, *J* = 6.2 Hz, 3H);

^{13}C NMR (150 MHz, $CDCl_3$) δ = 158.8, 138.7, 115.5, 69.3, 69.2, 62.0, 55.0, 52.2, 50.2, 24.2, 24.0, 23.0, 22.1, 20.0;

HRMS (ESI$^+$): Calc. for $C_{14}H_{27}N_2O_2$ [M+H]$^+$: 255.2067; found: 255.2072;

HPLC: Daicel Chiralpak® ID column with guard, 100% *n*-hexane, flow 0.8 mL/min, 30 °C, 230 nm, t_R = 9.53 min, t_R = 10.31 min.

Note: Using fewer than 2.00 equiv. of LDA resulted in the formation of separable mixtures of allylic alcohol (**2**) and epoxide (**S1**), which was isolated as a yellow oil following purification by column chromatography on silica gel (75% Et_2O in *n*-hexane).

Intermediate epoxide (S1)

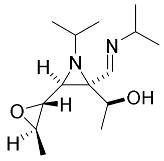

S1

***R*f** = 0.45 (75% Et_2O in *n*-hexane); stained with $KMnO_4$;

^{1}H NMR (400 MHz, $CDCl_3$) δ = 7.47 (s, 1H), 4.26 (s, 1H), 3.93 (q, *J* = 6.4 Hz, 1H), 3.33 (h, *J* = 6.8 Hz, 1H), 3.01 (qd, *J* = 5.2, 2.2 Hz, 1H), 2.84 (dd, *J* = 6.6, 2.3 Hz, 1H), 2.49 (hept, *J* = 6.2 Hz, 1H), 1.87 (d, *J* = 6.6 Hz, 1H), 1.33 (dd, *J* = 6.7, 5.8 Hz, 6H), 1.20 (d, *J* = 6.2 Hz, 3H), 1.18 (d, *J* = 6.3 Hz, 3H), 1.15 (d, *J* = 6.3 Hz, 3H), 1.02 (d, *J* = 6.2 Hz, 3H);

^{13}C NMR (101 MHz, $CDCl_3$) δ = 158.1, 67.6, 62.0, 57.4, 53.6, 52.1, 50.2, 50.1, 24.2, 24.0, 22.7, 22.0, 19.8, 17.3;

HRMS (ESI$^+$): Calc. for $C_{14}H_{26}N_2O_2$ [M+H]$^+$: 255.2067; found: 255.2076.

2.2. General Procedure for Chiral Lithium Amides

Chiral amines **4** and **S6** were commercially available. Bases **S2**[39], **S3**[40], **S4**[41] and **S5** were prepared as previously reported.

To a stirred solution of the required chiral amine (1.58 mmol, 2.00 equiv) in anhydrous THF (1.00 mL) under a N_2 atmosphere and cooled to −80 °C with the aid of a cryostat, was added *n*-BuLi (0.98 mL, 1.60 M in hexanes, 1.58 mmol, 2.00 equiv) dropwise over 10 seconds. The resulting solution/suspension was held at this temperature for 45 minutes and a colour change was generally observed. The temperature on the cryostat was then adjusted to the reaction temperature and the reaction mixture was allowed to equilibrate for 10 minutes. A solution of the imine (100 mg, 0.79 mmol, 1.00 equiv) in anhydrous THF (1.00 mL) was then added dropwise over *ca*. 15 seconds. The resulting solution was allowed to stir for 1.5 h before being gradually warmed to 0 °C by adjusting the temperature on the cryostat (typically over 90–120 min). The resulting solution was then stirred at 0 °C for a further 90 min before being quenched with water (5 mL) and extracted in CH_2Cl_2 (3 × 5 mL). The combined organic extracts were washed with brine (10 mL), dried over anhydrous Na_2SO_4, filtered, and concentrated under reduced pressure. The resulting orange oil was then purified by column chromatography on silica gel (75% Et_2O in *n*-hexane), affording allylic alcohol **2** as a colourless crystalline solid. The resulting solid was then analysed by HPLC on chiral stationary phase to determine the enantioselectivity of the reaction.

Fig. 1. Screening of chiral lithium amides and their resultant enantioselectivities.

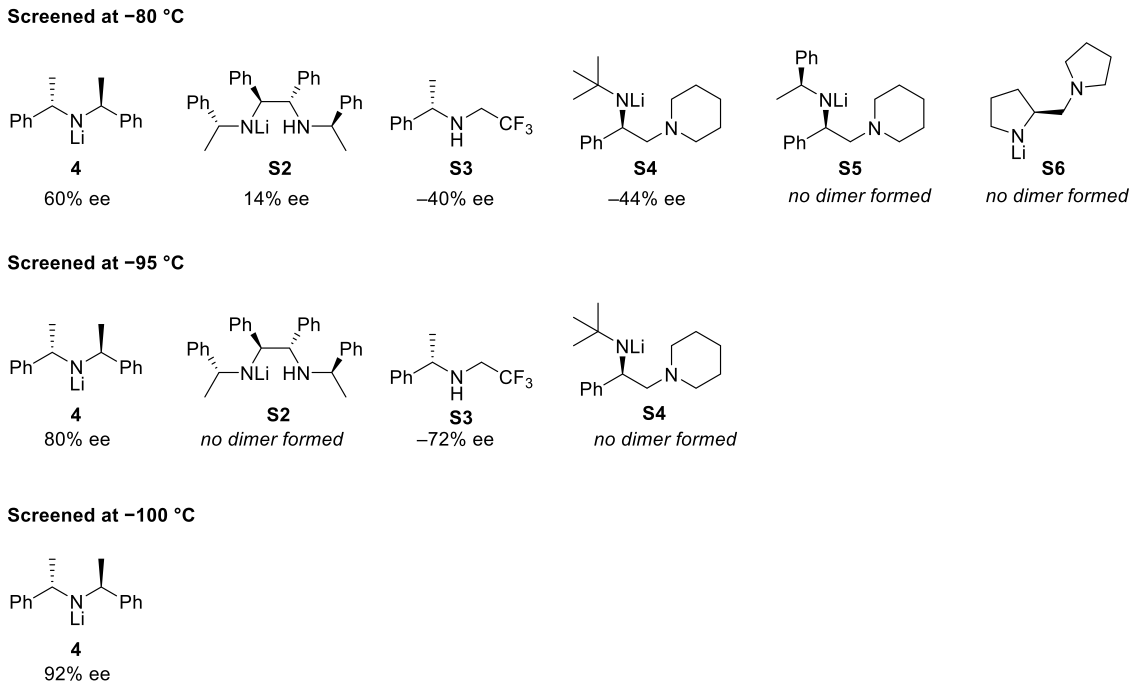

2.3. Stereoretentive Enantioconvergent Aza-Darzens Reaction

Enantioenriched trisubstituted aziridine (2)

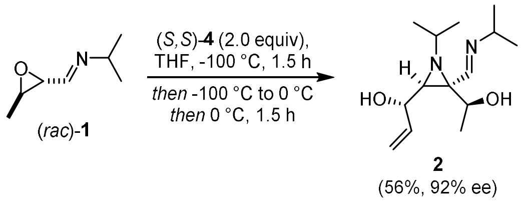

To a stirred solution of (*S,S*)-**4** (338 mg, 1.58 mmol, 2.00 equiv) in anhydrous THF (1.00 mL) under a N_2 atmosphere and cooled to −80 °C with the aid of a cryostat, was added *n*-BuLi (0.98 mL, 1.60 M in hexanes, 1.58 mmol, 2.00 equiv) dropwise over 10 seconds. The resulting solution was held at this temperature for 45 minutes and gradually turned deep purple. The temperature on the cryostat was adjusted to −100 °C and the mixture was allowed to equilibrate for 10 minutes. At this point, the chiral lithium amide had precipitated as a lavender solid. A solution of the imine (100 mg, 0.79 mmol, 1.00 equiv) in anhydrous THF (1.00 mL) was then added dropwise over *ca*. 15 seconds. The resulting suspension was allowed to stir for 1.5 h before being gradually warmed to 0 °C by adjusting the temperature on the cryostat (typically over 90–120 min). The resulting solution was then stirred at 0 °C for a further 90 min before being quenched with water (5 mL) and extracted in CH_2Cl_2 (3 × 5 mL). The combined organic extracts were washed with brine (10 mL), dried over anhydrous Na_2SO_4, filtered, and concentrated under reduced pressure. The resulting orange oil was then purified by column chromatography on silica gel (75% Et_2O in *n*-hexane), affording allylic alcohol **2** (57 mg, 0.22 mmol, 56%, 92% ee) as a colourless solid.

HPLC: Daicel Chiralpak® ID column with guard, 100% *n*-hexane, 0.8 ml/min, 30 °C, 230 nm, t_R = 9.22 min (Major), t_R = 10.13 min (Minor), e.r. 96:4 (92% ee).

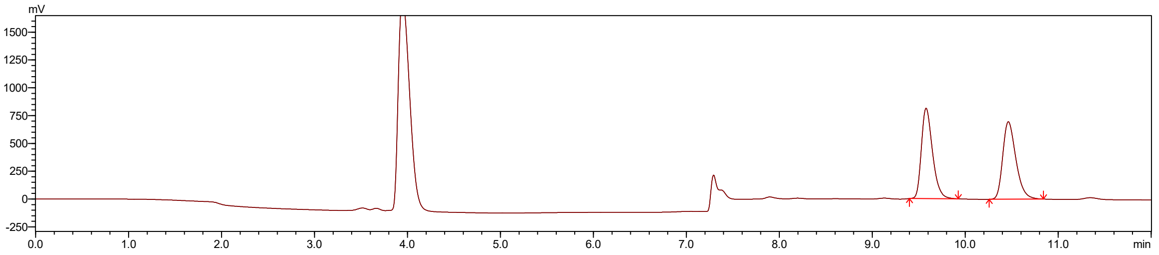

Peak#	Ret. Time	Area	Height	Conc.	Area%
1	9.578	6744916	812661	49.695	49.695
2	10.463	6827845	696419	50.305	50.305
Total		13572762	1509080	100.000	100.000

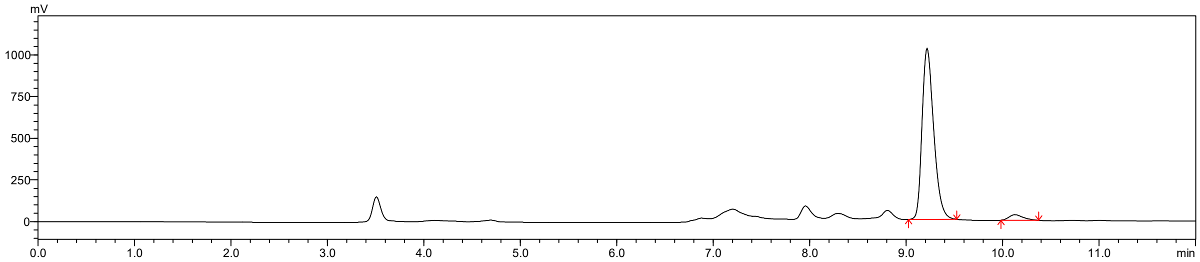

Peak#	Ret. Time	Area	Height	Conc.	Area%
1	9.216	8682285	1027816	96.203	96.203
2	10.127	342721	33375	3.797	3.797
Total		9025005	1061190	100.000	100.000

3. Stereoretentive Enantioconvergent Reaction with a Traceless Auxiliary

3.1. Synthesis of Racemic α-Aminoamides

Chloroamide ((*rac*)-10)

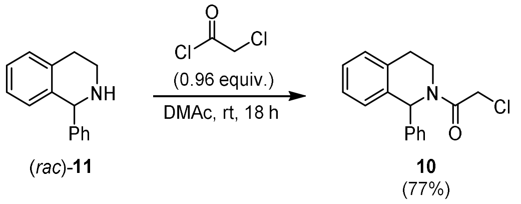

Amine (*rac*)-**11** (300 mg, 1.44 mmol, 1.00 equiv.) was dissolved in DMAc (2.00 mL) and added dropwise to a solution of chloroacetyl chloride (0.11 mL, 1.38 mmol, 0.96 equiv.) in DMAc (1.60 mL) at 0 °C over 5 min. The reaction mixture was allowed to stir for 18 h at ambient temperature. Water (15 mL) and aqueous 2 M HCl (0.80 mL) were added. The mixture was extracted with EtOAc:*n*-hexane (2:1, 4 × 10 mL) and the combined organic phases were washed with water (2 × 25 mL). The organic phases were dried over anhydrous Na_2SO_4, filtered, and concentrated under reduced pressure. The light orange oil was purified using column chromatography (50% EtOAc in *n*-hexane) to give chloroamide (*rac*)-**10** (302 mg, 1.06 mmol, 77%) as an off-white foam.

R_f = 0.53 (50% EtOAc in *n*-Hexane);

^{1}H NMR† (500 MHz, $CDCl_3$) δ = 7.41 – 7.17 (m, 9H), 6.87 (s, 1H), 4.31 – 4.07 (m, 2H), 3.90 – 3.76 (m, 1H), 3.55 (ddd, *J* = 13.6, 11.4, 4.3 Hz, 1H), 3.15 (ddd, *J* = 16.9, 11.3, 5.9 Hz, 1H), 2.88 (dt, *J* = 16.3, 3.7 Hz, 1H);

^{13}C NMR (126 MHz, $CDCl_3$) δ = 165.4, 142.0, 134.8, 134.1, 129.0, 128.9 (2 × overlapping signals), 128.5, 127.8, 127.4, 126.6, 55.9, 41.6, 40.4, 29.1;

IR (thin film, cm^{-1}): 1644, 1449, 1433, 742, 700;

HRMS (ESI$^+$): Calc. for $C_{17}H_{17}ClNO$ $[M+H]^+$: 286.0993; found 286.0999

†**Notes:** Chloroamide **10** exists as a pair of rotamers in $CDCl_3$ at rt (rotameric ratio = 4:1). NMR data for the major rotamer are reported.

Analytical and spectroscopic data matched those previously reported.[42]

α-Aminoamide dimers ((*rac*)-12)

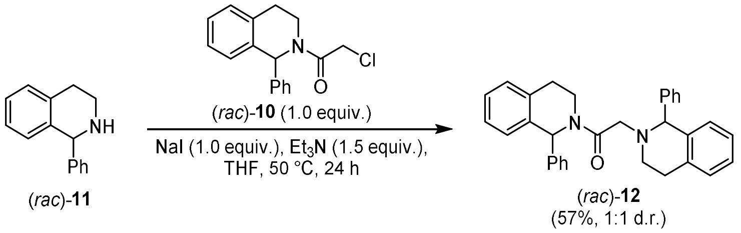

To a stirred solution of (*rac*)-chloroamide **10** (120 mg, 0.42 mmol, 1.00 equiv.) and (*rac*)-amine **11** (88 mg, 0.42 mmol, 1.00 equiv.) in anhydrous THF (2.00 mL) was added Et_3N (88 µL, 0.63 mmol, 1.50 equiv.) and NaI (63 mg, 0.42 mmol, 1.00 equiv.). The resulting orange solution was stirred at 50 °C for 24 h, after which time the solvent was removed under reduced pressure, giving a yellow foam, which was dissolved in CH_2Cl_2 (20 mL). The organic phase was washed with water (10 mL), brine (10 mL), dried over anhydrous Na_2SO_4, filtered, and concentrated under reduced pressure to give a yellow residue that was purified by column chromatography on silica gel (70% EtOAc in *n*-hexane) to afford α-aminoamide dimers (*rac*)-**12** (109 mg, 0.24 mmol, 57%, 1:1 d.r.) as a white foam.

Spectroscopic data for the homochiral diastereomer are reported on page 16, while spectroscopic data for the heterochiral diastereomer are reported on page 21.

HPLC: Daicel Chiracel® OD-H column with guard, 2% *i*-PrOH in *n*-hexane, 1.0 mL/min, 30 °C, 270 nm, t_R = 20.89 min (*R,S-isomer*), t_R = 24.78 min (*S,R-isomer*), t_R = 33.78 min (*S,S-isomer*), t_R = 39.20 min (*R,R-isomer*).

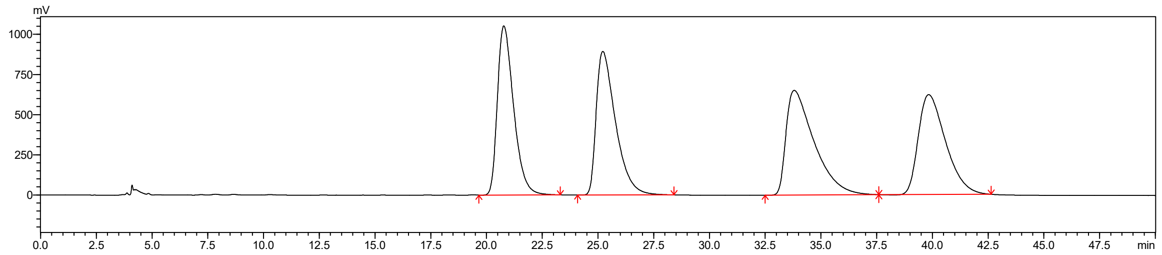

Peak#	Ret. Time	Area	Height	Conc.	Area%
1	20.776	53450199	1053584	24.576	24.576
2	25.217	53886522	893886	24.777	24.777
3	33.806	57147975	652570	26.276	26.276
4	39.834	53003409	622150	24.371	24.371
Total		217488105	3222189	100.000	100.000

3.2. HPLC Trace Assignment of Dimeric α-Aminoamides

Enantiopure chloroamide ((*S*)-10)

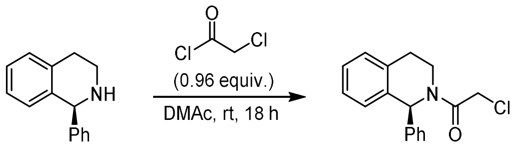

(*S*)-**11** (*S*)-**10** (63%)

Prepared in analogy to the procedure in **Section 3.1** that used racemic (*rac*)-**11**. Chloroamide (*S*)-**10** (1.11 g, 3.88 mmol, 63%) was obtained as an off-white foam.

Spectroscopic and analytical data were identical to the racemic material.

HPLC: Daicel Chiracel OD-H column with guard, 2% *i*-PrOH in *n*-hexane, flow 1.0 mL/min, 30 °C, 270 nm, t_R = 10.90 min (*S-isomer*), t_R = 14.03 min (*R-isomer*), >99% ee.

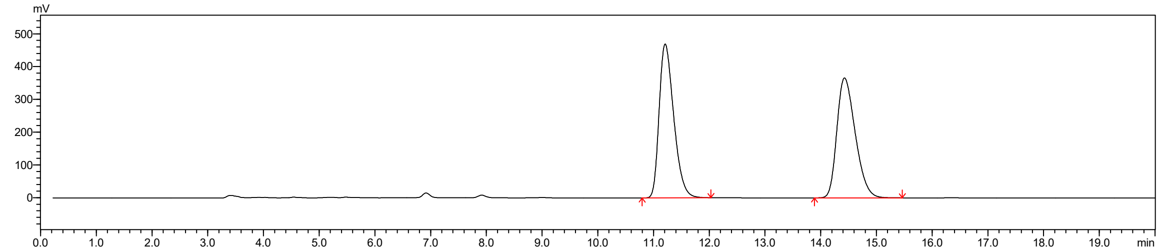

Peak#	Ret. Time	Area	Height	Conc.	Area%
1	10.987	8544950	469496	50.435	50.435
2	14.206	8397461	366026	49.565	49.565
Total		16942411	835522	100.000	100.000

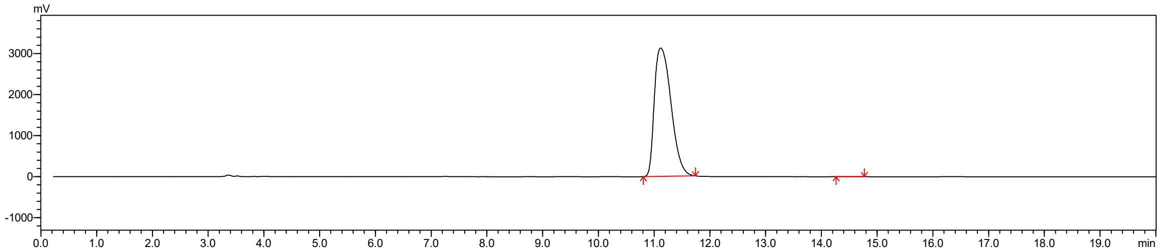

Peak#	Ret. Time	Area	Height	Conc.	Area%
1	10.896	65181081	3128370	99.924	99.924
2	14.288	49346	2866	0.076	0.076
Total		65230427	3131235	100.000	100.000

Homochiral α-aminoamide ((*S*,*S*)-12)) and heterochiral α-aminoamide ((*S*,*R*)-12))

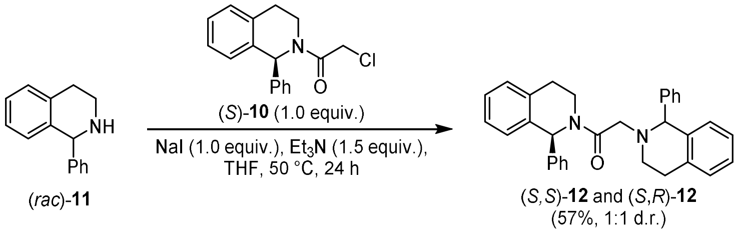

To a stirred solution of chloroamide (*S*)-**10** (120 mg, 0.42 mmol, 1.00 equiv.) and (*rac*)-amine **11** (88 mg, 0.42 mmol, 1.00 equiv.) in anhydrous THF (2.00 mL) was added Et_3N (88 μL, 0.63 mmol, 1.50 equiv.) and NaI (63 mg, 0.42 mmol, 1.00 equiv.). The resulting orange solution was stirred at 50 °C for 24 h, after which time the solvent was removed under reduced pressure, giving a yellow foam, which was dissolved in CH_2Cl_2 (20 mL). The organic phase was washed with water (10 mL), brine (10 mL), dried over anhydrous Na_2SO_4, filtered, and concentrated under reduced pressure giving a yellow residue that was purified by column chromatography on silica gel (70% EtOAc in *n*-hexane) to afford α-amimoamide dimers (*S*,*S*)- and (*S*,*R*)-**12** dimers (110 mg, 0.24 mmol, 57%, 1:1 d.r.) as a white foam.

Spectroscopic data for the homochiral diastereomer are reported on page 16, while spectroscopic data for the heterochiral diastereomer are reported on page 21.

HPLC: Daicel Chiracel OD-H column with guard, 2% *i*-PrOH in *n*-hexane, 1.0 mL/min, 30 °C, 270 nm, t_R = 25.79 min (*S,R-isomer*), t_R = 34.67 min (*S,S-isomer*). See page 17 for HPLC traces.

Homochiral α-aminoamide ((*S*,*S*)-12 and heterochiral α-aminoamide ((*R*,*S*)-12)

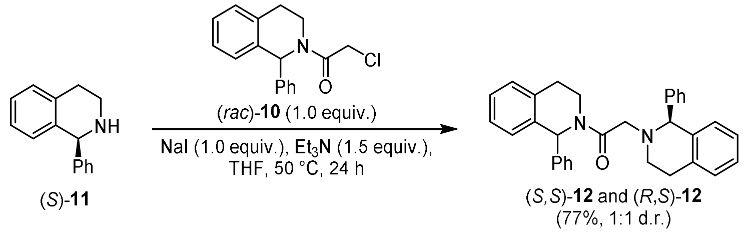

To a stirred solution of (*rac*)-chloroamide **10** (120 mg, 0.42 mmol, 1.00 equiv.) and amine (*S*)-**11** (88 mg, 0.42 mmol, 1.00 equiv.) in anhydrous THF (2.00 mL), was added Et_3N (88 μL, 0.63 mmol, 1.50 equiv.) and NaI (63 mg, 0.42 mmol, 1.00 equiv.). The resulting orange solution was stirred at 50 °C for 24 h, after which time the solvent was removed under reduced pressure, giving a yellow foam, which was dissolved in CH_2Cl_2 (20 mL). The organic phase was washed with water (10 mL), brine (10 mL), dried over anhydrous Na_2SO_4, filtered, and concentrated under reduced pressure giving a yellow residue that was purified by column chromatography on silica gel (70% EtOAc in *n*-hexane) to afford α-amimoamide dimers (*S*,*S*)- and (*R*,*S*)-**12** (148 mg, 0.32 mmol, 77%, 1:1 d.r.) as a white foam.

Spectroscopic data for the homochiral diastereomer are reported on page 16, while spectroscopic data for the heterochiral diastereomer are reported on page 21.

HPLC: Daicel Chiracel® OD-H column with guard, 2% *i*-PrOH in *n*-hexane, 1.0 mL/min, 30 °C, 270 nm, t_R = 20.92 min (*R,S-isomer*), t_R = 33.01 min (*S,S-isomer*). See page 17 for HPLC traces.

Homochiral α-aminoamide ((*S*,*S*)-12)

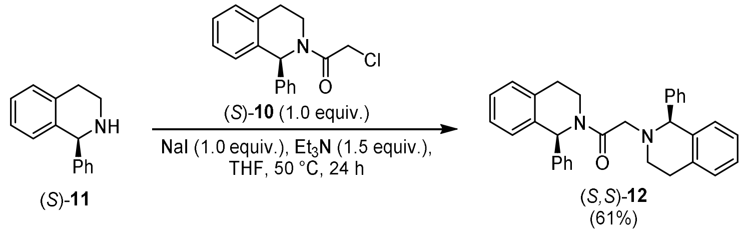

Prepared in analogy to the procedure above that used racemic (*rac*)-**11**. Chloroamide (*S*)-**10** (120 mg, 0.42 mmol, 1.00 equiv), amine (*S*)-**11** (88 mg, 0.42 mmol, 1.00 equiv.). Homodimer (*S*,*S*)-**12** (117 mg, 0.26 mmol, 61%) was isolated as a white foam following purification by column chromatography on silica gel (70% EtOAc in *n*-hexane).

***R*f** = 0.64 (70% EtOAc in *n*-hexane);

^{1}H NMR† (601 MHz, $CDCl_3$) δ = 7.40 – 6.96[^,*] (m, 18H, ArC**H**), 6.87[^] (s, 1H, O=CNC**H**Ph), 6.74 – 6.53[^,*] (m, 2H), 4.63[^] (s, 1H), 4.46[*] (s, 1H), 4.36 – 4.24[*] (m, 1H), 3.85[*] (d, *J* = 12.9 Hz, 1H), 3.72[^] (dt, *J* = 14.1, 4.4 Hz, 1H), 3.45[^] (d, *J* = 13.5 Hz, 1H), 3.29[^] (ddd, *J* = 13.8, 10.0, 5.7 Hz, 1H), 3.20[*] (ddd, *J* = 13.2, 9.7, 5.0 Hz, 1H), 3.11[^,*] (d, *J* = 12.9 Hz, 2H), 3.06 – 2.90[^,*] (m, 2H), 2.86 – 2.50[^,*] (m, 3H);

^{13}C NMR† (151 MHz, $CDCl_3$) δ = 169.0^, 143.4^, 142.9*, 142.6^, 141.4*, 138.1^,*, 136.1*, 135.1^, 135.0^, 134.7*, 134.6*, 134.5*, 130.1^, 129.9*, 128.9^, 128.81*, 128.79*, 128.6^, 128.5^, 128.4*, 128.24^, 128.20^, 128.1*, 127.8*, 127.8^, 127.74^, 127.70*, 127.3^, 127.2*, 127.1*, 126.9^, 126.2*, 126.1^, 126.0*, 125.7*, 125.7^, 69.9*, 68.8^, 59.8^, 58.4^, 58.2*, 55.2^, 48.8*, 48.5^, 39.3^, 37.1*, 29.23*, 29.15^, 28.7^, 27.8*;

IR (Thin film, cm^{-1}): 1637, 1492, 1450, 1438, 742, 698;

HRMS (ESI^{+}): calc. for $C_{32}H_{31}N_2O$ $[M+H]^+$: 459.2431; found: 459.2418;

HPLC: Daicel Chiracel® OD-H column with guard, 2% *i*-PrOH in *n*-hexane, 1.0 mL/min, 30 °C, 270 nm, t_R = 33.42 min (*S,S-isomer*), >99% ee.

†**Notes:** Dimer (*S,S*)-**12** exists as a mixture of rotamers in solution (rotameric ratio = 1:1.2). Rotamers are assigned as ^major or *minor where possible. Amide C=O not observed for minor rotamer. Several signals from the major and minor rotamers overlap in the ^{13}C NMR spectrum.

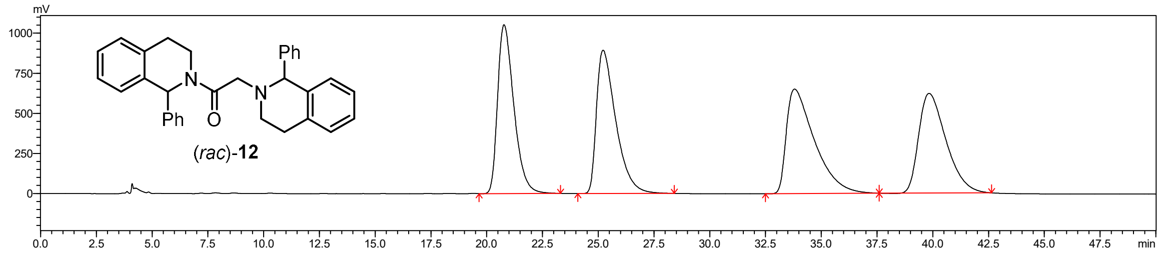
mV
(rac)-12
min

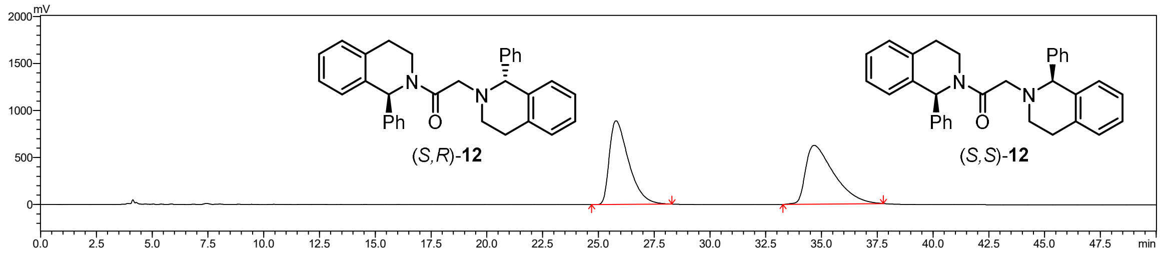
mV
(S,R)-12
(S,S)-12
min

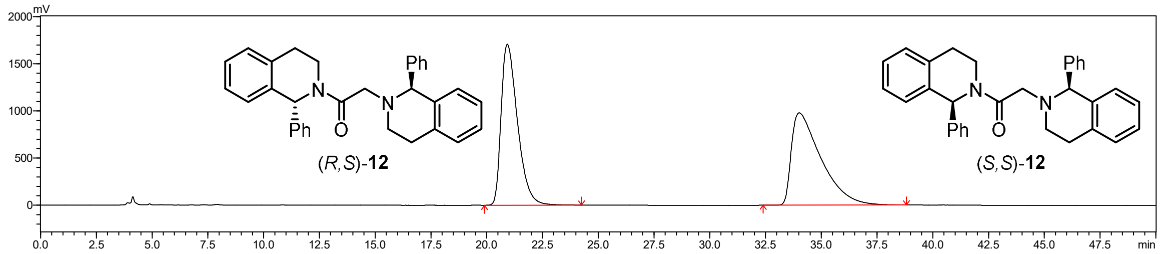
mV
(R,S)-12
(S,S)-12
min

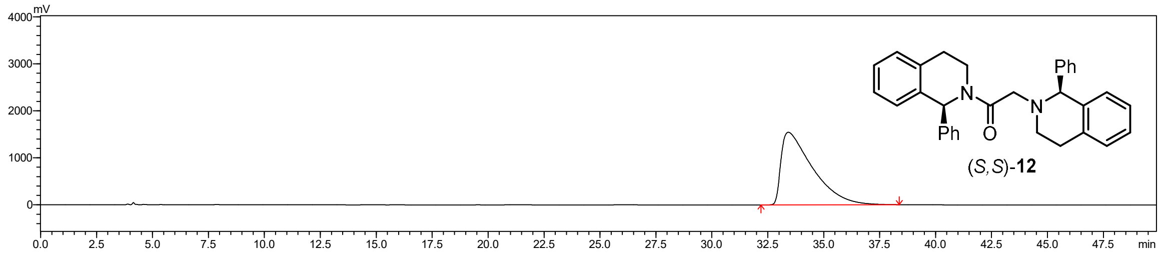
mV
(S,S)-12
min

3.3.Stereoretentive Enantioconvergent Traceless Auxillary Reaction

Hydroxamic α-chloroacetate (9)

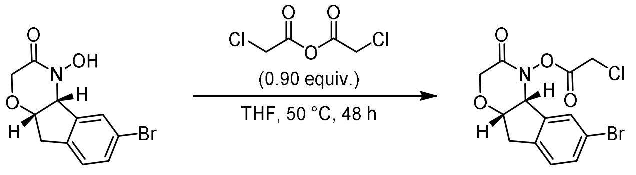

S7 **9** (71%)

To a stirring solution of commercially available hydroxamic acid **S7** (100 mg, 0.52 mmol, 1.00 equiv.) in THF (1.60 mL) was added chloroacetic anhydride (78 mg, 0.46 mmol, 0.90 equiv.). The reaction was heated at 50 °C for 24 h, after which time, an additional portion of chloroacetic anhydride (38 mg, 0.22 mmol) was added and the reaction was left for a further 24 h. The reaction mixture was concentrated under reduced pressure and the resulting off-white solid was dissolved in CH_2Cl_2 (30 mL) and washed with saturated aqueous $NaHCO_3$ (4 × 15 mL) and brine (2 × 15 mL). The organic phases were dried over anhydrous Na_2SO_4, filtered, and concentrated under reduced pressure to afford hydroxamic α-chloroacetate **9** (89 mg, 0.25 mmol, 71% yield) as a white solid. The compound was used directly in the subsequent kinetic resolution without further purification as it was found to degrade readily. A small portion was analysed by ^{1}H NMR spectroscopy to identify characteristic signals.

^{1}H NMR (601 MHz, $CDCl_3$) δ = 7.68 (d, *J* = 1.8 Hz, 1H), 7.48 (ddd, *J* = 8.1, 1.9, 0.8 Hz, 1H), 7.20 (d, *J* = 8.0 Hz, 1H), 5.10 (d, *J* = 4.4 Hz, 1H), 4.82 (td, *J* = 4.7, 1.2 Hz, 1H), 4.39 (s, 2H), 4.37 (d, *J* = 7.8 Hz, 2H), 3.31 – 2.98 (m, 2H);

Kinetic resolution of secondary amine (*rac*)-11

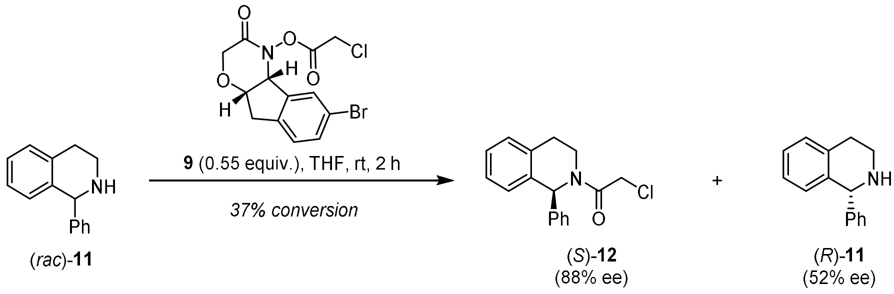

To a stirred solution of (*rac*)-amine **11** (45 mg, 0.20 mmol, 1.00 equiv.) in anhydrous THF (1.00 mL) was added hydroxamic α-chloroacetate **9** (40 mg, 0.11 mmol, 0.55 equiv.) in a single portion. The resulting solution was stirred at room temperature for 2 h. An aliquot of the reaction mixture was then collected and analysed by chiral stationary phase HPLC.

HPLC: Daicel Chiracel® OD-H column with guard, 5% *i*-PrOH in *n*-hexane, 0.5 mL/min, 30 °C, 230 nm, t_R = 16.12 min ((*S*)-**11**), t_R = 26.79 min ((*R*)-**11**), t_R = 33.72 min ((*S*)-**12**), t_R = 45.29 min ((*R*)-**12**).

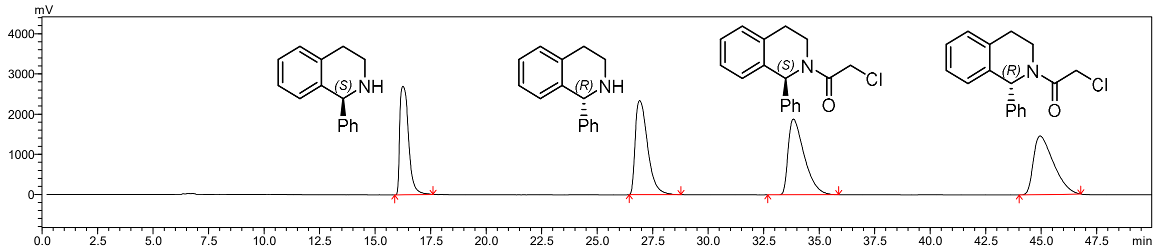

Peak#	Ret. Time	Area	Height	Conc.	Area%
1	16.031	74335226	2702020	22.186	22.186
2	26.691	71820312	2172525	21.435	21.435
3	33.618	95323986	1888830	28.450	28.450
4	44.729	93578202	1468252	27.929	27.929
Total		335057727	8231628	100.000	100.000

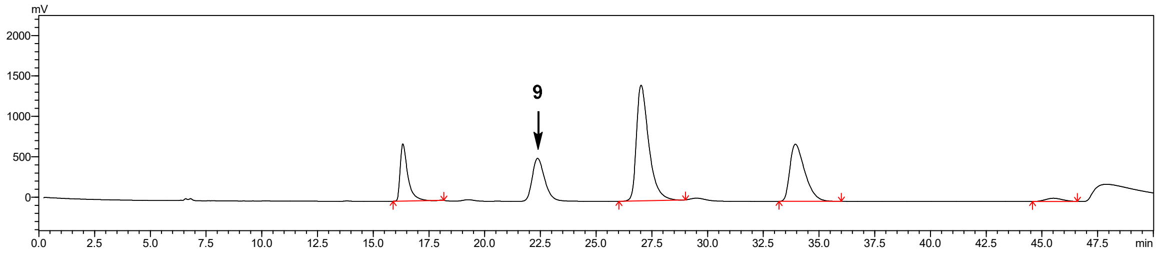

Peak#	Ret. Time	Area	Height	Conc.	Area%
1	16.109	16873432	707806	15.818	15.818
2	26.795	54698554	1426555	51.278	51.278
3	33.719	32987240	707618	30.925	30.925
4	45.295	2110816	39569	1.979	1.979
Total		106670042	2881548	100.000	100.000

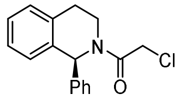

(*S*)-**12**
(88% ee)

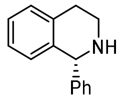

(*R*)-**11**
(52% ee)

Conversion determination by HPLC

$$Conv. = \frac{ee_{\mathbf{11}}}{(ee_{\mathbf{11}} + ee_{\mathbf{12}})} \times 100 = \frac{0.52}{(0.52 + 0.88)} \times 100 = \mathbf{37}\%$$

s-factor determination

$$s = \frac{\ln[(1 - conv.)(1 - ee_{\mathbf{11}})]}{\ln[(1 - conv.)(1 + ee_{\mathbf{11}})]} = \frac{\ln[(1 - 0.37)(1 - 0.52)]}{\ln[(1 - 0.37)(1 + 0.52)]} = \mathbf{28}$$

Heterochiral α-aminoamide ((*S*,*R*)-12)

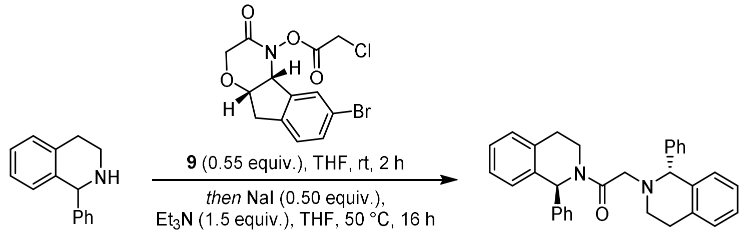

(*rac*)-**11**

(*S*,*R*)-**12**
(>95%, 97% ee, 6:1 d.r.)

To a stirred solution of (*rac*)-amine **11** (45 mg, 0.20 mmol, 1.00 equiv.) in anhydrous THF (1.00 mL) was added hydroxamic α-chloroacetate **9** (40 mg, 0.11 mmol, 0.55 equiv.) in a single portion. The resulting solution was stirred at room temperature for 2 h before anhydrous Et_3N (41 μL, 0.30 mmol, 1.50 equiv.) and NaI (15 mg, 0.10 mmol, 0.50 equiv.) were added in a single portion. The resulting mixture was heated to 50 °C for 16 h. Upon cooling to room temperature, the reaction mixture was concentrated under reduced pressure and ^{1}H NMR analysis indicated complete consumption of both starting materials and the formation of the dimer (>95%, 97% ee, 6:1 d.r., determined by HPLC). Purification by preparative TLC (50% EtOAc in *n*-hexane) afforded the dimer (39 mg, 0.085 mmol, 85%, 98% ee, 9:1 d.r.) as a white foam, slightly enriched in the major diastereomer. Further purification by preparative TLC, afforded a sample of pure heterochiral dimer (*ca.* 6 mg) for characterisation.

***R*f** = 0.68 (70% EtOAc in *n*-hexane);

^{1}H NMR† (601 MHz, $CDCl_3$): δ = 7.34–6.98[^,*] (m, 17H), 6.92[^] (s, 1H, O=CNC**H**Ph), 6.91[*] (s, 1H, O=CNC**H**Ph), 6.65[^] (d, *J* = 8.0 Hz, 1H, ArC**H**), 6.58[*] (d, *J* =8.0 Hz, 1H, ArC**H**), 6.02[*] (s, 1H, CH_2NC**H**Ph), 6.58[^] (s, 1H, CH_2NC**H**Ph), 4.52[*] (s, 2H, C**H₂**NCHPh), 4.49[^] (s, 2H, CH_2NC**H**Ph), 4.24–4.15[*] (m, 1H), 3.89[*] (d, *J* = 7.4 Hz, 1H), 3.83[*] (d, *J* = 7.4 Hz, 1H), 3.72[*] (d, *J* = 7.4 Hz, 1H), 3.59–3.54[^] (m, 2H), 3.49–3.45[*] (m, 1H), 3.25—3.12[^,*] (3H, m), 3.06–3.01[^] (m, 2H), 2.96–2.89[*] (m, 1H), 2.88–2.81[^] (m, 2H), 2.77–2.73[^] (m, 2H), 2.61 (dt, *J* = 7.3, 1.8 Hz, 1H)[*];

^{13}C NMR† (151 MHz, $CDCl_3$) δ = 160.9*, 168.9^, 143.5*, 143.4^, 142.7^, 141.8^, 138.2^, 138.1*, 136.0*, 135.2*, 135.0^, 134.8^, 134.5^, 134.4*, 130.2*, 130.0^, 129.1^, 129.0^, 128.90^, 128.86^, 128.54*, 128.48*, 128.4^, 128.31*, 128.2^, 127.6^, 127.5*, 127.4^, 127.3*, 127.2*, 126.9^, 126.5*, 126.13^, 126.11^, 126.06*, 125.9^, 125.8*, 125.7*, 69.7^, 69.6*, 59.6^, 59.5*, 55.7*, 55.2^, 49.3*, 48.8^, 39.1^, 37.9*, 29.7^, 29.5^, 29.0*, 28.5*;

HRMS (ESI$^+$): calc. for $C_{32}H_{31}N_2O$ $[M+H]^+$: 459.2431; found: 459.2420;

IR (Thin film, cm^{-1}): 1637, 1492, 1450, 1438, 742, 698;

HPLC: Daicel Chiracel® OD-H column with guard, 2% *i*-PrOH in *n*-hexane, 1.0 mL/min, 30 °C, 270 nm, t_R = 20.89 min (*R,S-isomer*), t_R = 24.78 min (*S,R-isomer*), t_R = 33.78 min (*S,S-isomer*), t_R = 39.20 min (*R,R-isomer*), 97% ee.

†**Notes:** Dimer (*S,R*)-**12** exists as a mixture of rotamers in $CDCl_3$ at rt (rotameric ratio = 1:3.7). Rotamers are assigned as ^major or *minor where possible. Amide C=O not observed for minor rotamer. Several signals for the major and minor rotamer overlap in the ^{13}C NMR spectrum.

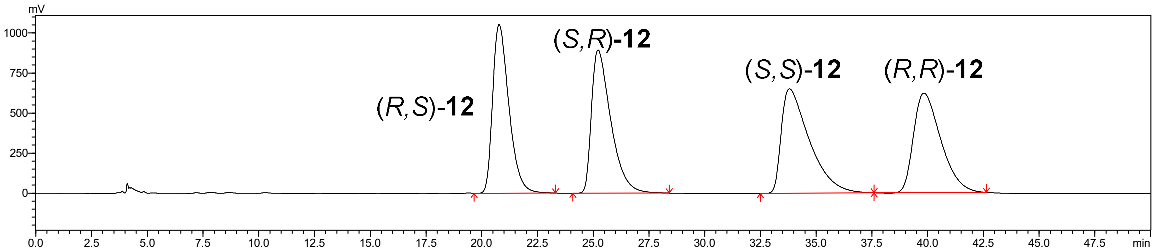

Peak#	Ret. Time	Area	Height	Conc.	Area%
1	20.776	53450199	1053584	24.576	24.576
2	25.217	53886522	893886	24.777	24.777
3	33.806	57147975	652570	26.276	26.276
4	39.834	53003409	622150	24.371	24.371
Total		217488105	3222189	100.000	100.000

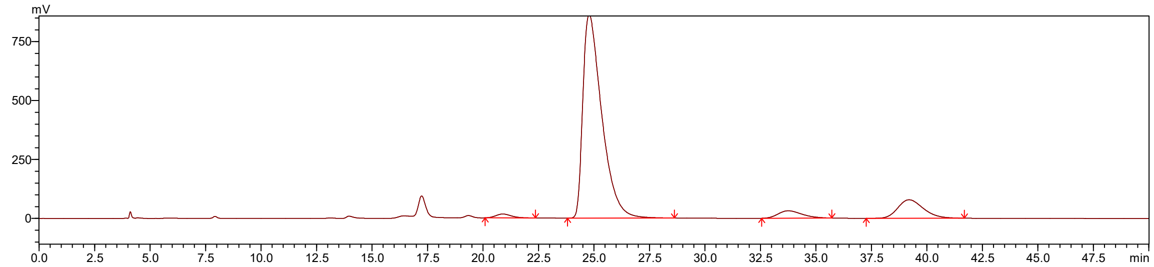

Peak#	Ret. Time	Area	Height	Conc.	Area%
1	20.894	797958	16337	1.317	1.317
2	24.783	51141260	864882	84.401	84.401
3	33.758	2372487	31459	3.915	3.915
4	39.198	6281425	78439	10.367	10.367
Total		60593130	991118	100.000	100.000

HPLC of (*S*,*R*)-12 following preparative TLC:

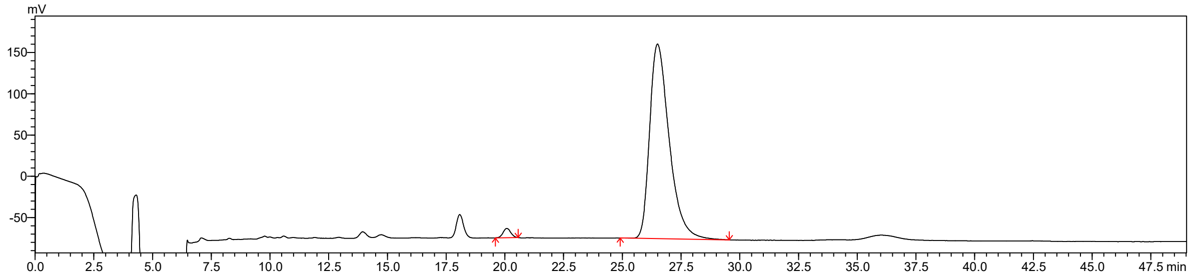

Peak#	Ret. Time	Area	Height	Conc.	Area%
1	20.077	162008	8664	1.171	1.171
2	26.497	13677996	235816	98.829	98.829
Total		13840004	244481	100.000	100.000

3. Biocatalytic Stereoretentive Enantioconvergent Reaction

3.1. Synthesis of Racemic-Homo/Heterodimer

Primary amine ((*rac*)-13)

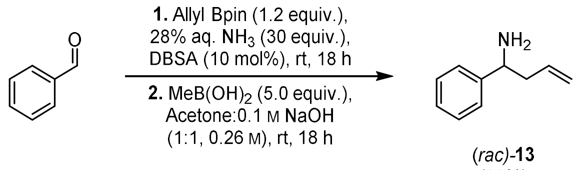

(*rac*)-**13**
(69%)

According to a procedure outlined by Kobayashi and co-workers,[43] to a 250 mL three-neck round-bottom flask was charged ammonium hydroxide (28% solution in water, 100 mL, 707 mmol, 30 equiv.) and 4-dodecylbenzenesulfonic acid (DBSA, 0.73 mL, 2.36 mmol, 10 mol%). The mixture was allowed to stir at room temperature until a clear solution was obtained before it was cooled to 0 °C and allylboronic acid pinacol ester (5.30 mL, 28.3 mmol, 1.20 equiv.) was added dropwise over 5 minutes (white precipitate formed). The reaction was then warmed up to room temperature and stirred for 30 minutes before benzaldehyde (2.39 mL, 23.6 mmol, 1.00 equiv.)[A] was added dropwise over 2 minutes. The reaction was then stirred at room temperature for 18 hours before transferring to a separating funnel containing brine (50 mL) and water (50 mL). The solution was extracted with EtOAc (4 × 100 mL) and the organic phases were combined, dried with Na_2SO_4, filtered, and concentrated under reduced pressure to afford a crude oil.

The crude material was dissolved in a solution of acetone and 0.1 M NaOH (1:1, 90 mL, 0.26 M) and to this was added methylboronic acid (7.05 g, 118 mmol, 5.00 equiv.) and the reaction was allowed to stir at room temperature for 18 hours.[B] Following this, the reaction was transferred to a separating funnel containing brine (100 mL) and extracted with EtOAc (4 × 100 mL). The organic phases were combined, dried over anhydrous Na_2SO_4, filtered, and concentrated under reduced pressure to give a crude residue. To this was added toluene (30 mL) and the solution was concentrated under reduced pressure to facilitate azeotropic removal of excess methylboronic acid and methylboronic acid pinacol ester. This azeotropic process was repeated twice more (2 × 30 mL) to afford a crude oil,[C] which was purified by column chromatography on silica gel (0–5% MeOH in CH_2Cl_2)[D,E] to afford primary amine (*rac*)-**13** (2.41 g, 16.4 mmol, 69%) as a light-orange oil.

Notes: (**A**) Benzaldehyde was freshly distilled prior to use. (**B**) In analogy to a procedure described by Klein and co-workers[44] methylboronic acid was used to perform a

transesterification with the pinacol formed during the reaction, which co-elutes with the product during column chromatography. (**C**) The use of a toluene azeotrope is critical to remove the excess methylboronic acid and methylboronic acid pinacol ester produced during the transesterification process. Otherwise, co-elution of impurities during column chromatography can be an issue. (**D**) Flash column chromatography often has to be repeated 2–3× to fully separate the product from impurities and unfortunately, the use of Et_3N to prevent streaking during column chromatography caused further co-elution. (**E**) Following column chromatography, and prior to performing any further reactions, primary amine (*rac*)-**13** was co-evaporated with toluene 3× and then placed under high-vacuum (0.1 mbar) to remove any residual water.

$\boldsymbol{R}_\mathbf{f}$ = 0.16 (5% MeOH in CH_2Cl_2), stained with $KMnO_4$;

^{1}H NMR (500 MHz, $CDCl_3$) δ = 7.37–7.30 (m, 4H), 7.29–7.21 (m, 1H), 5.76 (dddd, *J* = 16.9, 10.2, 8.0, 6.3 Hz, 1H), 5.12 (dq, *J* = 16.9, 1.7 Hz, 1H), 5.08 (ddt, *J* = 10.2, 1.7, 1.1 Hz, 1H), 4.00 (dd, *J* = 8.0, 5.3 Hz, 1H), 2.47 (app. dddt, *J* = 14.1, 6.3, 5.3, 1.7 Hz, 1H), 2.36 (app. dtt, *J* = 14.1, 8.0, 1.1 Hz, 1H), 1.55 (s, 2H);

^{13}C NMR (126 MHz, $CDCl_3$) δ = 146.0, 135.6, 128.5, 127.1, 126.4, 117.7, 55.5, 44.3;

HRMS (ESI$^+$): Calculated for $C_{10}H_{14}N$ $[M+H]^+$: 148.1121, found: 148.1122;

Analytical data are consistent with those reported previously in the literature.[45]

α-Chloroamide ((*rac*)-14)

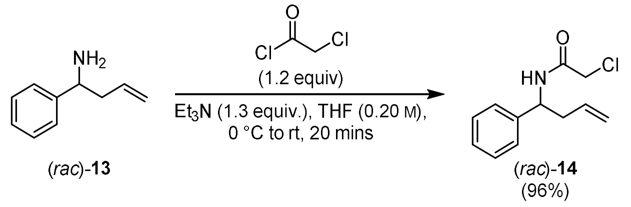

To an oven-dried two-neck round-bottom flask was added amine (*rac*)-**13** (250 mg, 1.70 mmol, 1.00 equiv.) and anhydrous THF (8.50 mL, 0.20 M). The solution was cooled to 0 °C and triethylamine (Et_3N, 0.31 mL, 2.21 mmol, 1.30 equiv.) was added followed by chloroacetyl chloride (0.16 mL, 2.04 mmol, 1.20 equiv.) dropwise over 1 minute (white precipitate formed).[A] The reaction was allowed to stir at 0 °C for 5 minutes before warming to room temperature and stirring for a further 15 minutes. The reaction was then transferred to a separating funnel containing saturated aqueous $NaHCO_3$ (25 mL) and the transferring flask was washed with EtOAc (25 mL). The aqueous phase was extracted with EtOAc (3 × 25 mL) and the organic phases were combined, washed with brine (50 mL), dried over anhydrous Na_2SO_4, filtered, and concentrated under reduced pressure to give a crude oil. The crude product was purified by column chromatography on silica gel (0–20% acetone in *n*-hexane) to afford α-chloroamide (*rac*)-**14** (365 mg, 1.63 mmol, 96%) as a red oil, which slowly crystallises to a light pink solid.[B]

Notes: (**A**) The white precipitate of $Et_3N{\cdot}HCl$ forms instantly and the reaction turns pink after 5 minutes and then to a deep purple colour after 15 minutes. (**B**) α-Chloroamide (*rac*)-**14** can be recrystallized from a solution of TBME/*n*-hexane to afford the product as colourless needles.

$\boldsymbol{R_f}$ = 0.28 (20% acetone in *n*-hexane), stained with $KMnO_4$;

MP: 59–60 °C (TBME/*n*-hexane);

^{1}H NMR (500 MHz, $CDCl_3$) δ = 7.39–7.31 (m, 2H), 7.31–7.24 (m, 3H), 6.97 (br. d, J = 7.8 Hz, 1H), 5.69 (ddt, J = 17.2, 10.2, 7.0 Hz, 1H), 5.19–5.04 (m, 3H), 4.08–3.97 (m, 2H), 2.60 (tt, J = 7.0, 1.2 Hz, 2H);

^{13}C NMR (126 MHz, $CDCl_3$) δ = 165.2, 140.9, 133.5, 128.8, 127.6, 126.4, 118.8, 52.9, 42.8, 40.5;

HRMS (ESI$^+$): Calculated for $C_{12}H_{14}ClNNaO$ $[M+Na]^+$: 246.0656, found: 246.0658;

IR (thin film, cm^{-1}): 3285, 3075, 3007, 2979, 2940, 2917, 1655, 1604, 1586, 1530, 1494, 1454, 1437, 1414, 1328, 1259, 1239, 1149, 1088, 992, 919 and 759;

Racemic-homo/heterodimer ((*rac*)-homo/hetero-15)

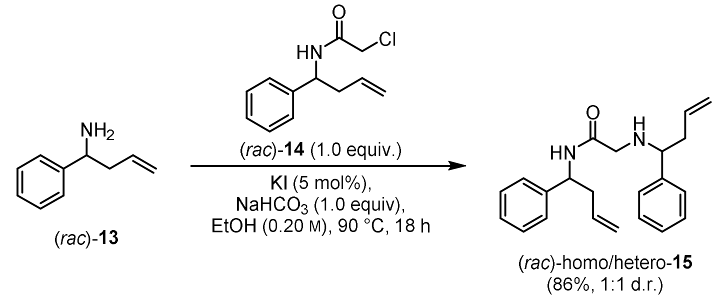

To an oven-dried pressure tube was charged primary amine (*rac*)-**13** (36.8 mg, 0.25 mmol, 1.00 equiv.) and anhydrous EtOH (1.25 mL, 0.20 M). To this solution was added α-chloroamide (*rac*)-**14** (55.9 mg, 0.25 mmol, 1.00 equiv.),[A] KI (2.10 mg, 12.5 μmol, 5 mol%), and $NaHCO_3$ (21.0 mg, 0.25 mmol, 1.00 equiv.)[B]. The reaction was then placed into a pre-heated oil bath at 90 °C and stirred at this temperature for 18 hours. Following this, the reaction was cooled to room temperature and transferred to a separating funnel containing brine (10 mL) and the aqueous phase was extracted with EtOAc (4 × 10 mL). The organic phases were combined, dried over anhydrous Na_2SO_4, filtered, and concentrated under reduced pressure to give a crude oil, which was purified by column chromatography on silica gel (0–30% acetone in *n*-hexane) to afford α-aminoamide (*rac*)-homo/hetero-**15** (72 mg, 0.22 mmol, 86%)[C] as a colourless oil.

Notes: (**A**) Recrystallised α-chloroamide (*rac*)-**14** (using the conditions stated above, page 26) was used; however, the non-recrystallised material can also be used with comparable results. (**B**) $NaHCO_3$ was stored in an oven at 120 °C for at least 24 hours prior to use. (**C**) Pure samples of each diastereomer (*syn* [(*rac*)-homo-**15**] and *anti* [(*rac)*-hetero-**15**]) for individual characterisation can be obtained by column chromatography on silica gel (0–30% acetone in *n*-hexane).

Racemic-homo/heterodimer ((*rac*)-homo/hetero-15)

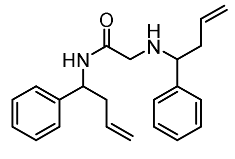

(*rac*)-homo/hetero-**15**
(1:1 d.r.)

$\boldsymbol{R}_f$ = 0.44 (30% acetone in *n*-hexane);

^{1}H NMR (601 MHz, $CDCl_3$) δ = 7.60 (d, *J* = 8.7 Hz, 0.5 × 1H, Heterodimer), 7.54 (d, *J* = 8.7 Hz, 0.5 × 1H, Homodimer), 7.37–7.19 (m, 10H), 5.81–5.61 (m, 2H), 5.17–5.03 (m, 5H), 3.65–3.59 (m, 1H), 3.22–3.10 (m, 2H), 2.63–2.55 (m, 1H), 2.55–2.51 (m, 1H), 2.49–2.36 (m, 2H), 1.93 (br. s, 1H);

*Note: The peaks at 7.60 ppm and 7.54 ppm are attributed to the (rac)-hetero-**15** (anti; [R,S and S,R]) and (rac)-homo-**15** (syn; [R,R and S,S]), respectively. They have been assigned as 0.5 × 1H to reflect the 1:1 diastereomeric ratio observed in the ^{1}H NMR.*

^{13}C NMR (151 MHz, $CDCl_3$) δ = 170.9,* 142.9, 142.8, 141.91, 141.85, 135.13, 135.09, 134.3, 134.1, 128.70,* 128.67, 128.6, 127.6,* 127.4, 127.3, 127.1,* 126.6, 126.4, 118.2, 118.13, 118.09, 118.0, 62.7, 62.6, 52.1, 52.0, 50.4, 50.3, 42.64, 42.61, 41.0, 40.8;

Note: Due to overlapping peaks in the ^{13}C NMR spectrum, four peaks are missing/not observed. Peaks that have overlapped a second peak have been marked with an asterisk (*).

HRMS (ESI$^+$): Calculated for $C_{22}H_{27}N_2O$ $[M+H]^+$: 335.2118, found: 335.2124;

IR (thin film, cm^{-1}): 3312, 3074, 3064, 3029, 2926, 2907, 1657, 1602, 1512, 1495, 1453, 1418, 1357, 1324, 1306, 1259, 1126, 1028, 995, 916, 759, 699 and 688;

HPLC: Daicel Chiralpak® IA column with guard, 5% *i*-PrOH in *n*-hexane, 0.8 mL/min, 30 °C, 214 nm, t_R = 16.93 min (*R*,*R*-isomer), t_R = 18.56 min (*R*,*S*-isomer), t_R = 22.33 min (*S*,*R*-isomer), t_R = 25.84 min (*S*,*S*-isomer), 50:50 e.r., 1:1 d.r.

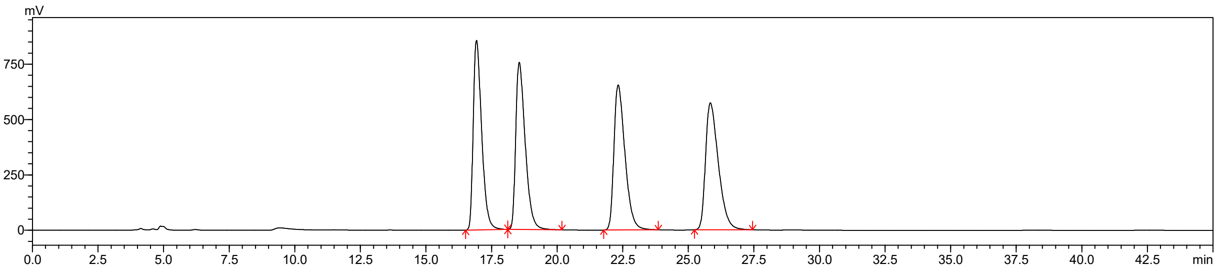

Peak#	Ret. Time	Area	Height	Conc.	Area%
1	16.926	19193145	855987	24.925	24.925
2	18.556	19037438	755260	24.723	24.723
3	22.326	19230253	655269	24.973	24.973
4	25.840	19543599	573374	25.380	25.380
Total		77004435	2839890	100.000	100.000

Racemic-heterodimer ((*rac*)-hetero-15)

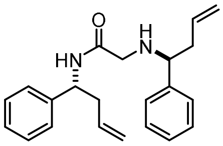

(*rac*)-hetero-**15**
(>95:5 d.r.)

***R*f** = 0.43 (30% acetone in *n*-hexane);

^{1}H NMR (601 MHz, $CDCl_3$) δ = 7.60 (d, *J* = 8.7 Hz, 1H), 7.35–7.28 (m, 4H), 7.28–7.20 (m, 4H), 7.22–7.17 (m, 2H), 5.79–5.68 (m, 2H), 5.17–5.05 (m, 5H), 3.64 (dd, *J* = 7.9, 5.8 Hz, 1H), 3.19–3.11 (m, 2H), 2.62–2.51 (m, 2H), 2.49–2.37 (m, 2H), 1.99 (br. s, 1H);

^{13}C NMR (151 MHz, $CDCl_3$) δ = 170.9, 142.7, 141.9, 135.1, 134.3, 128.8, 128.7, 127.6, 127.3, 127.2, 126.5, 118.21, 118.16, 62.7, 52.0, 50.3, 42.6, 41.1;

HRMS (ESI$^+$): Calculated for $C_{22}H_{27}N_2O$ $[M+H]^+$: 335.2118, found: 335.2123;

IR (thin film, cm^{-1}): 3312, 3075, 3064, 3029, 2978, 2927, 2907, 2838, 1655, 1602, 1585, 1512, 1495, 1453, 1436, 1417, 1357, 1324, 1306, 1275, 1258, 1199, 1126, 1071, 993, 966, 916, 843, 759 and 699;

HPLC: Daicel Chiralpak® IA column with guard, 5% *i*-PrOH in *n*-hexane, 0.8 mL/min, 30 °C, 214 nm, t_R = 18.51 min (*R*,*S*-isomer), t_R = 22.27 min (*S*,*R*-isomer), 50:50 e.r.

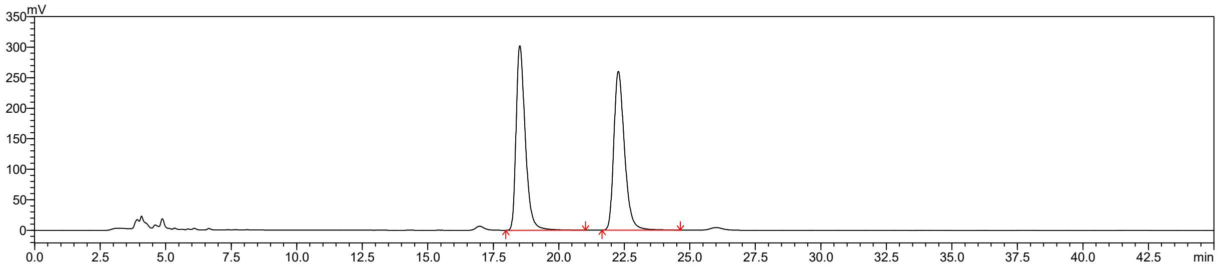

Peak#	Ret. Time	Area	Height	Conc.	Area%
1	18.512	7386710	302153	50.009	50.009
2	22.271	7383981	260030	49.991	49.991
Total		14770691	562184	100.000	100.000

Racemic-homodimer ((*rac*)-homo-15)

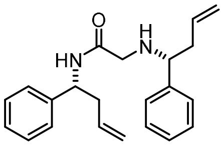

(*rac*)-homo-**15**
(>95:5 d.r.)

R_f = 0.45 (30% acetone in *n*-hexane);

^{1}H NMR (601 MHz, $CDCl_3$) δ = 7.53 (d, *J* = 8.6 Hz, 1H), 7.36–7.31 (m, 4H), 7.28–7.23 (m, 6H), 5.75–5.61 (m, 2H), 5.12–5.02 (m, 5H), 3.61 (dd, *J* = 8.0, 5.7 Hz, 1H), 3.20–3.10 (m, 2H), 2.52 (tt, *J* = 6.9, 1.3 Hz, 2H), 2.48–2.36 (m, 2H);

^{13}C NMR (151 MHz, $CDCl_3$) δ = 170.9, 142.8, 141.9, 135.1, 134.2, 128.8, 128.7, 127.7, 127.4, 127.2, 126.6, 118.2, 118.1, 62.7, 52.2, 50.4, 42.6, 40.9;

HRMS (ESI$^+$): Calculated for $C_{22}H_{27}N_2O$ $[M+H]^+$: 335.2118, found: 335.2119;

IR (thin film, cm^{-1}): 3316, 3064, 3092, 3005, 2979, 2907, 1657, 1620, 1585, 1515, 1495, 1454, 1436, 1417, 1357, 1327, 1308, 1275, 1258, 1200, 1071, 1028, 995, 916, 759 and 701;

HPLC: Daicel Chiralpak® IA column with guard, 5% *i*-PrOH in *n*-hexane, 0.8 mL/min, 30 °C, 214 nm, t_R = 16.92 min (*R*,*R*-isomer), t_R = 25.97 min (*S*,*S*-isomer), 50:50 e.r.

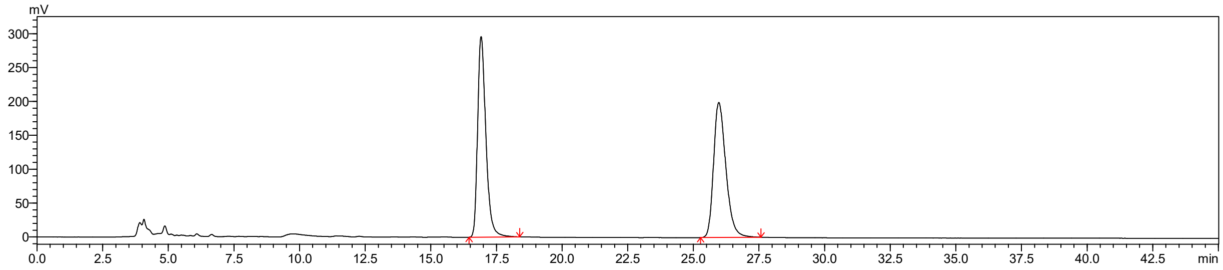

Peak#	Ret. Time	Area	Height	Conc.	Area%
1	16.915	6495287	295925	49.995	49.995
2	25.970	6496466	199366	50.005	50.005
Total		12991753	495291	100.000	100.000

3.2. Synthesis of (*R*,*S*)-Heterodimer

3.2.1. Kinetic Resolution

α-Chloroamide ((*R*)-14) and primary amine ((*S*)-13)

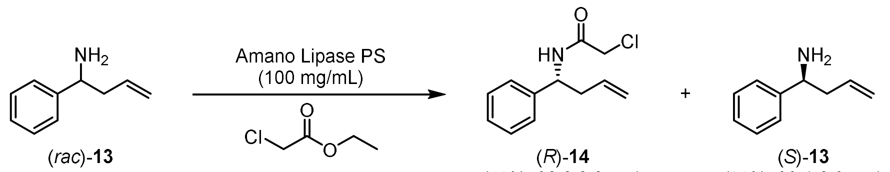

(*rac*)-**13**

(*R*)-**14** (48%, 98.0:2.0 e.r.)

(*S*)-**13** (38%, 99.1:0.9 e.r.)

To an oven-dried 7 mL screw neck sample vial containing a 1.0 cm magnetic stirrer bar under an atmosphere of nitrogen was charged primary amine (*rac*)-**13** (36.8 mg, 0.25 mmol, 1.00 equiv.) and anhydrous toluene (2.50 mL, 0.10 M). To this was added Amano Lipase PS (250 mg),[A] 4 Å molecular sieves (250 mg),[B] and ethyl chloroacetate (0.13 mL, 1.25 mmol, 5.00 equiv.). The sample vial was then sealed with a screw cap and placed into a pre-heated oil bath at 20 °C and stirred for 24 hours.[C] Following this, the reaction was filtered and the transferring flask was washed with EtOAc (25 mL in total). The filtrate was concentrated under reduced pressure and toluene (10 mL) was added. The solution was concentrated under reduced pressure again and the toluene azeotrope was repeated twice more (2 × 30 mL) to afford a colourless oil, which was placed under high vacuum (0.1 mbar) for 30 minutes.[D] The crude residue was then dissolved in Et_2O (1.25 mL, 0.20 M)[E,F] and cooled to 0 °C. To this was added 2 M HCl in Et_2O (0.07 mL, 0.14 mmol, 0.55 equiv.) slowly dropwise and the reaction was warmed to room temperature for 5 minutes. The white precipitate formed (the HCl salt of primary amine (*S*)-**13**)[G] was filtered, washed with Et_2O (5 mL) and the collected filtrate was concentrated under reduced pressure to afford α-chloroamide (*R*)-**14**[H] as a light-yellow oil, which slowly crystallised to a white solid. This was further purified by column chromatography on silica gel (0–20% acetone in *n*-hexane) to afford α-chloroamide (*R*)-**14** (26.8 mg, 48%, 98.0:2.0 e.r.) as a colourless oil, which slowly crystallised to a white solid. Meanwhile, the filtered white precipitate was dissolved in H_2O (2.50 mL) and to this was added saturated aqueous $NaHCO_3$ (2.50 mL). The aqueous phase was then extracted with EtOAc (6 × 5.0 mL) and the organic phases were collected, dried with Na_2SO_4, filtered, and concentrated under reduced pressure to afford primary amine (*S*)-**13**[I] (14.0 mg, 38%, 99.1:0.9 e.r.) as a colourless oil.

Notes: (**A**) Amano Lipase PS (immobilised on diatomite) was purchased from Sigma–Aldrich (product number: 708011). (**B**) 4 Å molecular sieves (powder, activated; CAS: 70955-01-0) were purchased from Alfa Aesar (product number: A11535) and stored in an oven at 120 °C for at least 24 hours prior to use. (**C**) To ensure reproducibility, other temperatures between 20–25 °C were screened, the results of which only gave very slight variation in enantiomeric excess (see **Table 1**). (**D**) A toluene azeotrope (rotary evaporator water bath temperature: 30 °C) followed by high-vaccum (0.1 mbar) is necessary to fully remove the remaining ethyl chloroacetate (BP: 143 °C). (**E**) At this stage an aliquot (~0.01 mL) can be taken and concentrated under high-vacuum (0.1 mbar) for analysis by Chiral HPLC (sample dissolved in 10% *i*-PrOH in *n*-hexane; Daicel Chiralcel® OD-H, 10% *i*-PrOH in *n*-hexane, 0.8 mL/min, 40 °C, 214 nm). (**F**) It has been observed that if the crude residue is left in the freezer overnight, a small amount of the HCl salt of enantioenriched amine (*S*)-**S8** precipitates out when Et_2O is added. If this occurs, then the HCl formation procedure given above is followed as normal. This HCl salt is proposed to form when performing rotary evaporation of the crude reaction mixture by either decomposition of ethyl chloroacetate or α-chloroamide (*R*)-**14** to release HCl that subsequently reacts with primary amine (*S*)-**13**. To mitigate this, we generally keep the water bath temperature on the rotary evaporator at 30 °C. (**G**) At this point, primary amine (*S*)-**13** can also be isolated as its HCl salt (20.0 mg, 44%, data given below). (**H**) At this stage, α-chloroamide (*R*)-**14** is generally clean enough to be carried through to further chemistry (see spectrum below, page 37); however, as stated above, if any minor impurities persist following work-up, column chromatography on silica gel (0–20% acetone in *n*-hexane) can be utilised to remove them. (**I**) Primary amine (*S*)-**13** is also clean enough after work-up to be used in further chemistry (see spectrum below, page 37). If any minor impurities remain, they can be removed by column chromatography on silica gel (0–5% MeOH in CH_2Cl_2); however, this typically results in some product loss on the column.

α-Chloroamide ((*R*)-14)

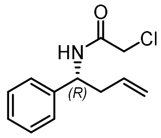

(*R*)-**14**
(98.0:2.0 e.r.)

$[\boldsymbol{\alpha}]_{\mathbf{D}}^{\mathbf{17}}$**:** +64.0 (*c* = 1.00, $CHCl_3$);

HPLC: Daicel Chiralcel® OD-H column with guard, 10% *i*-PrOH in *n*-hexane, 0.8 mL/min, 40 °C, 214 nm, t_R = 10.35 min (*R*-isomer), t_R = 11.15 min (*S*-isomer), 98.0:2.0 e.r.

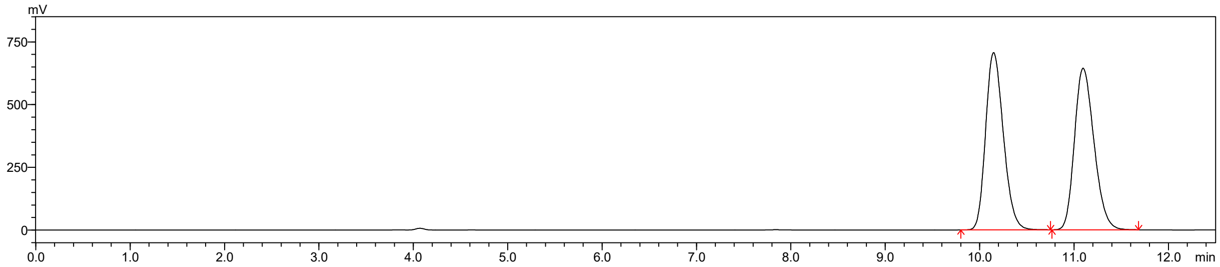

Peak#	Ret. Time	Area	Height	Conc.	Area%
1	10.148	9240641	706815	49.878	49.878
2	11.096	9285830	643757	50.122	50.122
Total		18526471	1350572	100.000	100.000

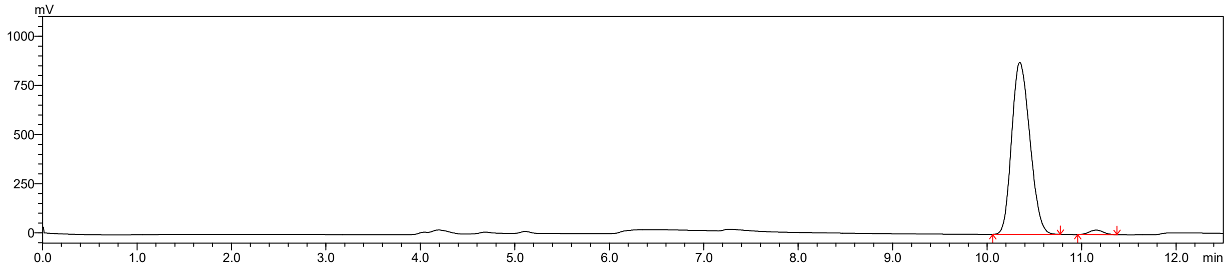

Peak#	Ret. Time	Area	Height	Conc.	Area%
1	10.346	11726017	873984	98.003	98.003
2	11.154	238985	23310	1.997	1.997
Total		11965002	897294	100.000	100.000

Primary amine ((*S*)-13)

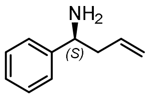

(*S*)-**13**
(99.1:0.9 e.r.)

$[\boldsymbol{\alpha}]_{\mathbf{D}}^{\mathbf{21}}$**:** −40.0 (*c* = 0.50, $CHCl_3$);

Optical rotation is consistent with that reported in the literature.[46]

HPLC: Daicel Chiralcel® OD-H column with guard, 10% *i*-PrOH in *n*-hexane, 0.8 mL/min, 40 °C, 214 nm, t_R = 6.77 min (*R*-isomer), t_R = 7.77 min (*S*-isomer), 99.1:0.9 e.r.

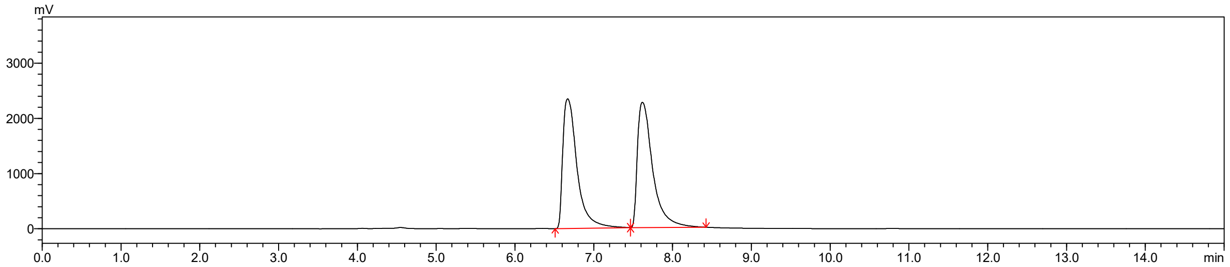

Peak#	Ret. Time	Area	Height	Conc.	Area%
1	6.669	29505665	2349245	49.112	49.112
2	7.617	30572651	2268977	50.888	50.888
Total		60078317	4618223	100.000	100.000

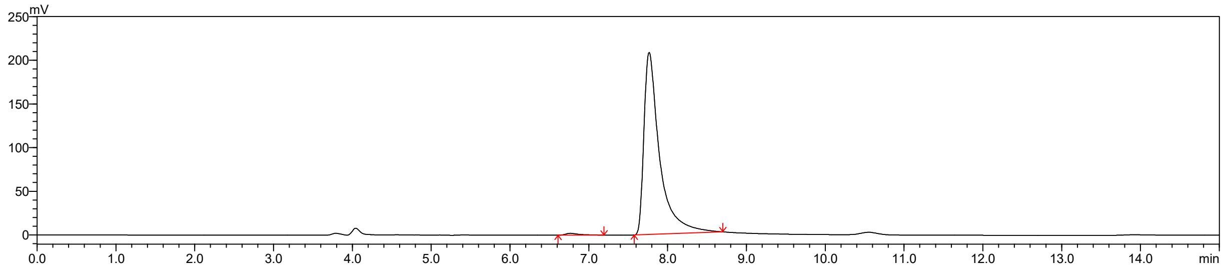

Peak#	Ret. Time	Area	Height	Conc.	Area%
1	6.766	25987	2108	0.892	0.892
2	7.765	2886547	208661	99.108	99.108
Total		2912534	210769	100.000	100.000

Primary amine·HCl salt ((*S*)-S8)

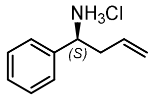

(*S*)-**S8**
(99.1:0.9 e.r.)

^{1}H NMR (601 MHz, $CDCl_3$) δ = 8.78 (br. s, 3H), 7.47 – 7.40 (m, 2H), 7.37–7.29 (m, 3H), 5.55 (ddt, *J* = 17.2, 10.2, 7.0 Hz, 1H), 5.11–5.00 (m, 2H), 4.22 (dd, *J* = 8.5, 6.2 Hz, 1H), 2.86 – 2.78 (m, 1H), 2.74–2.65 (m, 1H);

^{13}C NMR (151 MHz, $CDCl_3$) δ = 135.8, 131.6, 129.1, 129.1, 127.6, 120.3, 56.0, 38.9;

Note: Only one peak in the ^{13}C NMR spectrum at 129.1 ppm is observed with the second peak (listed above) being proposed to be underneath the first peak (in accordance with the literature where these two peaks are very close together; 128.98 and 128.96 ppm).

Analytical data is consistent with that reported in the literature.[47]

^{1}H NMR data of primary amine·HCl salt ((*S*)-S8), primary amine ((*S*)-13) and α-chloroamide ((*R*)-14) obtained from kinetic resolution following work-up:

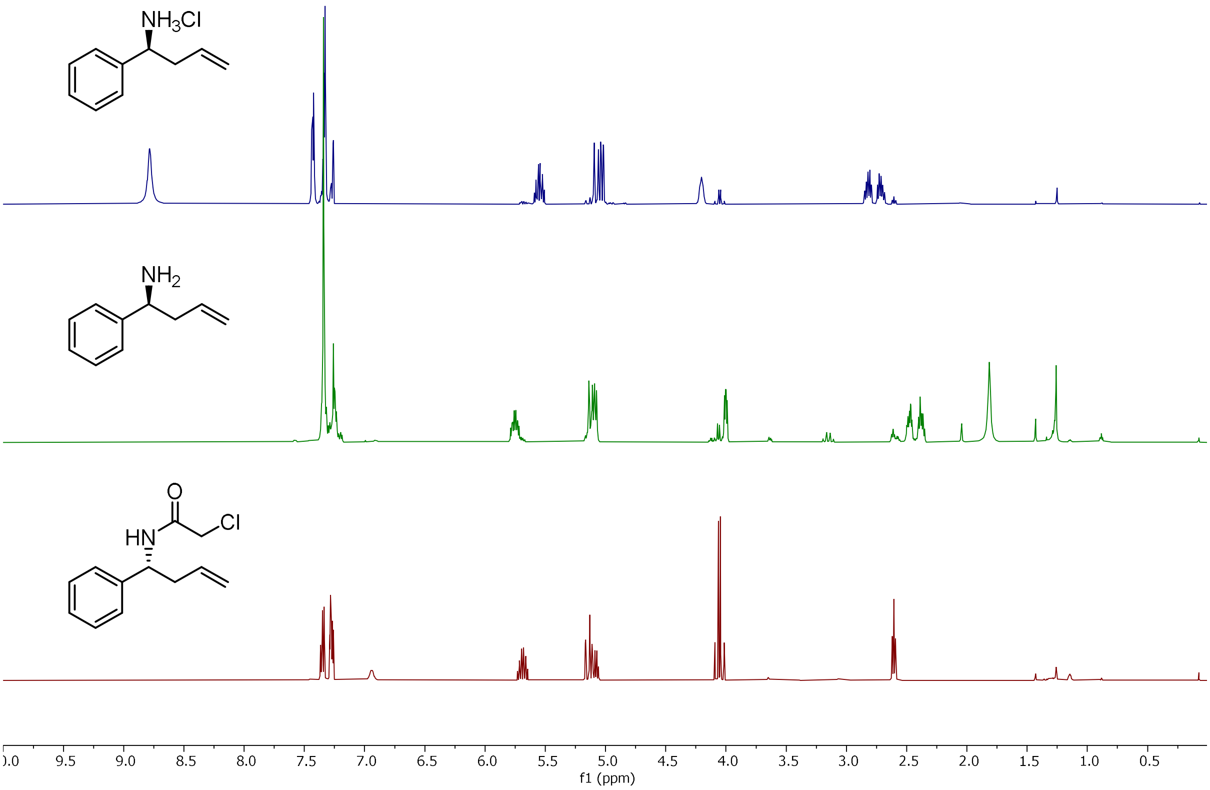

Primary amine·HCl salt ((*rac*)-S8)

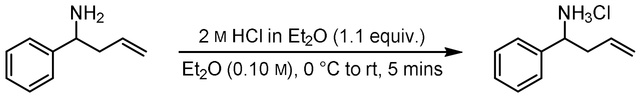

(*rac*)-**13**

(*rac*)-**S8**
(98%)

To an oven-dried 7 mL screw neck sample vial containing amine (*rac*)-**13** (36.8 mg, 0.25 mmol, 1.00 equiv.) and anhydrous Et_2O (2.50 mL, 0.10 M) at 0 °C was added 2 M HCl in Et_2O (0.14 mL, 0.28 mmol, 1.10 equiv.) dropwise over 1 minute (white precipitate formed). The reaction was then warmed to room temperature and allowed to stir for a further 5 minutes before filtering and washing with cold Et_2O (5 mL). The white precipitate was collected and placed under high vacuum (0.1 mbar) for 30 minutes to afford primary amine·HCl salt (*rac*)-**S8** (45 mg, 98%) as a white solid.

^{1}H NMR (601 MHz, $CDCl_3$) δ = 8.78 (br. s, 3H), 7.47–7.40 (m, 2H), 7.37–7.29 (m, 3H), 5.55 (ddt, *J* = 17.2, 10.2, 7.0 Hz, 1H), 5.11–5.00 (m, 2H), 4.22 (dd, *J* = 8.5, 6.2 Hz, 1H), 2.86 – 2.78 (m, 1H), 2.74 – 2.65 (m, 1H);

^{13}C NMR (151 MHz, $CDCl_3$) δ = 135.8, 131.6, 129.1, 129.1, 127.6, 120.3, 56.0, 38.9;

Note: Only one peak in the ^{13}C NMR spectrum at 129.1 ppm is observed with the second peak (listed above) being proposed to be underneath the first peak (in accordance with the literature these two peaks are very close together; 128.98 and 128.96 ppm).

Analytical data are consistent with those reported above and in the literature.[47]

3.2.2. Biocatalytic Stereoretentive Enantioconvergent Coupling

(*R*,*S*)-Heterodimer (15)

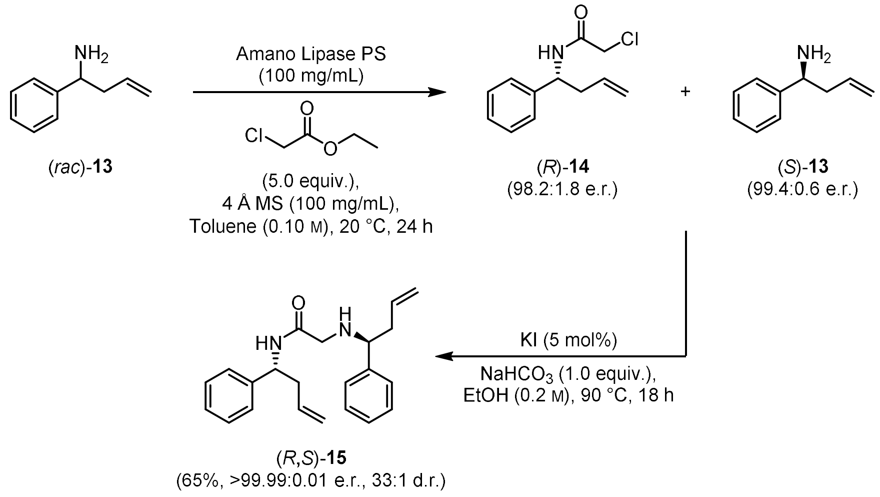

Kinetic Resolution: To an oven-dried 7 mL screw neck sample vial containing a 1.0 cm magnetic stirrer bar under an atmosphere of nitrogen was charged primary amine (*rac*)-**13** (36.8 mg, 0.25 mmol, 1.00 equiv.) and anhydrous toluene (2.50 mL, 0.10 M). To this was added Amano Lipase PS (250 mg),[A] 4 Å molecular sieves (250 mg),[B] and ethyl chloroacetate (0.13 mL, 1.25 mmol, 5.00 equiv.). The sample vial was then sealed with a screw cap and placed into a pre-heated oil bath at 20 °C and stirred for 24 hours.[C] Following this, the reaction was filtered and the transferring flask was washed with EtOAc (25 mL in total). The filtrate was concentrated under reduced pressure and toluene (10 mL) was added. The solution was concentrated under reduced pressure again and the toluene azeotrope was repeated twice more (2 × 30 mL) to afford a colourless oil, which was placed under high vacuum (0.1 mbar) for 30 minutes.[D]

Dimerisation: To this crude material was added anhydrous EtOH (0.63 mL, 0.20 M) and the solution was transferred to a pressure tube under an atmosphere of nitrogen.[E,F] Following this, KI (1.00 mg, 6.25 μmol, 5 mol%) and $NaHCO_3$ (10.5 mg, 0.125 mmol, 1.00 equiv.)[G] were added. The tube was then sealed and placed into a pre-heated oil bath at 90 °C for 18 hours. After this time, the reaction was cooled to room temperature and transferred to a separating funnel containing brine (10 mL). The aqueous phase was extracted with EtOAc (4 × 10 mL) and the organic phases were combined, dried with Na_2SO_4, filtered, and concentrated under reduced pressure to give a crude colourless oil. The crude product was purified by flash column chromatography (0–30% acetone in *n*-hexane) to afford enantioenriched heterodimer (*R*,*S*)-**15** (27 mg, 65%, >99.99:0.01 e.r., 33:1 d.r.) as a colourless oil.[G]

Notes: (**A**) Amano Lipase PS (immobilised on diatomite) was purchased from Sigma–Aldrich (product number: 708011). (**B**) 4 Å molecular sieves (powder, activated; CAS: 70955-01-0) were purchased from Alfa Aesar (product number: A11535) and stored in an oven at 120 °C for at least 24 hours prior to use. (**C**) To ensure reproducibility, other temperatures between 20–25 °C were screened, the results of which only gave very slight variation in enantiomeric excess (see **Table 1**). (**D**) A toluene azeotrope (rotary evaporator water bath temperature: 30 °C) followed by high-vaccum (0.1 mbar) is necessary to fully remove the remaining ethyl chloroacetate (BP: 143 °C). (**E**) On larger scale (1 mmol) a small amount (~5 mg) of primary amine·HCl salt (*S*)-**S8** precipitated out when EtOH was added. This HCl salt (as stated above) is proposed to form when performing rotary evaporation of the crude reaction mixture by either decomposition of ethyl chloroacetate or α-chloroamide (*R*)-**14** to release HCl that subsequently reacts with primary amine (*S*)-**13**. To mitigate this, the temperature of the water bath on the rotary evaporator is kept at 30 °C. However, if precipitation does occur, quickly transferring the solution and precipitate into the pressure tube and continuing as normal gives only a very slight reduction in yield (60% *vs.* 65%; see **Note H**). (**F**) At this stage an aliquot (~0.01 mL) can be taken and concentrated under high-vacuum (0.1 mbar) for analysis by Chiral HPLC (sample dissolved in 10% *i*-PrOH in *n*-hexane; Daicel Chiralcel® OD-H, 10% *i*-PrOH in *n*-hexane, 0.8 mL/min, 40 °C, 214 nm) - the crude data for the kinetic resolution reaction prior to dimerisation is given below. (**G**) $NaHCO_3$ was stored in an oven at 120 °C for at least 24 hours prior to use. (**H**) On 1 mmol scale (147.2 mg of amine **13**) the reaction afforded enantioenriched heterodimer (*R*,*S*)-**15** (99.6 mg, 60%, >99.99:0.01 e.r., 33:1 d.r.).

$\boldsymbol{R_f}$ = 0.43 (30% acetone in *n*-hexane);

^{1}H NMR (601 MHz, $CDCl_3$) δ = 7.60 (d, *J* = 8.7 Hz, 1H), 7.35–7.28 (m, 4H), 7.28–7.20 (m, 4H), 7.22–7.17 (m, 2H), 5.79–5.68 (m, 2H), 5.17–5.05 (m, 5H), 3.64 (dd, *J* = 7.9, 5.8 Hz, 1H), 3.19–3.11 (m, 2H), 2.62–2.51 (m, 2H), 2.49–2.37 (m, 2H), 1.99 (br. s, 1H);

^{13}C NMR (151 MHz, $CDCl_3$) δ = 170.9, 142.7, 141.9, 135.1, 134.3, 128.8, 128.7, 127.6, 127.3, 127.2, 126.5, 118.21, 118.16, 62.7, 52.0, 50.3, 42.6, 41.1;

HRMS (ESI$^+$): Calculated for $C_{22}H_{27}N_2O$ $[M+H]^+$: 335.2118, found: 335.2123;

IR (thin film, cm^{-1}): 3312, 3075, 3064, 3029, 2978, 2927, 2907, 2838, 1655, 1602, 1585, 1512, 1495, 1453, 1436, 1417, 1357, 1324, 1306, 1275, 1258, 1199, 1126, 1071, 993, 966, 916, 843, 759 and 699;

$[\alpha]_D^{24}$: +16.8 (c = 1.00, $CHCl_3$);

HPLC: Daicel Chiralpak® IA column with guard, 5% *i*-PrOH in *n*-hexane, 0.8 mL/min, 30 °C, 214 nm, t_R = 16.89 min (*R*,*R*-isomer [minor homodimer]), t_R = 18.30 min (*R*,*S*-isomer [major heterodimer]), t_R = 22.28 min (*S*,*R*-isomer [minor heterodimer]), t_R = 25.81 min (*S*,*S*-isomer [major homodimer]), 99.995:0.005 e.r., 33:1 d.r.

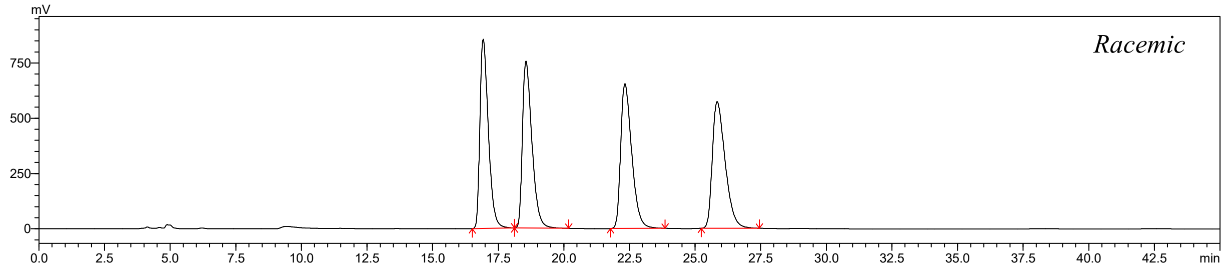

Peak#	Ret. Time	Area	Height	Conc.	Area%
1	16.926	19193145	855987	24.925	24.925
2	18.556	19037438	755260	24.723	24.723
3	22.326	19230253	655269	24.973	24.973
4	25.840	19543599	573374	25.380	25.380
Total		77004435	2839890	100.000	100.000

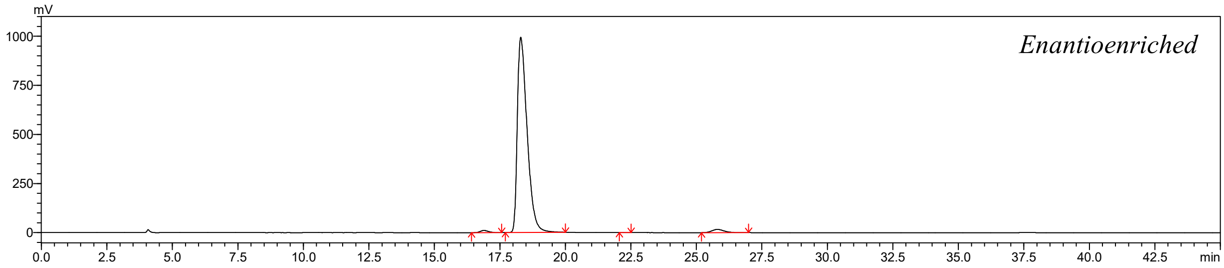

Peak#	Ret. Time	Area	Height	Conc.	Area%
1	16.891	241856	11113	0.909	0.909
2	18.296	25827179	994385	97.061	97.061
3	22.280	1415	88	0.005	0.005
4	25.806	538678	16488	2.024	2.024
Total		26609128	1022073	100.000	100.000

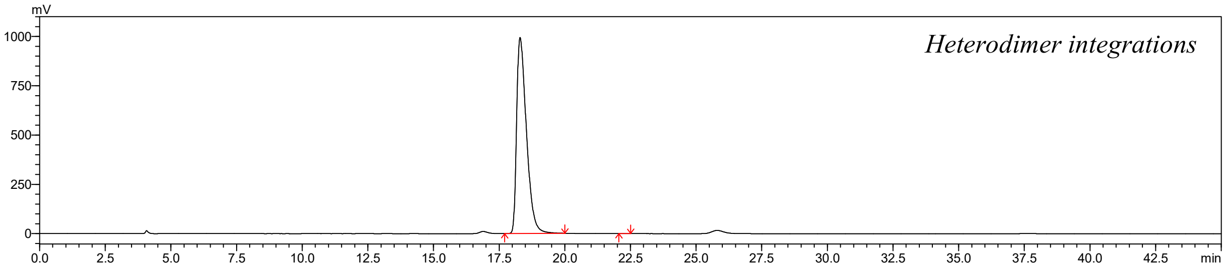

Peak#	Ret. Time	Area	Height	Conc.	Area%
1	18.296	25827179	994385	99.995	99.995
2	22.280	1415	88	0.005	0.005
Total		25828594	994472	100.000	100.000

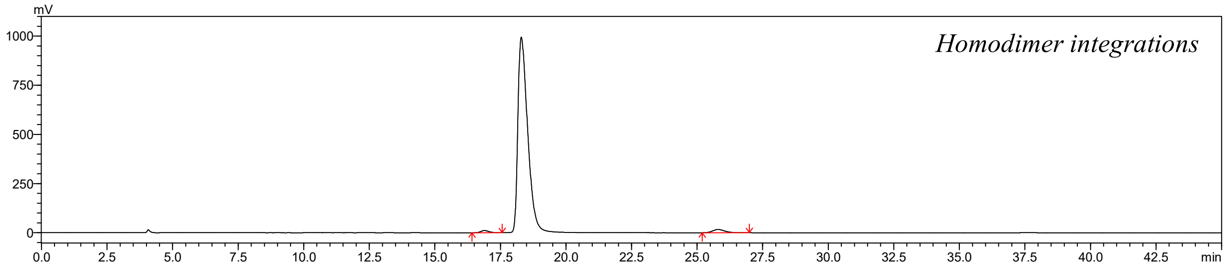

Peak#	Ret. Time	Area	Height	Conc.	Area%
1	16.891	241856	11113	30.986	30.986
2	25.806	538678	16488	69.014	69.014
Total		780534	27601	100.000	100.000

HPLC (Aliquot from Kinetic Resolution): Daicel Chiralcel® OD-H column with guard, 10% *i*-PrOH in *n*-hexane, 0.8 mL/min, 40 °C, 214 nm, t_R = 6.73 min (*R*-isomer of primary amine **13**), t_R = 7.73 min (*S*-isomer of primary amine **13**), 99.4:0.6 e.r., t_R = 10.35 min (*R*-isomer of α-chloroamide **14**), t_R = 11.15 min (*S*-isomer of α-chloroamide **14**), 98.2:1.8 e.r.

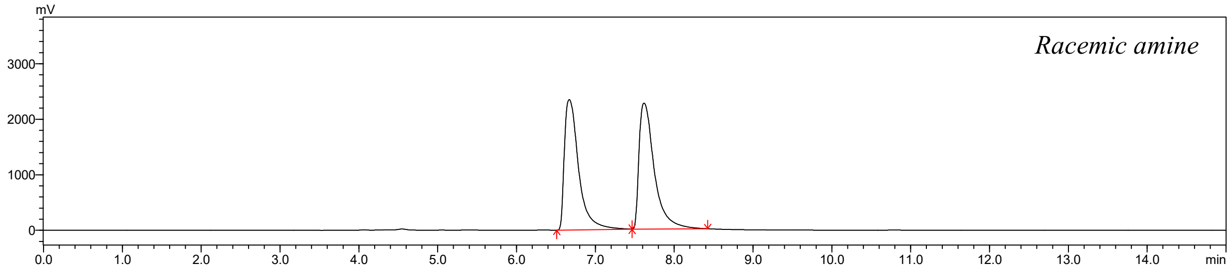

Peak#	Ret. Time	Area	Height	Conc.	Area%
1	6.669	29505665	2349245	49.112	49.112
2	7.617	30572651	2268977	50.888	50.888
Total		60078317	4618223	100.000	100.000

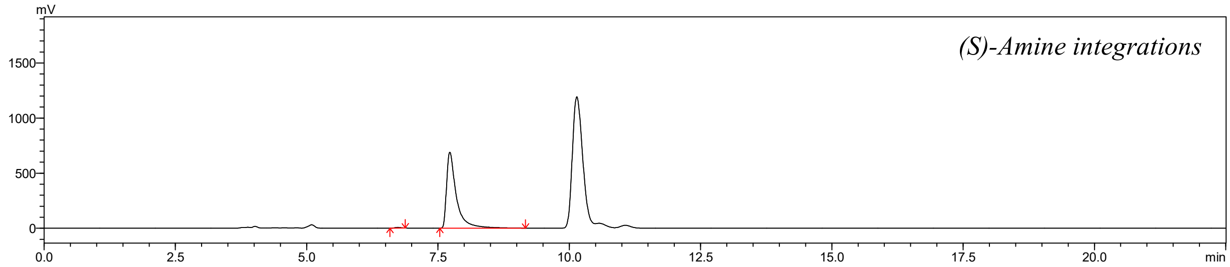

Peak#	Ret. Time	Area	Height	Conc.	Area%
1	6.730	53098	6302	0.589	0.589
2	7.725	8959441	689710	99.411	99.411
Total		9012539	696012	100.000	100.000

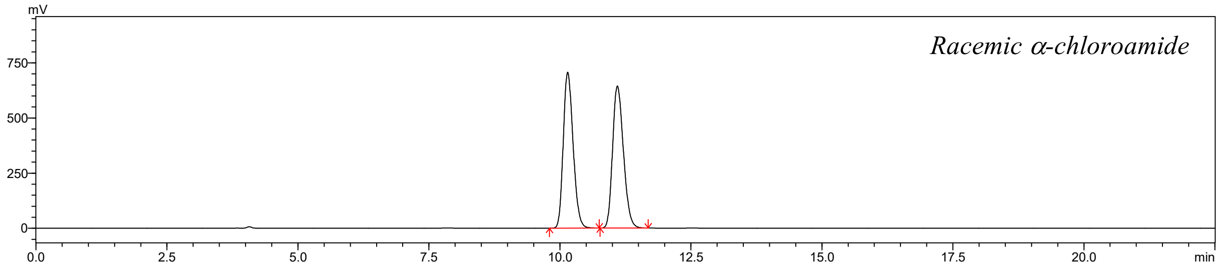

Peak#	Ret. Time	Area	Height	Conc.	Area%
1	10.148	9240641	706815	49.878	49.878
2	11.096	9285830	643757	50.122	50.122
Total		18526471	1350572	100.000	100.000

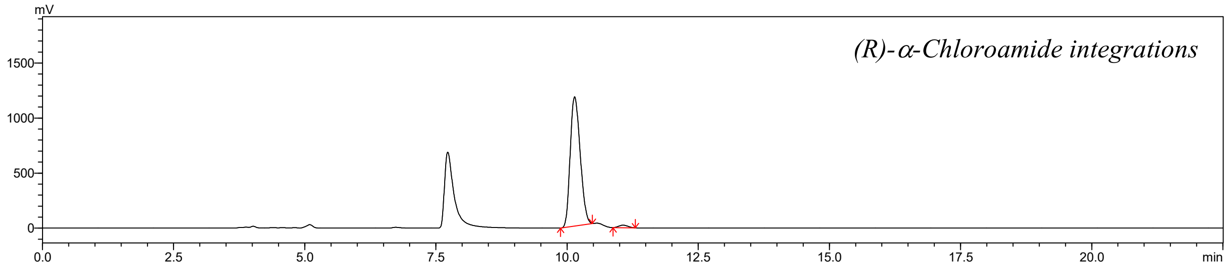

Peak#	Ret. Time	Area	Height	Conc.	Area%
1	10.143	15549944	1174255	98.215	98.215
2	11.067	282604	23062	1.785	1.785
Total		15832548	1197317	100.000	100.000

3.2.3. Dimer HPLC Trace Assignment

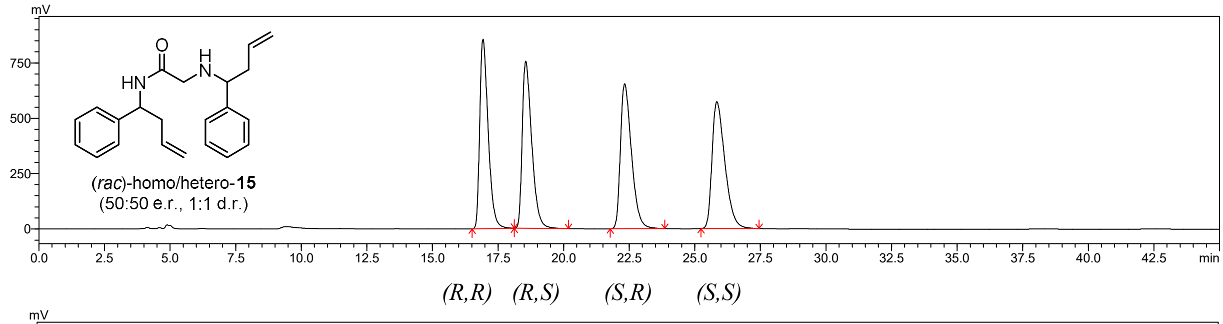

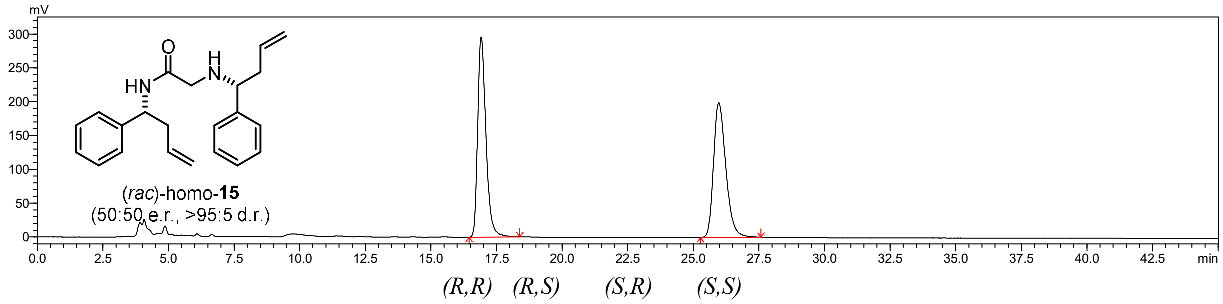

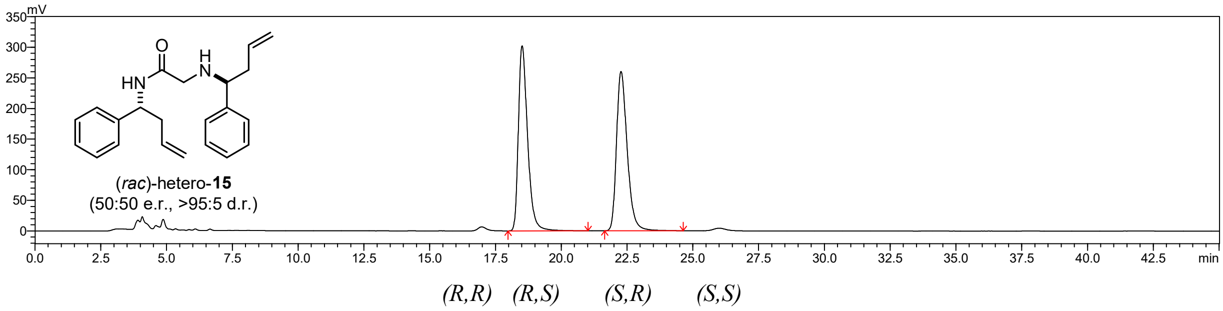

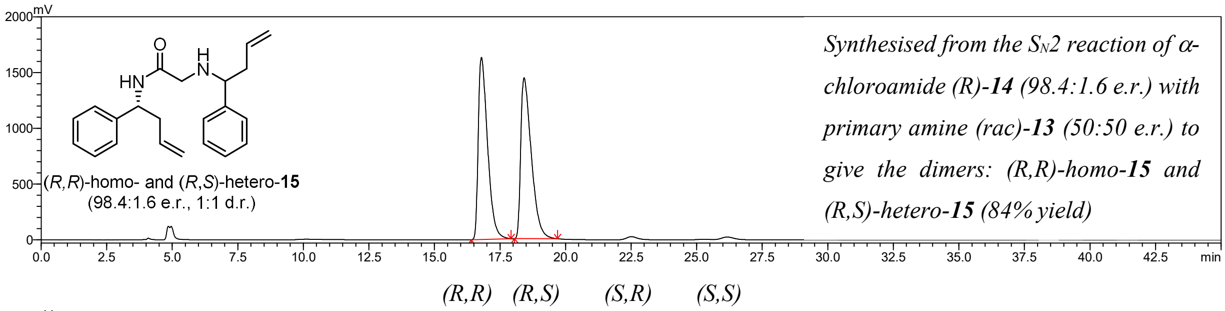

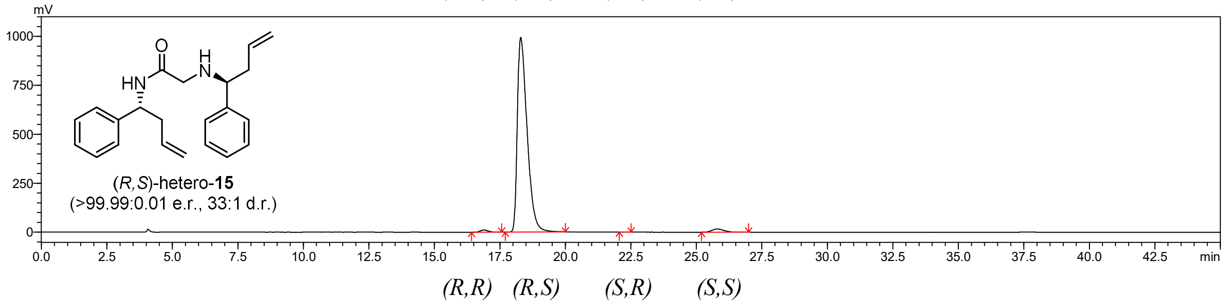

3.3. Optimisation of the Biocatalytic Kinetic Resolution and S_N2 Dimerisation

3.3.1. Biocatalytic Kinetic Resolution

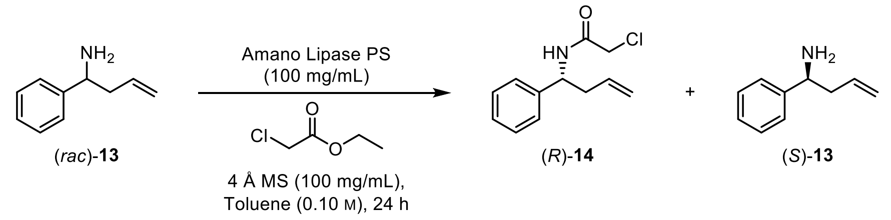

Table 1. Optimisation of the kinetic resolution (0.25 mmol scale; 36.8 mg of (*rac*)-**13**).

**Entry**	**Ethyl chloroacetate**	**Temp.**	**(*S*)-13**[A]	**(*R*)-14**[A]	**Conv.**[B]
Ethyl chloroacetate equivalents					
**1**[C]	2.00 equiv.	21 °C	75:25	99.95:0.05	33%
**2**[C]	5.00 equiv.	21 °C	85:15	99.7:0.3	41%
**3**[C]	10.0 equiv.	21 °C	80:20	99.6:0.4	38%
**4**[D]	5.00 equiv.	21 °C	98.8:1.2	98.4:1.6	50%
**5**[D]	10.0 equiv.	21 °C	96.0:4.0	98.0:2.0	49%
Temperature					
**6**[D]	5.00 equiv.	23 °C	98.8:1.2	98.5:1.5	50%
**7**[D]	5.00 equiv.	22 °C	97.8:2.2	98.6:1.4	50%
**8**[D]	5.00 equiv.	21 °C	98.0:2.0	98.6:1.4	50%
**9**[D]	5.00 equiv.	20 °C	99.1:0.09 (38%)[E]	98.0:2.0 (48%)[E]	51%

A: Enantiomeric ratio determined by chiral HPLC (Daicel Chiralcel® OD-H column with guard, 10% *i*-PrOH in *n*-hexane, 0.8 mL/min, 40 °C, 214 nm). **B:** The conversion was calculated from the obtained enantiomeric ratios using the formula: $Conv. = \frac{\text{\% e.e.of recovered starting material}}{(\text{\% e.e.of recovered starting material + \% e.e.of product})} \times 100$ **C:** 4 Å molecular sieves (beads, 4–8 mesh; CAS: 70955-01-0) were used. **D:** 4 Å molecular sieves (powder, activated; CAS: 70955-01-0) were used. **E:** Isolated yields.

3.3.2. S_N2 Dimerisation

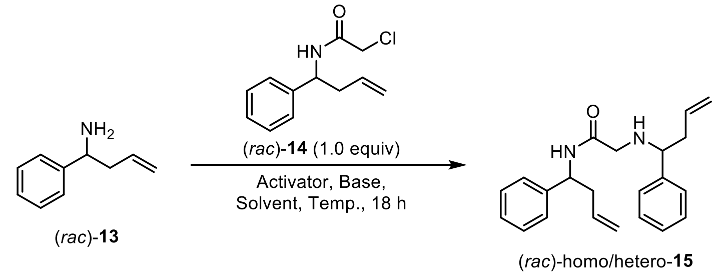

Table 2. Optimisation of the S_N2 Reaction (0.25 mmol scale; 36.8 mg of (*rac*)-**13**).

Entry	Activator	Base	Solvent	Temp.	Yield[A]	Conv.[B]
Activator (KI) loading						
**1**	-	K_2CO_3 (1 equiv.)	EtOH (0.1 M)	90 °C	32%	54%
**2**	KI (2 mol%)	K_2CO_3 (1 equiv.)	EtOH (0.1 M)	90 °C	43%	65%
**3**	KI (5 mol%)	K_2CO_3 (1 equiv.)	EtOH (0.1 M)	90 °C	44%	65%
**4**	KI (10 mol%)	K_2CO_3 (1 equiv.)	EtOH (0.1 M)	90 °C	41%	68%
**5**	KI (20 mol%)	K_2CO_3 (1 equiv.)	EtOH (0.1 M)	90 °C	0%	77%
**6**	KI (50 mol%)	K_2CO_3 (1 equiv.)	EtOH (0.1 M)	90 °C	0%	90%
Solvent						
**7**	KI (5 mol%)	K_2CO_3 (1 equiv.)	Acetone (0.1 M)	90 °C	18%	24%
**8**	KI (5 mol%)	K_2CO_3 (1 equiv.)	THF (0.1 M)	90 °C	18%	30%
**9**	KI (5 mol%)	K_2CO_3 (1 equiv.)	MeCN (0.1 M)	90 °C	0%	53%
**10**	KI (5 mol%)	K_2CO_3 (1 equiv.)	Dioxane (0.1 M)	90 °C	36%	47%
Iodide source						
**11**	NaI (5 mol%)	$NaHCO_3$ (1 equiv.)	EtOH (0.1 M)	90 °C	70%	83%
**12**	TBAI (5 mol%)	$NaHCO_3$ (1 equiv.)	EtOH (0.1 M)	90 °C	60%	74%
Base						
**13**	KI (5 mol%)	$NaHCO_3$ (1 equiv.)	EtOH (0.1 M)	90 °C	72%	86%

**14**	KI (5 mol%)	$KHCO_3$ (1 equiv.)	EtOH (0.1 M)	90 °C	62%	82%
**15**	KI (5 mol%)	K_2CO_3 (1 equiv.)	EtOH (0.1 M)	90 °C	44%	65%
**16**	KI (5 mol%)	Cs_2CO_3 (1 equiv.)	EtOH (0.1 M)	90 °C	20%	-
**17**	KI (5 mol%)	Ag_2CO_3 (1 equiv.)	EtOH (0.1 M)	90 °C	30%	46%
**18**	KI (5 mol%)	K_3PO_4 (1 equiv.)	EtOH (0.1 M)	90 °C	41%	-
**19**	KI (5 mol%)	K_2HPO_4 (1 equiv.)	EtOH (0.1 M)	90 °C	62%	82%
**20**	KI (5 mol%)	DIPEA (1 equiv.)	EtOH (0.1 M)	90 °C	65%	72%
**21**	KI (5 mol%)	Et_3N (1 equiv.)	EtOH (0.1 M)	90 °C	54%	78%
**22**	KI (5 mol%)	DMAP (1 equiv.)	EtOH (0.1 M)	90 °C	18%	26%
Equivalents of base						
**23**	KI (5 mol%)	$NaHCO_3$ (0.5 equiv.)	EtOH (0.1 M)	90 °C	61%	73%
**24**	KI (5 mol%)	$NaHCO_3$ (1.0 equiv.)	EtOH (0.1 M)	90 °C	72%	86%
**25**	KI (5 mol%)	$NaHCO_3$ (1.5 equiv.)	EtOH (0.1 M)	90 °C	71%	87%
**26**	KI (5 mol%)	$NaHCO_3$ (2.0 equiv.)	EtOH (0.1 M)	90 °C	60%	76%
Concentration						
**27**	KI (5 mol%)	$NaHCO_3$ (1 equiv.)	EtOH (0.15 M)	90 °C	80%	87%
**28**	KI (5 mol%)	$NaHCO_3$ (1 equiv.)	EtOH (0.20 M)	90 °C	85%	96%
Temperature						
**29**	KI (5 mol%)	$NaHCO_3$ (1 equiv.)	EtOH (0.2 M)	70 °C	72%	82%
**30**	KI (5 mol%)	$NaHCO_3$ (1 equiv.)	EtOH (0.2 M)	80 °C	56%	60%
Final conditions and isolated yield						
**31**	KI (5 mol%)	$NaHCO_3$ (1 equiv.)	EtOH (0.20 M)	90 °C	86%	-

A: NMR yields determined by ^{1}H NMR analysis of the crude reaction mixture using 1,4-dinitrobenzene as an internal standard (30 s relaxation delay applied). **B:** Conversion (of α-chloroamide) determined by ^{1}H NMR

analysis of the crude reaction mixture using 1,4-dinitrobenzene as an internal standard (30 s relaxation delay applied).

4. Organocatalytic Stereoretentive Enantioconvergent Reaction

4.1. Synthesis of Starting Materials

Synthesis of Bifunctional Organocatalyst

9-amino-(9-deoxy)-*epi*-cinchonidine (S9)

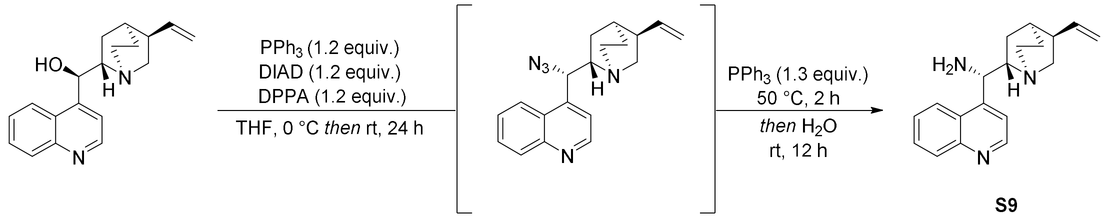

S9
(84%)

Based on conditions reported by Soós and co-workers.[48] Cinchonidine (1.47 g, 5.00 mmol, 1.00 equiv.) and triphenylphosphine (1.57 g, 6.00 mmol, 1.20 equiv.) were dissolved in anhydrous THF (25 mL) in an oven-dried Schlenk flask under N_2. The solution was cooled to 0 °C, before diisopropyl azodicarboxylate (DIAD) (1.18 mL, 6.00 mmol, 1.20 equiv.) was added in one portion. A solution of diphenyl phosphoryl azide (DPPA) (1.29 mL, 6.00 mmol, 1.20 equiv.) in anhydrous THF (10 mL) was then added dropwise at 0 °C, and the resulting mixture was allowed to warm to rt and stir for 24 h. The solution was then heated to 50 °C for 2 h before triphenylphosphine (1.70 g, 6.50 mmol, 1.30 equiv.) was added, and heating was maintained until gas evolution had ceased (2 h). The solution was cooled to rt, H_2O (3 mL) was added, and the reaction mixture was stirred for 12 hours. The solvents were removed under reduced pressure and the residue was dissolved in CH_2Cl_2 and 10% aqueous HCl solution (1:1, 100 mL). The layers were separated, and the aqueous phase was extracted with CH_2Cl_2 (3 × 50 mL). Then, the aqueous phase was made alkaline with pellets of sodium hydroxide to pH = 13, before it was extracted with CH_2Cl_2 (3 × 20 mL). The combined organic extracts were dried over anhydrous Na_2SO_4, filtered, and concentrated under reduced pressure. The residue was purified by column chromatography on silica gel (EtOAc/MeOH/Et_3N 50:50:1) to afford the title compound (1.23 g, 4.19 mmol, 84%) as a colourless viscous oil.

Analytical data were consistent with those reported previously in the literature.[49]

***R*$_f$** = 0.15 (50% MeOH in EtOAc), UV active, stained with $KMnO_4$;

^{1}H NMR (500 MHz, $CDCl_3$): δ = 8.89 (d, *J* = 4.6 Hz, 1H), 8.32 (brs, 1H), 8.12 (dd, *J* = 8.4, 1.4 Hz, 1H), 7.70 (ddd, *J* = 8.3, 6.8, 1.4 Hz, 1H), 7.61–7.52 (m, 2H), 5.77 (ddd, *J* = 17.4, 10.4,

7.3 Hz, 1H), 5.05–4.95 (m, 2H), 4.73 (brs, 1H), 3.37–3.18 (m, 3H), 2.95–2.82 (m, 4H), 2.33 (brs, 1H), 1.68–1.57 (m, 3H), 1.51–1.39 (m, 1H), 0.77 (ddt, J = 13.8, 7.4, 2.0 Hz, 1H);

^{13}C NMR (125 MHz, $CDCl_3$): δ = 150.5, 148.6, 148.3, 141.1, 130.6, 129.3, 127.7, 126.8, 123.2, 119.6, 115.0, 62.0, 55.9, 41.1, 39.4, 27.6, 27.5, 25.9;

HRMS (ESI$^+$): Calc. for $C_{19}H_{24}N_3$ $[M+H]^+$: 294.1965; found: 294.1973; calc. for $C_{19}H_{23}N_3Na$ $[M+Na]^+$: 316.1784; found: 316.1782.

Bifunctional cinchona-thiourea catalyst (18)

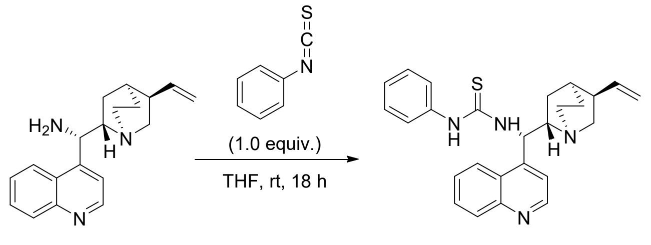

S9

18
(90%)

Based on conditions reported by Niu and co-workers.[50] To a solution of 9-amino-(9-deoxy)-*epi*-cinchonidine **S9** (1.20 g, 4.09 mmol, 1.00 equiv.) in anhydrous THF (12 mL) was added slowly over 1 minute a solution of phenyl isothiocyanate (0.50 mL, 4.09 mmol, 1.00 equiv.) in anhydrous THF (6 mL) under N_2 atmosphere. The reaction mixture was left stirring at rt for 18 h, whereupon the solvent was removed under reduced pressure. The residue was purified by column chromatography on silica gel (10% MeOH in EtOAc) to afford the title compound (1.57 g, 3.66 mmol, 90%) as a colourless solid.

Analytical data were consistent with those reported previously in the literature.[50]

$\boldsymbol{R}_\mathbf{f}$ = 0.44 (17% MeOH in EtOAc), UV active, stained with $KMnO_4$;

MP: 127–129 °C (EtOAc/MeOH);

^{1}H NMR (600 MHz, CD_3OD): δ = 8.82 (d, J = 4.7 Hz, 1H), 8.68 (d, J = 8.6 Hz, 1H), 8.06 (dd, J = 8.6, 1.3 Hz, 1H), 7.79 (s, 1H), 7.70 (s, 1H), 7.59 (d, J = 4.7 Hz, 1H), 7.36–7.29 (m, 4H), 7.18–7.14 (m, 1H), 6.21 (d, J = 10.8 Hz, 1H), 5.76 (ddd, J = 17.1, 10.4, 7.5 Hz, 1H), 4.99 (dt, J = 17.1, 1.5 Hz, 1H), 4.93 (dt, J = 10.4, 1.4 Hz, 1H), 3.42 (d, J = 14.0 Hz, 1H), 3.30–3.20 (m, 2H), 2.86–2.77 (m, 1H), 2.74 (ddd, J = 13.8, 5.1, 2.5 Hz, 1H), 2.37–2.30 (m, 1H), 1.74 (dddd, J = 12.3, 10.2, 5.1, 3.2 Hz, 1H), 1.68–1.59 (m, 2H), 1.29 (ddt, J = 13.3, 10.8, 3.4 Hz, 1H), 0.97 (ddt, J = 13.7, 7.2, 2.0 Hz, 1H);

^{13}C NMR (150 MHz, $CDCl_3$): δ = 180.8, 150.2, 148.6, 141.1, 137.5, 130.5, 129.7, 129.3, 127.4, 126.8, 126.7, 125.2, 124.0, 114.8, 61.5, 55.4, 41.3, 39.5, 27.8, 27.4, 25.8;

HRMS (ESI$^+$): Calc. for $C_{26}H_{29}N_4S_1$ $[M+H]^+$: 429.2107; found: 429.2114.

Synthesis of Racemic Nitroalcohol

(*rac*)-Nitroallylic alcohol (17)

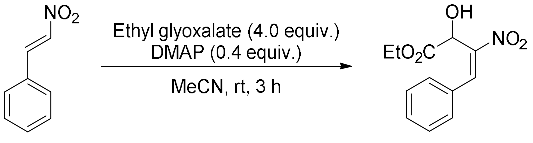

17
(41%)

Based on conditions reported by Namboothiri and co-workers.[51] DMAP (3.30 g, 27.0 mmol, 0.40 equiv.) was added to a stirred solution of *trans*-*β*-nitrostyrene (10.0 g, 67.0 mmol, 1.00 equiv.) in anhydrous MeCN (135 mL), followed by addition of ethyl glyoxalate (50% in toluene, 53.0 mL, 268 mmol, 4.00 equiv). The reaction mixture was stirred at rt until consumption of nitrostyrene (3 h, monitored by TLC). The resulting suspension was filtered under vacuum through a plug of Celite® and rinsed with EtOAc (300 mL). The filtrate was transferred to a separating funnel, H_2O (250 mL) was added and the layers were separated. The aqueous phase was extracted with EtOAc (3 × 200 mL), and the combined organic extracts were dried over anhydrous Na_2SO_4, filtered, and concentrated under reduced pressure. The residue was purified by column chromatography on silica gel (14% EtOAc in *n*-hexane) to afford the title compound (6.90 g, 27.5 mmol, 41%) as a yellow solid.

Analytical data were consistent with those reported previously in the literature.[51]

***R*f** = 0.44 (50% EtOAc in *n*-hexane), UV active, stained with $KMnO_4$;

MP: 56–58 °C (*n*-hexane/EtOAc);

^{1}H NMR (500 MHz, $CDCl_3$): δ = 8.33 (s, 1H), 7.60 – 7.54 (m, 2H), 7.49 (m, 3H), 5.23 (s, 1H), 4.30 (app. dq, *J* = 10.7, 7.1 Hz, 2H), 3.67 (br s, 1H), 1.27 (t, *J* = 7.1 Hz, 3H);

^{13}C NMR (150 MHz, $CDCl_3$): δ = 170.6, 148.0, 139.4, 131.2, 131.0, 130.0, 129.4, 66.0, 63.2, 14.1;

HRMS (ESI$^+$): Calc. for $C_{12}H_{14}N_1O_5$ $[M+H]^+$: 252.0867; found: 252.0858; calc. for $C_{12}H_{13}N_1O_5Na$ $[M+Na]^+$: 274.0686; found: 274.0696.

Screening of Linkers for the Acylation

In order to select the optimal linker, three unsymmetrical cyclic anhydrides were screened: 2,2-dimethylglutaric anhydride **16**, 2,2-dimethylsucccinic anhydride **S10** and homophthalic anhydride **S13**. In order to test their suitability, each of them was reacted with (*rac*)-nitroallylic alcohol **17** under DABCO catalysis as shown in **Fig. 2**.

Gratifyingly, the reaction with **16** afforded the desired carboxylic acid **19** as a single regioisomer as confirmed by HMBC analysis. In sharp contrast, the reaction with **S10** provided an inseparable mixture of regioisomers **S11** and **S12** in a 3:1 ratio.[52] Finally, no product formation was observed in the reaction with **S13**. Therefore, **16** was chosen as the optimal linker for the designed sequence.

Fig. 2. Screening of unsymmetrical linkers.

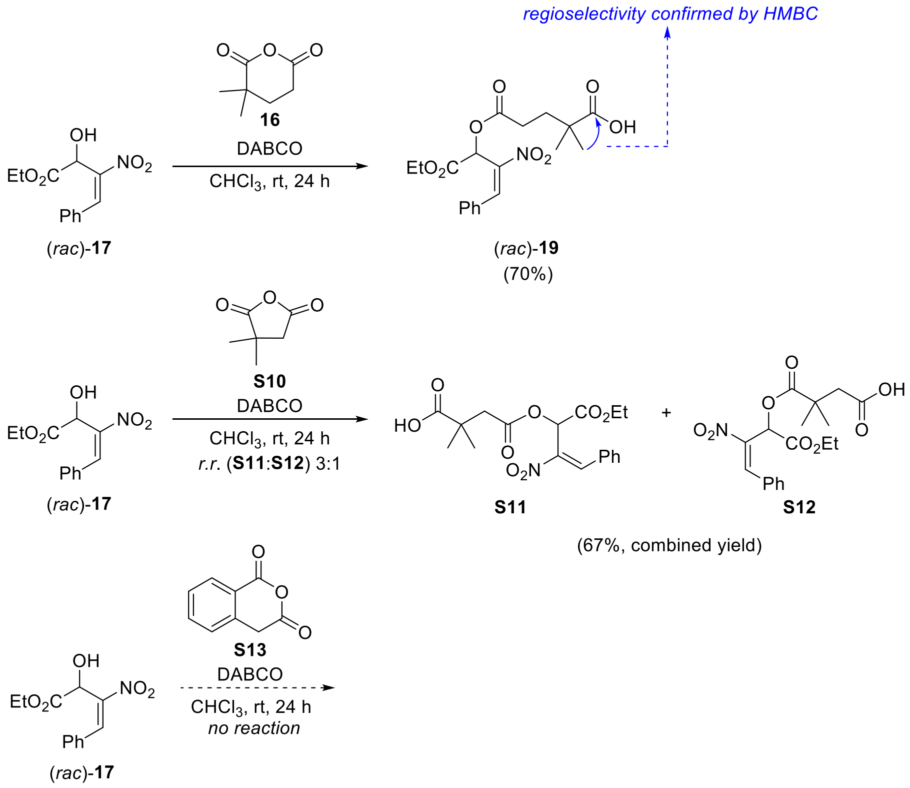

Synthesis of Racemic Carboxylic acid

(*rac*)-Carboxylic acid (19)

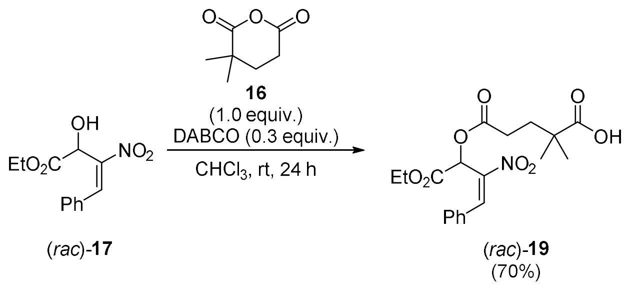

Based on conditions reported by Chen and co-workers.[35] To a solution of **17** (3.00 g, 11.9 mmol, 1.00 equiv.) in anhydrous $CHCl_3$ (31 mL), were added sequentially 2,2-dimethylglutaric anhydride **16** (1.70 g, 11.9 mmol, 1.00 equiv.) and DABCO (0.40 g, 3.58 mmol, 0.30 equiv.). The reaction mixture was stirred at rt for 24 h and was then concentrated under reduced pressure. The residue was purified by column chromatography on silica gel (33% EtOAc in *n*-hexane) to afford the title compound (3.30 g, 8.36 mmol, 70%) as a pale-yellow solid.

$\boldsymbol{R}_\mathbf{f}$ = 0.28 (50% EtOAc in *n*-hexane), UV active, stained with $KMnO_4$;

MP: 106–108 °C (*n*-hexane/EtOAc);

^{1}H NMR (500 MHz, $CDCl_3$): δ = 8.40 (s, 1H), 7.54–7.44 (m, 5H), 6.59 (s, 1H), 4.27–4.15 (m, 2H), 2.61–2.42 (m, 2H), 1.95 (tt, J = 8.5, 7.1 Hz, 2H), 1.27–1.19 (m, 9H);

^{13}C NMR (150 MHz, $CDCl_3$): δ 183.5, 171.9, 166.0, 145.0, 140.7, 131.6, 130.5, 129.9, 129.5, 65.7, 62.8, 41.6, 34.5, 29.9, 25.1, 24.8, 14.0;

IR (film, cm^{-1}): 3060, 1760, 1744, 1693, 1522, 1332, 1291, 1217, 1169, 1152, 1140, 1040, 769, 639;

HRMS (ESI$^+$): Calc. for $C_{19}H_{24}NO_8$ $[M+H]^+$: 394.1496; found: 394.1489; calc. for $C_{19}H_{23}NO_8Na$ $[M+Na]^+$: 416.1316; found: 416.1315.

4.2. Optimisation of the second esterification

Summary of Esterification Conditions

A wide range of esterification conditions were screened (**Fig. 3**), including benchmark esterification protocols with carbodiimide-based reagents, BOP and COMU reagents, acyl chloride activation, Yamaguchi[53] and Shiina esterification.[54] Most of them led to cleavage of the starting carboxylic acid **19** and/or transesterification, resulting in the formation of 2,2-dimethylglutaric acid and/or 2,2-dimethylglutaric anhydride **16**; the latter two methods led to the formation of the desired diester **20** albeit in unsatisfactory yields.

Fig. 3. Screening of esterification conditions.

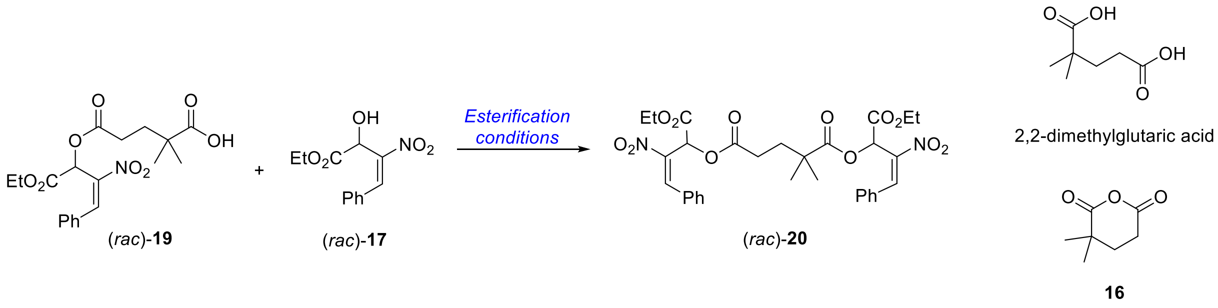

Carcbodiimide conditions
DCC, DMAP (cleavage)
EDCI, DMAP (cleavage)
EDCI, HOBt, DMAP (cleavage)
EDCI, HOBt, DIPEA (cleavage)
BOP, DIPEA (cleavage)
COMU, DBU (cleavage)

Through the acyl chloride
$(COCl)_2$ *then* pyridine/LiCl (cleavage)
$(COCl)_2$ *then* Et_3N (cleavage)
$(COCl)_2$ *then* NaH (cleavage)

Macrolactonisation conditions
MNBA, Et_3N, DMAP (**20**=25%)
$Cl_3C_6H_2COCl$, DIPEA, DMAP (**20**=33%)

Miscellaneous conditions
KPF_6 (*decomposition*)
$(NH_4)_2[Ce(NO_3)_6]$ (*n.r.*)

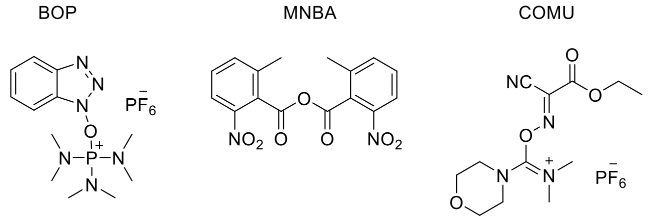

Screening of Aromatic Anhydrides for the Sc(OTf)$_3$-Catalysed Esterification

Efforts were next focused on activation of the carboxylic as a mixed anhydride. Yamamoto *et al.* have reported a protocol for the Sc(OTf)$_3$-esterification between alcohols and free carboxylic acids in the presence of aromatic anhydrides.[36] Based on that, the **Fig. 4** below summarises the screening process for the selection of the optimal aromatic anhydride, taking into account both the conversion and chemoselectivity (side-product **S14** results from the esterification of nitroalcohol **17** with the acyl group of the corresponding aromatic anhydride).[55] Thiophene-2-carboxylic anhydride **21** exhibited the optimal chemoselectivity and almost full conversion.

Fig. 4. Screening of aromatic anhydrides.

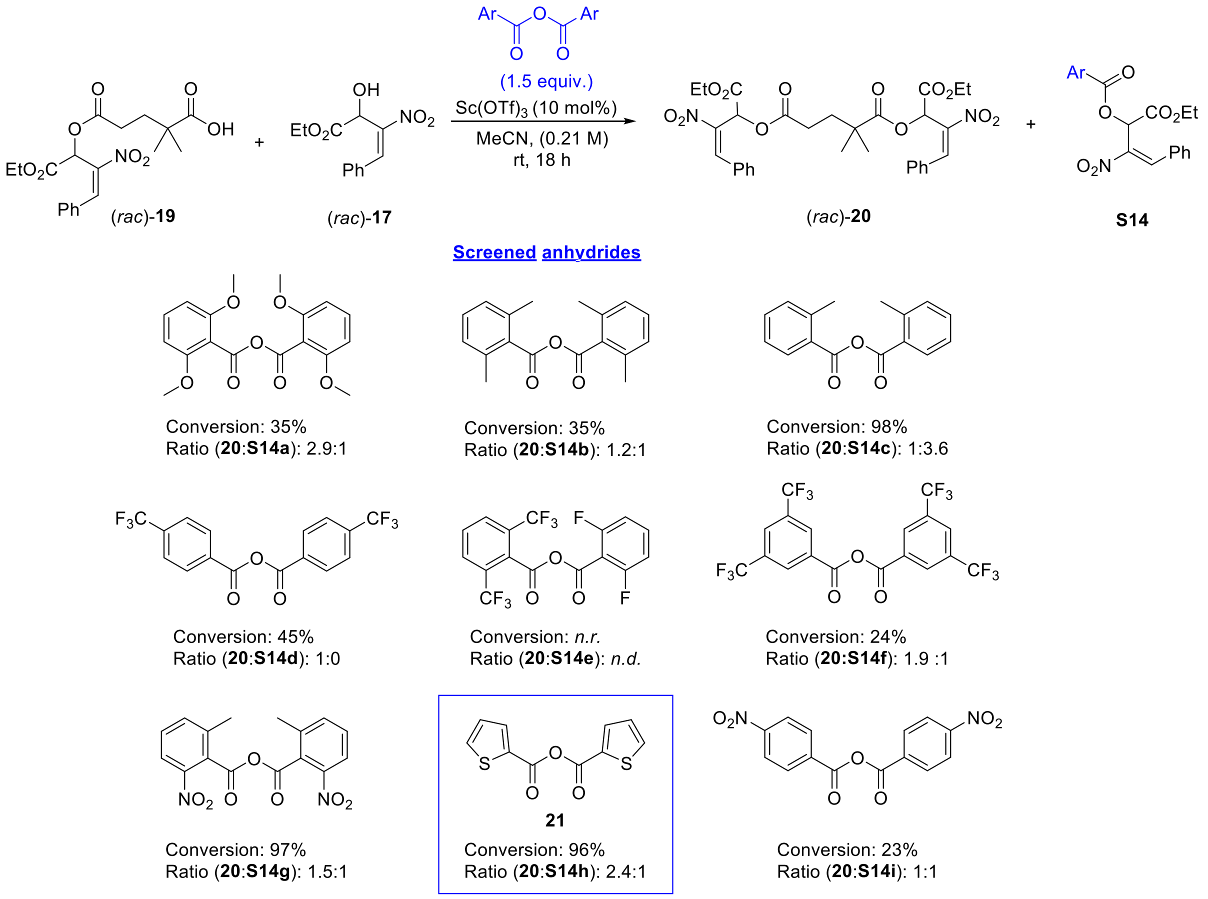

Optimisation of the $Sc(OTf)_3$-Catalysed Esterification

Table 3 below summarises the attempts to improve the observed chemoselectivity. Unfortunately, the entries with improved chemoselectivity were accompanied by significantly lower conversions.

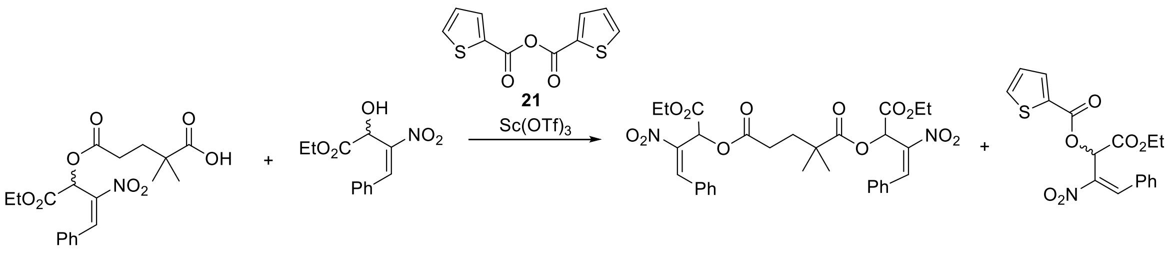

(*rac*)-**19** (*rac*)-**17** (*rac*)-**20** **S14h**

Table 3. Optimisation of Sc-catalysed esterification.

Entry	Studied parameter	Temperature	Procedure[a]	Conversion	20:S14h ratio	Notes
1	Procedure	r.t.	1	96%	2.4:1	Standard conditions.[b]
2		r.t.	2	74%	3.3:1	Alcohol added after 4 h.
3		r.t	2	*n.r.*	*n.d.*	Alcohol added after 15 h.
4	Stoichiometry	r.t.	1	22%	3.7:1	1.0 equiv. of **21**.
5		r.t.	2	30%	7.2:1	1.1 equiv. of 21. **17** added after 1 h.
6		r.t	2	43%	8.2:1	1.1 equiv. of **21**. **17** added after 4 h.
7	Temperature	0 °C	1	46%	3:1	43 h reaction time.
8	Cat. loading	r.t.	2	48%	4.6:1	25 mol% $Sc(OTf)_3$. **17** added after 4 h.
9	Solvent	r.t.	1	40%	2:1	$MeNO_2$
10		r.t.	1	traces	*n.d.*	1,4-dioxane

11	r.t.	1	*n.r.*	*n.d.*	CH_2Cl_2

[a]Procedure 1: A (0.08 M) solution of $Sc(OTf)_3$ was added dropwise to a mixture of **19**, **17** and **21**; Procedure 2: A solution of **17** was added dropwise to a pre-stirred mixture of **19**, **21** and $Sc(OTf)_3$; [b]Standard conditions refer to those in **Fig. 4**. Tp=thiophene; *n.r.* = no reaction; *n.d.* = not determined.

4.3. Synthesis of Racemic Diester

(*rac*)-Diester (20)

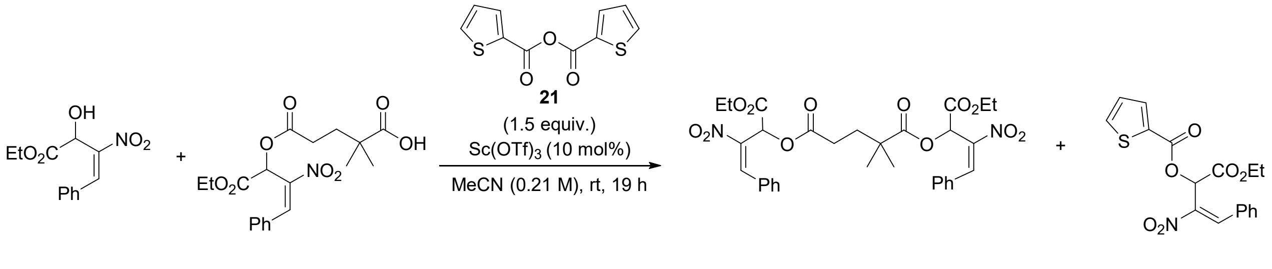

(*rac*)-**17** (*rac*)-**19** (*rac*)-**20** *d.r.* 1:1 (65%) **SI.14h** (25%)

Adapted from the conditions reported by Yamamoto and co-workers.[36] A mixture of (*rac*)-nitroalcohol **17** (52.0 mg, 0.21 mmol, 1.00 equiv.) and (*rac*)-acid **19** (82.0 mg, 0.21 mmol, 1.00 equiv.) was co-evaporated three times with toluene in a round-bottom flask and dried thoroughly under high vacuum, before thiophene-2-carboxylic anhydride **21** (74.0 mg, 0.31 mmol, 1.50 equiv.) was added. The mixture was evacuated and backfilled with N_2 three times and was then dissolved in anhydrous MeCN (1 mL) under N_2. To the resulting solution was added slowly over 2 minutes a solution of Sc(OTf)$_3$ (10.0 mg, 0.02 mmol, 0.10 equiv.) in anhydrous MeCN (0.25 mL). The reaction mixture was stirred at rt for 19 h, before it was quenched with saturated aqueous $NaHCO_3$ (2 mL). The layers were separated and the aqueous phase was extracted with EtOAc (3 × 10 mL), and the combined organic extracts were washed with brine (15 mL), dried over Na_2SO_4, filtered, and concentrated under reduced pressure. The residue was purified by column chromatography on silica gel (gradient 1.2% to 2.4% EtOAc in toluene) to afford the inseparable mixture of diastereomers of (*rac*)-**20** (*d.r.* 1:1, 85.0 mg, 0.14 mmol, 65%) as a pale yellow wax and the side-product **S14h** as a yellow viscous oil (19.0 mg, 0.05 mmol, 25%).

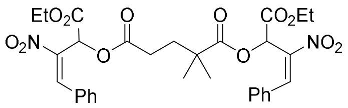

(*rac*)-**20**
d.r. 1:1

***R*f** = 0.31 (5% EtOAc in toluene), UV active, stained with $KMnO_4$;

^{1}H NMR (600 MHz, $CDCl_3$): δ = (both diastereomers) 8.44–8.35 (m, 4H), 7.55–7.42 (m, 20H), 6.58–6.52 (m, 4H), 4.24–4.13 (m, 8H), 2.67–2.42 (m, 4H), 2.08–1.91 (m, 4H), 1.25–1.17 (m, 24H);

^{13}C NMR (150 MHz, $CDCl_3$): δ = (both diastereomers) 175.5, 175.4, 171.84, 171.79, 165.99,* 165.96,* 145.03, 144.99,* 144.97, 140.71, 140.70, 140.64, 140.61, 131.6,* 131.54,* 130.53,* 130.47,* 129.9,* 129.8,* 129.51,* 129.48,* 65.82, 65.78, 65.75,* 62.82, 62.79, 62.76,* 42.08, 42.05, 34.62, 34.58, 29.80, 29.77, 25.4, 25.1, 24.9, 24.6, 14.1,* 14.0*;

Note: Due to overlapping peaks in the ^{13}C NMR spectrum, some peaks are missing/not observed. Peaks that have overlapped a second peak have been marked with an asterisk (*).

IR (neat, cm^{-1}): 1763, 1740, 1530, 1335, 1298, 1212, 1118, 1041, 1028, 766;

HRMS (ESI$^+$): Calc. for $C_{31}H_{34}N_2O_{12}Na$ [M+Na]$^+$: 649.2004; found: 649.2005.

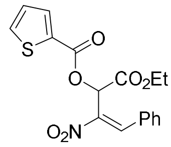

S14h

***R*f** = 0.47 (5% EtOAc in toluene), UV active, stained with $KMnO_4$;

^{1}H NMR (500 MHz, $CDCl_3$): δ = 8.47 (s, 1H), 7.89 (dd, *J* = 3.8, 1.3 Hz, 1H), 7.64 (dd, *J* = 5.0, 1.3 Hz, 1H), 7.55–7.48 (m, 5H), 7.13 (dd, J = 5.0, 3.8 Hz, 1H), 6.78 (s, 1H), 4.25 (qq, *J* = 7.1, 3.6 Hz, 2H), 1.23 (t, *J* = 7.1 Hz, 3H);

^{13}C NMR (125 MHz, $CDCl_3$): δ = 166.0, 160.6, 144.9, 140.9, 135.1, 134.1, 131.9, 131.6, 130.5, 130.0, 129.5, 128.2, 66.2, 62.9, 14.1;

IR (film, cm^{-1}): 1764, 1717, 1530, 1526, 1360, 1336, 1251, 1216, 1070, 742, 693;

HRMS (ESI$^+$): Calc. for $C_{17}H_{16}NO_6S$ [M+H]$^+$: 362.0693; found: 362.0686; calc. for $C_{17}H_{15}N_1O_6S_1Na_1$ [M+Na]$^+$: 384.0512; found: 384.0518.

4.4. Kinetic Resolution of (*rac*)-nitroalcohol 17

(*R*)-Carboxylic acid (19)

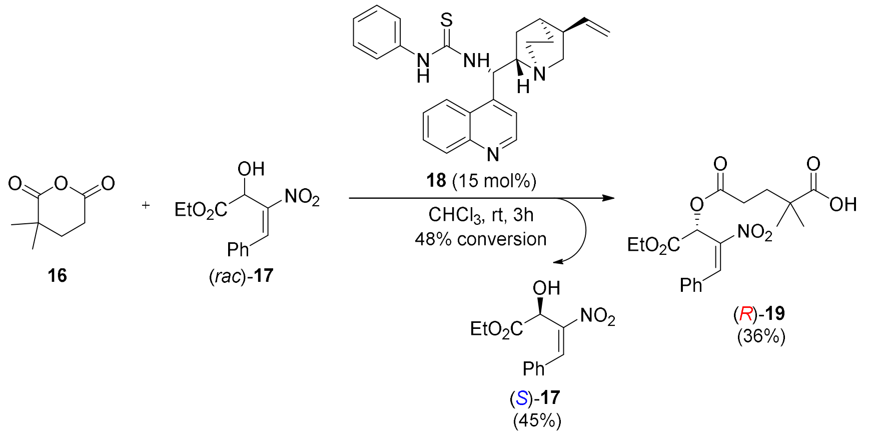

Based on conditions reported by Chen and co-workers.[35] To a solution of (*rac*)-nitroalcohol **17** (180 mg, 0.72 mmol, 1.00 equiv.) in anhydrous $CHCl_3$ (1.5 mL), were added sequentially 2,2-dimethylglutaric anhydride (61.0 mg, 0.43 mmol, 0.60 equiv.) and the chiral organocatalyst **18** (46.0 mg, 0.11 mmol, 0.15 equiv.). The reaction mixture was stirred at rt until ~50% conversion (48%, 3 h, monitored by ^{1}H-NMR spectroscopy) and was then concentrated under reduced pressure. The residue was partially purified through a short plug of silica (eluting with 50% EtOAc in *n*-hexane) to separate the mixture of (*S*)-**17** and (*R*)-**19** from the catalyst. For characterisation purposes the residue was purified by column chromatography on silica gel (gradient 12% to 66% EtOAc in *n*-hexane) to afford the enantioenriched (*S*)-nitroalcohol **17** (81.0 mg, 45%), and (*R*)-acid **19** (100 mg, 36%). Their spectroscopic data are in agreement with the respective racemic substrates (*rac*)-**17** and (*rac*)-**19**.

For (*S*)-**17**: The enantiomeric excess (87% ee) was determined by HPLC [Daicel Chiralpak® AD-H column with guard, 15% *i*-PrOH in *n*-hexane, 0.4 mL/min, 30 °C, 220 nm, t_R = 26.82 min (*R* isomer, minor), 62.68 min (*S* isomer, major)], or [Chiralcel® OD-H column with guard, 10% *i*-PrOH in *n*-hexane, 0.5 mL/min, 254 nm, t_R = 18.88 min (*R*-isomer, minor), 21.85 min (*S*-isomer, major)]. The HPLC traces and retention times of the latter method are in agreement with the ones reported in the literature for (*S*)-enriched **17**.[35]

For (*R*)-**19**: The enantiomeric excess (80% ee) was determined by HPLC [Daicel Chiralpak® AD-H column with guard, 23% *i*-PrOH in *n*-hexane, 0.5 mL/min, 254 nm, t_R = 15.27 min (*S*-isomer, minor), t_R = 17.24 min (*R*-isomer, major)].

HPLC traces of (*rac*)- and (*S*)-nitroalcohol 17

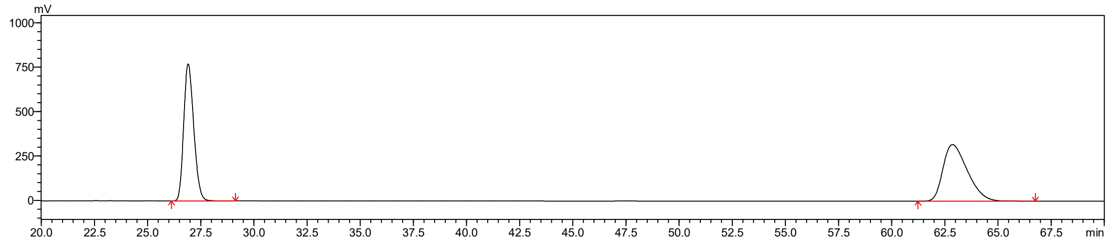

Peak#	Ret. Time	Area	Height	Conc.	Area%
1	26.817	20210103	618548	50.078	50.078
2	62.679	20147162	258159	49.922	49.922
Total		40357265	876707	100.000	100.000

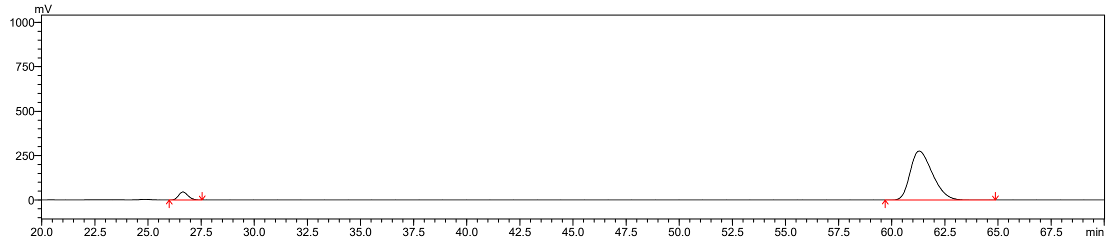

Peak#	Ret. Time	Area	Height	Conc.	Area%
1	26.645	1384206	44643	6.456	6.456
2	61.295	20057488	276206	93.544	93.544
Total		21441694	320849	100.000	100.000

HPLC traces of (*rac*)- and (*R*)-acid 19

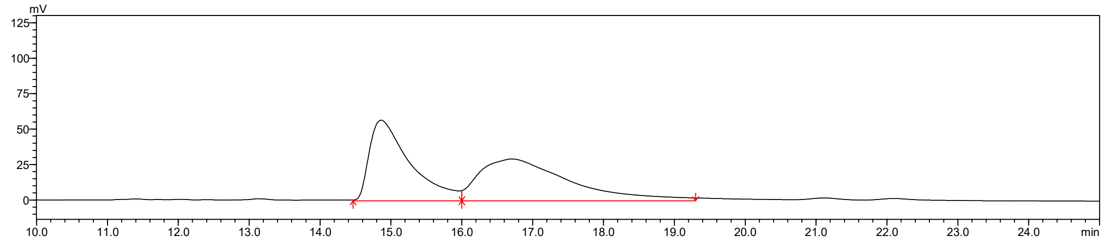

Peak#	Ret. Time	Area	Height	Conc.	Area%
1	14.861	2217690	56154	47.434	47.434
2	16.707	2457641	28795	52.566	52.566
Total		4675331	84949	100.000	100.000

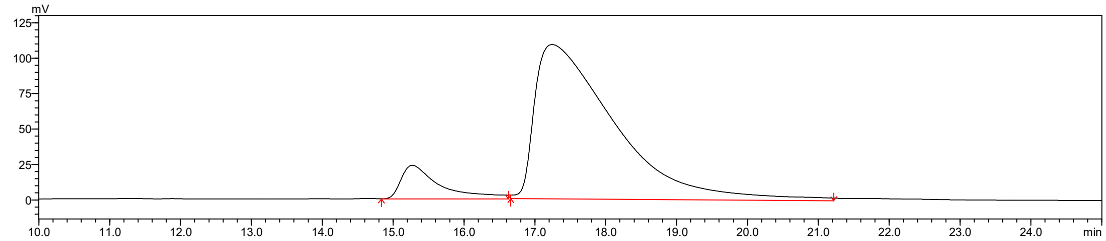

Peak#	Ret. Time	Area	Height	Conc.	Area%
1	15.271	918985	23746	9.836	9.836
2	17.243	8424349	108973	90.164	90.164
Total		9343333	132719	100.000	100.000

4.5 HPLC trace assignment of diester 20 isomers

The HPLC trace assignment for each isomer of diester **20** was conducted through a series of Sc(OTf)3-catalysed esterification reactions between scalemic mixtures of the reaction components (following the procedure described in **Section 4.3.**).

➢ (*R*,*S*) and (*R*,*R*) assignment through esterification between the (*R*)-acid **19** and (*rac*)-nitroalcohol **17**.

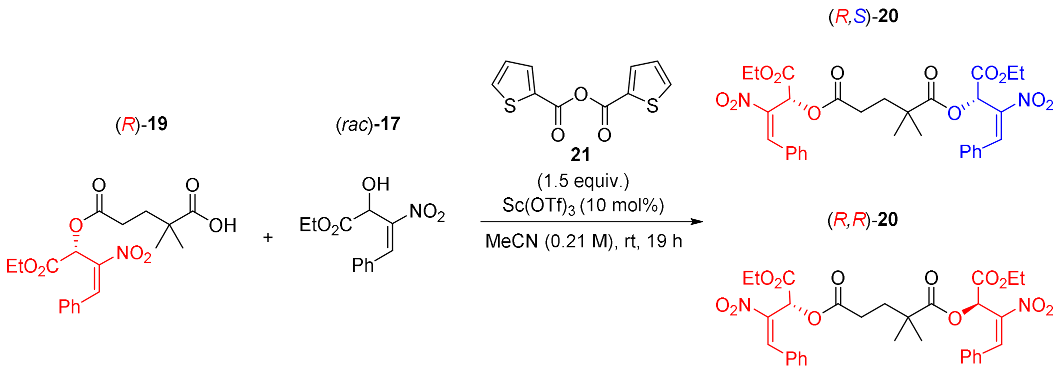

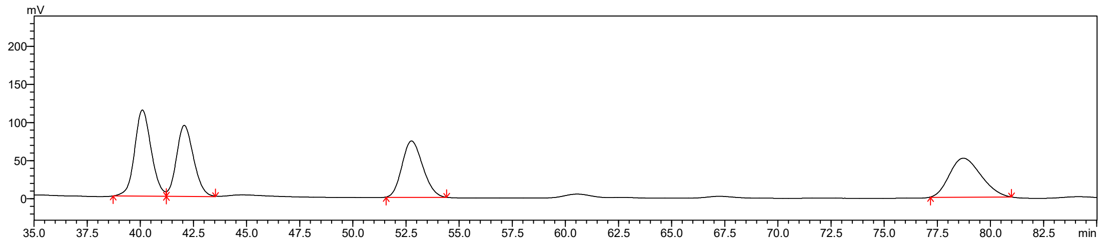

Peak#	Ret. Time	Area	Height	Conc.	Area%
1	40.098	5363859	107210	25.743	25.743
2	42.070	5118721	92551	24.566	24.566
3	52.761	5044260	74001	24.209	24.209
4	78.729	5309355	51279	25.481	25.481
Total		20836195	325042	100.000	100.000

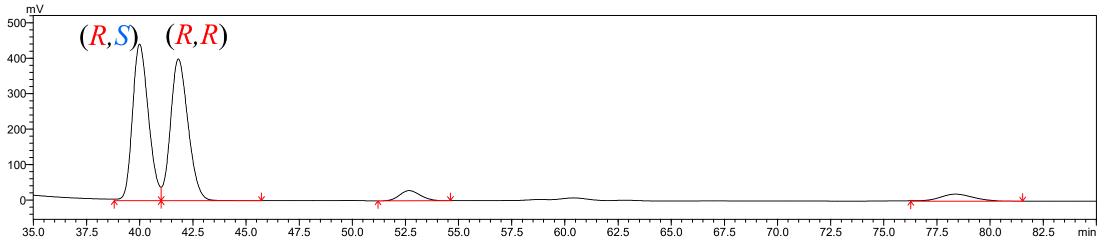

Peak#	Ret. Time	Area	Height	Conc.	Area%
1	39.992	23082670	441708	46.106	46.106
2	41.818	22848501	400020	45.638	45.638
3	52.674	1949539	28630	3.894	3.894
4	78.372	2183896	19792	4.362	4.362
Total		50064605	890149	100.000	100.000

➢ (*R*,*S*) and (*S*,*S*) assignment through esterification between the (*rac*)-acid **19** and (*S*)-nitroalcohol **17**.

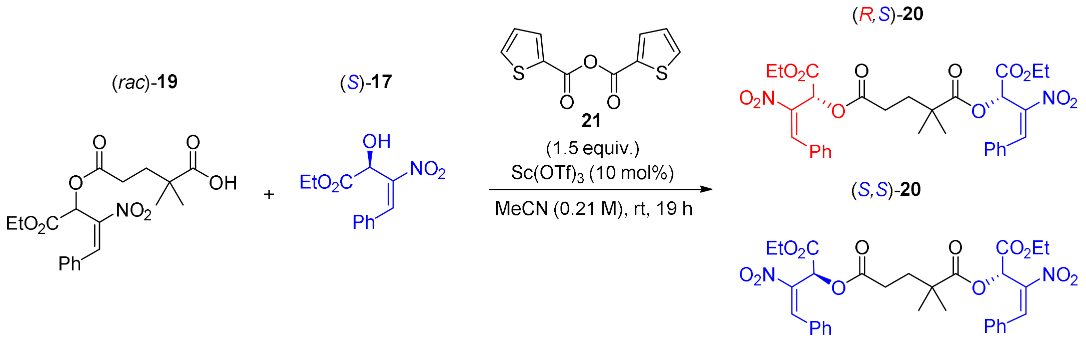

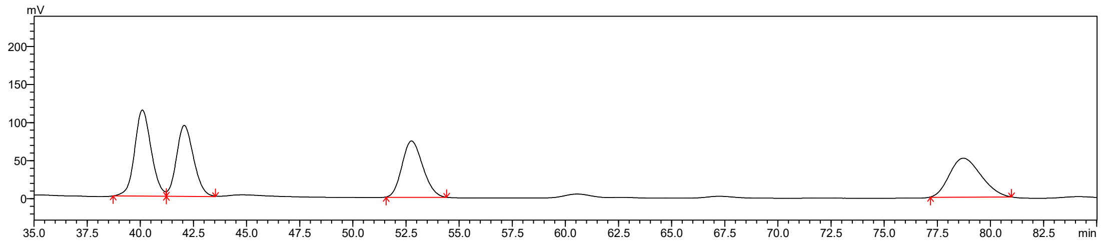

Peak#	Ret. Time	Area	Height	Conc.	Area%
1	40.098	5363859	107210	25.743	25.743
2	42.070	5118721	92551	24.566	24.566
3	52.761	5044260	74001	24.209	24.209
4	78.729	5309355	51279	25.481	25.481
Total		20836195	325042	100.000	100.000

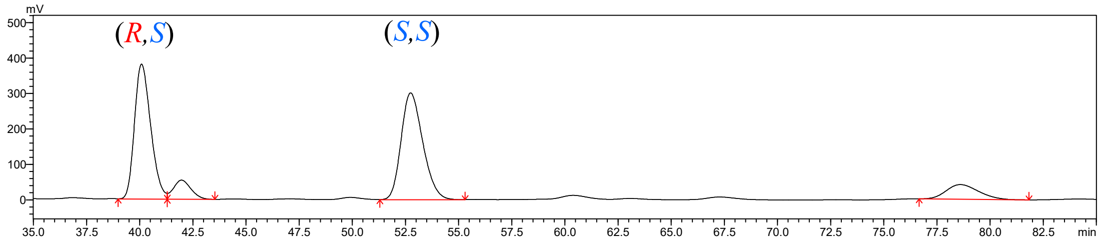

Peak#	Ret. Time	Area	Height	Conc.	Area%
1	40.087	20261181	380931	41.255	41.255
2	41.967	3077661	53892	6.267	6.267
3	52.742	21394409	301341	43.562	43.562
4	78.604	4379136	41328	8.917	8.917
Total		49112387	777492	100.000	100.000

➢ (*S*,*S*) and (*S*,*R*) assignment through esterification between the (*S*)-acid **19** and (*rac*)-nitroalcohol **17**.

The (*S*)-acid **19** was prepared from the corresponding (*S*)-nitroalcohol **17** according to the experimental procedure in **Section 4.2**.

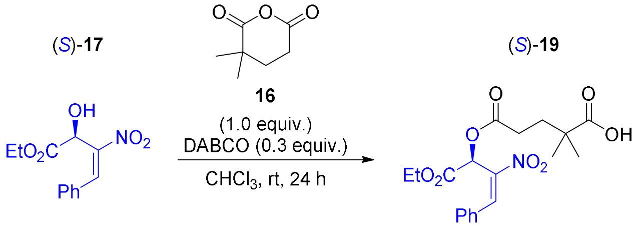

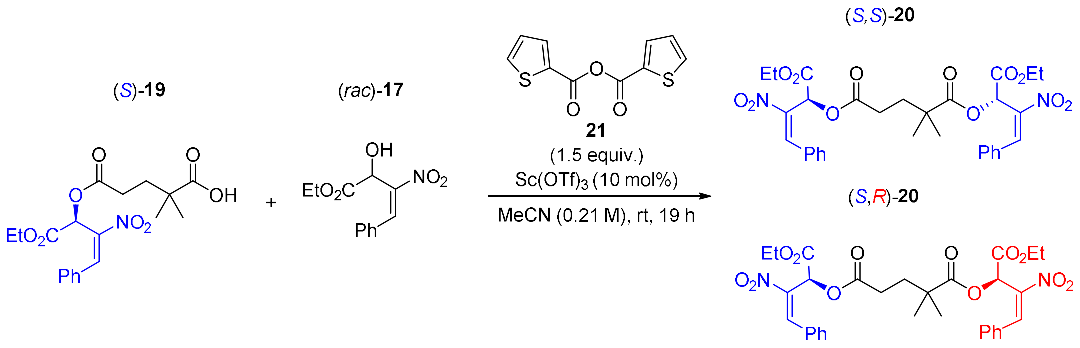

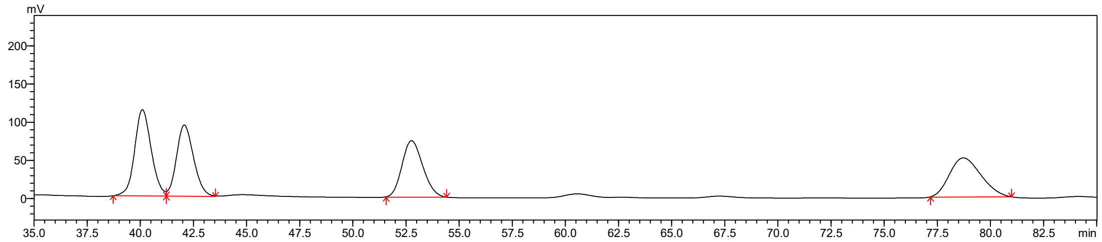

Peak#	Ret. Time	Area	Height	Conc.	Area%
1	40.098	5363859	107210	25.743	25.743
2	42.070	5118721	92551	24.566	24.566
3	52.761	5044260	74001	24.209	24.209
4	78.729	5309355	51279	25.481	25.481
Total		20836195	325042	100.000	100.000

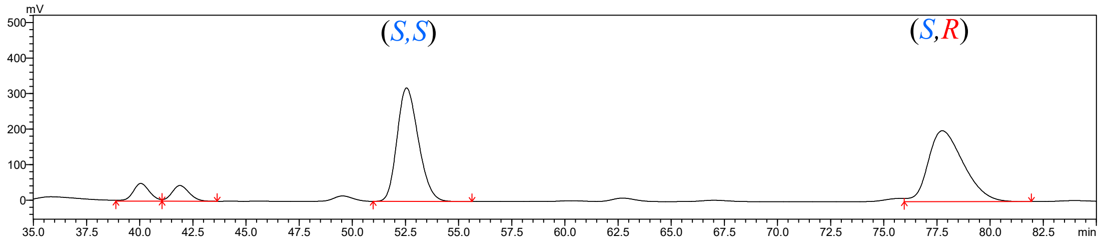

Peak#	Ret. Time	Area	Height	Conc.	Area%
1	40.052	2743891	50116	5.422	5.422
2	41.891	2501727	44289	4.944	4.944
3	52.551	22346593	319616	44.161	44.161
4	77.755	23010163	199584	45.472	45.472
Total		50602374	613606	100.000	100.000

4.6. Synthesis of Enantioenriched Diester

Synthesised according to the procedure of **Section 4.3.** The spectroscopic data were in agreement with those of the corresponding racemic diester (*rac*)-**20**.

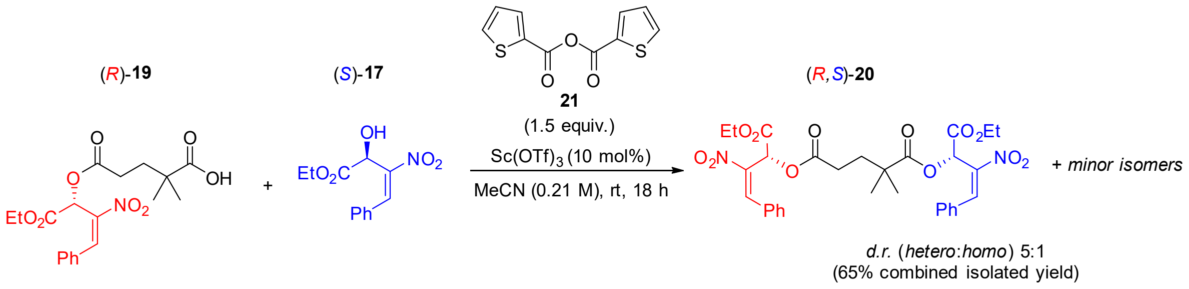

The enantiomeric excess (98.2%) for the heterochiral **20** was determined by HPLC [Daicel Chiralcel® AD-H column with guard, 15% *i*-PrOH in *n*-hexane, 0.4 mL/min, 30 °C, 220 nm, t_R = 39.21 min (*R,S*-isomer, major), t_R =76.89 min (*S,R*-isomer, minor)].

HPLC traces of enantioenriched diester **20**

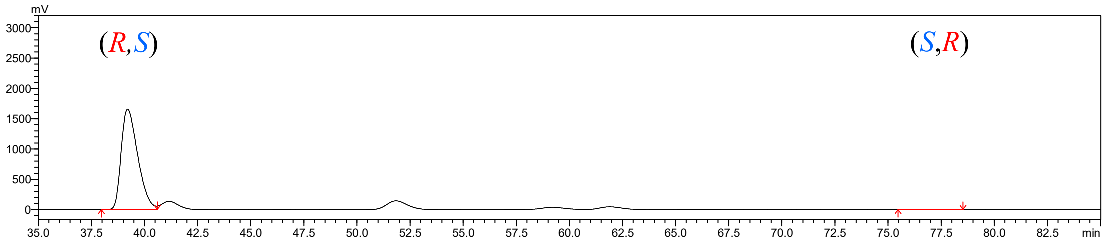

Peak#	Ret. Time	Area	Height	Conc.	Area%
1	39.205	89953346	1658147	99.104	99.104
2	76.889	813685	8707	0.896	0.896
Total		90767031	1666854	100.000	100.000

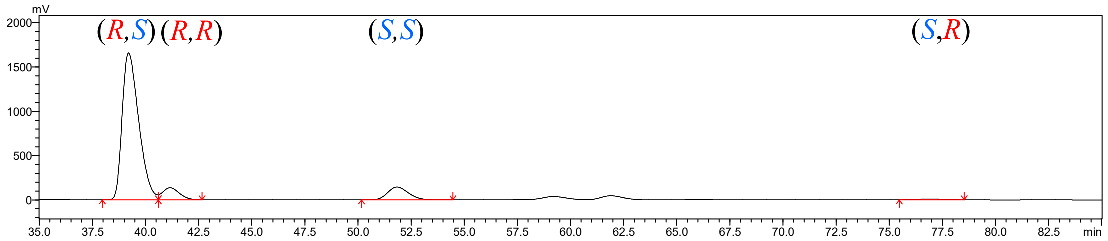

Peak#	Ret. Time	Area	Height	Conc.	Area%
1	39.205	89953346	1658147	83.142	83.142
2	41.158	7548732	136489	6.977	6.977
3	51.838	9876428	144991	9.129	9.129
4	76.889	813685	8707	0.752	0.752
Total		108192191	1948334	100.000	100.000

The *d.r.* (*hetero*:*homo*) was determined by the HPLC chromatogram integrations and was further confirmed by quantitative ^{13}C NMR experiment with relaxation time t_1=14 sec (based on the distinctive, highlighted in red methyl carbons of the spectrum below). The calculated *d.r.* value (5.1:1) via this method is consistent with the one calculated from HPLC analysis (**Fig. 5**).

Fig. 5. Determination of diastereomeric ratio by quantitative ^{13}C NMR (125 MHz, $CDCl_3$).

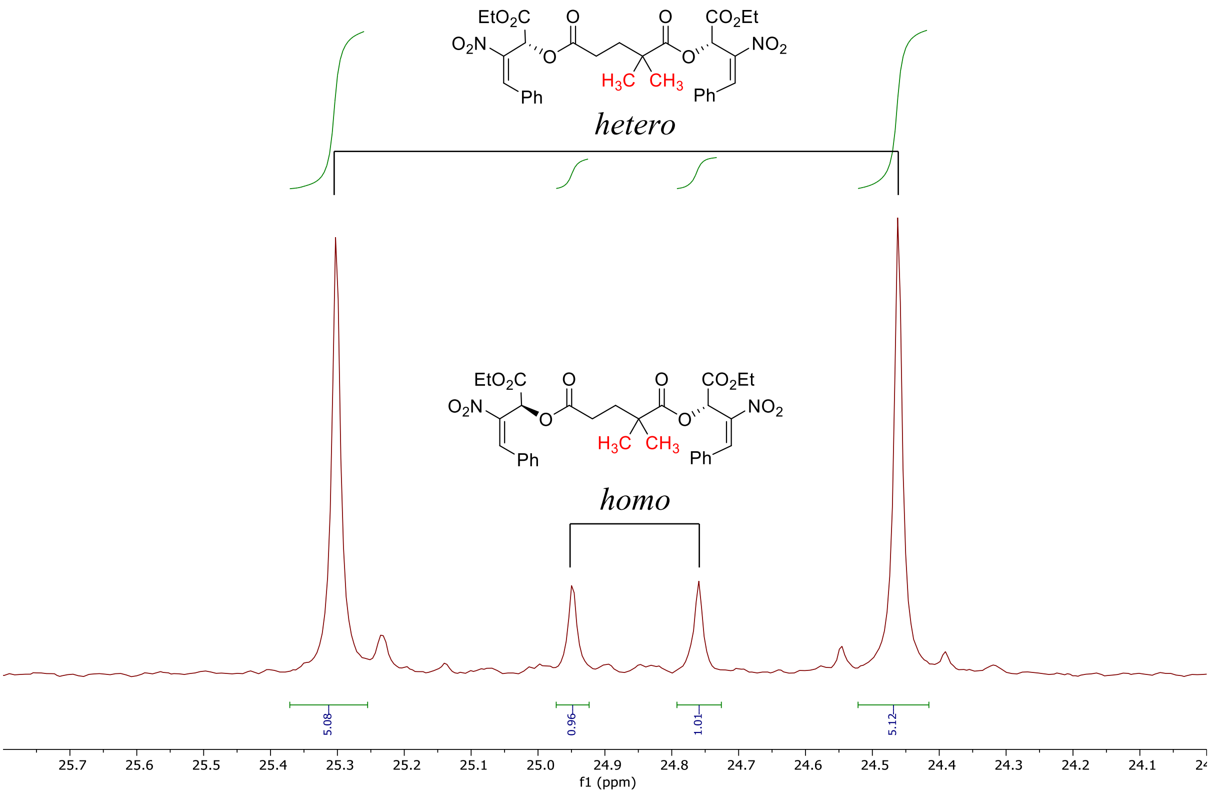

5. Organocatalytic Stereoretentive Diastereoconvergent Reaction

Note: Attempts to identify a suitable achiral unsymmetrical bis-nucleophilic linker to utilize Deng and co-worker's $(DHQD)_2AQN$ catalytic system for enantioconvergent multi-component reactions was unsuccessful.[38] When using 3-methyl-1,3-butanediol no dimer was observed and when using 4-hydroxy benzyl alcohol or ethanolamine there was insufficient selectivity between the nucleophilic sites. Other potential linkers such as 4- (hydroxymethyl)cyclohexan-1-ol or 4-(hydroxymethyl)cyclobutanol proved too difficult to readily access in diastereopure form. In summary, further work will be required to identify suitable achiral bis-nucleophilic linkers for this particular catalytic system. Nevertheless, use of (*R*)/(*S*)-1,2-propanediol demonstrates that triple stereodifferentiation can be observed and leveraged using this $(DHQD)_2AQN$ catalytic system.

5.1. Preparation of starting materials

N-carboxyanhydride (S15)

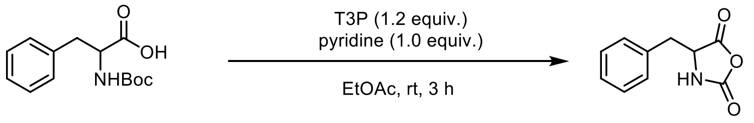

S15
(73%)

Based on the procedure reported by Martinez and co-workers.[56] *N*-Boc-DL-phenylalanine (3.00 g, 11.0 mmol, 1.00 equiv.) was dissolved in ethyl acetate (225 mL) at room temperature. Propyl phosphonic anhydride (T3P) (50% w/w in EtOAc, 8.40 g, 13.2 mmol, 1.20 equiv.) was added followed by pyridine (0.89 mL, 11.0 mmol, 1.00 equiv.). The resulting solution was stirred at room temperature for 3 h. Following this, 150 mL of ice water was added. The biphasic mixture was partitioned and the organic phase was washed with brine (2 × 150 mL), dried over $MgSO_4$ and concentrated under reduced pressure to give a wet beige solid, which was washed with cold *n*-hexane (15 mL) and filtered. The desired *N*-carboxyanhydride (1.53 g, 8.00 mmol, 73%) was obtained as a white solid after filtration.

Analytical data were consistent with those reported in the literature.[57]

^{1}H NMR (500 MHz, $CDCl_3$): δ = 7.39–7.29 (m, 3H), 7.22–7.16 (m, 2H), 6.12 (s, 1H), 4.53 (ddd, *J* = 8.2, 4.1, 0.9 Hz, 1H), 3.28 (dd, *J* = 14.1, 4.2 Hz, 1H), 3.01 (dd, *J* = 14.1, 8.3 Hz, 1H);

^{13}C NMR (126 MHz, $CDCl_3$): δ = 168.8, 151.8, 134.1, 129.4, 129.3, 128.2, 59.0, 38.0.

***N*-Cbz-N-carboxyanhydride (23)**

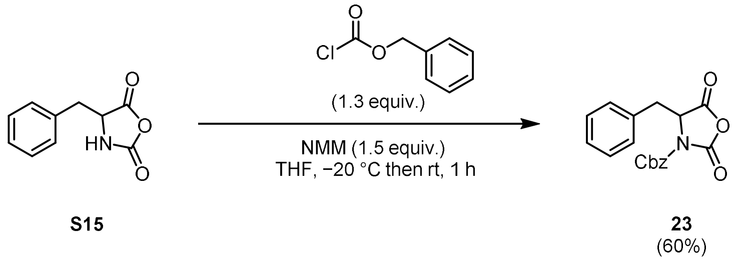

Based on the procedure reported by Naider and co-workers.[58] A solution of *N*-carboxyanhydride (1.50 g, 7.80 mmol, 1.00 equiv.) in dry THF (20 mL) was cooled to −20 °C to which benzyl chloroformate (1.45 mL, 10.1 mmol, 1.30 equiv.) was added. Then, a solution of *N*-methylmorpholine (NMM) (1.29 mL, 11.7 mmol, 1.50 equiv.) in dry THF (5 mL) was added slowly over 10 min. After addition, the reaction mixture was warmed up to room temperature and stirred for 1 h. Following this, the reaction mixture was cooled back to −20 °C and acidified with HCl (4 M in 1,4-dioxane) to pH = 3 (*ca.* 1.5 mL). The suspension was then filtered on Celite®, and the solid was washed with dry THF (4 × 10 mL). The filtrate was then concentrated under reduced pressure. The pale white solid was recrystallised from hot TBME (40 mL) and hot *n*-hexane (15 mL), where crystallisation occurred at −20 °C overnight. The white crystals were filtered, washed with cold *n*-hexane and dried to give the desired *N*-carboxybenzyl carboxyanhydride (1.53 g, 4.71 mmol, 60%).

Analytical data were consistent with those reported in the literature.[59]

^{1}H NMR (500 MHz, $CDCl_3$): δ = 7.49–7.40 (m, 5H), 7.30–7.18 (m, 3H), 6.94–6.88 (m, 2H), 5.41 (s, 2H), 4.94 (dd, *J* = 5.8, 2.6 Hz, 1H), 3.48 (dd, *J* = 14.3, 5.8 Hz, 1H), 3.30 (dd, *J* = 14.3, 2.6 Hz, 1H);

^{13}C NMR (101 MHz, $CDCl_3$): δ = 165.5, 149.4, 145.7, 134.3, 132.1, 129.5, 129.32, 129.28, 129.0, 128.9, 128.4, 70.0, 61.1, 35.2.

5.2. Procedure for the Kinetic Resolution

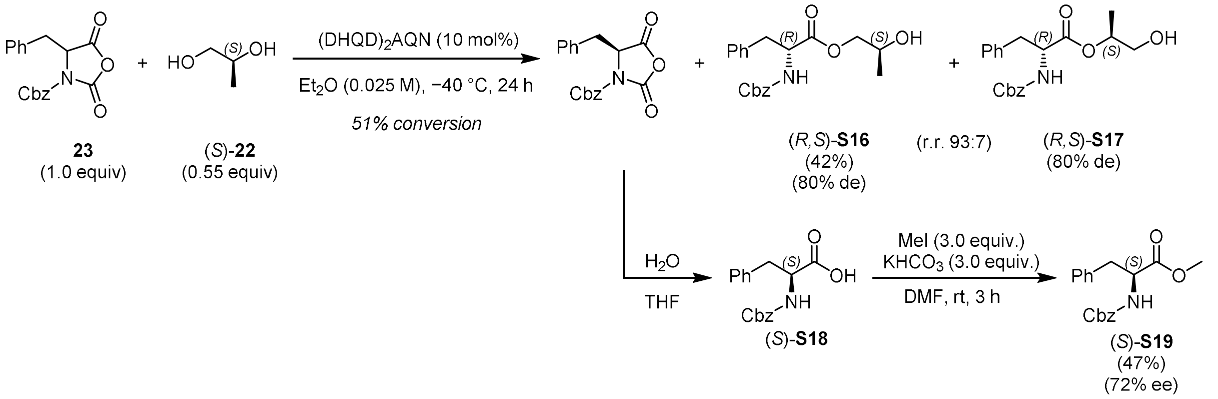

A 10 mL Schlenk tube was charged with *N*-carboxybenzyl carboxyanhydride **23** (65 mg, 0.20 mmol, 1.00 equiv.) and placed under high vacuum overnight. Following this, the Schlenk tube was filled with N_2 and dry Et_2O (6.0 mL) was added and the mixture was stirred for 20 min, until *N*-carboxybenzyl carboxyanhydride was fully dissolved. Molecular sieves 4Å (powdered, 50 mg) were then added and the solution was stirred for 15 min at room temperature. Following this, $(DHQD)_2AQN$ (17 mg, 0.02 mmol, 10 mol%) was added and the reaction was cooled down to −40 °C. At this temperature, 1.0 mL of a stock solution of (*S*)-1,2-propanediol (8.4 mg, 0.11 mmol, 0.55 equiv.) in dry Et_2O was added and the reaction mixture was stirred at −40 °C for 24 h. The following day, HCl in Et_2O (2 M, 1.0 mL) was added at −40 °C. The reaction mixture was stirred for five additional minutes and was then brought back to room temperature. The reaction mixture was then washed with aqueous HCl (2 M, 2 × 3 mL), further with brine (5 mL), dried over Na_2SO_4 and concentrated under reduced pressure to give a colourless oil as a crude residue. (Conversion was measured at this stage by ^1H NMR spectroscopy using 1,2-dimethoxyethane as internal standard.)

The crude residue was then dissolved in THF (8 mL) and H_2O (2 mL). The solution was left to stir for 16 h at room temperature. Following this, THF was removed under reduced pressure and diethyl ether (10 mL) was added to the resulting aqueous phase. The organic phase was then washed with aqueous Na_2CO_3 (1 M, 2 × 5 mL), dried over Na_2SO_4, filtered and concentrated under reduced pressure to give the crude ester (26 mg, 0.08 mmol, 42%, 80% de) as an inseparable 93:7 mixture of both regioisomers **S16:S17**.[A] The aqueous phase was acidified with concentrated aqueous HCl to pH = 4, extracted with EtOAc (3 × 10 mL), dried over Na_2SO_4 and concentrated under reduced pressure to give the crude acid (29 mg, 0.1 mmol) as a colourless oil.

Subsequently, the crude acid was dissolved in dry DMF (1.4 mL). $KHCO_3$ (41 mg, 0.41 mmol, 4.10 equiv.) was added followed by MeI (26 μL, 0.41 mmol, 4.10 equiv.) and the reaction

mixture was stirred at room temperature for 3 h. Following this, the reaction was diluted with EtOAc (10 mL) and H_2O (5 mL). The organic phase was washed with H_2O (3 × 5 mL), then with aqueous Na_2CO_3 (1 M, 5 mL) and brine (5 mL). The organic phase was dried over Na_2SO_4, filtered and concentrated under reduced pressure to give the crude methyl ester **S19** (29.8 mg, 0.09 mmol, 47%, 72% ee) as a colourless oil that was used without further purification.

Note: (**A**) Attempts to purify **S16** by flash chromatography were thwarted by acyl 1,4-migration which gave ester **S17**.

N-Cbz-DL-phenylalanine ester ((*R*,*S*)-S16)

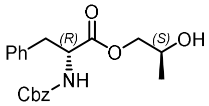

(*R*,*S*)-**S16**

***R*f** = 0.42 (50% EtOAc in *n*-hexane), stained with $KMnO_4$;

^{1}H NMR (500 MHz, $CDCl_3$): δ = 7.39–7.22 (m, 8H), 7.15 (d, *J* = 6.9 Hz, 2H), 5.26–5.18 (m, 1H), 5.10 (t, *J* = 3.2 Hz, 2H), 4.63 (q, *J* = 6.7 Hz, 1H), 4.11 (dd, *J* = 14.8, 8.0 Hz, 1H), 3.93 (dd, *J* = 11.9, 6.4 Hz, 2H), 3.18 – 3.06 (m, 2H), 1.92 (br s, 1H), 1.13 (d, *J* = 6.3 Hz, 3H);

^{13}C NMR (126 MHz, $CDCl_3$): δ = 171.7, 156.0, 136.2, 135.9, 129.4, 128.9, 128.7, 128.4, 128.3, 127.4, 70.6, 67.3, 65.8, 55.3, 38.5, 18.9;

HRMS (ESI$^+$): Calc. for $C_{20}H_{23}NO_5Na$ $[M+Na]^+$: 380.1468, found: 380.1459;

IR (thin film, cm^{-1}): 3346, 2976, 1701, 1514, 1497, 1263, 1249, 1052, 1027, 733;

HPLC: Daicel Chiralpak® IA column with guard, gradient 5% *i*-PrOH in *n*-hexane over 45 min; 5–18% *i*-PrOH in *n*-hexane over 20 min; 18% *i*-PrOH in *n*-hexane over 45 min; 1.0 mL/min, 40 °C, 220 nm, t_R = 41.34 min (*S,S*-isomer), t_R = 45.95 min (*R,S*-isomer), 80% de.

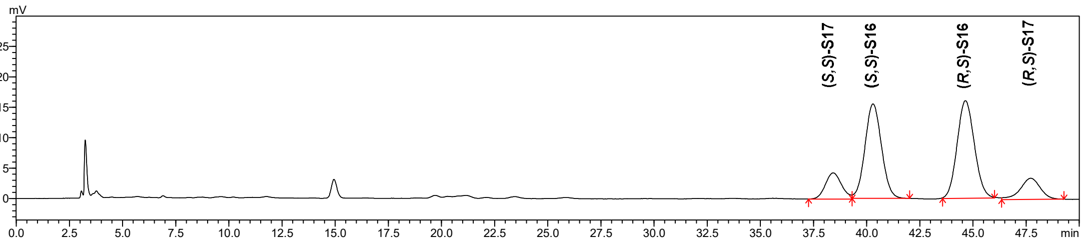

Peak#	Ret. Time	Area	Height	Conc.	Area%
1	38.427	228491	4303	10.364	10.364
2	40.296	842793	15538	38.229	38.229
3	44.646	908331	16034	41.201	41.201
4	47.724	225005	3463	10.206	10.206
Total		2204620	39338	100.000	100.000

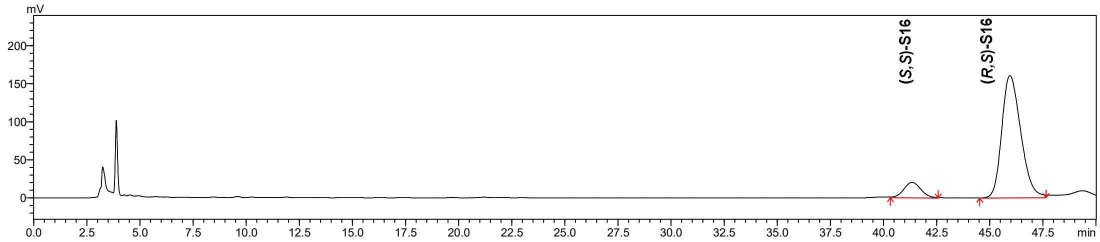

Peak#	Ret. Time	Area	Height	Conc.	Area%
1	41.342	1150840	20397	10.304	10.304
2	45.952	10017606	160771	89.696	89.696
Total		11168446	181168	100.000	100.000

***N*-Cbz-DL-phenylalanine methyl ester ((*S*)-S19)**

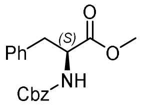

(*S*)-**S19**

Analytical data were consistent with those reported in the literature.[60]

^{1}H NMR (400 MHz, $CDCl_3$): δ = 7.41–7.19 (m, 8H), 7.13–7.06 (m, 2H), 5.22 (d, *J* = 7.4 Hz, 1H), 5.10 (d, *J* = 3.5 Hz, 2H), 4.67 (dt, *J* = 8.3, 5.9 Hz, 1H), 3.72 (s, 3H), 3.11 (qd, *J* = 13.7, 6.0 Hz, 2H);

^{13}C NMR (126 MHz, $CDCl_3$): δ = 172.1, 155.8, 136.4, 135.8, 129.4, 128.8, 128.7, 128.3, 128.2, 127.3, 67.1, 54.9, 52.4, 38.4;

HPLC: Daicel Chiralcel® OD-H column with guard, gradient 5% *i*-PrOH in *n*-hexane; 1.0 mL/min, 30 °C, 220 nm, t_R = 22.02 min (*S*-isomer), t_R = 26.84 min (*R*-isomer), 72% ee.

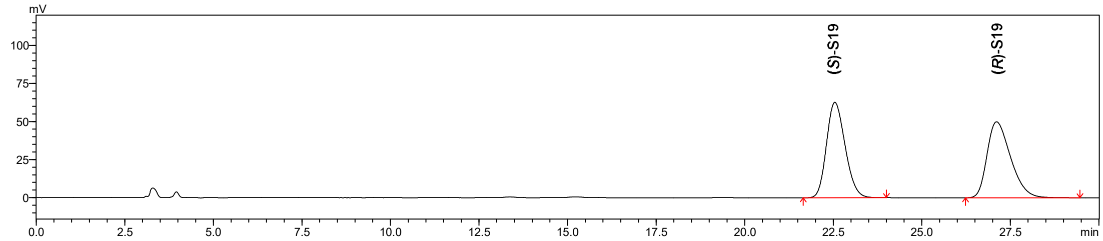

Peak#	Ret. Time	Area	Height	Conc.	Area%
1	22.545	2297856	62727	49.841	49.841
2	27.111	2312476	49983	50.159	50.159
Total		4610332	112709	100.000	100.000

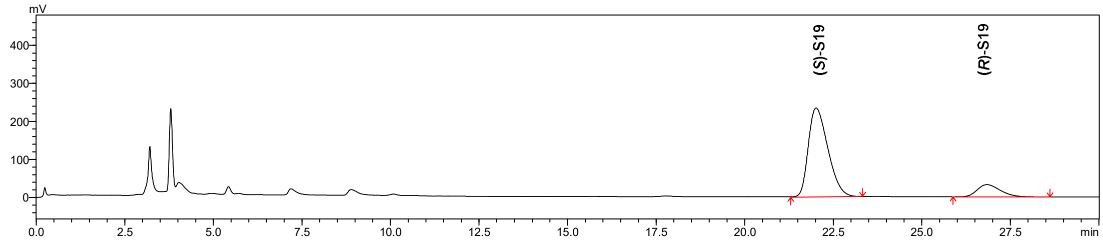

Peak#	Ret. Time	Area	Height	Conc.	Area%
1	22.016	8889289	232675	85.915	85.915
2	26.841	1457281	32069	14.085	14.085
Total		10346570	264744	100.000	100.000

5.3. Procedure for the Stereoretentive Diastereoconvergent Coupling:

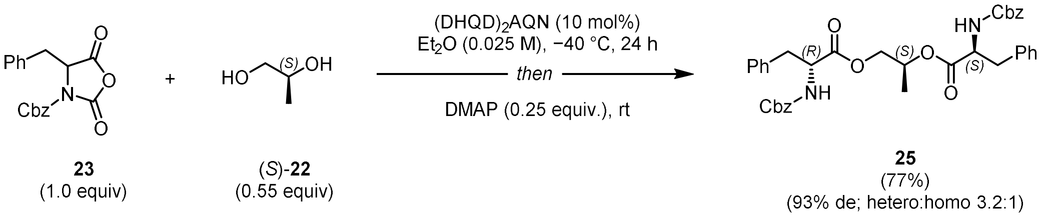

A 10 mL Schlenk tube was charged with *N*-carboxybenzyl carboxyanhydride **23** (65 mg, 0.20 mmol, 1.00 equiv.) and placed under high vacuum overnight. Following this, the Schlenk tube was filled with N_2 and dry Et_2O (6.0 mL) was added and the mixture was stirred for 20 min, until *N*-carboxybenzyl carboxyanhydride was fully dissolved. Molecular sieves 4Å (powdered, 50 mg) were then added and the solution was stirred for 15 min at rt. Following this, $(DHQD)_2AQN$ (17 mg, 0.02 mmol, 10 mol%) was added and the reaction was cooled down to −40 °C. At this temperature, 1.0 mL of a stock solution of (*S*)-1,2-propanediol **22** (8.4 mg, 0.11 mmol. 0.55 equiv.) in dry Et_2O was added and the reaction mixture was stirred at −40 °C for 24 h.[A] The following day DMAP (6 mg, 0.05 mmol, 0.25 equiv.) was added and the reaction mixture was brought back to room temperature and stirred for an additional 24 h. Following this, the reaction was cooled to 0 °C and HCl in Et_2O (2 M, 1.0 mL) was added. The reaction mixture was then washed with aqueous HCl (2 M, 2 × 3 mL), further with brine (5 mL), dried over Na_2SO_4 and concentrated under reduced pressure to give a colourless oil as a crude residue.

To accurately determine the diastereoisomeric ratios of products, the crude reaction mixture had to be analysed by chiral stationary phase HPLC using an internal standard for quantification. Therefore, a stock solution of this crude residue in *i*-PrOH (5 mL) was prepared. An analytical solution of the crude residue in *i*-PrOH (2 mL) was prepared from 500 μL of stock solution and 500 μL of stock solution of internal standard (*R,R*)-**S22** (4.0 mg/mL).[B] The desired product was found to be formed in 93% de, hetero:homo 3.2:1.

Following this, samples were combined and purified by column chromatography on silica gel (5–20% EtOAc in *n*-hexane) to give the desired dimer **25** (50 mg, 0.08 mmol, 77% yield, 93% de, hetero:homo 3.2:1) as a colourless oil.[C] The diastereoisomers can be further separated by PTLC (20% EtOAc in *n*-hexane) to give the dimers (99.5% de, hetero:homo 8:1).[D]

Notes: **(A)** On a 1.0 mmol scale, (*S*)-1,2-propanediol **22** (41.8 mg, 0.55 mmol. 0.55 equiv.) was dissolved in dry Et_2O (5 mL) and added slowly over 5 min. **(B)** For a 1.0 mmol scale reaction, the crude product (455 mg) was dissolved in a minimum of CH_2Cl_2 and an aliquot was taken and concentrated under reduced pressure. The resulting crude material (67.8 mg) was used for

the preparation of a stock solution in *i*-PrOH (5 mL). An analytical solution of the crude residue in *i*-PrOH (2 mL) was prepared from 500 μL of stock solution and 500 μL of stock solution of internal standard (4.0 mg/mL). Diastereoisomeric ratios were determined for the crude sample (92% de, hetero:homo 2.8:1). **(C)** On a 1.0 mmol scale, samples were combined and purified by column chromatography on silica gel (5–20% EtOAc in *n*-hexane) to give the desired dimer **25** (276 mg, 0.43 mmol, 86% yield, 92% de, hetero:homo 2.8:1) as a colourless oil. **(D)** Preparation of an equimolar mixture of the racemic diastereoisomer was performed on 0.2 mmol scale following the same procedure at room temperature, using NEt_3 (60.0 μL, 0.44 mmol, 2.20 equiv.) instead of $(DHQD_2)AQN$.

The following characterisation were recorded for stereopure samples prepared through the procedures reported **Section 5.4**.

Heterochiral dimer ((*R*,*S*,*S*)-25)

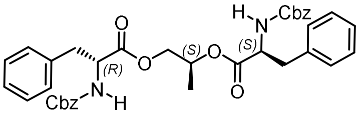

(*R*,*S*,*S*)-**25**

***R*f** = 0.15 (30% EtOAc in *n*-hexane), stained with $KMnO_4$;

MP: 103–105 °C (*n*-hexane/EtOAc);

^{1}H NMR (500 MHz, $CDCl_3$): δ = 7.39–7.19 (m, 16H), 7.16–7.08 (m, 4H), 5.27 (d, *J* = 8.4 Hz, 1H), 5.23 (d, *J* = 8.3 Hz, 1H), 5.18–5.00 (m, 5H), 4.64 (td, *J* = 8.5, 4.3 Hz, 2H), 4.11 (qd, *J* = 11.9, 4.7 Hz, 2H), 3.12 (ddd, *J* = 14.8, 9.2, 5.8 Hz, 2H), 3.03 (dd, *J* = 14.0, 6.7 Hz, 2H), 1.20 (d, *J* = 6.5 Hz, 3H);

^{13}C NMR (126 MHz, $CDCl_3$): δ = 171.4, 171.0, 155.80, 155.77, 136.4, 136.3, 135.8, 135.7, 129.4, 129.3, 128.79, 128.74, 128.6, 128.3, 128.22, 128.20, 127.32, 127.30, 69.5, 67.13, 67.10, 66.7, 55.0, 54.9, 38.12, 38.09, 16.4;

HRMS (ESI$^+$): Calc. for $C_{37}H_{38}N_2O_8Na$ $[M+Na]^+$: 661.2520, found: 661.2494;

IR (thin film, cm^{-1}): 3032, 2960, 2925, 1739, 1511, 1497, 1456, 1259;

$[\alpha]_D^{25}$= +3.8 (*c* = 0.62, $CHCl_3$);

HPLC: Daicel Chiralpak® IA column with guard, gradient 5% *i*-PrOH in *n*-hexane over 45 min; 5–18% *i*-PrOH in *n*-hexane over 20 min; 18% *i*-PrOH in *n*-hexane over 45 min; 1.0 mL/min, 40 °C, 220 nm, t_R = 85.92 min (*R,S,R* isomer), t_R = 88.68 min (*S,S,R* isomer), t_R = 90.35 min (*S,S,S* isomer), t_R = 96.68 min (*internal standard* (*R,R*)-**S22**), t_R = 99.39 min (*R,S,S* isomer), 92% de, hetero:homo 2.8:1.

Heterochiral dimer ((*S,S,R*)-25)

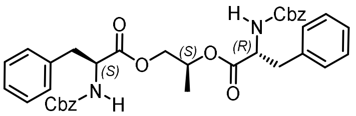

(*S,S,R*)-**25**

***R*f** = 0.17 (30% EtOAc in *n*-hexane), stained with $KMnO_4$;

^{1}H NMR (500 MHz, $CDCl_3$): δ = 7.39–7.20 (m, 16H), 7.18–7.08 (m, 4H), 5.48 (d, *J* = 8.4 Hz, 1H), 5.35 (d, *J* = 8.0 Hz, 1H), 5.14–4.99 (m, 5H), 4.67 (q, *J* = 7.0 Hz, 1H), 4.60 (q, *J* = 6.9 Hz, 1H), 4.25 (dd, *J* = 11.8, 3.4 Hz, 1H), 4.00 (dd, *J* = 11.8, 7.0 Hz, 1H), 3.18–3.10 (m, 2H), 3.05 (td, *J* = 13.0, 6.8 Hz, 2H), 1.14 (d, *J* = 6.6 Hz, 3H);

^{13}C NMR (126 MHz, $CDCl_3$): δ = 171.3, 171.2, 155.9, 155.8, 136.4, 136.3, 136.0, 135.8, 129.4, 129.3, 128.72, 128.70, 128.6, 128.3, 128.21, 128.17, 128.1, 127.3, 127.2, 69.3, 67.1, 67.0, 66.6, 55.0, 38.1, 38.0, 16.2;

HRMS (ESI$^+$): Calc. for $C_{37}H_{38}N_2O_8Na$ $[M+Na]^+$: 661.2520, found: 661.2494;

IR (thin film, cm^{-1}): 3300, 3030, 1704, 1518, 1497, 1205, 1052, 690;
$[\alpha]_D^{25}$= +2.19 (*c* = 0.82, $CHCl_3$);

HPLC: Daicel Chiralpak® IA column with guard, gradient 5% *i*-PrOH in *n*-hexane over 45 min; 5–18% *i*-PrOH in *n*-hexane over 20 min; 18% *i*-PrOH in *n*-hexane over 45 min; 1.0 mL/min, 40 °C, 220 nm, t_R = 85.92 min (*R,S,R* isomer), t_R = 88.68 min (*S,S,R* isomer), t_R = 90.35 min (*S,S,S* isomer), t_R = 96.68 min (*internal standard* (*R,R*)-**S22**), t_R = 99.39 min (*R,S,S* isomer).

Homochiral dimer ((*S,S,S*)-25)

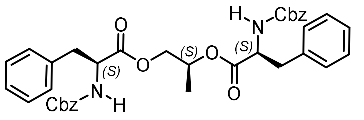

(*S,S,S*)-**25**

***R*f** = 0.15 (30% EtOAc in *n*-hexane), stained with $KMnO_4$;

^{1}H NMR (500 MHz, $CDCl_3$): δ = 7.38–7.21 (m, 16H), 7.13 (dd, *J* = 12.5, 6.9 Hz, 4H), 5.36 (d, *J* = 9.5 Hz, 1H), 5.33–5.27 (m., 1H), 5.17–5.01 (m, 5H), 4.65 (ddd, *J* = 15.0, 8.7, 6.3 Hz, 2H), 4.21 (dd, *J* = 11.8, 3.6 Hz, 1H), 4.00 (dd, *J* = 11.8, 6.8 Hz, 1H), 3.14 (dd, *J* = 14.0, 6.1 Hz, 2H), 3.09–3.00 (m, 2H), 1.20 (d, *J* = 6.6 Hz, 3H);

^{13}C NMR (126 MHz, $CDCl_3$): δ = 171.3, 171.0, 155.80, 155.77, 136.33, 136.29, 135.81, 135.77, 129.4, 129.3, 128.8, 128.7, 128.6, 128.3, 128.2, 127.3, 69.5, 67.07, 67.05, 66.7, 55.04, 55.01, 38.3, 38.1, 16.3;

HRMS (ESI$^+$): Calc. for $C_{37}H_{38}N_2O_8Na$ $[M+Na]^+$: 661.2520, found: 661.2514;

IR (thin film, cm^{-1}): 3303, 2954, 1745, 1514, 1455, 1254, 1053, 697;

$[\alpha]_D^{25}$= +27.6 (*c* = 1.04, $CHCl_3$);

HPLC: Daicel Chiralpak® IA column with guard, gradient 5% *i*-PrOH in *n*-hexane over 45 min; 5–18% *i*-PrOH in *n*-hexane over 20 min; 18% *i*-PrOH in *n*-hexane over 45 min; 1.0 mL/min, 40 °C, 220 nm, t_R = 85.92 min (*R,S,R* isomer), t_R = 88.68 min (*S,S,R* isomer), $\boldsymbol{t_R}$ = 90.35 min (*S,S,S* isomer), t_R = 96.68 min (*internal standard* (*R,R*)-**S22**), t_R = 99.39 min (*R,S,S* isomer).

Homochiral dimer ((*R,S,R*)-25)

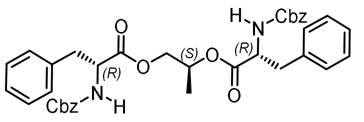

(*R,S,R*)-**25**

***R*f** = 0.17 (30% EtOAc in *n*-hexane), stained with $KMnO_4$;

MP: 94–98 °C (*n*-hexane/EtOAc);

^{1}H NMR (500 MHz, $CDCl_3$): δ = 7.39−7.20 (m, 16H), 7.18−7.08 (m, 4H), 5.48 (d, *J* = 8.4 Hz, 1H), 5.35 (d, *J* = 8.0 Hz, 1H), 5.14−4.99 (m, 5H), 4.67 (q, *J* = 7.0 Hz, 1H), 4.60 (q, *J* = 6.9 Hz,

1H), 4.25 (dd, J = 11.8, 3.4 Hz, 1H), 4.00 (dd, J = 11.8, 7.0 Hz, 1H), 3.18−3.10 (m, 2H), 3.05 (td, J = 13.0, 6.8 Hz, 2H), 1.14 (d, J = 6.6 Hz, 3H);

^{13}C NMR (126 MHz, $CDCl_3$): δ = 171.5, 171.2, 156.0, 155.9, 136.4, 136.3, 136.0, 135.8, 129.44, 129.38, 128.76, 128.74, 128.6, 128.27, 128.24, 128.20, 127.3, 127.2, 69.3, 67.12, 67.08, 66.7, 55.1, 38.2, 38.0, 16.2;

HRMS (ESI$^+$): Calc. for $C_{37}H_{38}N_2O_8Na$ $[M+Na]^+$: 661.2520, found: 661.2494;

IR (thin film, cm^{-1}): 3330, 2966, 1748, 1734, 1696, 1534, 1346, 1257, 1211, 1046, 745, 690;

$[\boldsymbol{\alpha}]_{\mathbf{D}}^{\mathbf{25}}$= −8.80 ($c$ = 1.35, $CHCl_3$);

HPLC: Daicel Chiralpak® IA column with guard, gradient 5% i-PrOH in n-hexane over 45 min; 5–18% i-PrOH in n-hexane over 20 min; 18% i-PrOH in n-hexane over 45 min; 1.0 mL/min, 40 °C, 220 nm, t_R = 85.92 min (*R,S,R* isomer), t_R = 88.68 min (*S,S,R* isomer), t_R = 90.35 min (*S,S,S* isomer), t_R = 96.68 min (*internal standard* (*R,R*)-**S22**), t_R = 99.39 min (*R,S,S* isomer).

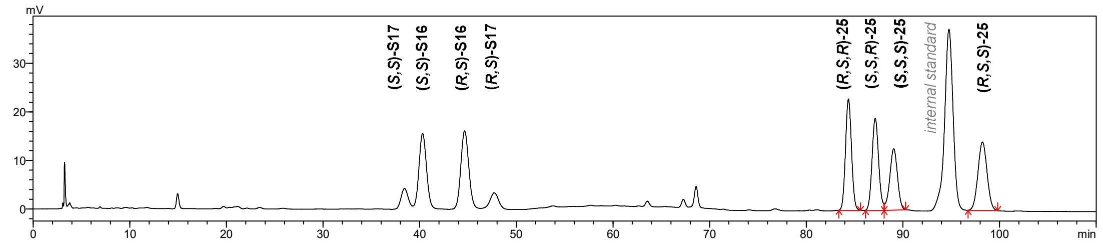

Peak#	Ret. Time	Area	Height	Conc.	Area%
1	84.364	1003161	22981	28.241	28.241
2	87.137	910798	19058	25.640	25.640
3	89.055	707137	12670	19.907	19.907
4	98.234	931091	14137	26.212	26.212
Total		3552186	68845	100.000	100.000

HPLC chromatogram of the crude reaction mixture (0.2 mmol scale)

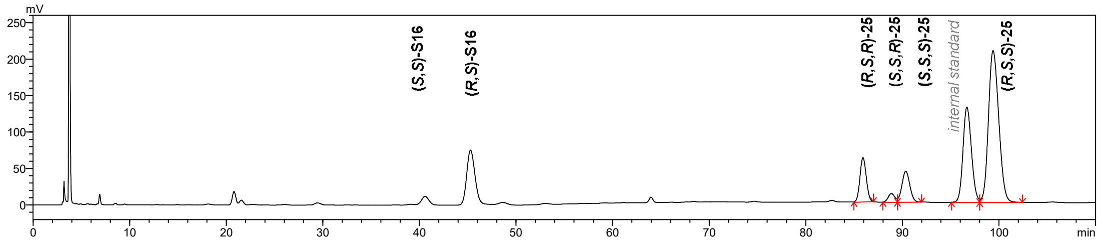

Peak#	Ret. Time	Area	Height	Conc.	Area%
1	85.921	2786157	60815	9.503	9.503
2	88.867	584386	11992	1.993	1.993
3	90.353	2426565	42517	8.276	8.276
4	96.680	8015925	130995	27.340	27.340
5	99.391	15506357	208429	52.888	52.888
Total		29319390	454748	100.000	100.000

Peak#	Ret. Time	Area A	Area/Area^{IS}	Corr. A/A^{IS}	d.r.
1	85.21	2786157	0.347577	0.331121	4.7
2	88.867	584386	0.072903	0.069965	1.0
3	90.353	2426565	0.302718	0.296492	4.2
4	96.680	8015925	-	-	
5	99.391	15506357	1.934443	1.926929	27.5

Note: Corrected A/A^{IS} were calculated using the correction factors obtained from the calibration curve for the homo- and heterodimer: $k^{R,S,S} = 1.0086$; $k^{S,S,R} = 1.0420$; $k^{S,S,S} = 1.0210$; $k^{R,S,R} = 1.0497$. (See full details in **Section 5.6**)

HPLC chromatogram of the purified reaction mixture (1.0 mmol scale)

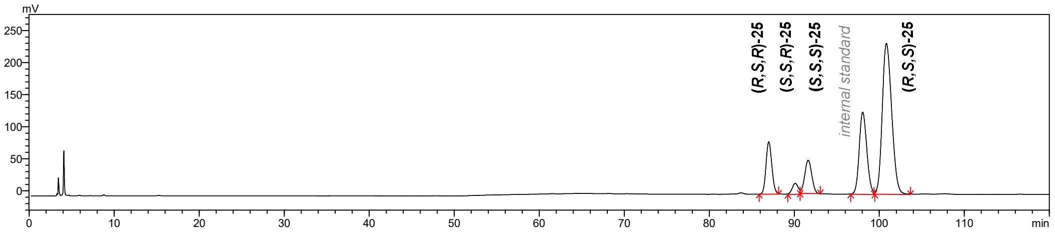

Peak#	Ret. Time	Area	Height	Conc.	Area%
1	86.756	3905735	81555	11.500	11.500
2	89.873	837978	17099	2.467	2.467
3	91.396	3005112	51923	8.848	8.848
4	97.810	8006421	128197	23.574	23.574
5	100.595	18208060	235173	53.611	53.611
Total		33963306	513948	100.000	100.000

Peak#	Ret. Time	Area A	Area/Area^{IS}	Corr. A/A^{IS}	d.r.
1	86.756	3905735	0.487825	0.464728	4.6
2	89.873	837978	0.104663	0.100444	1.0
3	91.396	3005112	0.375338	0.367618	3.7
4	97.810	8006421	-	-	
5	100.595	18208060	2.274182	2.265347	22.6

HPLC chromatogram of the purified reaction mixture by PTLC (0.2 mmol scale)

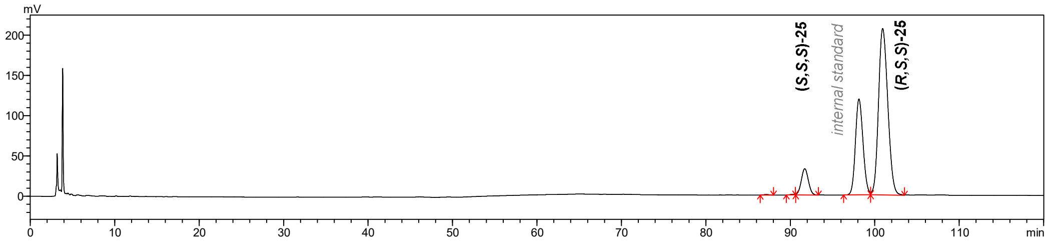

Peak#	Ret. Time	Area	Height	Conc.	Area%
1	87.160	36642	817	0.145	0.145
2	90.596	30158	772	0.119	0.119
3	91.712	1923714	32702	7.615	7.615
4	98.128	7435692	119120	29.434	29.434
5	100.938	15835778	206644	62.686	62.686
Total		25261985	360055	100.000	100.000

Peak#	Ret. Time	Area A	Area/Area^{IS}	Corr. A/A^{IS}	d.r.
1	87.16	36642	0.004928	0.004695	1.2
2	90.596	30158	0.004056	0.003892	1.0
3	91.712	1923714	0.258714	0.253392	65.1
4	98.128	7435692	-	-	-
5	100.938	15835778	2.129698	2.12142	545.1

5.4. Preparation of Authentic Samples

TBS-protected diol (S20)

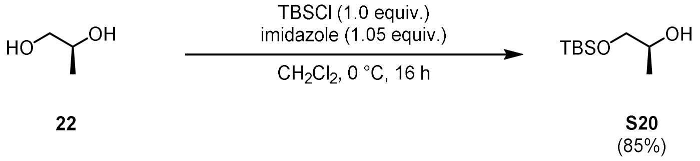

Based on the procedure reported by Harran and co-workers.[61] (*S*)-1,2-propanediol **22** (1.00 g, 13.1 mmol, 1.00 equiv.) was dissolved in CH_2Cl_2 (100 mL). Imidazole (940 mg, 13.8 mmol, 1.05 equiv.) was added and the reaction mixture was cooled down to 0 °C. TBSCl (1.97 g, 13.1 mmol, 1.00 equiv.) was added at 0 °C and the reaction was brought back to room temperature and stirred for 16 h. Following this, the reaction mixture was filtered through cotton and concentrated under reduced pressure. The crude was purified by silica plug (10% EtOAc in *n*-hexane) to give the protected diol (2.11 g, 11.1 mmol, 85%) as a colourless liquid.

Analytical data were consistent with those reported in the literature.[61]

^{1}H NMR (400 MHz, $CDCl_3$): δ = 3.82 (dqd, *J* = 7.7, 6.3, 3.3 Hz, 1H), 3.59 (dd, *J* = 9.8, 3.4 Hz, 1H), 3.35 (dd, *J* = 9.9, 7.8 Hz, 1H), 1.12 (d, *J* = 6.3 Hz, 3H), 0.91 (s, 9H), 0.08 (s, 6H);

^{13}C NMR (101 MHz, $CDCl_3$): δ = 68.7, 68.1, 26.0, 18.5, 18.4, −5.2, −5.3.

N-Cbz-L-phenylalanine or *N*-Cbz-D-phenylalanine (S18)

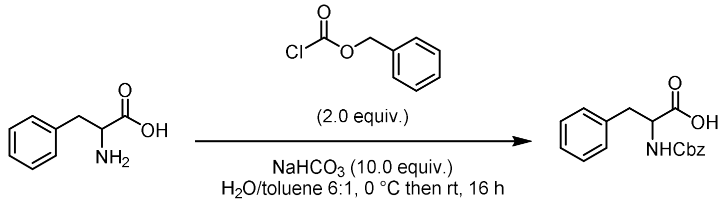

S18

Based on the procedure reported by Schoenebeck and co-workers.[62] D- or L-phenylalanine (1.65 g, 10.0 mmol, 1.00 equiv.) was suspended in water (40 mL) at 0 °C followed by the addition of sodium bicarbonate (8.40 g, 100 mmol, 10 equiv.). A solution of benzyl chloroformate (2.85 mL, 20.0 mmol, 2.0 equv.) in toluene (7 mL) was added in a dropwise manner at 0 °C. After addition, the reaction mixture was warmed to room temperature and stirred overnight. Following this, the phases were separated and the aqueous phase was washed with Et_2O (4 × 20 mL). The aqueous phase was then acidified with HCl 1 M (*ca.* 95 mL) to pH = 1 and extracted with ethyl acetate (3 × 50 mL). The combined organic phases were then dried over Na_2SO_4, filtered, and concentrated under reduced pressure to give a thick colourless oil (*R*-enantiomer, 2.35 g, 7.9 mmol, 79% yield; *S*-enantiomer, 1.70 g, 5.7 mmol, 57% yield) after drying under high vacuum.

Analytical data were consistent with those reported in the literature.[63]

^{1}H NMR (600 MHz, $CDCl_3$): δ = 7.39–7.23 (m, 8H), 7.17–7.13 (m, 2H), 5.17 (d, *J* = 7.7 Hz, 1H), 5.14–5.06 (m, 2H), 4.71 (dt, *J* = 8.4, 5.9 Hz, 1H), 3.21 (dd, *J* = 14.0, 5.5 Hz, 1H), 3.12 (dd, *J* = 14.2, 6.2 Hz, 1H);

^{13}C NMR (126 MHz, $CDCl_3$): δ = 176.4, 156.0, 136.2, 135.6, 129.5, 128.8, 128.7, 128.4, 128.3, 127.4, 67.3, 54.7, 37.9.

TBS-protected alcohol ((*S,S*)-S21 or (*R,R*)-S21)

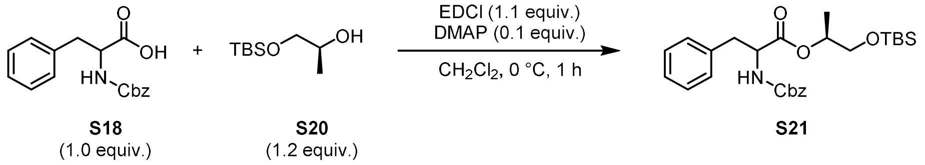

D or L- *N*-benzyloxycarbonyl-phenylalanine **S18** (180 mg, 0.60 mmol, 1.00 equiv.) was dissolved in CH_2Cl_2 (6 mL) at 0 °C. DMAP (7.30 mg, 0.06 mmol, 0.10 equiv.) and EDCl (102 mg, 0.66 mmol, 1.10 equiv.) were then added followed by a solution of the alcohol **S20** (137 mg, 0.72 mmol, 1.20 equiv.) in CH_2Cl_2 (2 mL) at 0 °C. The reaction mixture was left to stir for 1 h at this temperature. Following this, the reaction mixture was washed with saturated aqueous $NaHCO_3$ (10 mL), the organic phase was collected and further washed with brine (15 mL), dried over Na_2SO_4, filtered and concentrated under reduced pressure. The crude residue was purified by column chromatography on silica gel (10–20% EtOAc in *n*-hexane) to give the desired ester **S21** (*S*-enantiomer, 200 mg, 0.43 mmol, 71%; *R*-enantiomer, 200 mg, 0.43 mmol, 71%) as a colourless oil.

TBS-protected alcohol ((*S,S*)-21)

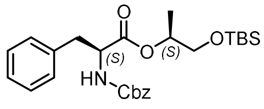

(*S,S*)-**S21**

$\boldsymbol{R_f}$ = 0.26 (5% EtOAc in *n*-hexane), stained with $KMnO_4$;

^{1}H NMR (500 MHz, $CDCl_3$): δ = 7.38–7.30 (m, 5H), 7.29–7.21 (m, 3H), 7.16–7.13 (m, 2H), 5.22 (d, *J* = 8.4 Hz, 1H), 5.09 (q, *J* = 12.3 Hz, 2H), 4.95 (td, *J* = 6.2, 4.7 Hz, 1H), 4.65 (dt, *J* = 8.4, 5.8 Hz, 1H), 3.61 (dd, *J* = 10.8, 5.9 Hz, 1H), 3.55 (dd, *J* = 10.7, 4.7 Hz, 1H), 3.16 (dd, *J* = 13.9, 5.8 Hz, 1H), 3.08 (dd, *J* = 13.9, 6.0 Hz, 1H), 1.21 (d, *J* = 6.5 Hz, 3H), 0.90 (s, 9H), 0.07 (d, *J* = 4.1 Hz, 6H);

^{13}C NMR (126 MHz, $CDCl_3$): δ = 171.2, 155.7, 136.5, 135.9, 129.6, 128.63, 128.61, 128.3, 128.2, 127.2, 73.1, 67.0, 65.5, 55.0, 38.4, 26.0, 18.4, 16.4, −5.25, −5.27;

HRMS (ESI$^+$): Calc. for $C_{26}H_{37}NO_5Na$ $[M+Na]^+$: 494.2333, found: 494.2333;

IR (thin film, cm^{-1}): 2954, 1724, 1506, 1251, 837, 697;

$[\alpha]_D^{20}$= +14.0 (*c* = 1.20, $CHCl_3$).

TBS-protected alcohol ((*R*,*S*)-S21)

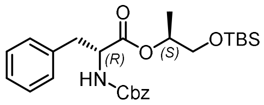

(*R*,*S*)-**S21**

***R*f** = 0.29 (5% EtOAc in *n*-hexane), stained with $KMnO_4$;

^{1}H NMR (500 MHz, $CDCl_3$): δ = 7.39–7.29 (m, 5H), 7.29–7.21 (m, 3H), 7.17–7.09 (m, 2H), 5.27 (d, *J* = 8.2 Hz, 1H), 5.10 (d, *J* = 2.2 Hz, 2H), 4.95 (h, *J* = 6.3 Hz, 1H), 4.64 (dt, *J* = 8.2, 6.0 Hz, 1H), 3.61 (dd, *J* = 10.7, 5.5 Hz, 1H), 3.54 (dd, *J* = 10.8, 5.0 Hz, 1H), 3.12 (qd, *J* = 13.9, 5.9 Hz, 2H), 1.18 (d, *J* = 6.5 Hz, 3H), 0.89 (s, 9H), 0.05 (br s, 6H);

^{13}C NMR (126 MHz, $CDCl_3$): δ = 171.1, 155.6, 136.5, 135.9, 129.6, 128.63, 128.61, 128.3, 128.2, 127.2, 72.9, 67.0, 65.4, 55.0, 38.5, 25.9, 18.4, 16.3, −5.2, −5.3;

HRMS (ESI$^+$): Calc. for $C_{26}H_{37}NO_5Na$ $[M+Na]^+$: 494.2333, found: 494.2334;

IR (thin film, cm^{-1}): 2954, 1717, 1497, 1251, 1204, 776, 697;

$[\alpha]_D^{20}$= −20.9 (*c* = 1.33, $CHCl_3$);

Primary alcohol ((*R*,*S*)-S17 or (*S*,*S*)-S17)

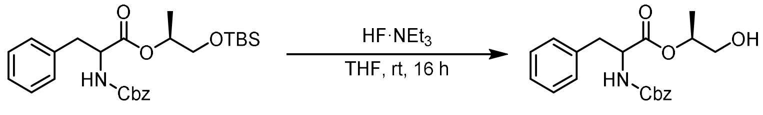

S21 **S17**

Compound **S21** (400 mg, 0.84 mmol, 1.00 equiv.) was dissolved in THF (10 mL) at 0 °C. NEt_3·HF (1 mL) was added slowly over 2 min. The clear solution was stirred at 0 °C for 10 min and then at room temperature for 3 h. Following this, the reacton was diluted with ethyl acetate (30 mL) and a solution of saturated $CaCO_3$ was added (40 mL). The biphasic mixture was vigorously stirred for 10 min and phases were then separated. The organic phase was washed with a saturated aqueous $NaHCO_3$ (30 mL), then with brine (20 mL), dried over Na_2SO_4, filtered and concentrated under reduced pressure to give a crude desired primary alcohol (*S*-enantiomer, 285 mg, 0.79 mmol, 94% yield; *R*-enantiomer, 287 mg, 0.80 mmol, 96% yield) as a colourless oil. The crude oil was used directly in the next step as the product undergoes intramolecular [1,4]-acyl migration on silica gel.

Primary alcohol ((*R*,*S*)-S17)

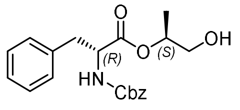

(*R*,*S*)-**S17**

$\boldsymbol{R_f}$ = 0.54 (50% EtOAc in *n*-hexane), stained with $KMnO_4$;

^{1}H NMR (500 MHz, $CDCl_3$): δ = 7.40–7.21 (m, 8H), 7.17–7.12 (m, 2H), 5.25 (d, *J* = 6.3 Hz, 1H), 5.09 (d, *J* = 4.1 Hz, 2H), 5.01 (tt, *J* = 9.9, 5.0 Hz, 1H), 4.55 (dt, *J* = 7.6, 6.5 Hz, 1H), 3.63 (dd, *J* = 12.3, 3.3 Hz, 1H), 3.52 (dd, *J* = 12.3, 6.6 Hz, 1H), 3.16–3.04 (m, 2H), 1.13 (d, *J* = 6.6 Hz, 3H);

^{13}C NMR (126 MHz, $CDCl_3$): δ = 171.7, 156.0, 136.2, 135.8, 129.5, 128.8, 128.7, 128.4, 128.3, 127.4, 73.5, 67.3, 65.6, 55.5, 38.2, 16.0;

HRMS (ESI^{+}): Calc. for $C_{20}H_{23}NO_5Na$ $[M+Na]^{+}$: 380.1468, found: 380.1466;

IR (thin film, cm^{-1}): 3405, 2980, 1705, 1526, 1497, 1259, 1213, 1056, 699;

$[\alpha]_D^{20}$ = −12.4 (*c* = 1.80, $CHCl_3$);

HPLC: Daicel Chiralpak® IA column with guard, gradient 5% *i*-PrOH in *n*-hexane over 45 min; 5–18% *i*-PrOH in *n*-hexane over 20 min; 18% *i*-PrOH in *n*-hexane over 45 min; 1.0 mL/min, 40 °C, 220 nm, t_R = 48.90 min (*R,S* isomer).

Primary alcohol ((*S,S*)-S17)

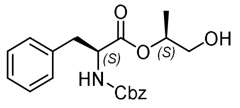

(*S,S*)-**S17**

***R*f** = 0.51 (50% EtOAc in *n*-hexane), stained with $KMnO_4$;

^{1}H NMR (500 MHz, $CDCl_3$): δ = 7.39–7.23 (m, 8H), 7.21–7.16 (m, 2H), 5.23 (d, *J* = 7.9 Hz, 1H), 5.10 (s, 2H), 4.99 (td, *J* = 6.6, 3.1 Hz, 1H), 4.60 (q, *J* = 6.7 Hz, 1H), 3.57–3.48 (m, 1H), 3.41 (dd, *J* = 12.3, 6.9 Hz, 1H), 3.15 (dd, *J* = 14.0, 6.4 Hz, 1H), 3.08 (dd, *J* = 13.9, 6.8 Hz, 1H), 1.76 (s, 1H), 1.22–1.09 (m, 3H).

^{13}C NMR (126 MHz, $CDCl_3$): δ = 171.5, 156.1, 136.2, 135.9, 129.4, 128.9, 128.7, 128.4, 128.30, 127.5, 73.7, 67.3, 65.5, 55.3, 38.4, 16.1;

HRMS (ESI$^+$): Calc. for $C_{20}H_{23}NO_5Na$ $[M+Na]^+$: 380.1468, found: 380.1466;

IR (thin film, cm^{-1}): 3405, 2934, 1739, 1514, 1497, 1257, 1213, 1090, 697;

$[\alpha]_D^{20}$= +17.7 (*c* = 1.65, $CHCl_3$);

HPLC: Daicel Chiralpak® IA column with guard, gradient 5% *i*-PrOH in *n*-hexane over 45 min; 5–18% *i*-PrOH in *n*-hexane over 20 min; 18% *i*-PrOH in *n*-hexane over 45 min; 1.0 mL/min, 40 °C, 220 nm, t_R = 39.18 min (*S,S* isomer).

Preparation of Authentic Samples of Hetero and Homodimers

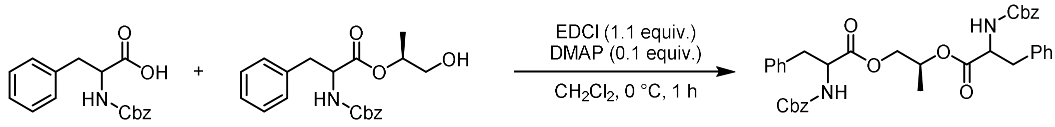

(*R*)-**S18** or (*S*)-**S17** (1.0 equiv.) + (*R,S*)-**S17** or (*S,S*)-**S17** (1.0 equiv.) → **25** (68–76%)

Heterodimers:

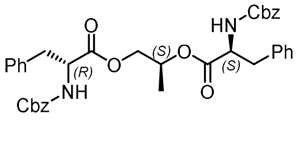

(*R,S,S*)-**25**
(68%)
from (*R*)-**S18** and (*S,S*)-**S17**

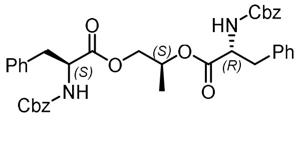

(*S,S,R*)-**25**
(71%)
from (*S*)-**S18** and (*R,S*)-**S17**

Homodimers:

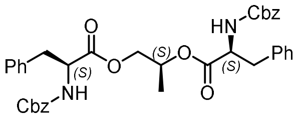

(*S,S,S*)-**25**
(76%)
from (*S*)-**S18** and (*S,S*)-**S17**

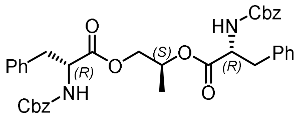

(*R,S,R*)-**25**
(70%)
from (*R*)-**S18** and (*R,S*)-**S17**

D- or L-*N*-benzyloxycarbonyl-phenylalanine **S18** (89.8 mg, 0.30 mmol, 1.00 equiv.) was dissolved in CH_2Cl_2 (3 mL) at 0 °C. DMAP (3.7 mg, 0.03 mmol, 0.10 equiv.) and EDCl (63.5 mg, 0.33 mmol, 1.10 equiv.) were then added followed by a solution of the (*S,S*)- or (*R,S*)-primary alcohol **S17** (107 mg, 0.30 mmol, 1.20 equiv.) in CH_2Cl_2 (1 mL) at 0 °C. The reaction mixture was left to stir for 1 h at this temperature. Following this, the reaction mixture was washed with saturated aqueous $NaHCO_3$ (10 mL), the organic phase was collected and further washed with brine (15 mL), dried over Na_2SO_4, filtered and concentrated under reduced pressure. The crude residue was purified by column chromatography on silica gel (20–30% EtOAc in *n*-hexane) to give the desired hetero or homodimer **25** (130–146 mg, 0.20–0.23 mmol, 68–76%) as a colourless oil.

HPLC Internal standard ((*R,R*)-S22)

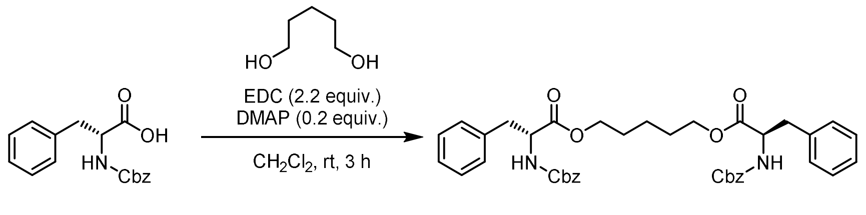

(*R*)-**S18**

(*R,R*)-**S22**
(91%)

D-*N*-benzyloxycarbonyl-phenylalanine (*R*)-**S18** (450 mg, 1.50 mmol, 2.0 equiv.) was dissolved in CH_2Cl_2 (15 mL) then cooled to 0 °C. DMAP (18.0 mg, 0.15 mmol, 0.20 equiv.) and EDC (316 mg, 1.65 mmol, 2.20 equiv.) were then added followed by a solution of pentan-1,5-diol (78.0 mg, 0.75 mmol, 1.00 equiv.) in CH_2Cl_2 (3.00 mL) at 0 °C. The reaction was then brought to rt and stirred for 3 h at this temperature. Following this, the reaction mixture was washed with saturated aqueous $NaHCO_3$ (20 mL), the organic phase was collected and further washed with brine (20 mL), dried over anhydrous Na_2SO_4, filtered, and concentrated under reduced pressure. The crude residue was purified by column chromatography on silica gel (30% EtOAc in *n*-hexane) to give the desired diester (*R,R*)-**S22** (455 mg, 0.68 mmol, 91% yield) as a colourless oil.

$\boldsymbol{R_f}$ = 0.63 (50% EtOAc in *n*-hexane), stained with $KMnO_4$;

^{1}H NMR (500 MHz, $CDCl_3$): δ = 7.37–7.21 (m, 16H), 7.14–7.09 (m, 4H), 5.30–5.24 (m, 2H), 5.16–5.03 (m, 4H), 4.65 (dt, *J* = 8.4, 6.1 Hz, 2H), 4.15–4.01 (m, 4H), 3.16–3.02 (m, 4H), 1.57 (p, *J* = 6.9 Hz, 4H), 1.25 (tdd, *J* = 15.8, 8.6, 6.0 Hz, 2H).

^{13}C NMR (126 MHz, $CDCl_3$): δ = 171.7, 155.8, 136.4, 135.9, 129.4, 128.7, 128.6, 128.3, 128.2, 127.2, 67.1, 65.2, 55.0, 38.5, 28.1, 22.3;

HRMS (ESI$^+$): Calc. for $C_{39}H_{42}N_2O_8Na$ $[M+Na]^+$: 689.2833, found: 689.2816;

IR (thin film, cm^{-1}): 3063, 1712, 1514, 1253, 1204, 1052, 739, 697;

$[\boldsymbol{\alpha}]_{\mathbf{D}}^{\mathbf{20}}$ = −41.6 (*c* = 0.50, $CHCl_3$);

HPLC: Daicel Chiralpak® IA column with guard, gradient 5% *i*-PrOH in *n*-hexane over 45 min; 5–18% *i*-PrOH in *n*-hexane over 20 min; 18% *i*-PrOH in *n*-hexane over 45 min; 1.0 mL/min, 40 °C, 220 nm, t_R = 96.68 min (*internal standard* (*R,R*)-**S22**).

5.5 HPLC Traces Assignment

Assignment for the monoadduct intermediate

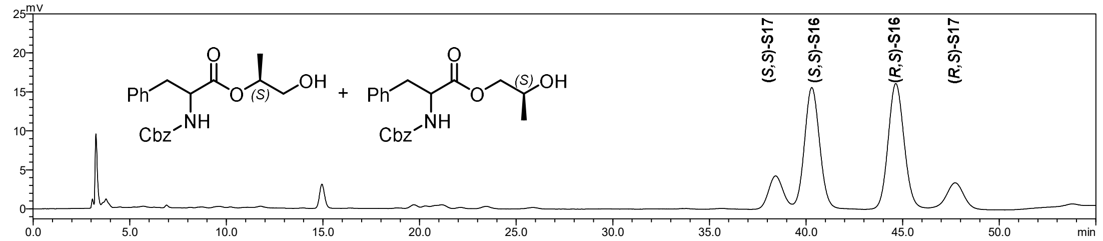

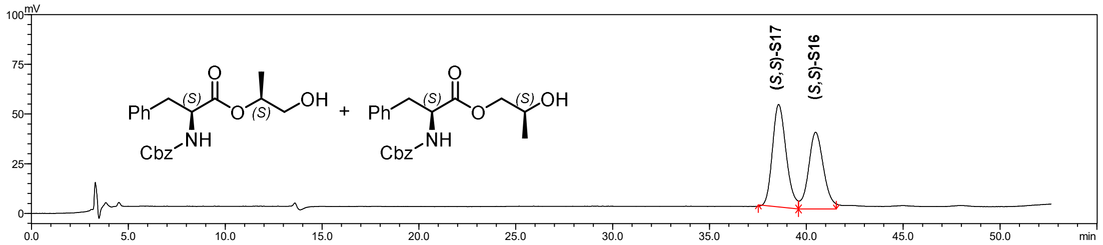

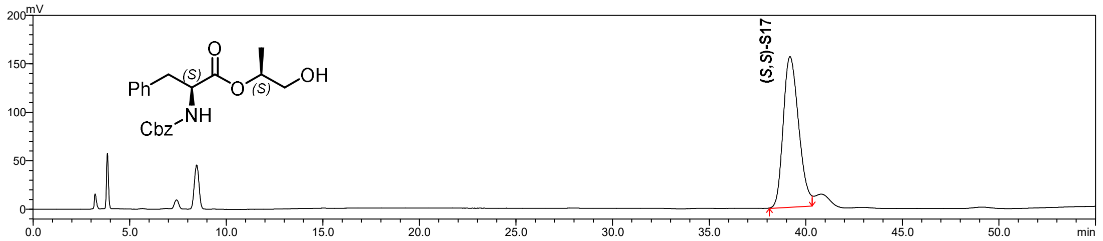

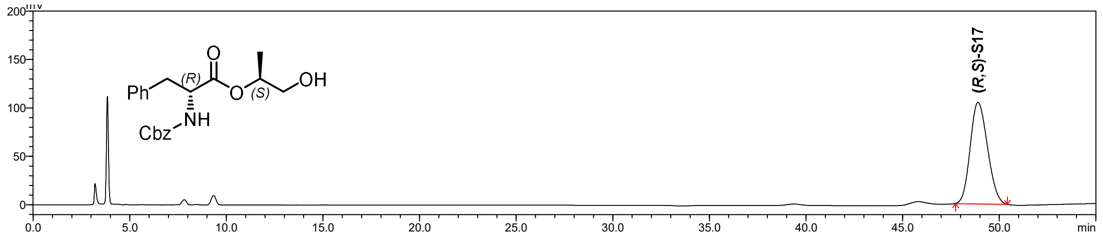

Assignment of the homo and heterodimeric mixture

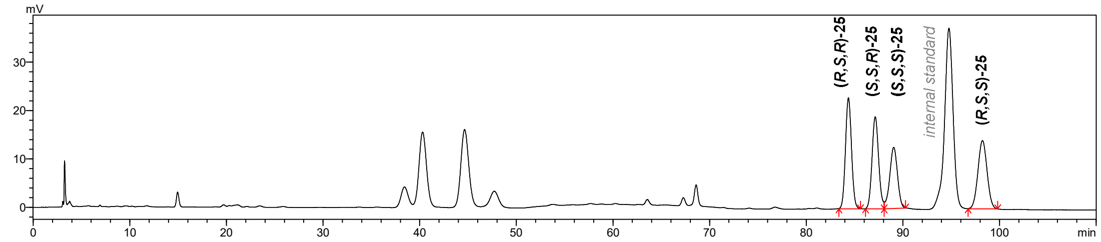

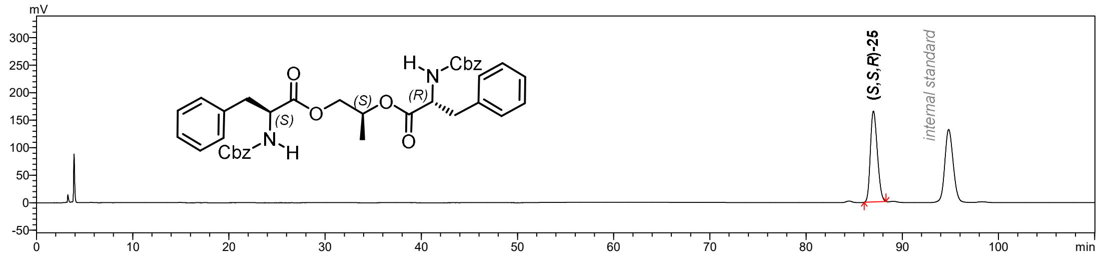

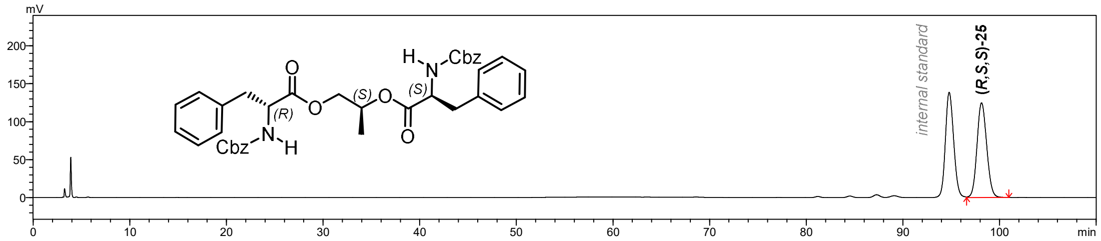

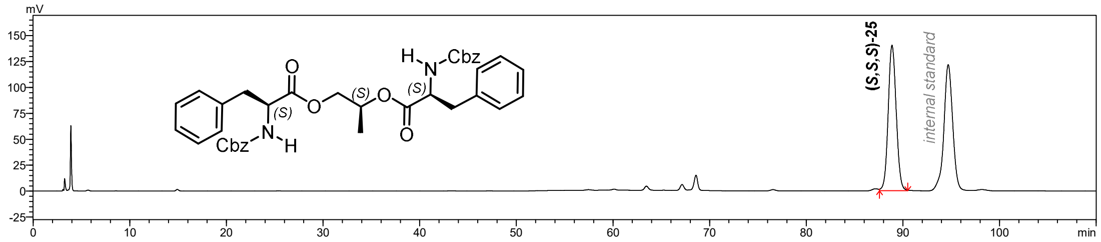

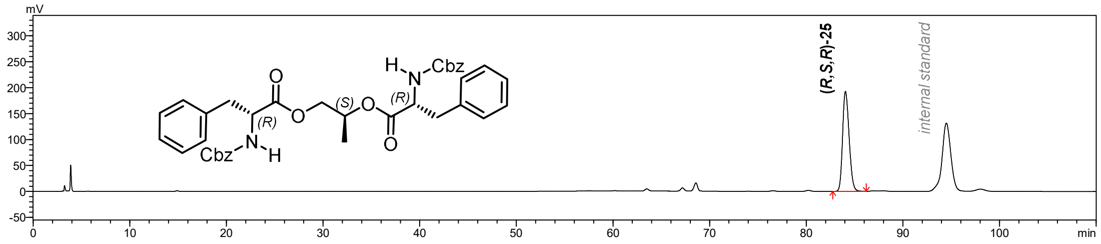

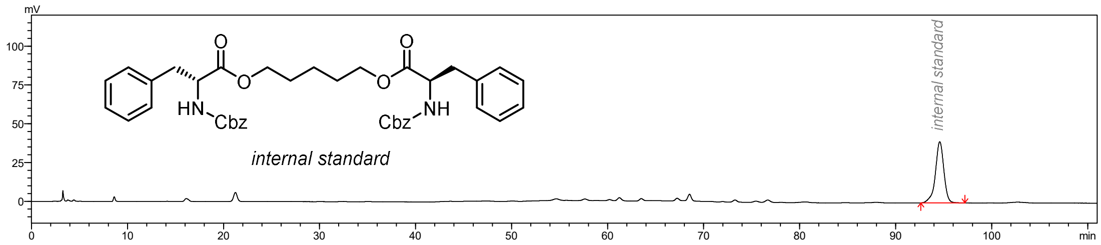

5.6. Calibration

For this reaction, as products are now diastereomeric to one another, direct integrations of the chromatogram would be inaccurate. To circumvent this issue, HPLC integrations needed calibration to allow the accurate measurement of the diastereomeric ratios. Therefore, authentic samples of each dimers were used to build a calibration curve $Area/Area^{IS} = k \times c$, where k is the correction factor of the dimer. These correction factors were then used to generate a corrected table from which the diastereomeric ratios were determined.

Calibration curve for the (*R,S,S*)-heterodimer

Solutions of heterodimer (*R,S,S*)-**25** in *i*-PrOH (2 mL) using diester (*R,R*)-**S22** (2.0 mg) as internal standard were prepared from stock solutions (4.0 mg/mL). Solutions were analysed by chiral stationary phase HPLC (Daicel Chiralpak® IA column with guard, gradient 5% *i*-PrOH in *n*-hexane over 45 min; 5–18% *i*-PrOH in *n*-hexane over 20 min; 18% *i*-PrOH in *n*-hexane over 45 min; 1.0 mL/min, 40 °C, 220 nm).

(*R,S,S*)-heterodimer t_R= 99.39 min; diester (*R,R*)-**S22** (IS) t_R= 96.68 min.

Hetero (*R,S,S*) (mg/mL)	IS (mg/mL)	Area Hetero (*R,S,S*)	Area IS	Area (R,S,S) /Area IS
0.04	2.0	341662	7840510	0.043576502
0.04	2.0	336075	7980305	0.04211305
0.04	2.0	323284	7823675	0.04132125
0.5	2.0	4273962	7948694	0.537693614
0.5	2.0	4247009	7889487	0.53831244
0.5	2.0	4242184	7643197	0.555027432
1.0	2.0	8545315	8105259	1.054292651
1.0	2.0	8233085	7892585	1.043141759
1.0	2.0	8442349	7909351	1.067388336
2.0	2.0	15793211	7723400	2.044852138
2.0	2.0	15522514	7769473	1.997885056
2.0	2.0	15777919	7854122	2.008871138

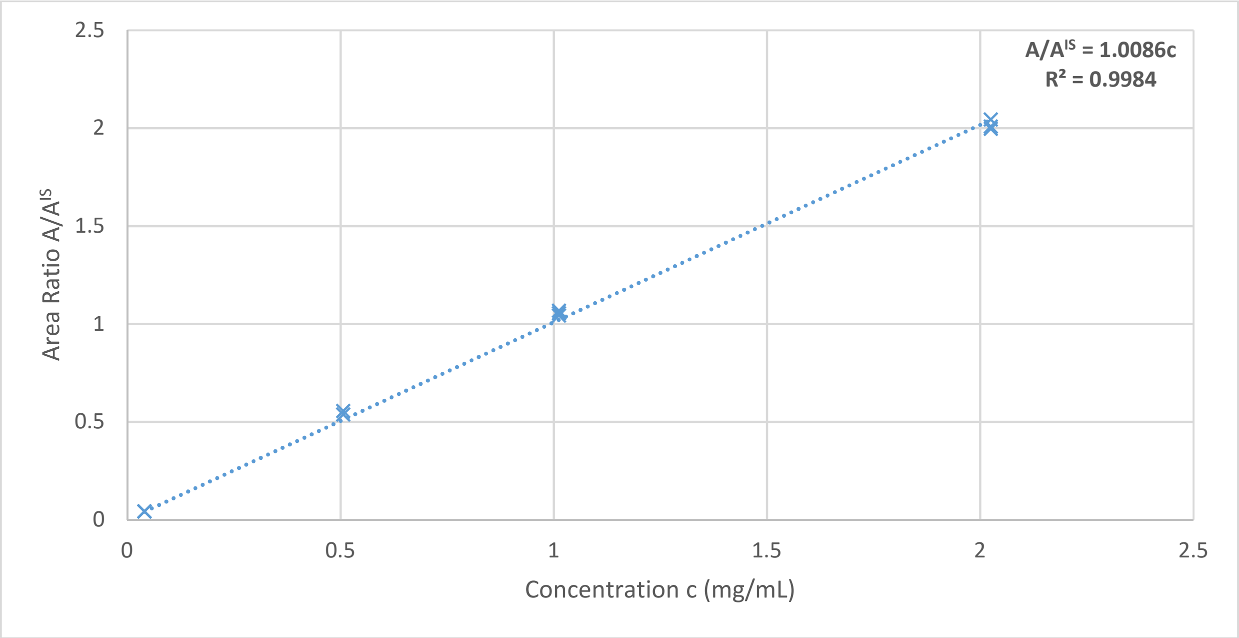

Correction factor $k^{R,S,S}$= 1.0086

Calibration curve for the (*S,S,R*)-heterodimer

Solutions of heterodimer (*S,S,R*)-**25** in *i*-PrOH (2 mL) using diester (*R,R*)-**S22** (2.0 mg) as internal standard were prepared from stock solutions (4.0 mg/mL). Solutions were analysed by chiral stationary phase HPLC (Daicel Chiralpak® IA column with guard, gradient 5% *i*-PrOH in *n*-hexane over 45 min; 5–18% *i*-PrOH in *n*-hexane over 20 min; 18% *i*-PrOH in *n*-hexane over 45 min; 1.0 mL/min, 40 °C, 220 nm).

(*S,S,R*)-heterodimer t_R= 88.68 min; diester (*R,R*)-**S22** (IS) t_R= 96.68 min.

Hetero (*S,S,R*) (mg/mL)	IS (mg/mL)	Area Hetero (*S,S,R*)	Area IS	Area (*S,S,R*) /Area IS
0.04	2.0	359799	7653920	0.047008461
0.04	2.0	369010	7746997	0.04763265
0.04	2.0	352554	7707058	0.045744303
0.5	2.0	4394713	7413266	0.59281739
0.5	2.0	4591468	7565956	0.606858935
0.5	2.0	4610443	7784969	0.592223681
1.0	2.0	8328671	7959517	1.046378945
1.0	2.0	8516348	7997964	1.064814495
1.0	2.0	8429541	7650063	1.10189171
2.0	2.0	16088014	7725205	2.082535544
2.0	2.0	16115133	7441863	2.165470259
2.0	2.0	16149721	7660406	2.108206928

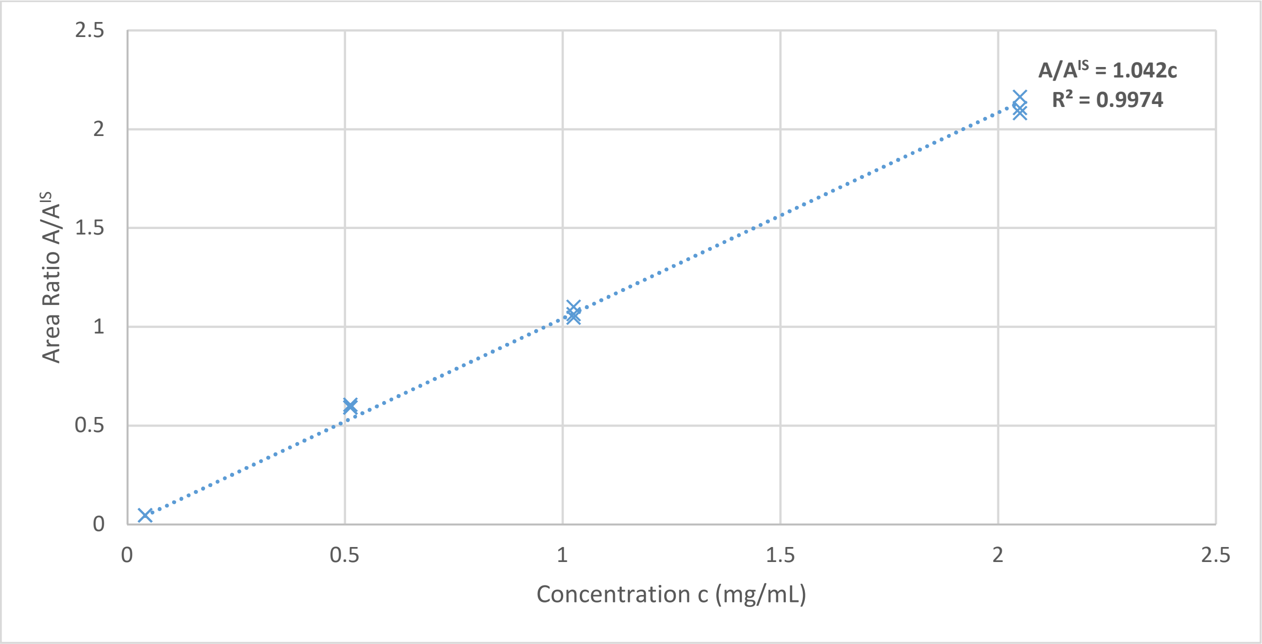

Correction factor $k^{S,S,R}$= 1.0420

Calibration curve for the (*S,S,S*)-homodimer

Solutions of homodimer (*S,S,S*)-**25** in *i*-PrOH (2 mL) using diester (*R,R*)-**S22** (2.0 mg) as internal standard were prepared from stock solutions (4.0 mg/mL). Solutions were analysed by chiral stationary phase HPLC (Daicel Chiralpak® IA column with guard, gradient 5% *i*-PrOH in *n*-hexane over 45 min; 5–18% *i*-PrOH in *n*-hexane over 20 min; 18% *i*-PrOH in *n*-hexane over 45 min; 1.0 mL/min, 40 °C, 220 nm).

(*S,S,S*)-homodimer t_R= 90.35 min; diester (*R,R*)-**S22** (IS) t_R= 96.68 min.

Homo (*S,S,S*) (mg/mL)	IS (mg/mL)	Area Homo (*S,S,S*)	Area IS	Area (S,S,S) /Area IS
0.04	2.0	383122	7679441	0.049889308
0.04	2.0	305449	7530144	0.040563501
0.04	2.0	361962	7762671	0.046628538
0.5	2.0	4077724	7768237	0.524922708
0.5	2.0	4183070	7745968	0.540031924
0.5	2.0	3959782	7518482	0.52667307
1.0	2.0	7852322	7663770	1.024603035
1.0	2.0	7918435	7861332	1.007263782
1.0	2.0	8153924	7881594	1.034552655
2.0	2.0	14783420	7260931	2.036022653
2.0	2.0	14733493	7430139	1.982936389
2.0	2.0	14774672	7137280	2.070070391

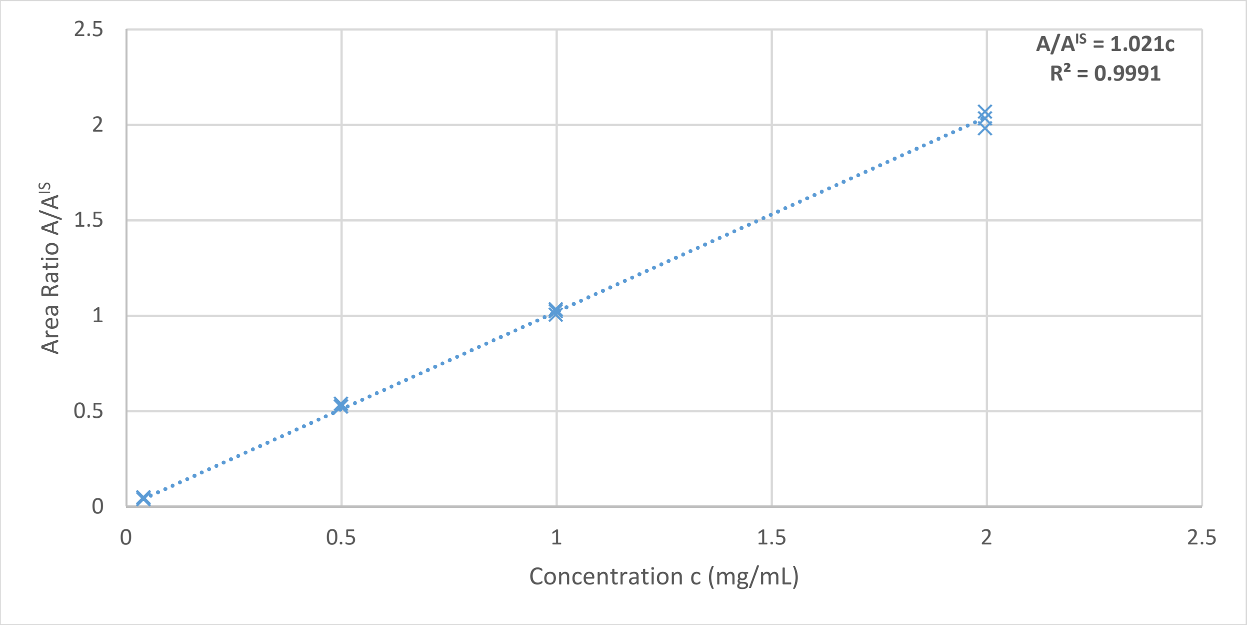

Correction factor $k^{S,S,S}$= 1.021

Calibration curve for the (*R,S,R*)-homodimer

Solutions of homodimer (*R,S,R*)-**25** in *i*-PrOH (2 mL) using diester (*R,R*)-**S22** (2.0 mg) as internal standard were prepared from stock solutions (4.0 mg/mL). Solutions were analysed by chiral stationary phase HPLC (Daicel Chiralpak® IA column with guard, gradient 5% *i*-PrOH in *n*-hexane over 45 min; 5–18% *i*-PrOH in *n*-hexane over 20 min; 18% *i*-PrOH in *n*-hexane over 45 min; 1.0 mL/min, 40 °C, 220 nm).

(*R,S,R*)-homodimer t_R= 85.92 min; diester (*R,R*)-**S22** (IS) t_R= 96.68 min.

Homo (*R,S,R*) (mg/mL)	IS (mg/mL)	Area Homo (*R,S,R*)	Area IS	Area (*R,S,R*) /Area IS
0.04	2.0	325222	7032289	0.046246962
0.04	2.0	335145	7560650	0.044327538
0.04	2.0	363812	7984506	0.045564748
0.5	2.0	4533388	7603791	0.596201027
0.5	2.0	4518121	7807025	0.578725059
0.5	2.0	4568779	7776274	0.587528037
1.0	2.0	9033411	8256267	1.09412777
1.0	2.0	8792333	7847248	1.120435215
1.0	2.0	9032095	8213084	1.099720276
2.0	2.0	16759541	7770558	2.156800194
2.0	2.0	16409611	7945996	2.065142117
2.0	2.0	16396794	7920119	2.070271166

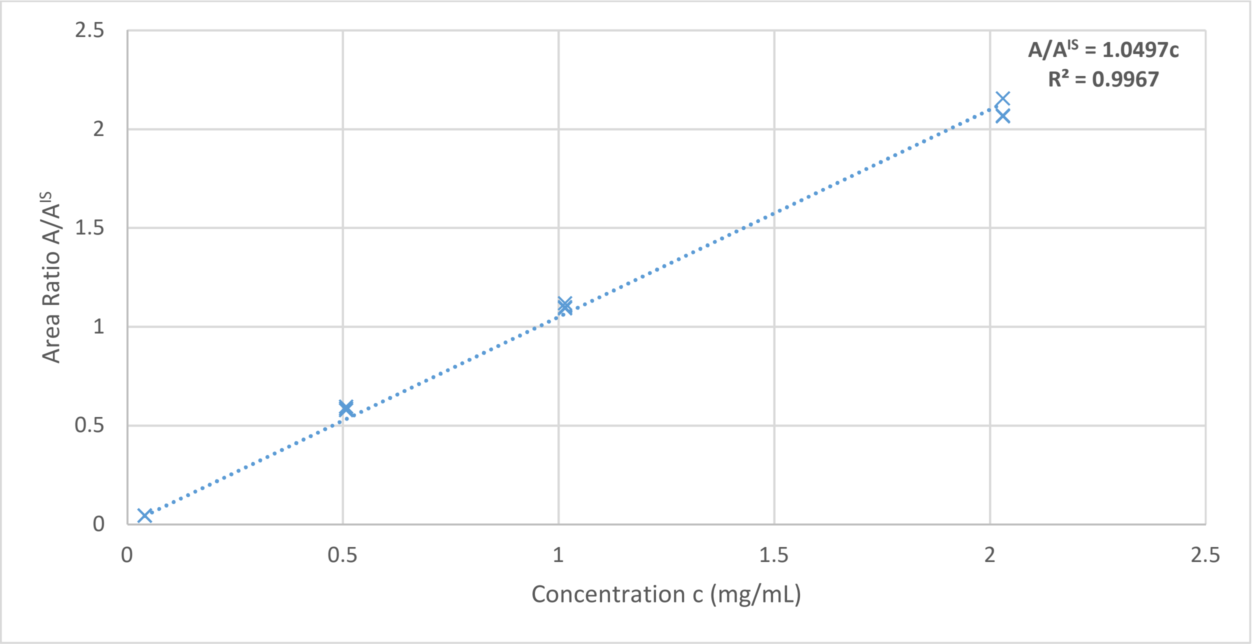

Correction factor $k^{R,S,R}$= 1.0497

5.7. Optimisation of the Stereoretentive Diasteroconvergent Reaction

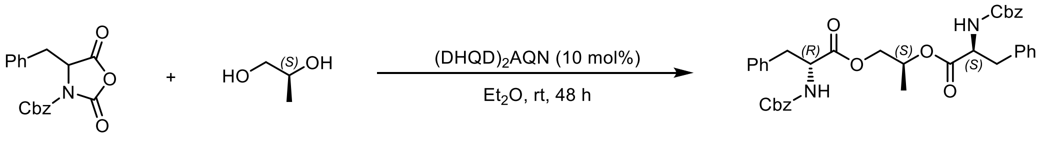

Table 4. Concentration and stoichiometry of **22** optimisation

Entry	Conc. (M)	22 (equiv.)	Conv. (%)[A]	Dimer (%)[B]	de	hetero:homo
1	0.015	0.55	100	31	80	2:1
2	0.05	0.55	100	39	84	2:1
3	0.025	0.55	100	39	84	2:1
4	0.025	0.5	100	*n.i*	78	1.8:1

Reactions conducted on 0.2 mmol scale. **(A)** Determined by ^{1}H NMR using 1,2-dimethoxyethane as internal standard; **(B)** Determined by HPLC using diester (*R,R*)-**S22** as an internal standard.

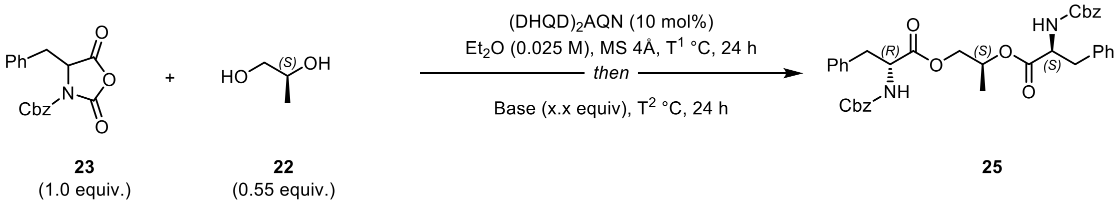

Table 5. Temperature and base screening

Entry	Base (equiv.)	T^1 (°C)	T^2 (°C)	Conv. (%)[A]	Dimer (%)[B]	de	hetero:homo
1	-	rt	rt	100	39	80	1.4:1
2	-	−20	−20	55	5	92	2:1
3	-	−20	rt	92	52	80	1.4:1
4	DIPEA (1.0)	−20	−20	58	15	92	2.1:1
5	DIPEA (1.0)	−20	rt	90	57	88	3.4:1
6	DIPEA (1.5)	−40	rt	92	53	90	2.6:1
7	DIPEA (2.5)	−40	rt	96	49	90	2.5:1
8	DIPEA (1.0)<br>DMAP (0.2)	−40	rt	100	83	92	2.7:1
9	DMAP (0.2)	−40	rt	100	86 (77)[C]	93	3.2:1
10[D]	DMAP (0.1)	−40	rt	100	76	84	1.9:1

Reactions conducted on 0.2 mmol scale. **(A)** Determined by ^{1}H NMR using 1,2-dimethoxyethane as internal standard; **(B)** Determined by HPLC using diester (*R*,*R*)-**S22** as an internal standard; **(C)** Isolated yield; **(D)** DMAP was introduced from the beginning of the reaction.

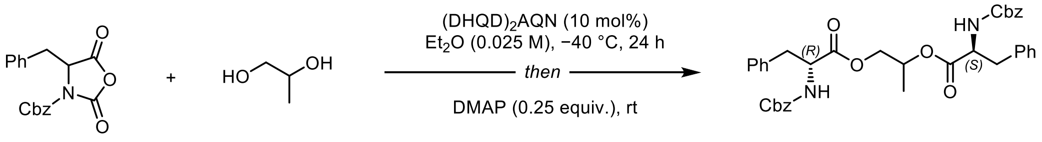

23
(1.0 equiv.)

(*R*)-22 or (*S*)-22
(0.55 equiv.)

25

Table 6. Study of the match/mismatch effect of the linker.

Entry	Enantiomer of **22**	Conv. (%)[A]	Dimer (%)[B]	de	hetero:homo
1	***S***	100	77	93	3.2:1
2	***R***	100	55	66	1.4:1

Reactions conducted on 0.2 mmol scale. **(A)** Determined by ^{1}H NMR using 1,2-dimethoxyethane as internal standard; **(B)** Determined by HPLC using diester (*R,R*)**-S22** as an internal standard.

Stereoretentive diastereoconvergent reaction using (*R*)-diol 22

(*For reaction using (S)-diol under the same conditions, see Section 5.3)*

HPLC: Daicel Chiralpak® ID column with guard, gradient 10% *i*-PrOH in *n*-hexane over 10 min; 10–20% *i*-PrOH in *n*-hexane over 10 min; 20% *i*-PrOH in *n*-hexane over 20 min; 20–50% *i*-PrOH in *n*-hexane over 20 min; 50% *i*-PrOH in *n*-hexane over 40 min; 1.0 mL/min, 30 °C, 220 nm, t_R = 69.95 min (*R,R,R* isomer), t_R = 72.46 min (*S,R,S* isomer), t_R = 75.82 min (*R,R,S* isomer), t_R = 79.96 min (*S,R,R* isomer), t_R = 104.94 (*internal standard*), 66% de, hetero:homo 1.4:1.

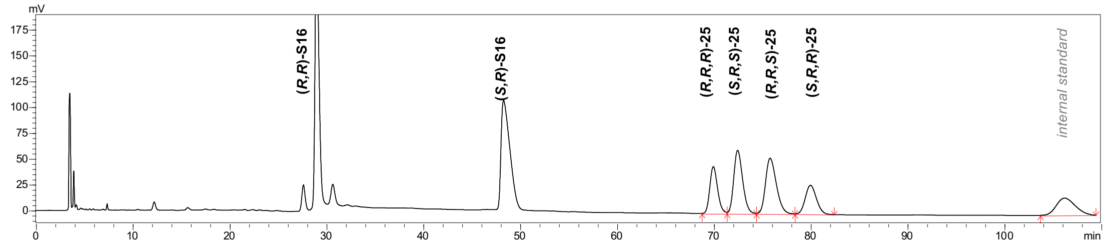

Peak#	Ret. Time	Area	Height	Conc.	Area%
1	69.951	2553947	45446	16.828	16.828
2	72.463	3946768	61350	26.005	26.005
3	75.819	4172774	53823	27.494	27.494
4	79.982	2234251	28025	14.722	14.722
5	106.218	2269024	16613	14.951	14.951
Total		15176764	205257	100.000	100.000

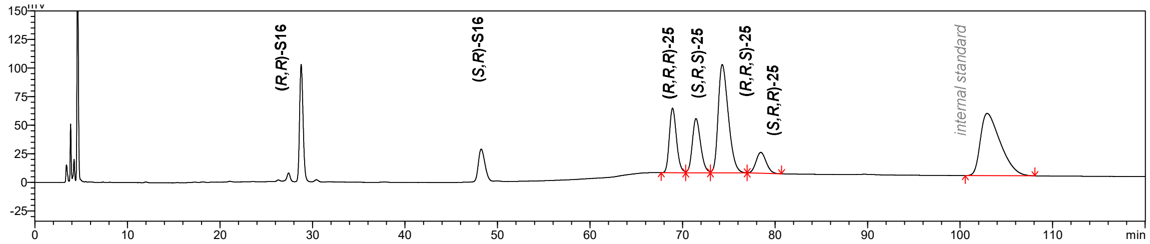

Peak#	Ret. Time	Area	Height	Conc.	Area%
1	68.918	3042566	56488	13.365	13.365
2	71.457	2958629	47433	12.997	12.997
3	74.293	7228342	94768	31.753	31.753
4	78.464	1389332	18282	6.103	6.103
5	102.927	8145561	54444	35.782	35.782
Total		22764429	271416	100.000	100.000

Peak#	Ret. Time	Area A	Area/Area^{IS}	Corr. A/A^{IS}	d.r.
1	68.92	3042566	0.373524	0.365842	2.2
2	71.46	2958629	0.363219	0.346022	2.0
3	74.29	7228342	0.887396	0.851628	5.0
4	78.760	1389332	0.170563	0.169900	1.0
5	102.93	8145561			

Assignment of the (*R*)-derived monoadduct and dimeric mixture:

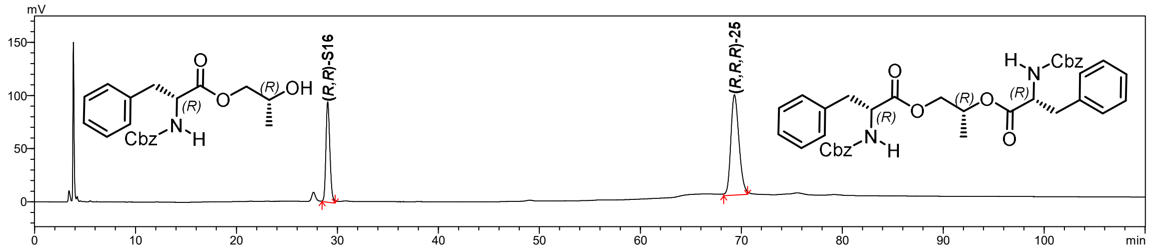

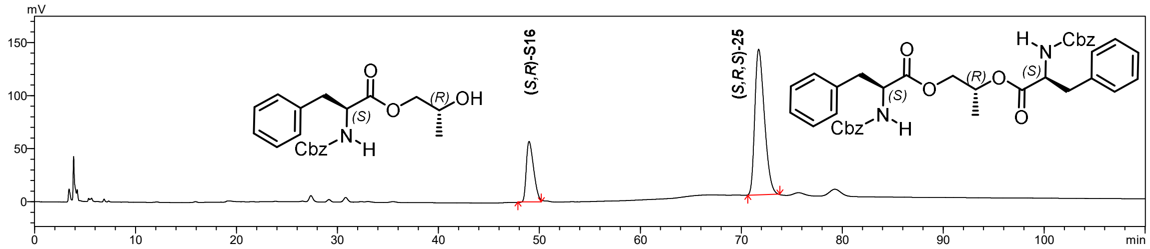

Note: these mixtures were prepared by reaction of enantiopure (*R*)- or (*S*)-**23** with (*R*)-1,2-propanediol, using NEt_3 and DMAP.

Kinetic resolution data for the reaction using (*R*)-diol 22:

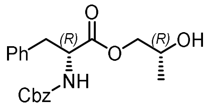

(*R*,*R*)-**S16**

Isolated as a mixture of diastereoisomers and regioisomers.

***R*f** = 0.42 (50% EtOAc in *n*-hexane), stained with $KMnO_4$;

^{1}H NMR (500 MHz, $CDCl_3$): δ 7.38–7.23 (m, 8H), 7.17–7.12 (m, 2H), 5.31–5.21 (m, 1H), 5.10 (s, 2H), 4.67–4.56 (m, 1H), 4.15–4.03 (m, 1H), 3.97–3.86 (m, 2H), 3.11 (h, *J* = 7.3 Hz, 2H), 1.66 (br s, 1H), 1.13 (d, *J* = 6.5 Hz, 3H);

^{13}C NMR (126 MHz, $CDCl_3$): δ = 171.8, 156.0, 136.2, 135.8, 129.4, 128.9, 128.7, 128.4, 128.3, 127.4, 70.7, 67.3, 65.7, 55.3, 38.4, 18.9;

HRMS (ESI$^+$): Calc. for $C_{20}H_{23}NO_5Na$ $[M+Na]^+$: 380.1468, found: 380.1474;

IR (thin film, cm^{-1}): 3353, 2926, 1704, 1512, 1497, 1455, 1263, 1212, 1054, 1028, 735;

HPLC: Daicel Chiralpak® ID column with guard, gradient 10% *i*-PrOH in *n*-hexane over 10 min; 10–20% *i*-PrOH in *n*-hexane over 10 min; 20% *i*-PrOH in *n*-hexane over 20 min; 20–50% *i*-PrOH in *n*-hexane over 20 min; 1.0 mL/min, 30 °C, 220 nm, t_R = 28.64 min (*R,R* isomer), t_R = 48.22 min (*S,R* isomer), 43% de.

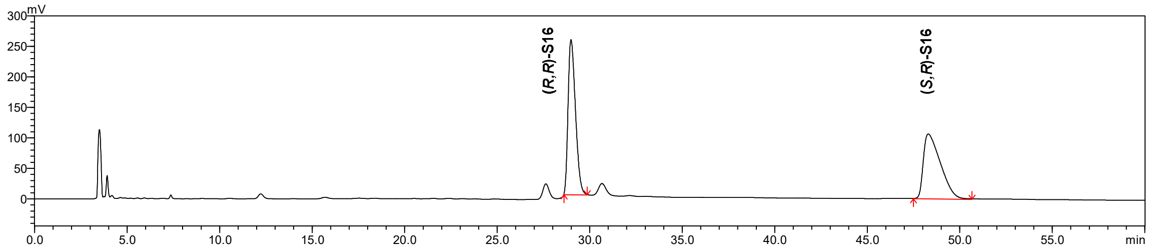

Peak#	Ret. Time	Area	Height	Conc.	Area%
1	28.990	7098046	254381	50.818	50.818
2	48.299	6869432	106501	49.182	49.182
Total		13967478	360882	100.000	100.000

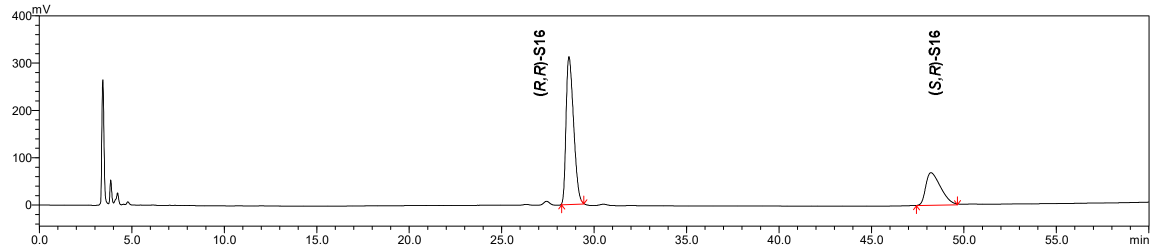

Peak#	Ret. Time	Area	Height	Conc.	Area%
1	28.640	9353161	317821	71.016	71.016
2	48.219	3817272	68585	28.984	28.984
Total		13170433	386406	100.000	100.000

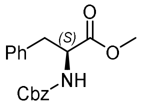

(*S*)-**S19**

HPLC: Daicel Chiralcel® OD-H column with guard, gradient 5% *i*-PrOH in *n*-hexane; 1.0 mL/min, 30 °C, 220 nm, t_R = 20.25 min (*S*-isomer), t_R = 23.54 min (*R*-isomer), 42% ee.

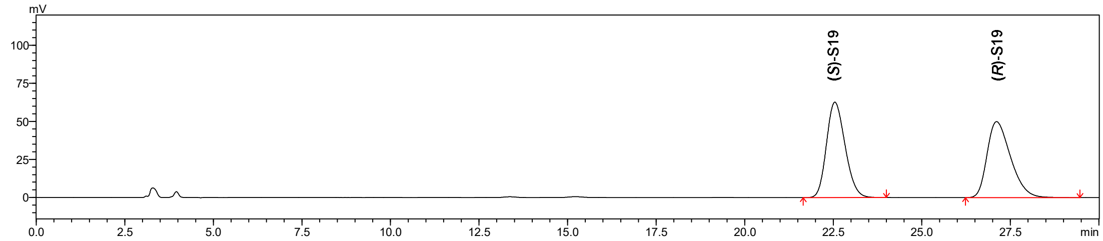

Peak#	Ret. Time	Area	Height	Conc.	Area%
1	22.545	2297856	62727	49.841	49.841
2	27.111	2312476	49983	50.159	50.159
Total		4610332	112709	100.000	100.000

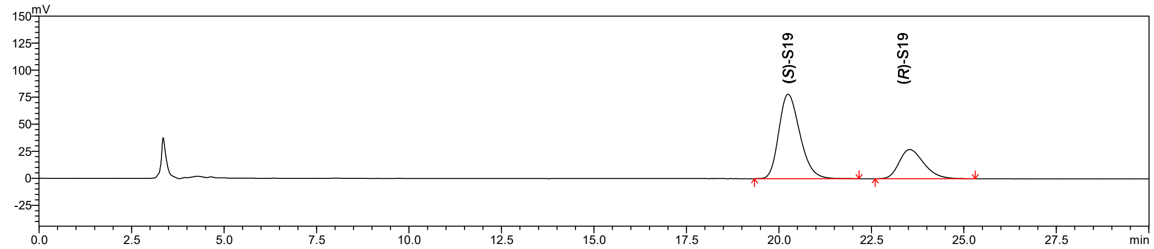

Peak#	Ret. Time	Area	Height	Conc.	Area%
1	20.248	3097527	78129	71.103	71.103
2	23.537	1258879	27043	28.897	28.897
Total		4356406	105171	100.000	100.000

6. NMR Spectra (with Table of Contents)

6. NMR Spectra (with Table of Contents) .... - 102 -

6.1 Stereoretentive Enantioconvergent *Aza*-Darzens Dimerization of Epoxide- 103 -

6.2 Stereoretentive Enantioconvergent Reaction with a Traceless Auxiliary... - 105 -

6.3. Biocatalytic Stereoretentive Enantioconvergent Reaction......................... - 111 -

6.4. Organocatalytic Stereoretentive Enantioconvergent Reaction .... - 117 -

6.5. Organocatalytic Stereoretentive Diastereoconvergent Reaction .... - 128 -

6.1 Stereoretentive Enantioconvergent *Aza*-Darzens Dimerization of Epoxide

6.1.1 ^{1}H NMR Spectrum of Aziridine 2 (600 MHz, $CDCl_3$)

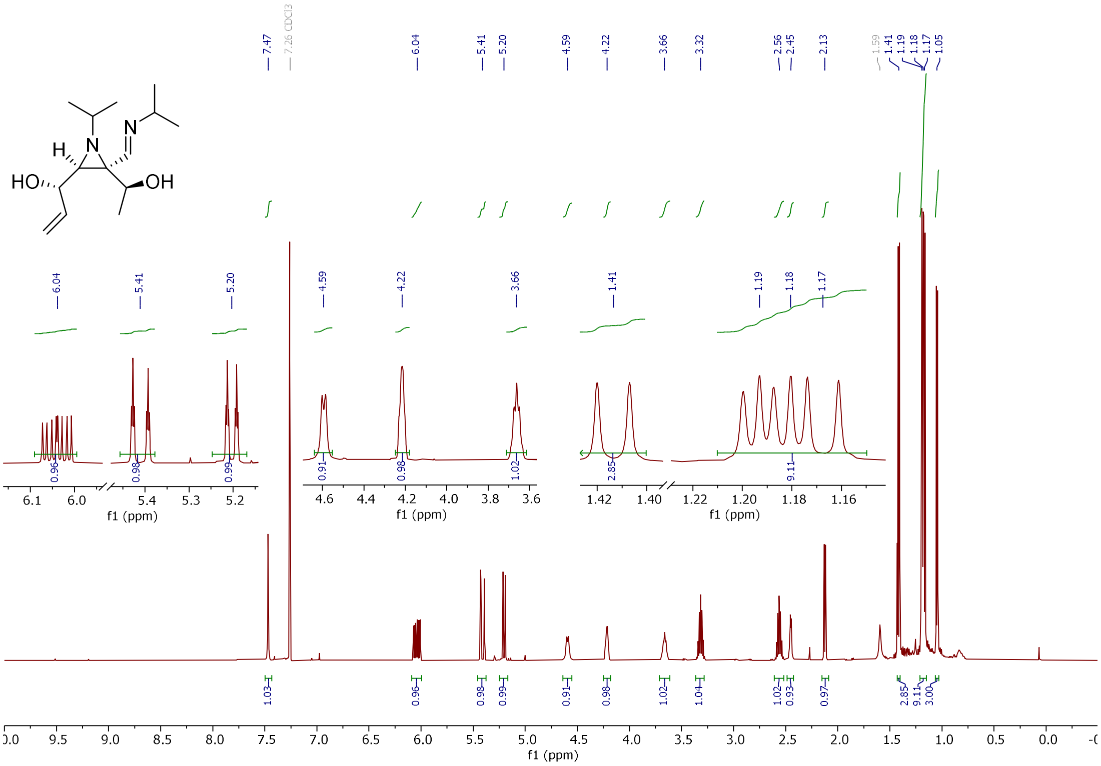

6.1.2 ^{13}C NMR Spectrum of Aziridine 2 (150 MHz, $CDCl_3$)

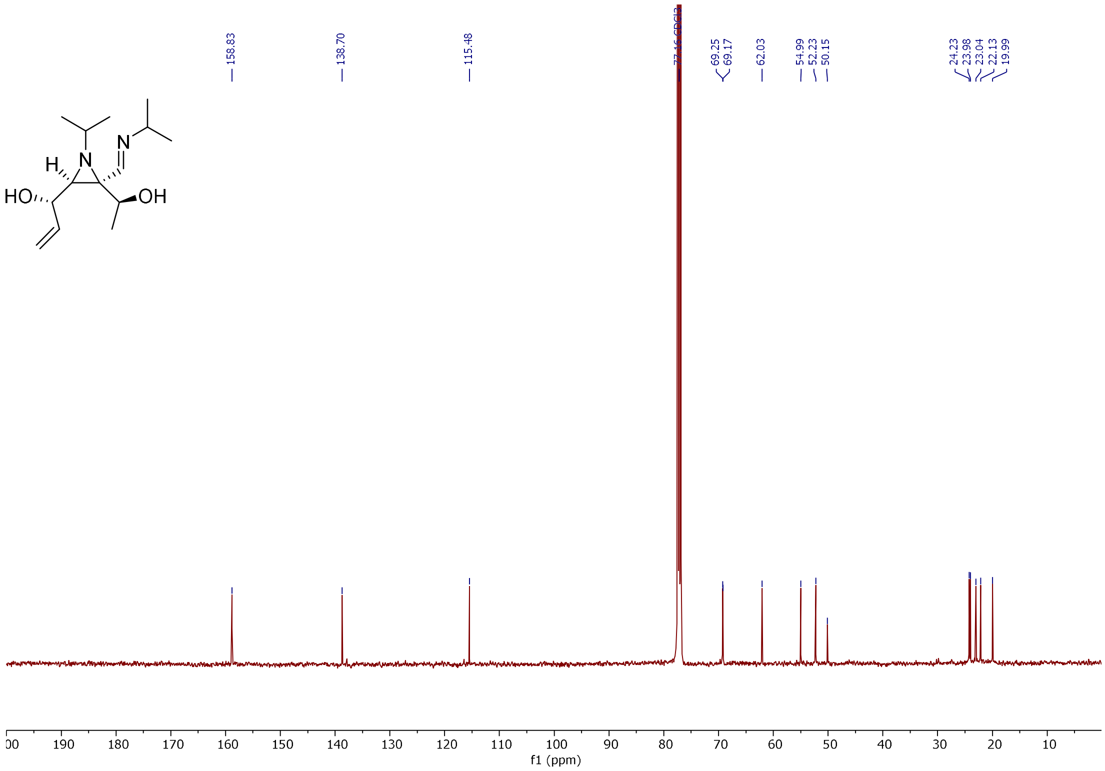

6.1.3 ^{1}H NMR Spectrum of Epoxide S1 (600 MHz, $CDCl_3$)

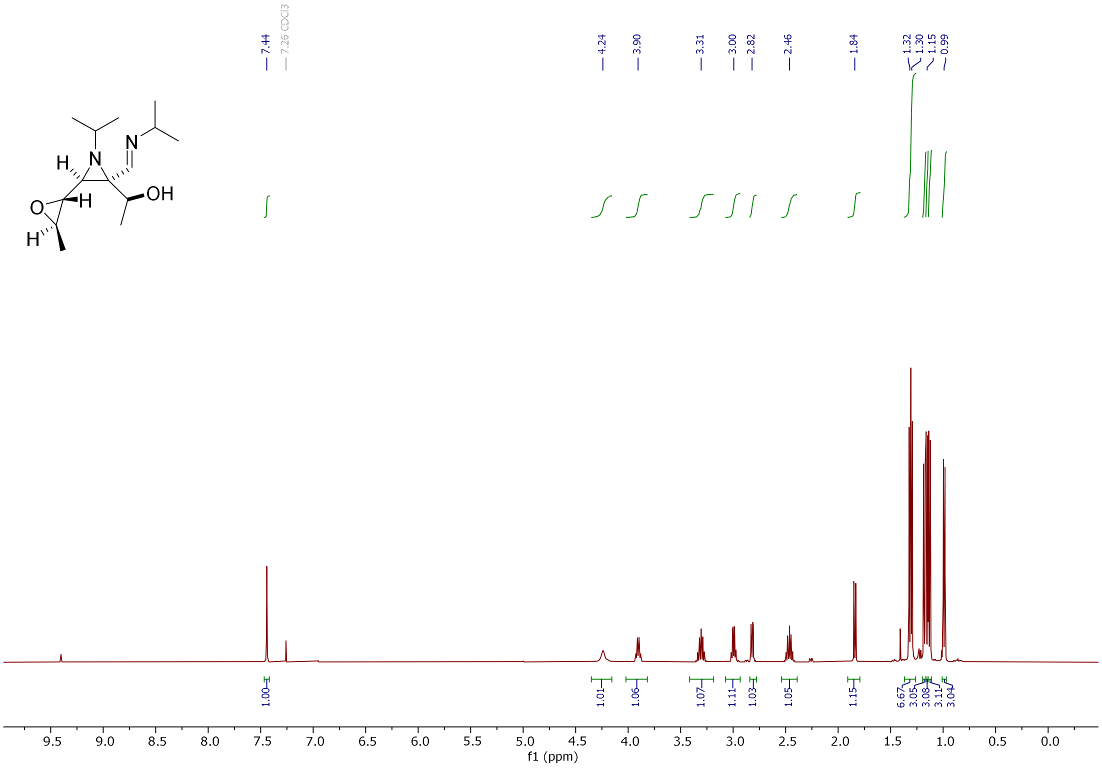

6.1.4 ^{13}C NMR Spectrum of epoxide S1 (150 MHz, $CDCl_3$)

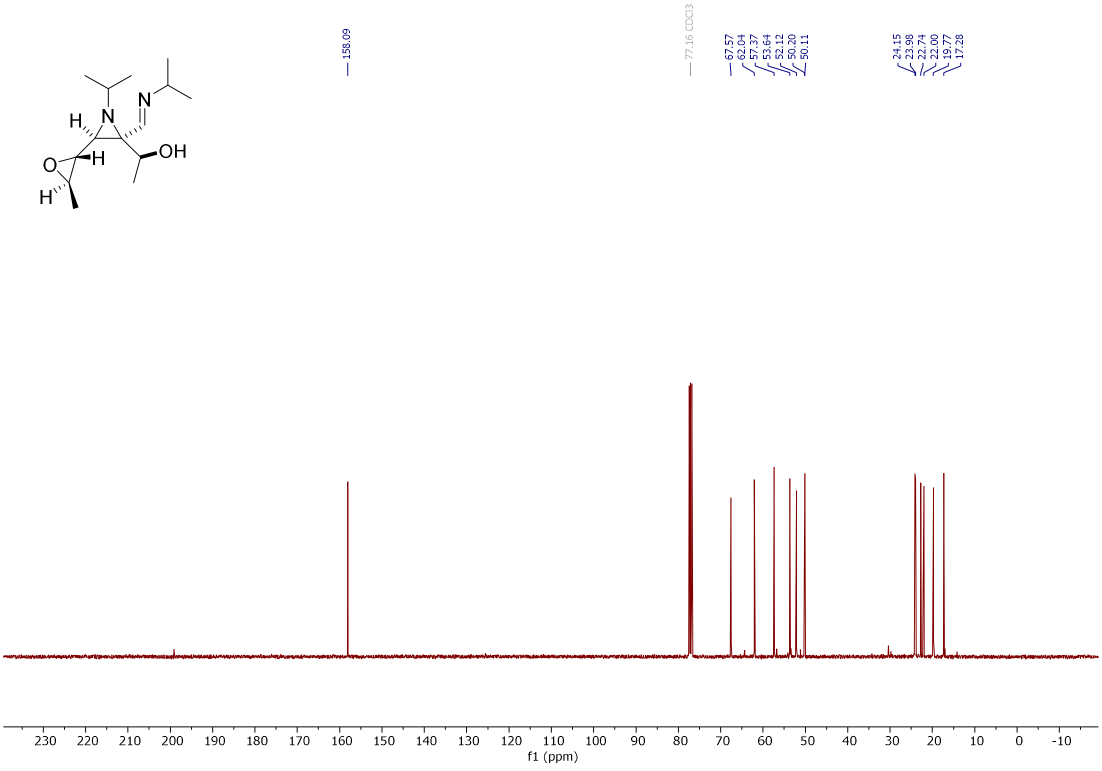

6.2 Stereoretentive Enantioconvergent Reaction with a Traceless Auxiliary

6.2.1 ^{1}H NMR Spectrum of Dimer (*S,R*)-12 (600 MHz, $CDCl_3$)

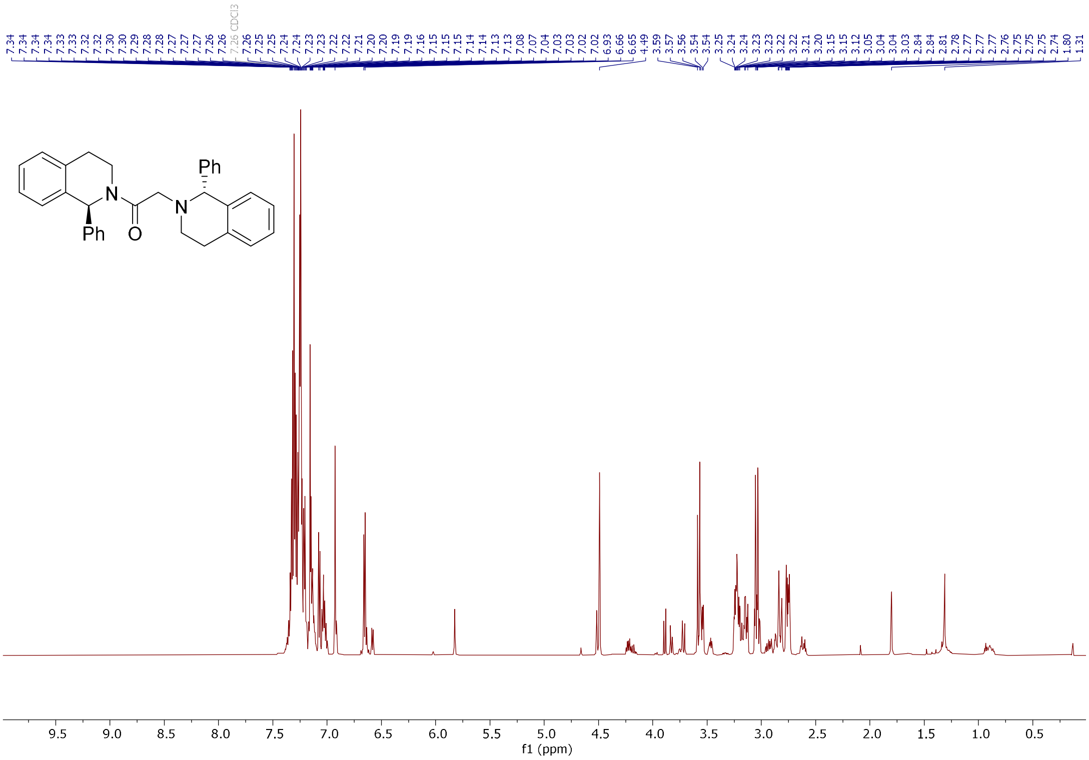

6.2.2 ^{13}C NMR Spectrum of Dimer (*S,R*)-12 (150 MHz, $CDCl_3$)

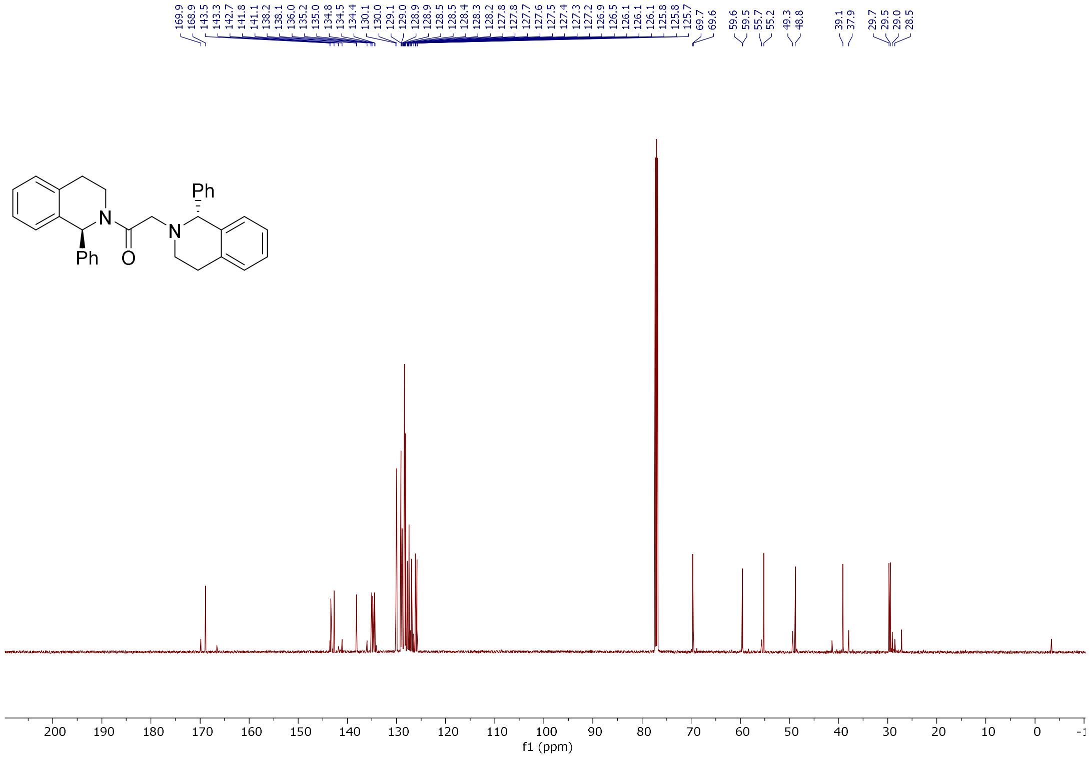

6.2.3 COSY Spectrum of Dimer (*S*,*R*)-12

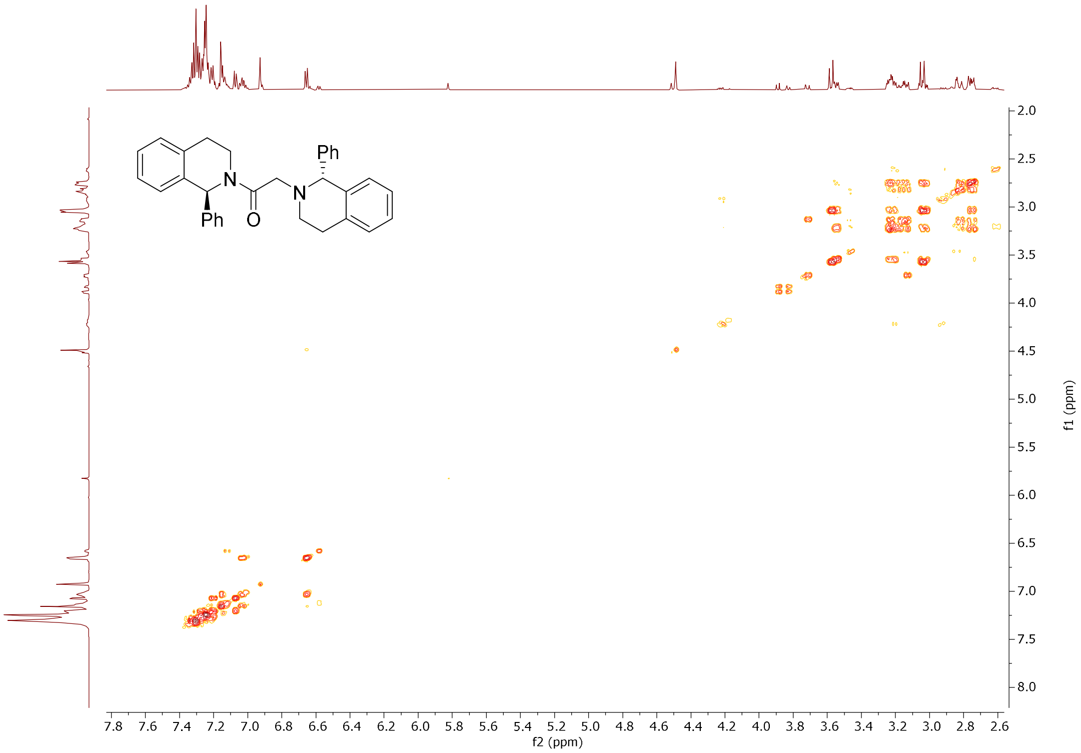

6.2.4 HSQC Spectrum of Dimer (*S*,*R*)-12

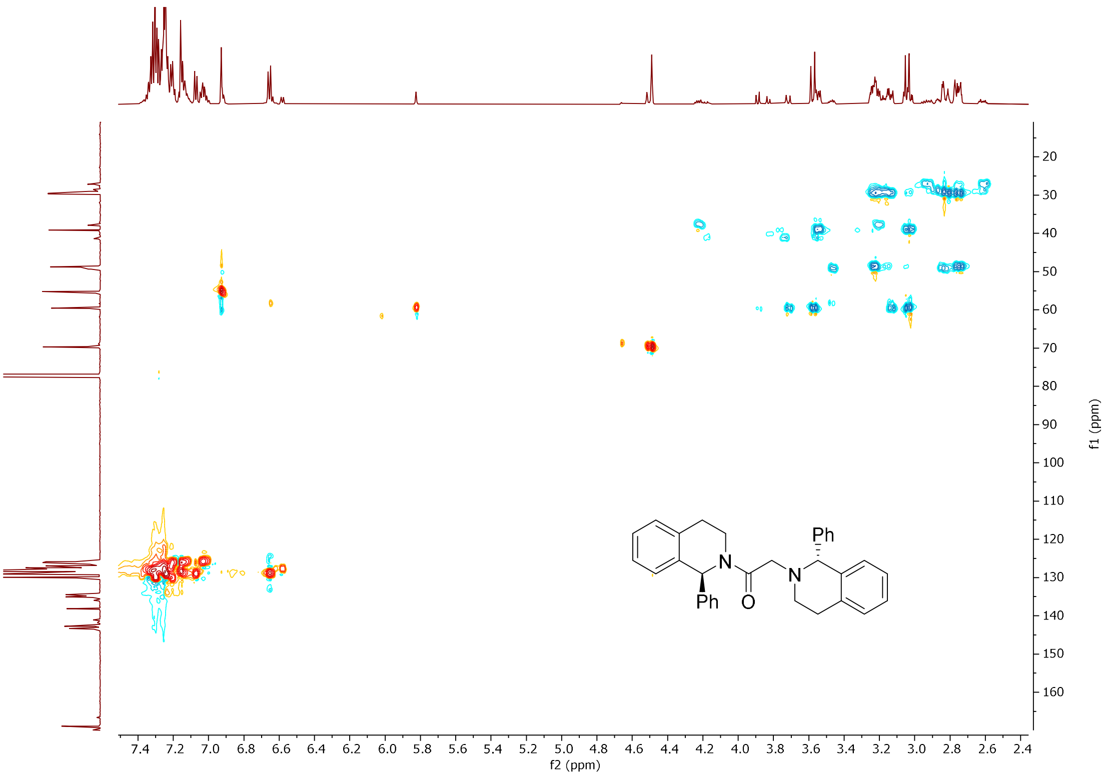

6.2.5 HMBC Spectrum of Dimer (*S*,*R*)-12

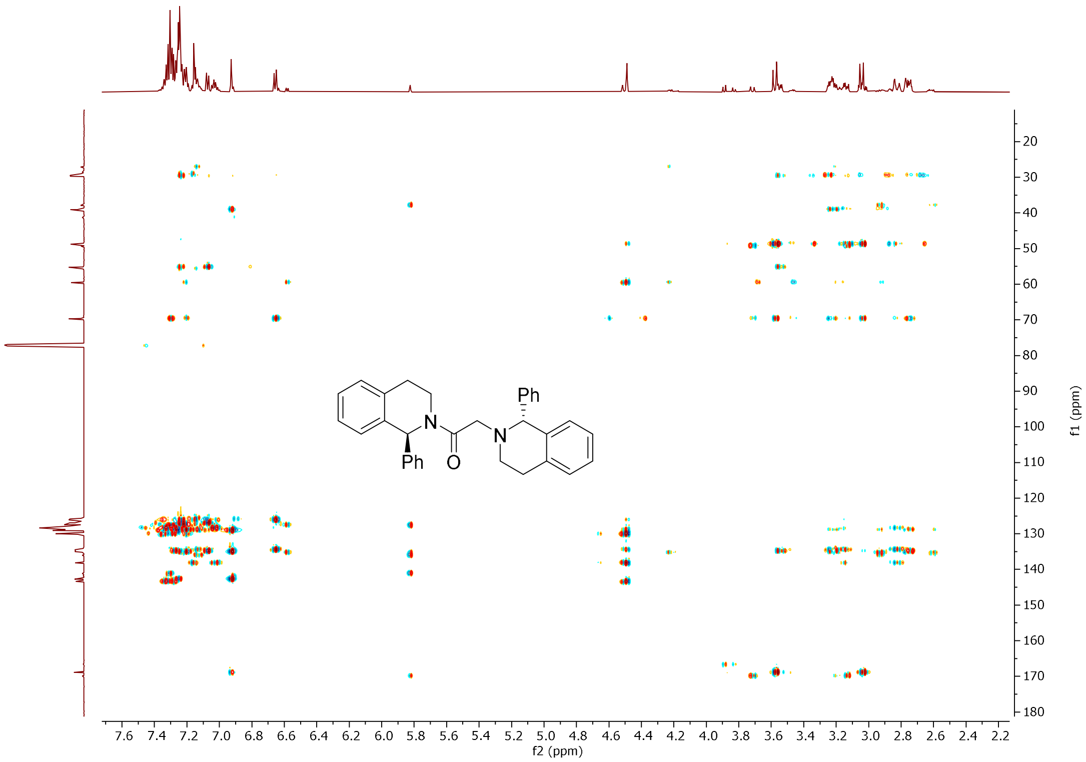

6.2.6 ^{1}H NMR Spectrum of Dimer (*S,S*)-12 (600 MHz, CDCl$_3$)

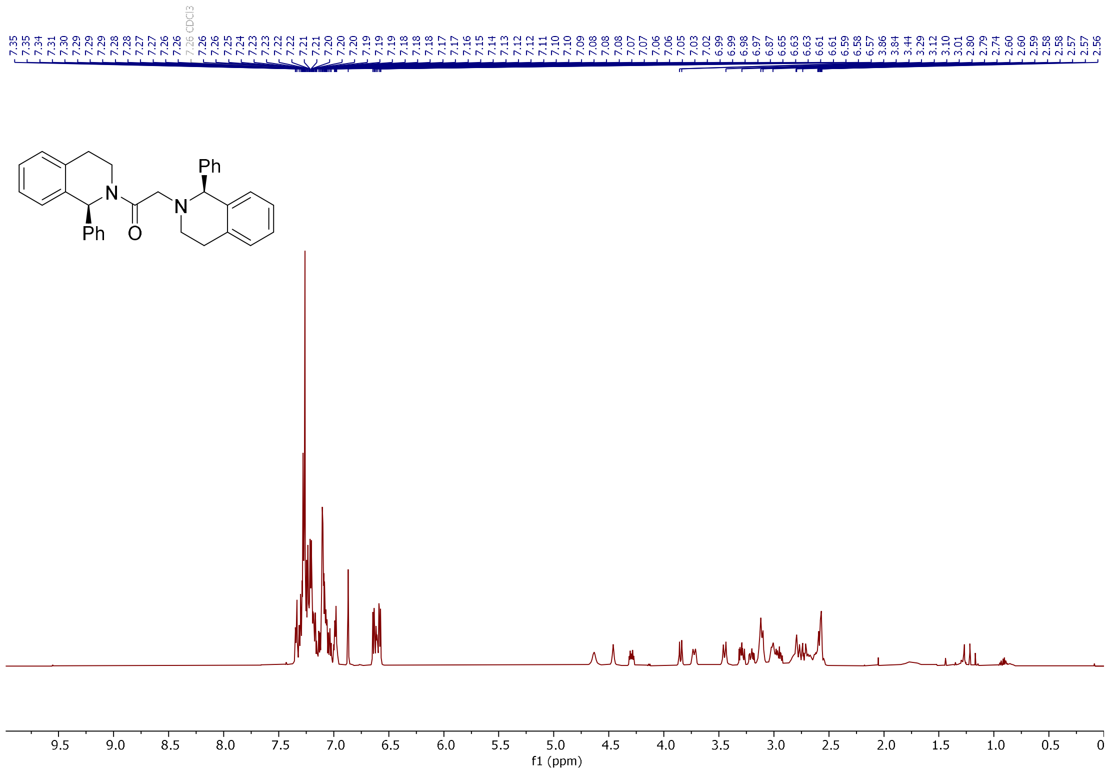

6.2.7 ^{13}C NMR Spectrum of Dimer (*S,S*)-12 (150 MHz, CDCl$_3$)

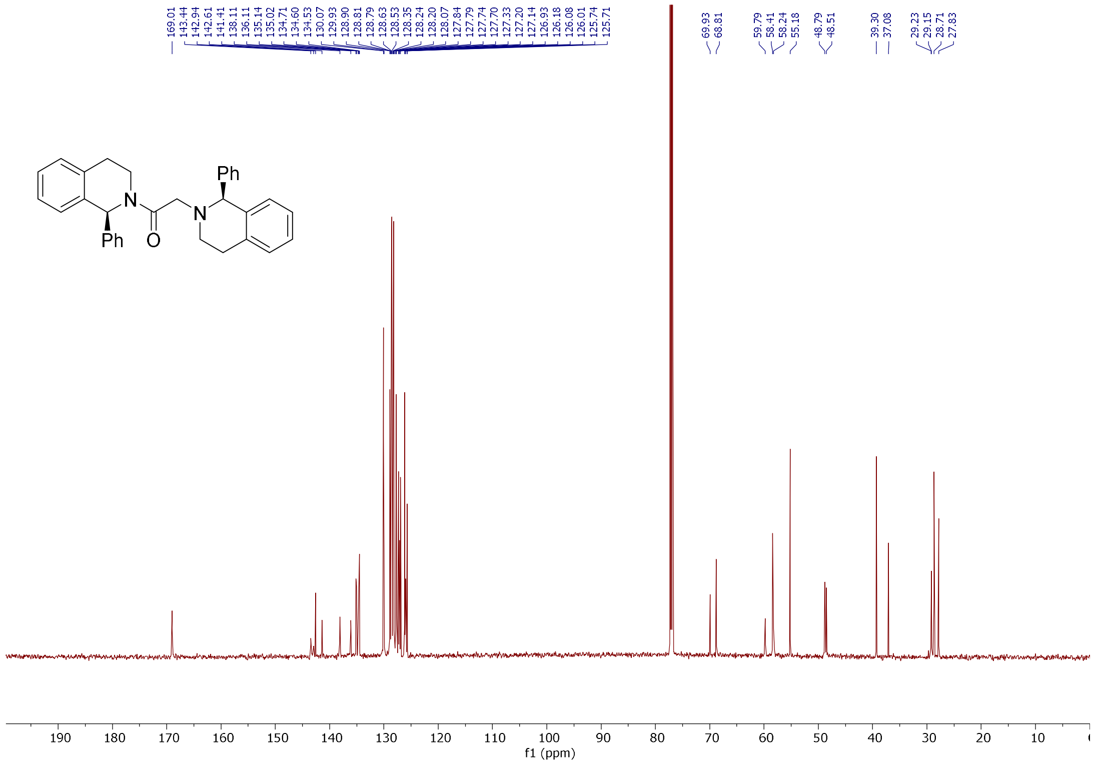

6.2.8 COSY Spectrum of Dimer (*S*,*S*)-12

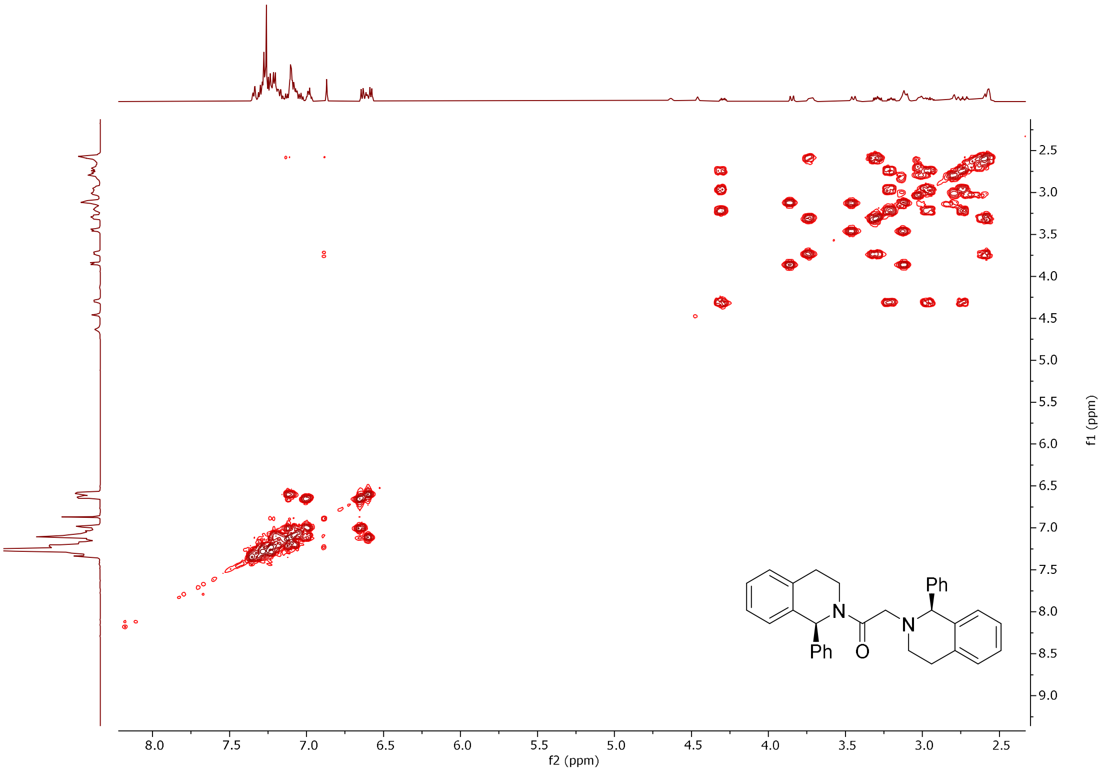

6.2.9 HSQC Spectrum of Dimer (*S*,*S*)-12

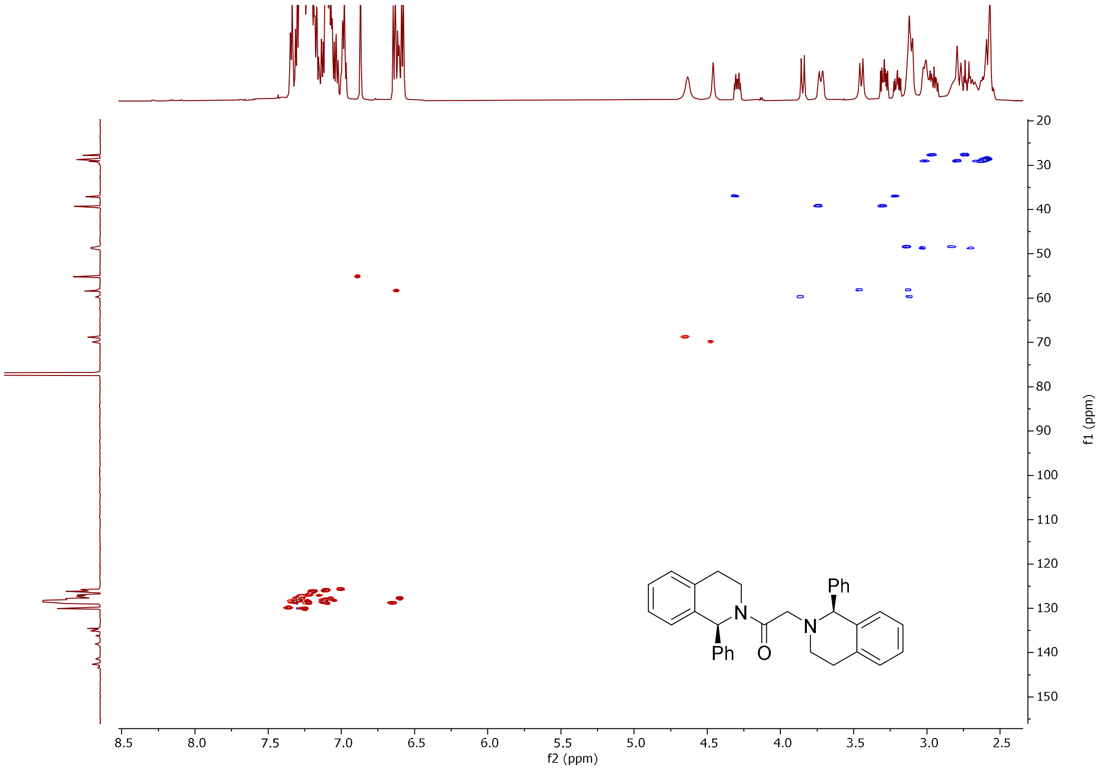

6.2.10 HMBC Spectrum of Dimer (*S*,*S*)-12

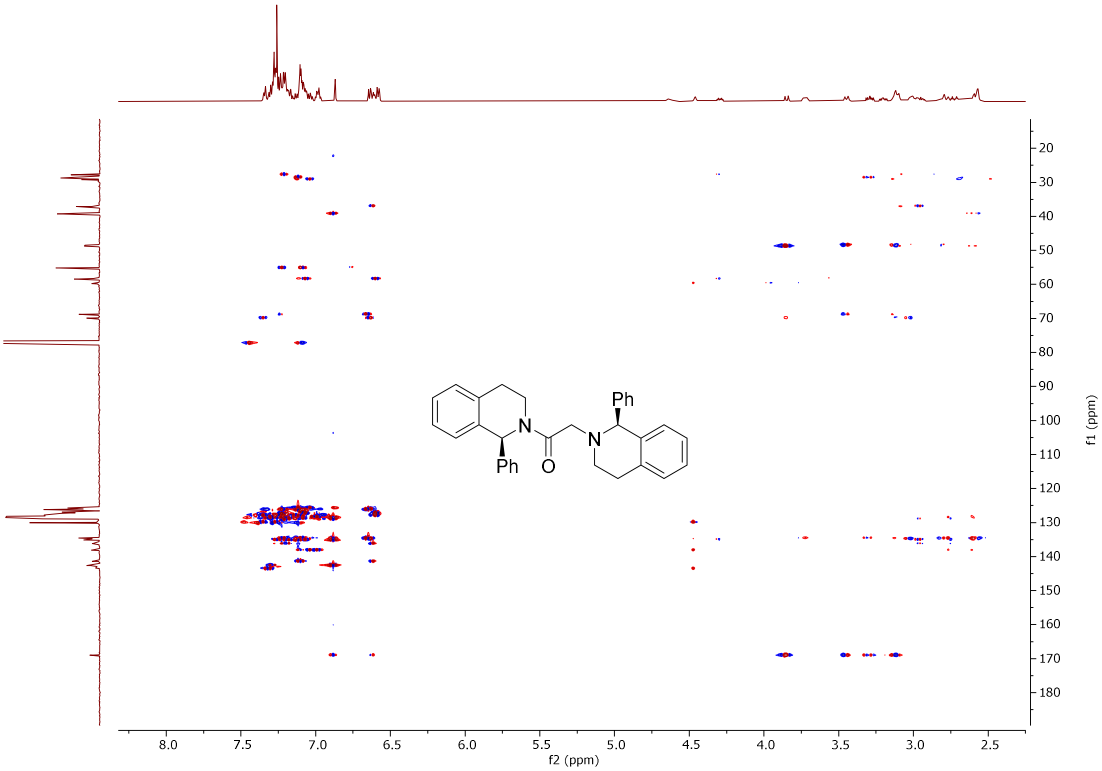

6.3. Biocatalytic Stereoretentive Enantioconvergent Reaction

6.3.1. ^{1}H NMR Spectrum of Compound (*rac*)-13 (500 MHz, $CDCl_3$):

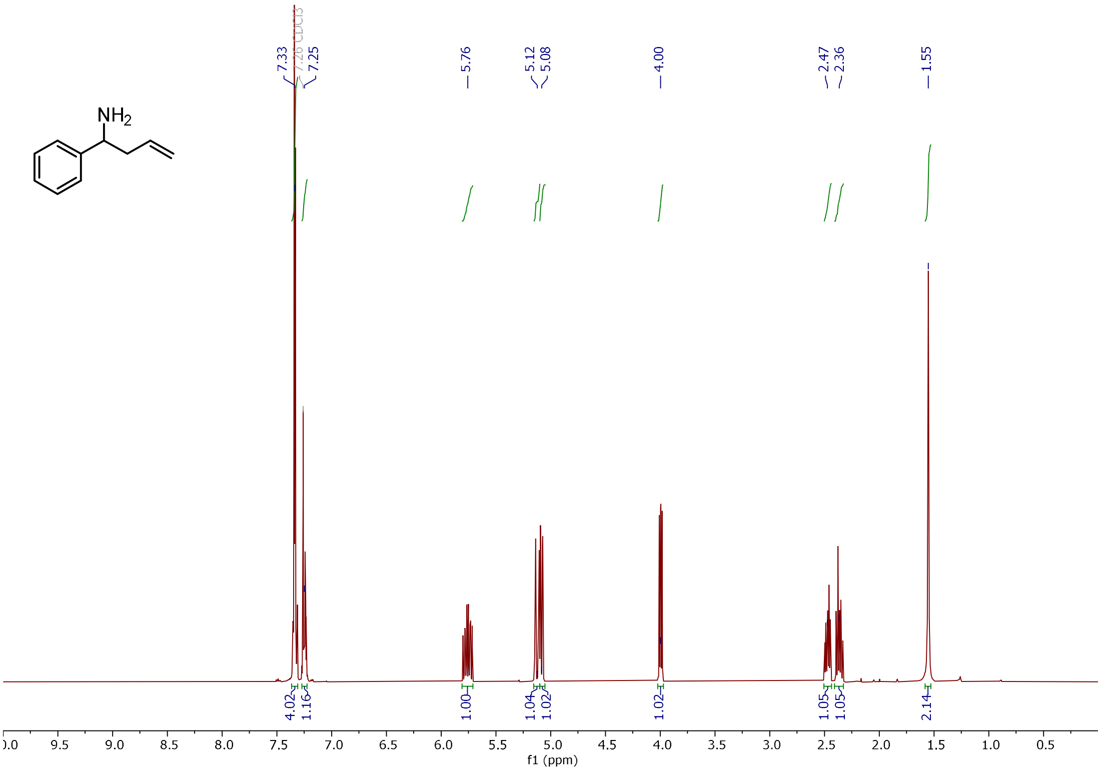

6.3.2. ^{13}C NMR Spectrum of Compound (*rac*)-13 (126 MHz, $CDCl_3$):

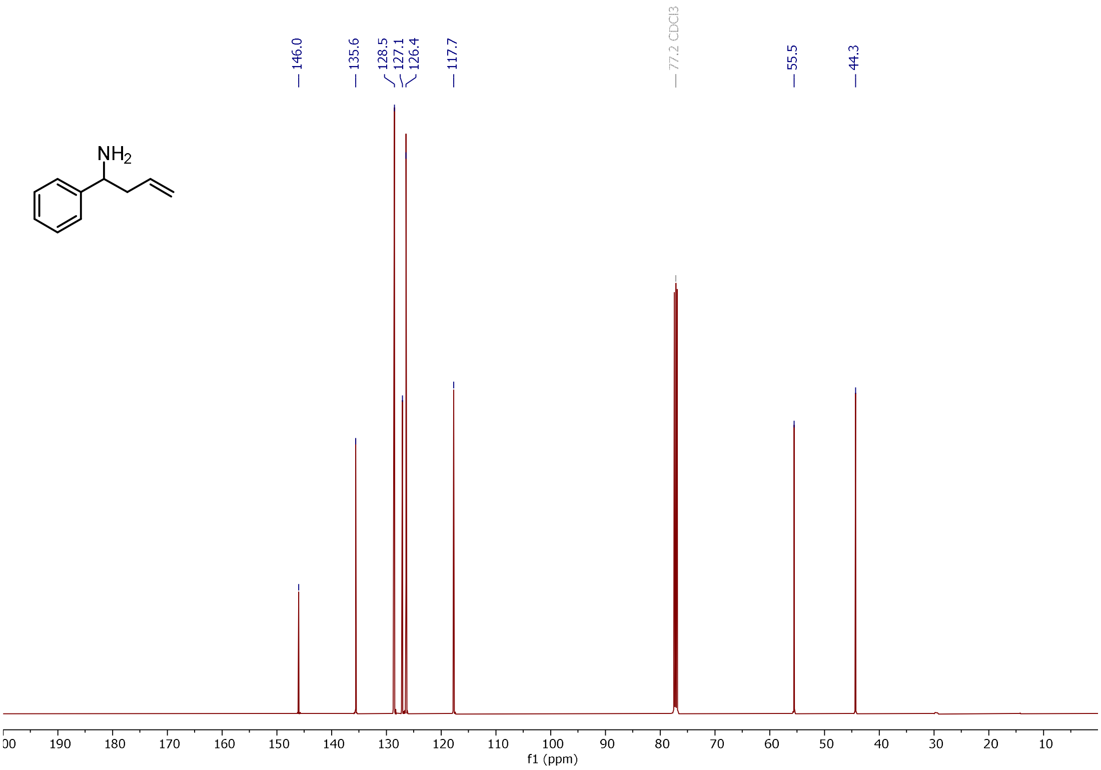

6.3.3. ^{1}H NMR Spectrum of Compound (*rac*)-14 (500 MHz, $CDCl_3$):

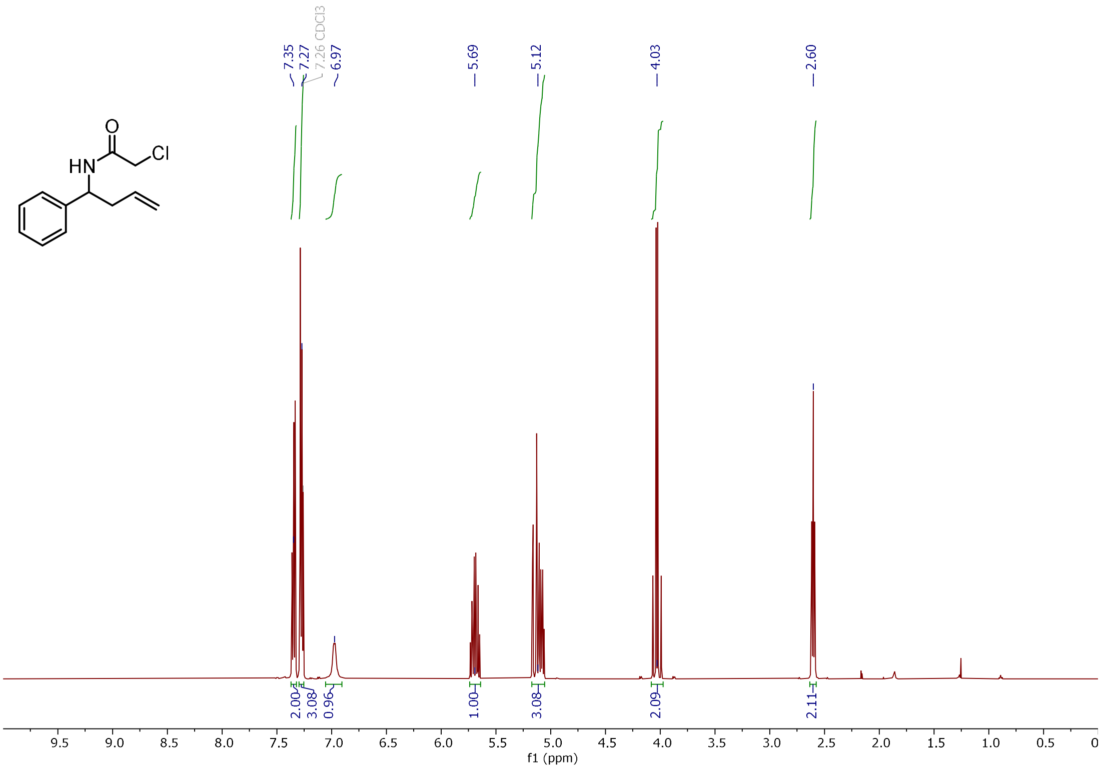

6.3.4. ^{13}C NMR Spectrum of Compound (*rac*)-14 (126 MHz, $CDCl_3$):

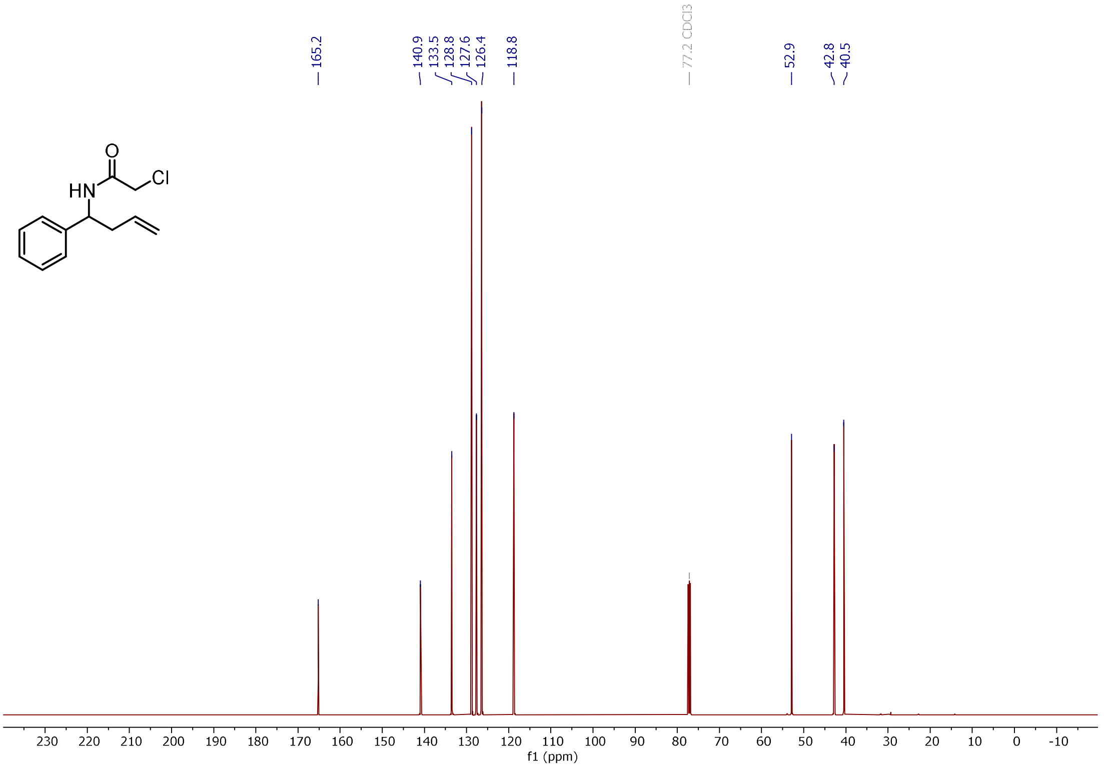

6.3.5. ^{1}H NMR Spectrum of Compound (*rac*)-homo/hetero-15 (601 MHz, $CDCl_3$):

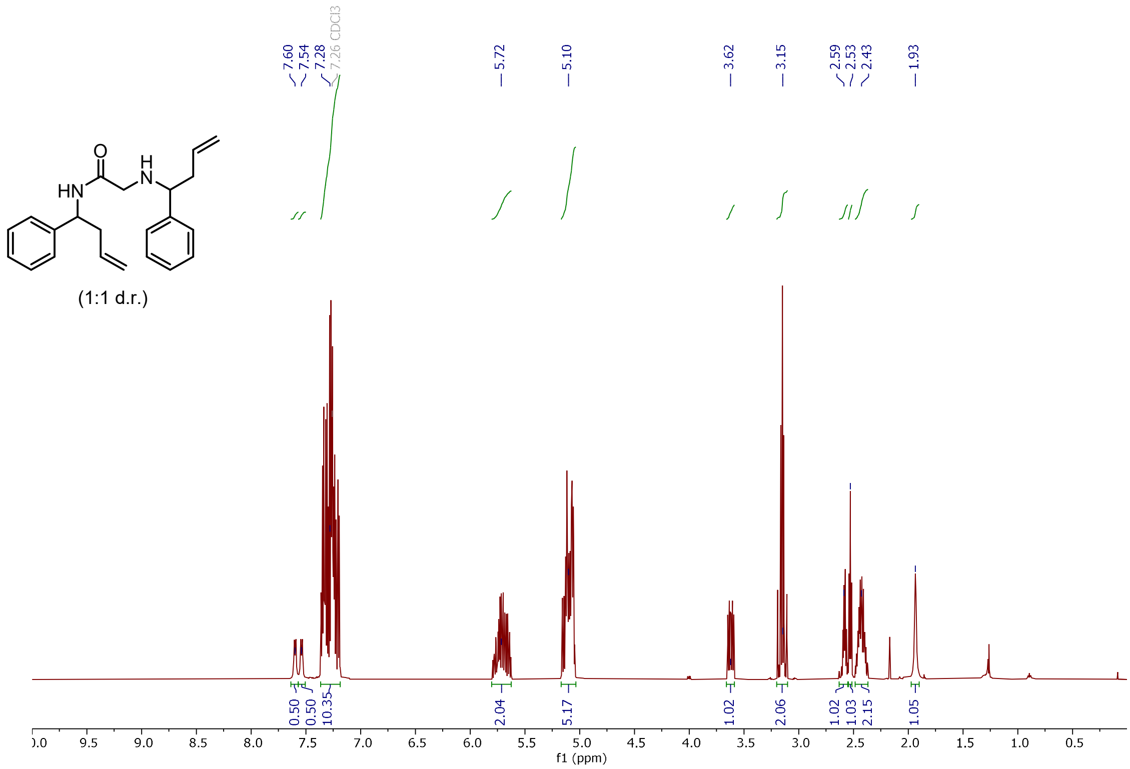

6.3.6. ^{13}C NMR Spectrum of Compound (*rac*)-homo/hetero-15 (151 MHz, $CDCl_3$):

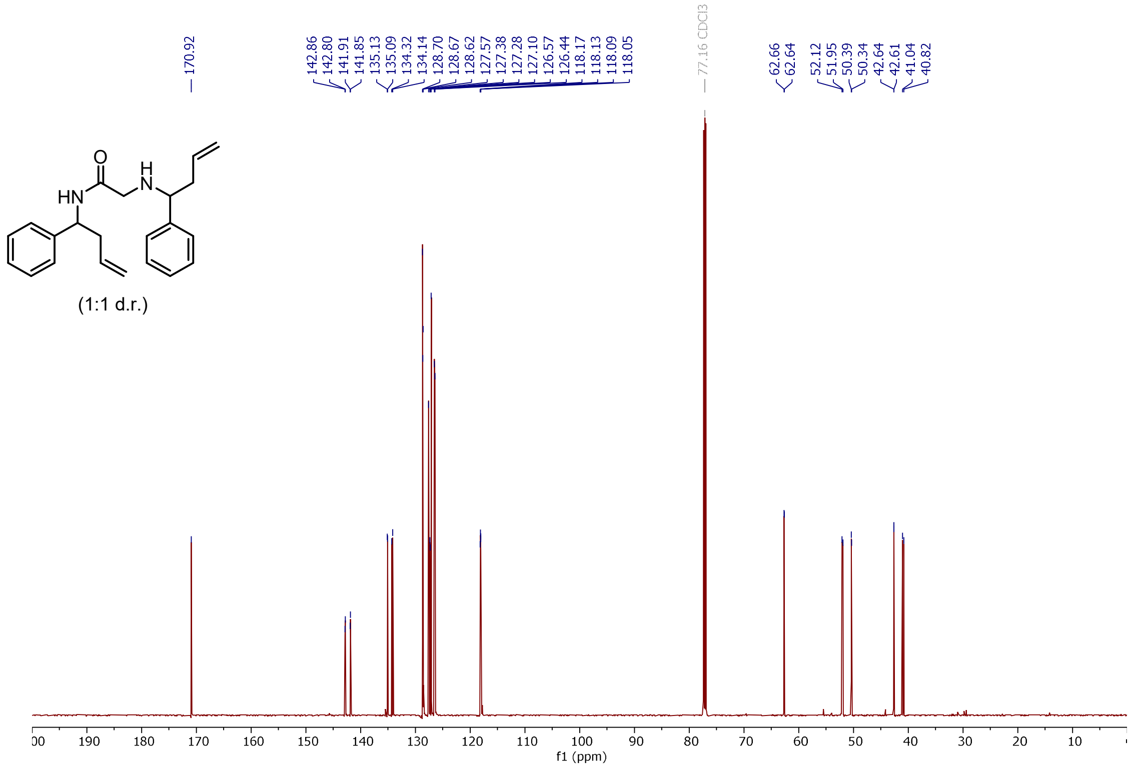

6.3.7. ^{1}H NMR Spectrum of Compound (*rac*)-homo-15 (601 MHz, CDCl3):

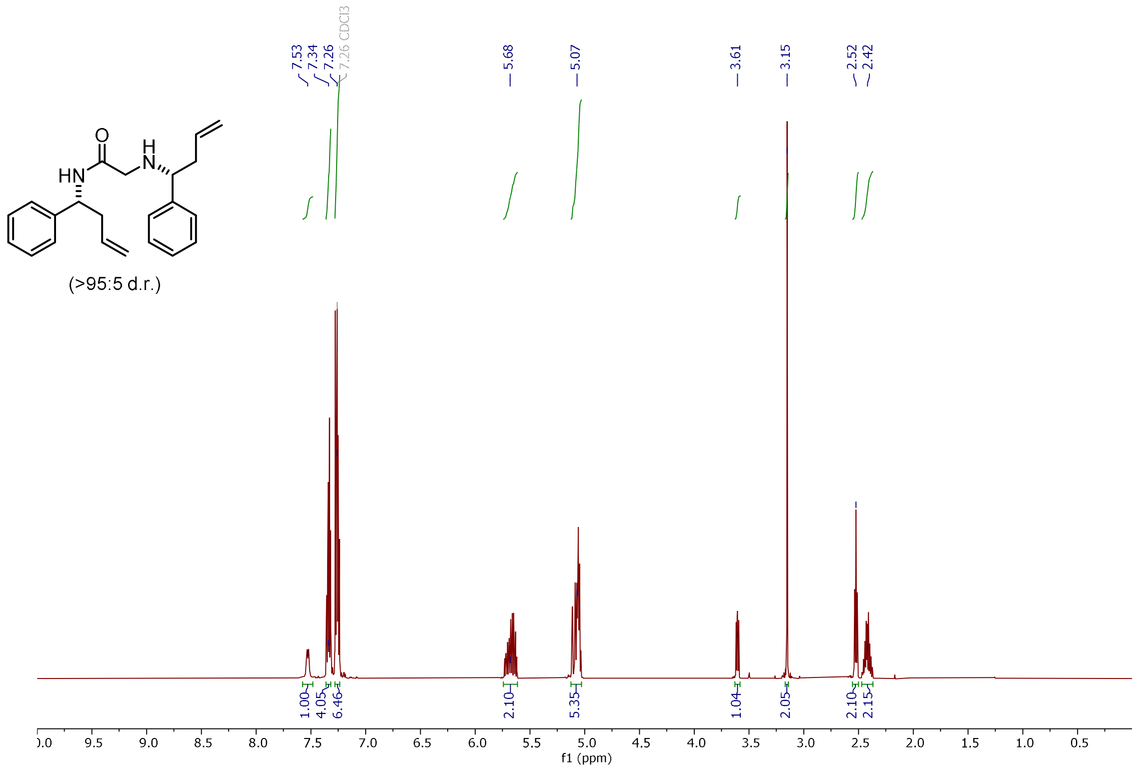

6.3.8. ^{13}C NMR Spectrum of Compound (*rac*)-homo-15 (151 MHz, CDCl3):

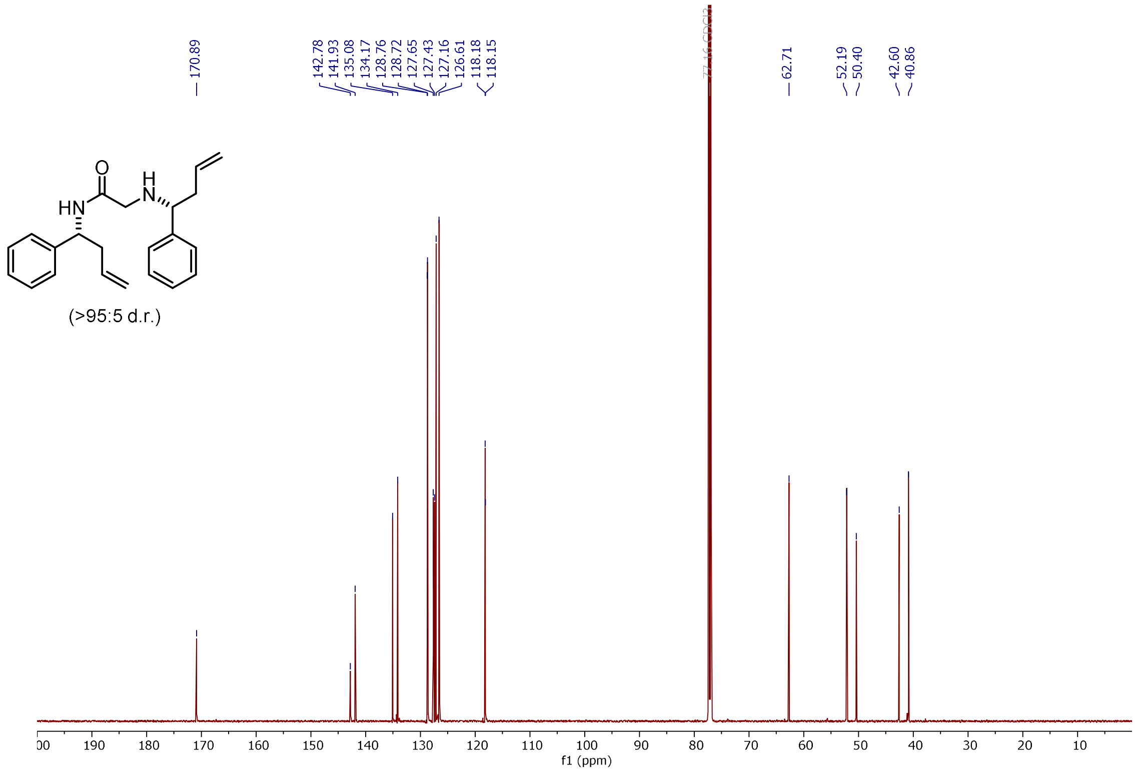

6.3.9. ^{1}H NMR Spectrum of Compound (*rac*)-S8 (601 MHz, $CDCl_3$):

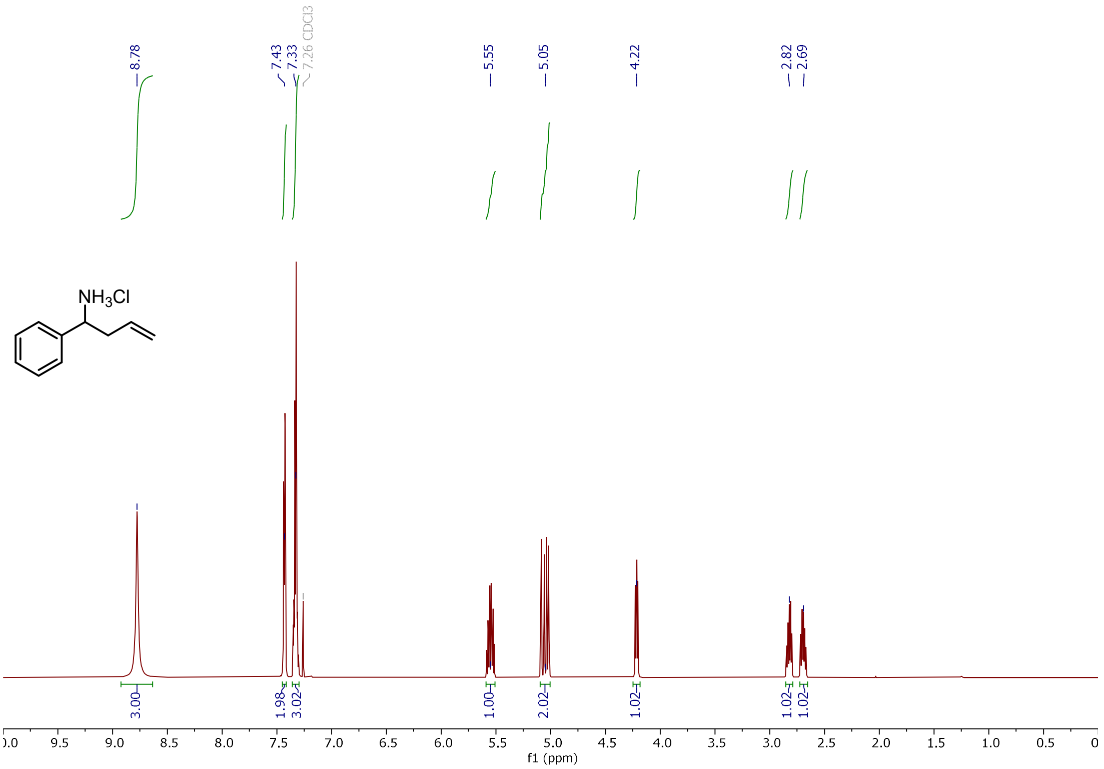

6.3.10. ^{13}C NMR Spectrum of Compound (*rac*)-S8 (151 MHz, $CDCl_3$):

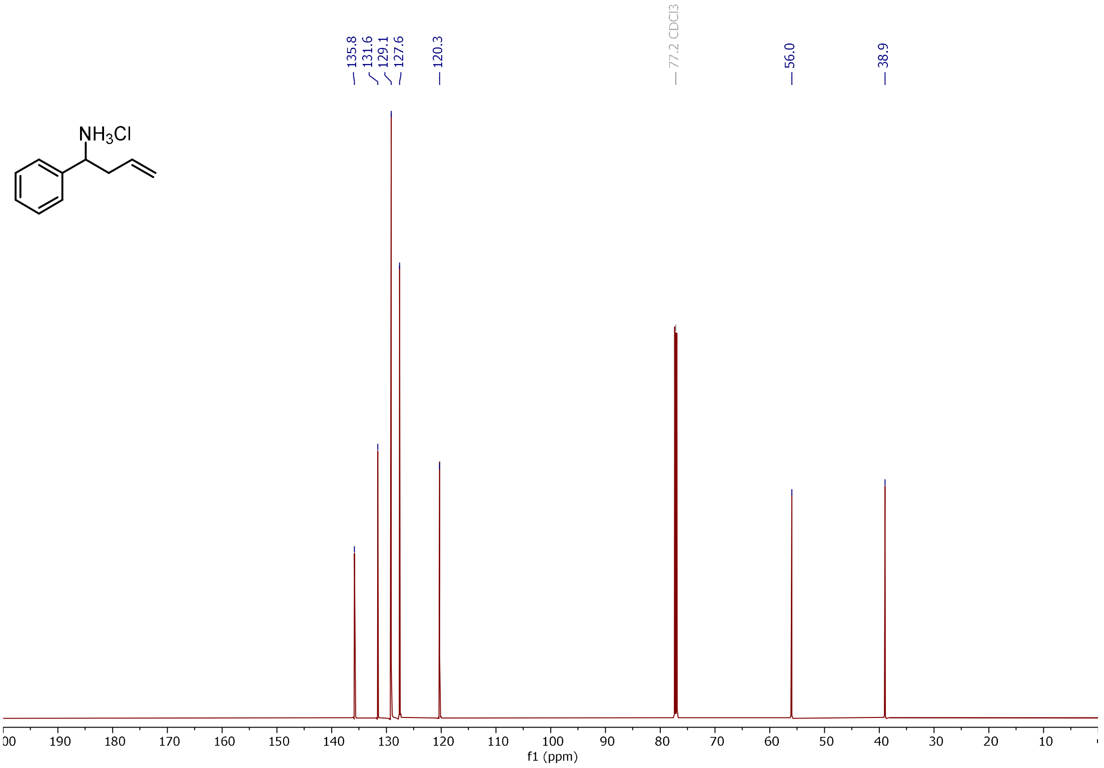

6.3.11. ^{1}H NMR Spectrum of Compound (*R*,*S*)-Hetero-15 (601 MHz, CDCl$_3$):

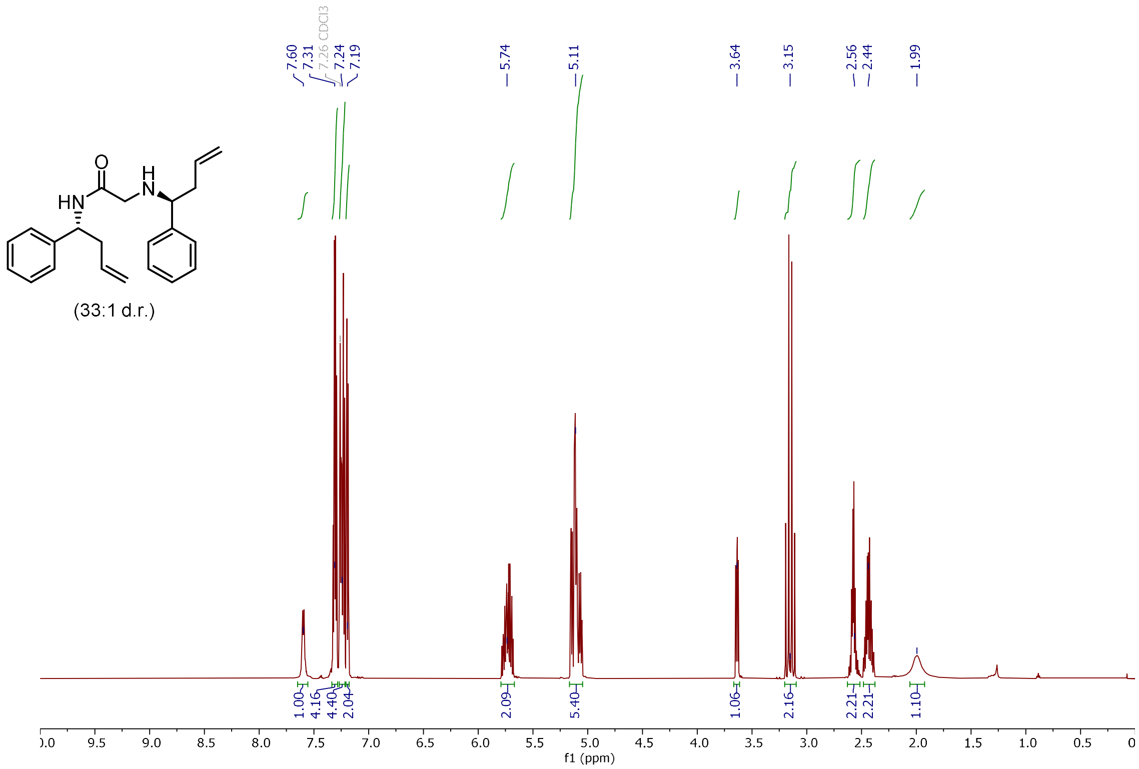

6.3.12. ^{13}C NMR Spectrum of Compound (*R*,*S*)-Hetero-15 (151 MHz, CDCl$_3$):

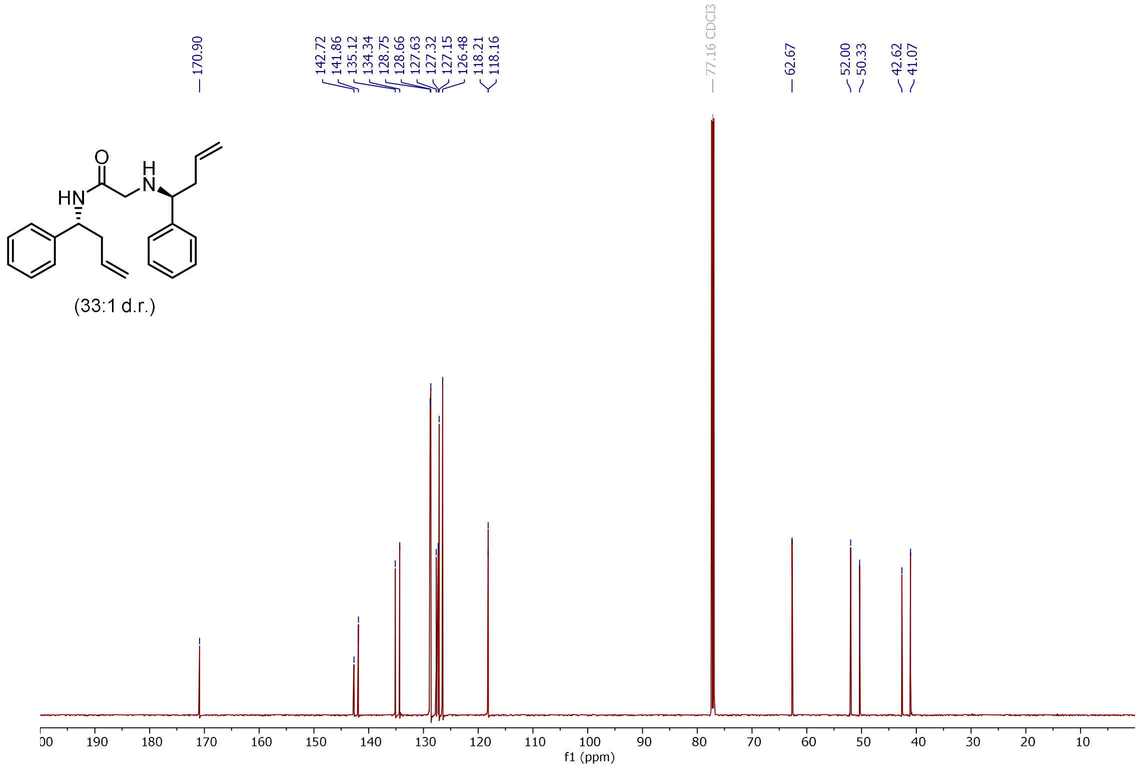

6.4. Organocatalytic Stereoretentive Enantioconvergent Reaction

6.4.1 ^{1}H NMR Spectrum of Compound S9 (500 MHz, $CDCl_3$)

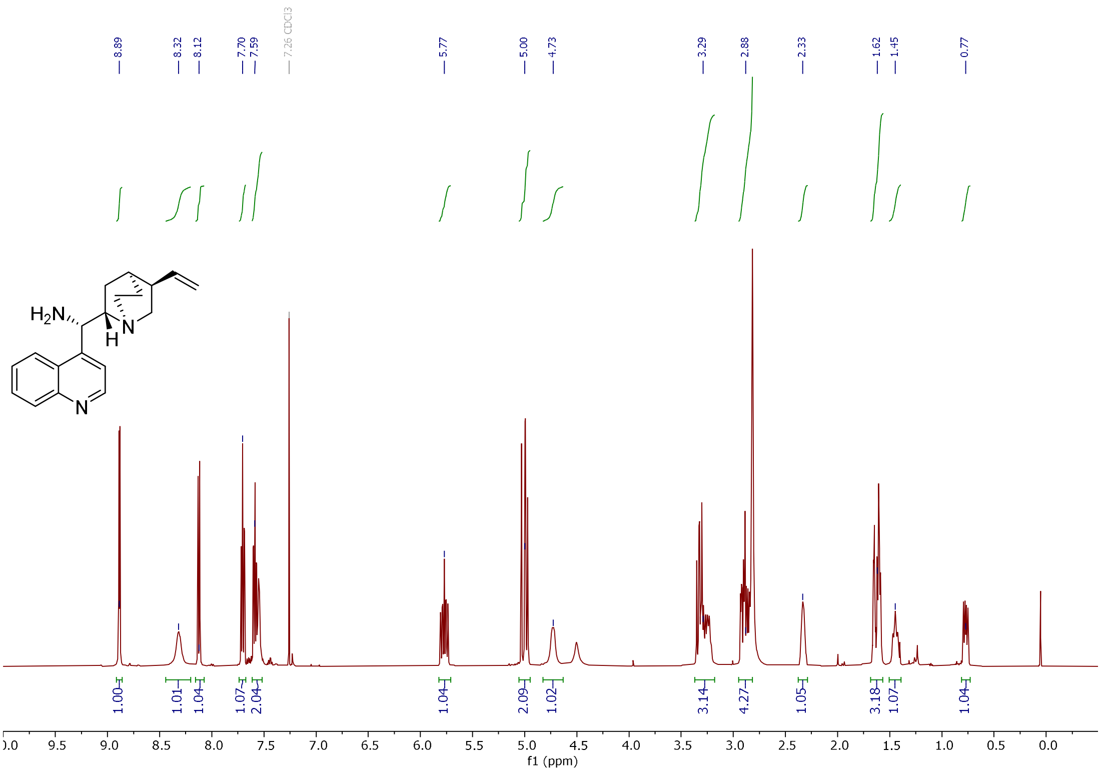

6.4.2 ^{13}C NMR Spectrum of Compound S9 (125 MHz, $CDCl_3$)

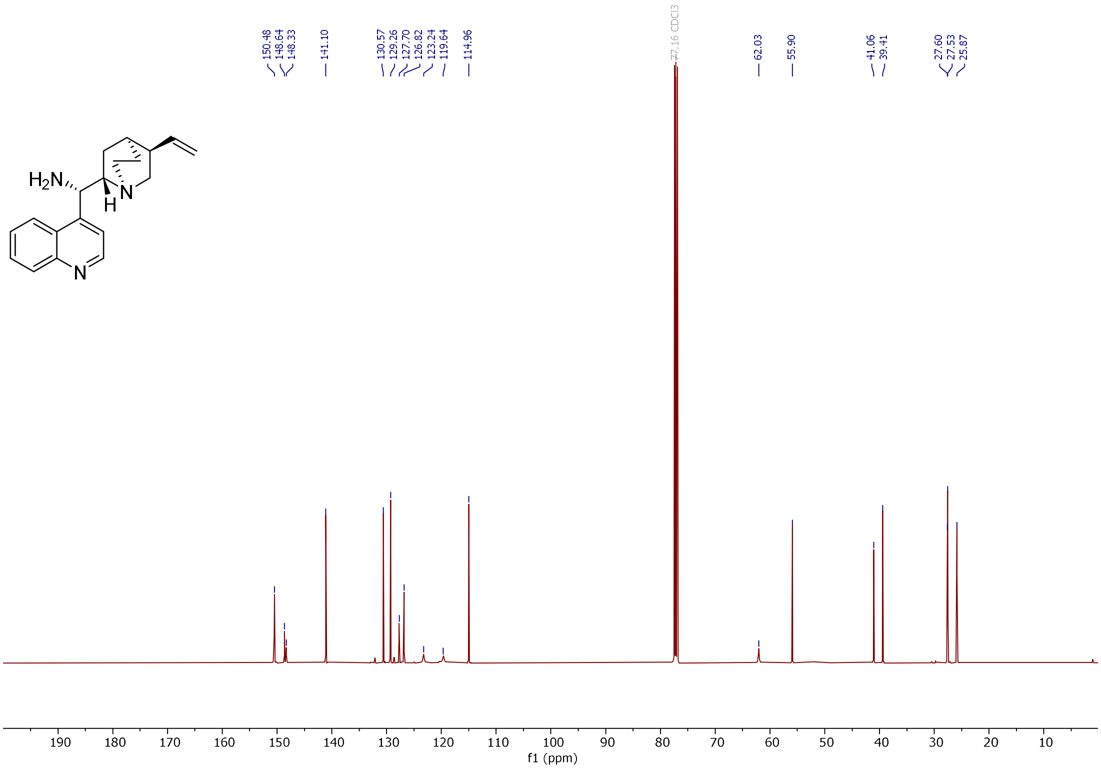

6.4.3 ^{1}H NMR Spectrum of Compound 18 (600 MHz, MeOD)

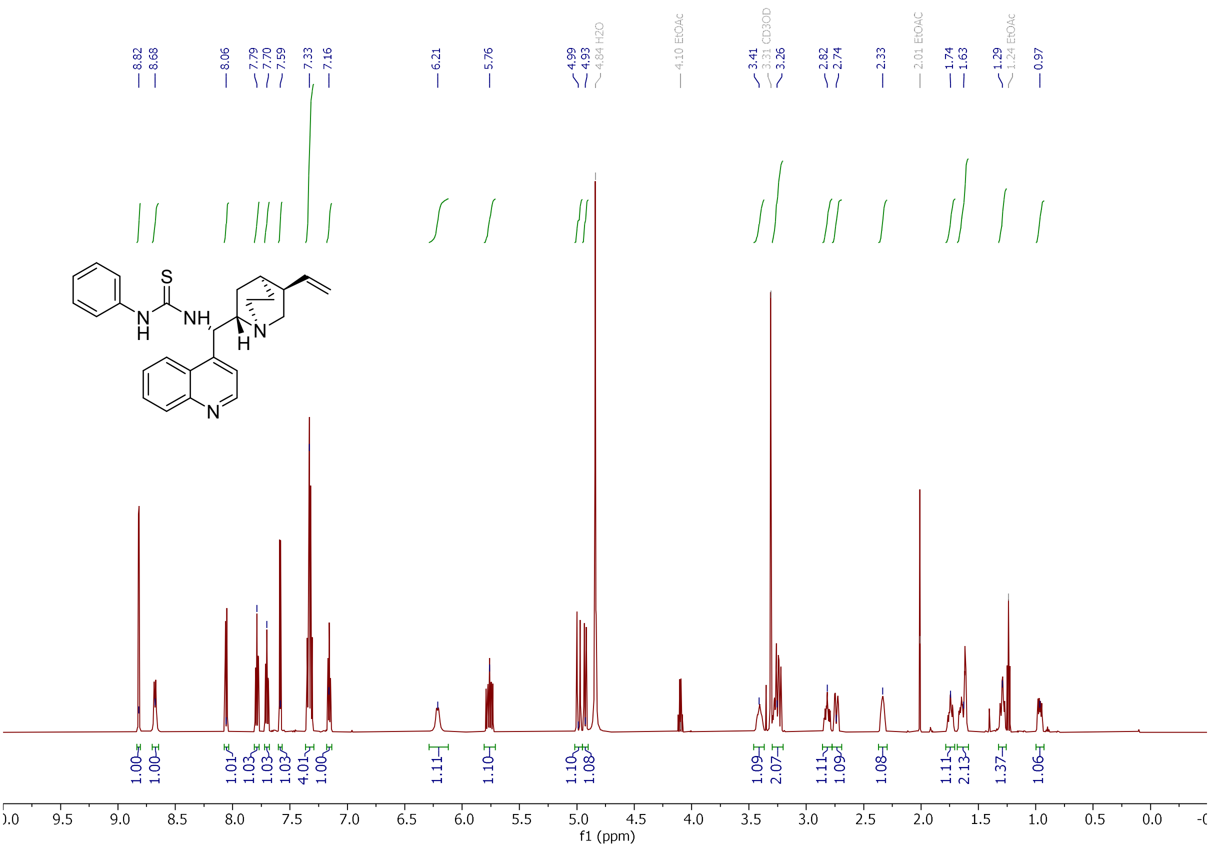

6.4.4 ^{13}C NMR Spectrum of Compound 18 (150 MHz, $CDCl_3$)

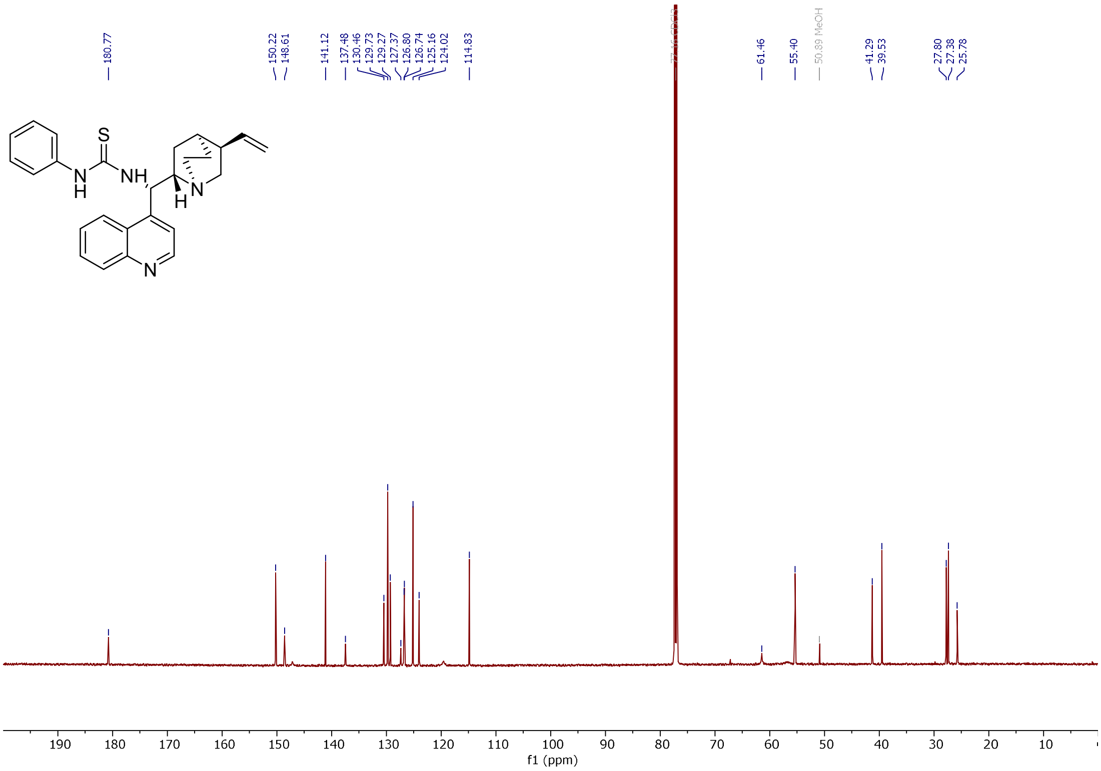

6.4.5 ^{1}H NMR Spectrum of Compound (*rac*)-17 (500 MHz, $CDCl_3$)

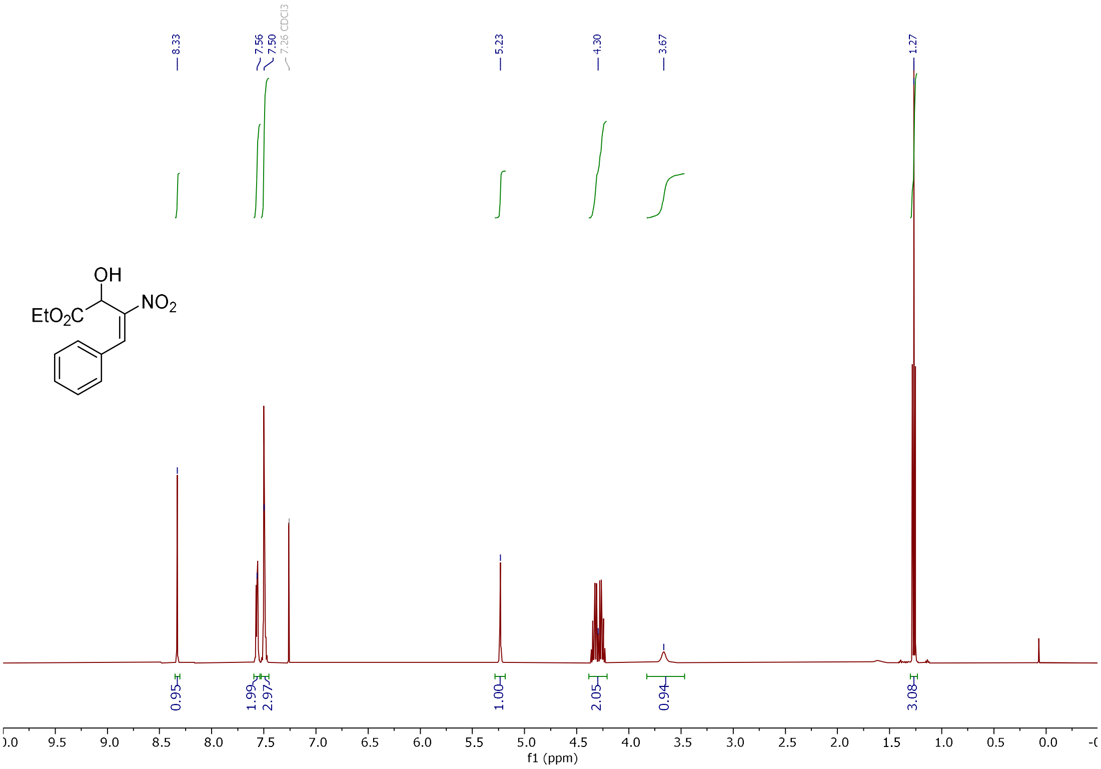

6.4.6 ^{13}C NMR Spectrum of Compound (*rac*)-17 (150 MHz, $CDCl_3$)

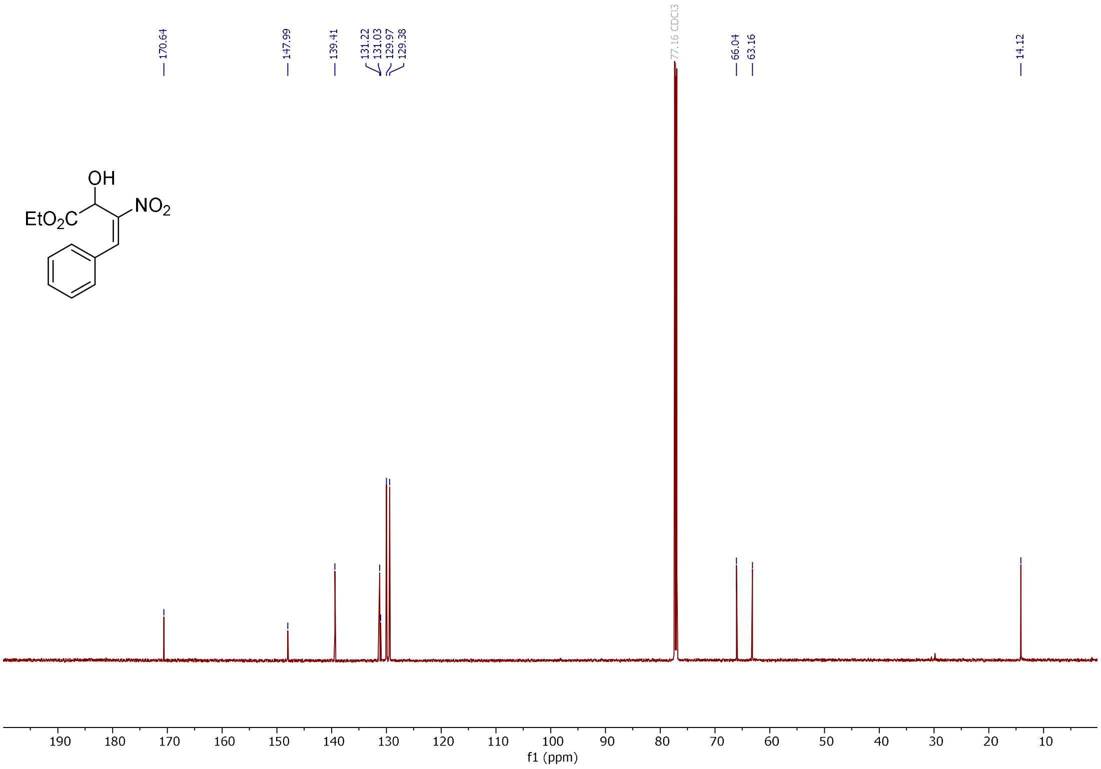

6.4.7 ^{1}H NMR Spectrum of Compound (*rac*)-19 (500 MHz, $CDCl_3$)

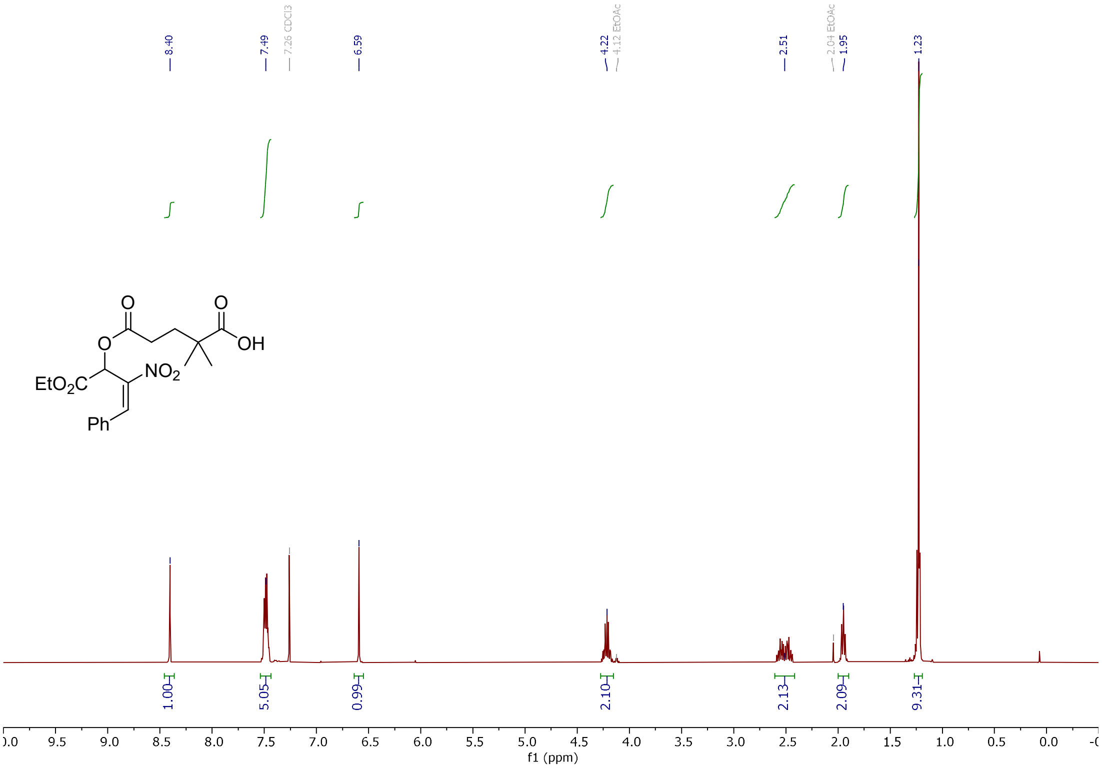

6.4.8 ^{13}C NMR Spectrum of Compound (*rac*)-19 (150 MHz, $CDCl_3$)

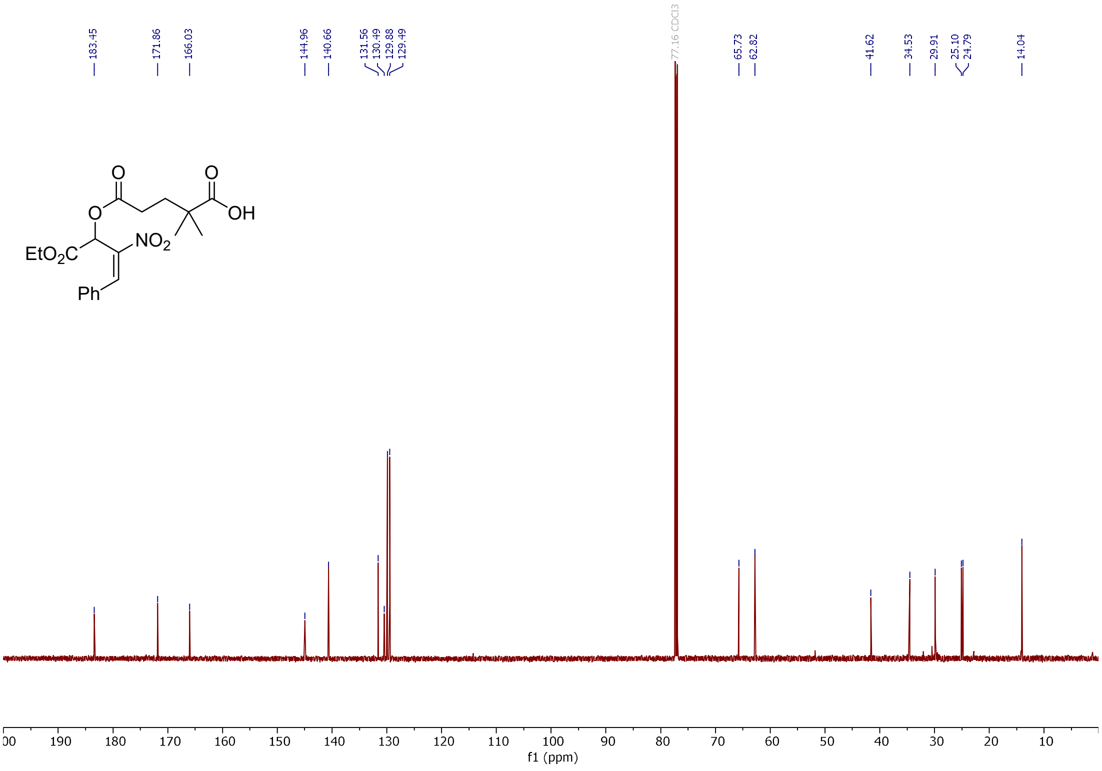

6.4.9 COSY Spectrum of Compound (*rac*)-19

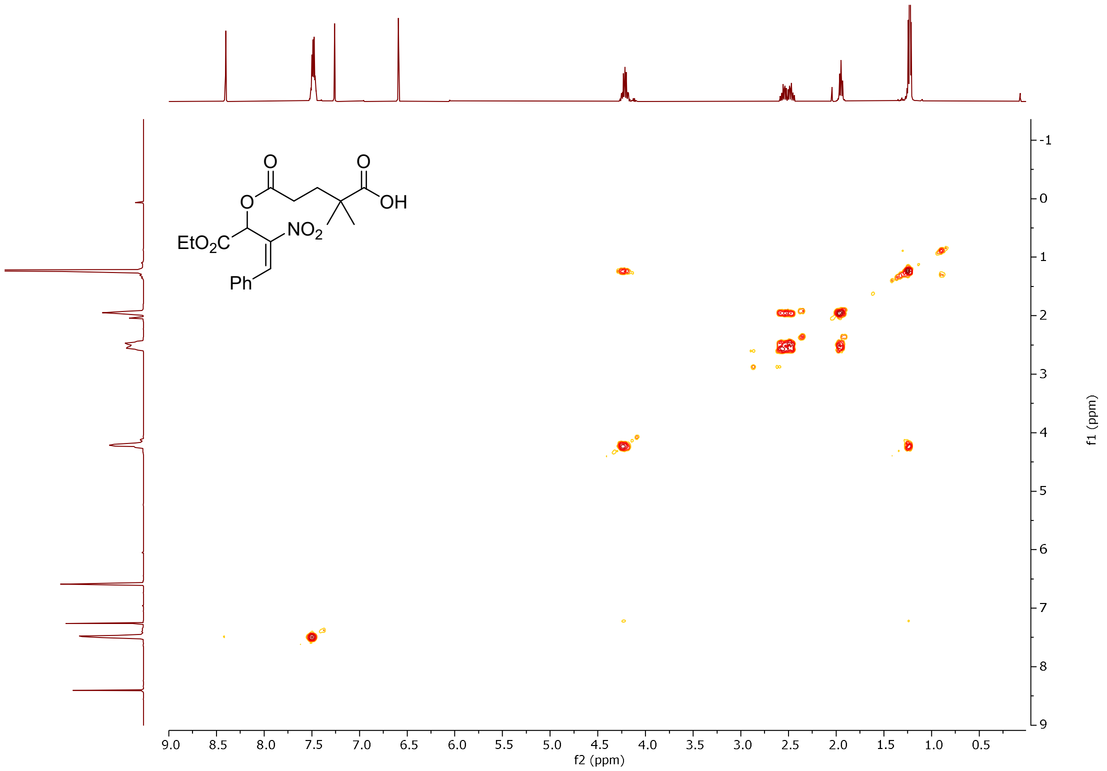

6.4.10 HSQC Spectrum of Compound (*rac*)-19

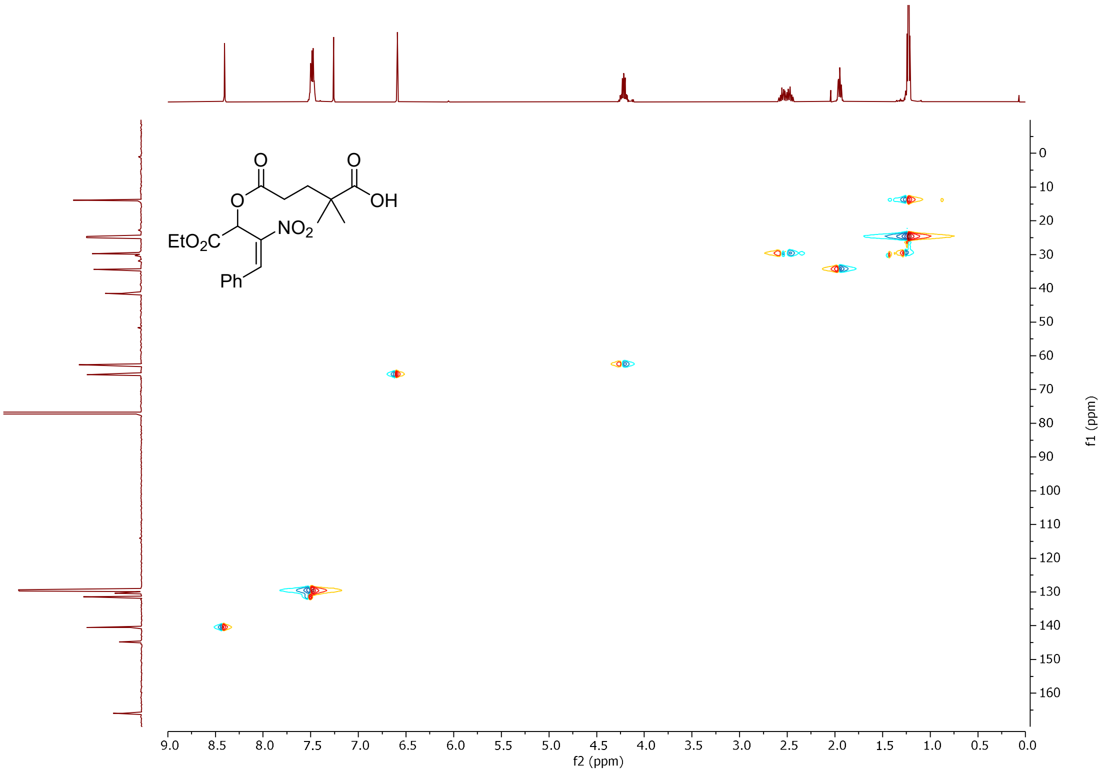

6.4.11 HMBC Spectrum of Compound (*rac*)-19

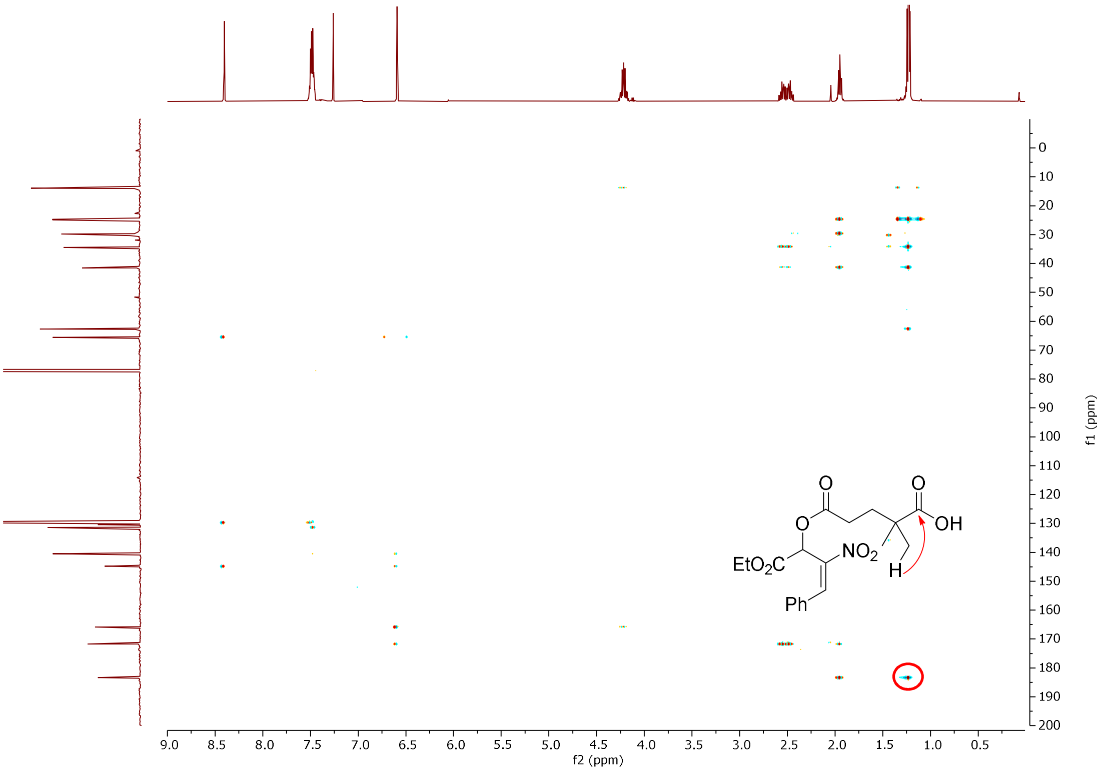

6.4.12 ¹H NMR Spectrum of the 1:1 mixture of diastereomers of Compound (*rac*)-20 (600 MHz, $CDCl_3$)

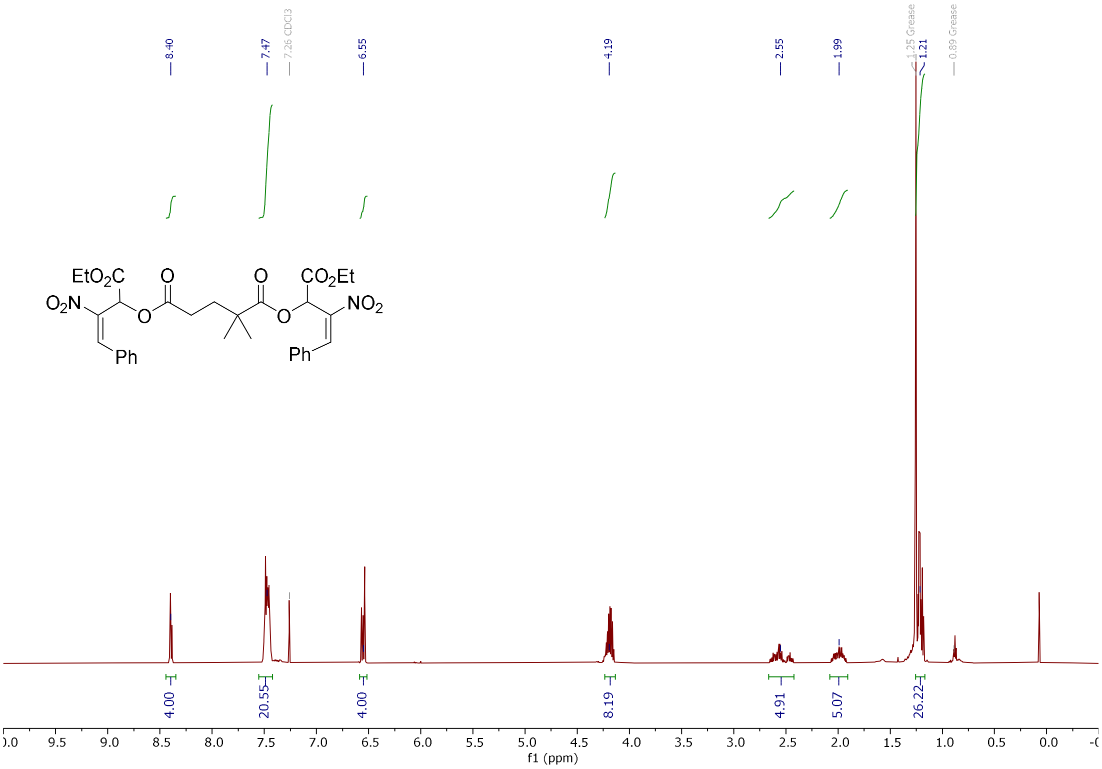

6.4.13 ¹³C NMR Spectrum of the 1:1 mixture of diastereomers of Compound (*rac*)-20 (150 MHz, $CDCl_3$)

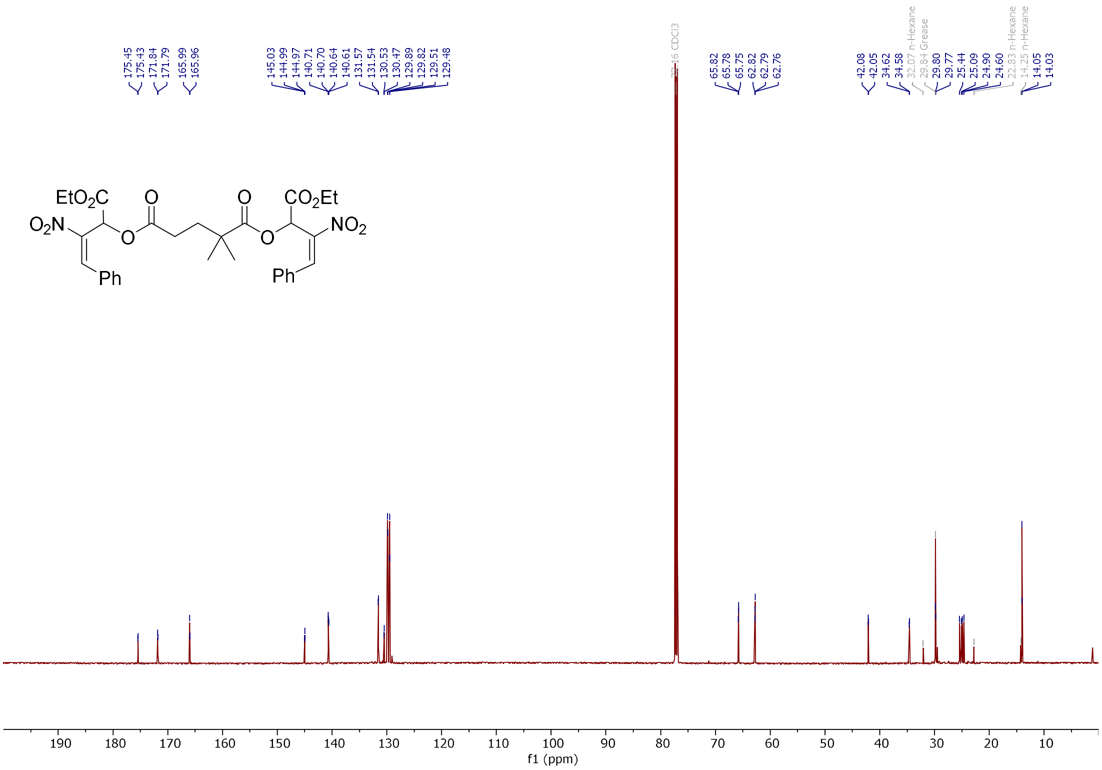

6.4.14 COSY Spectrum of the 1:1 mixture of diastereomers of Compound (*rac*)-20

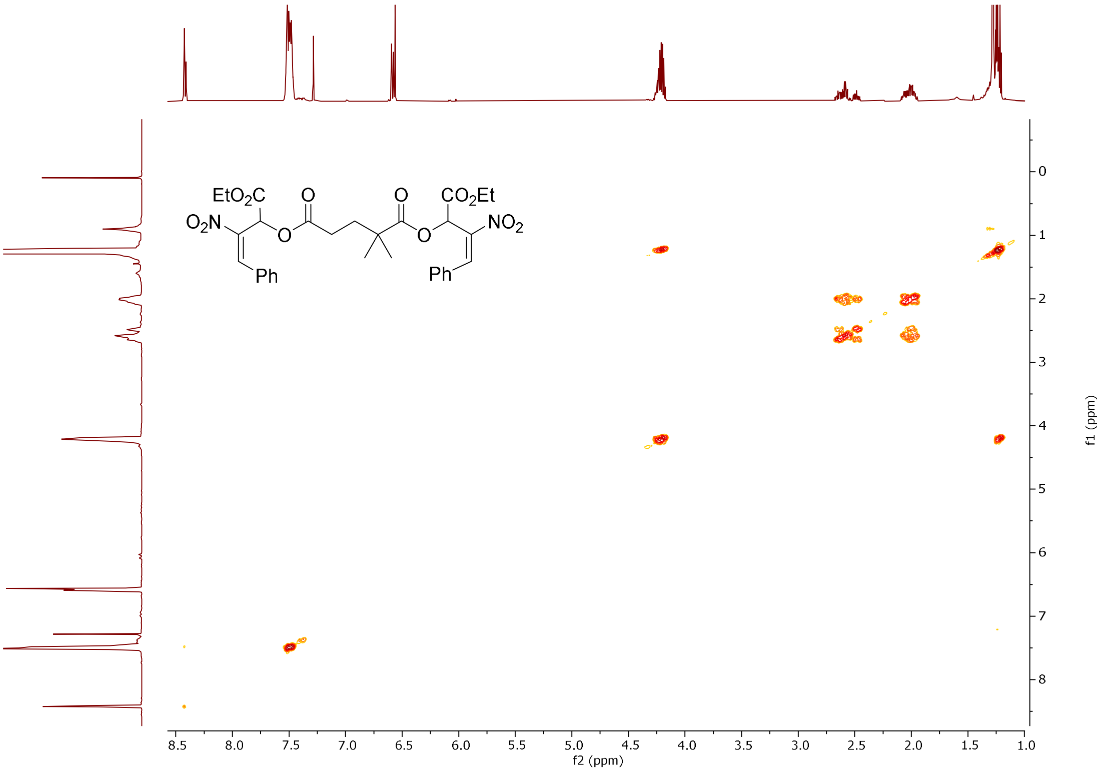

6.4.15 HSQC Spectrum of the 1:1 mixture of diastereomers of Compound (*rac*)-20

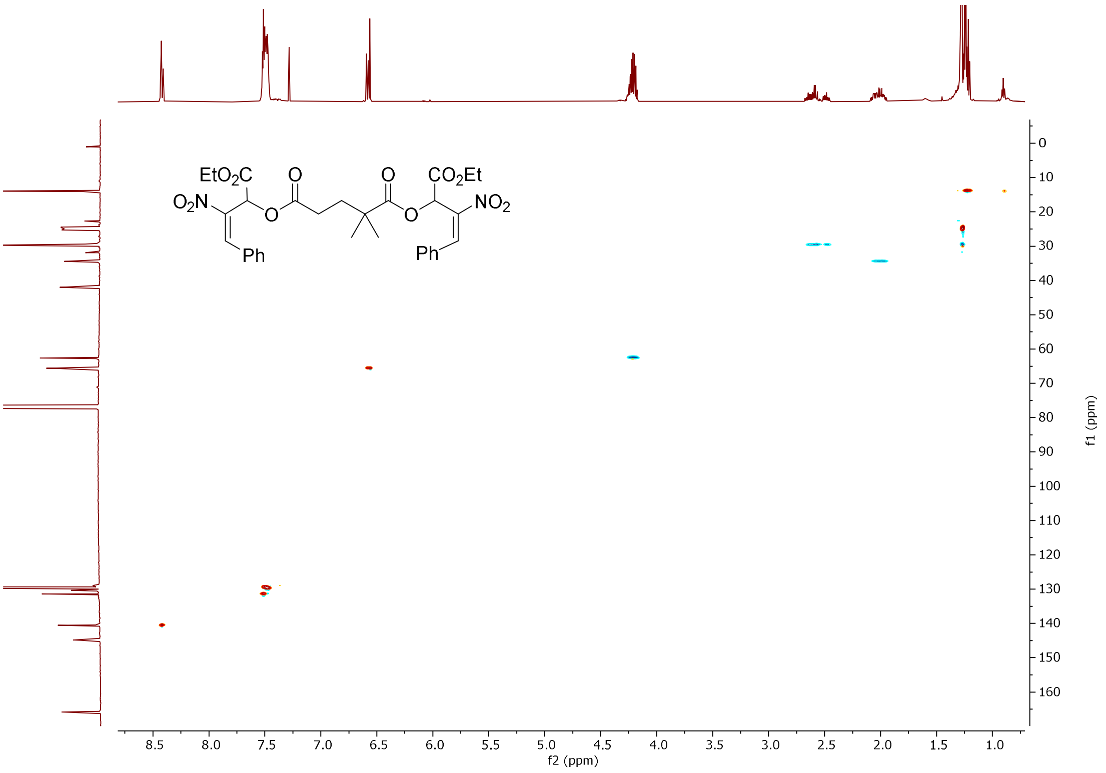

6.4.16 HMBC Spectrum of the 1:1 mixture of diastereomers of Compound (*rac*)-20

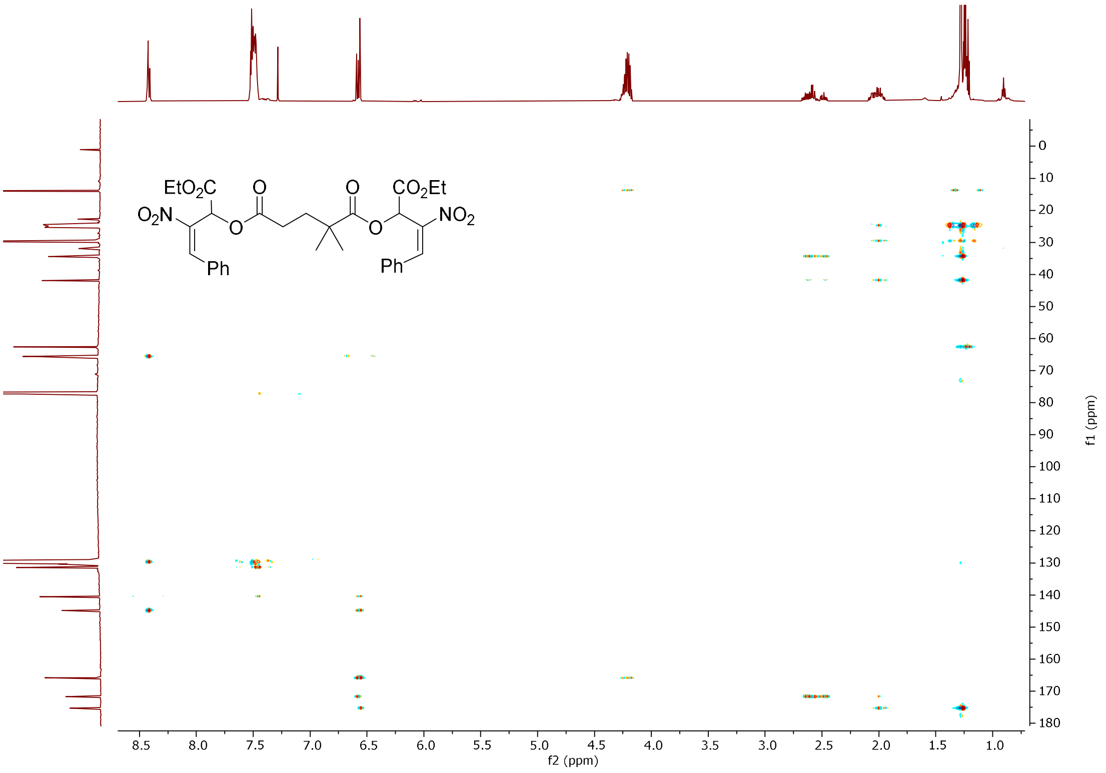

6.4.17 ^{1}H NMR Spectrum of Compound S14h (500 MHz, $CDCl_3$)

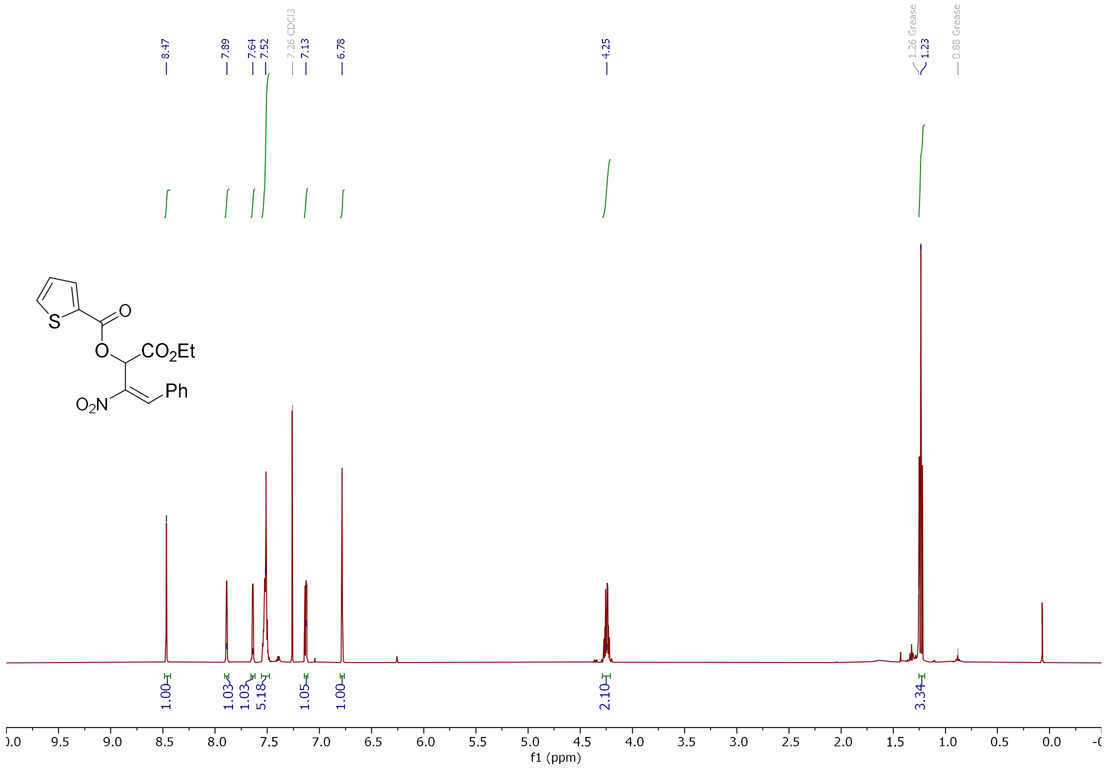

6.4.18 ^{13}C NMR Spectrum of Compound S14h (125 MHz, $CDCl_3$)

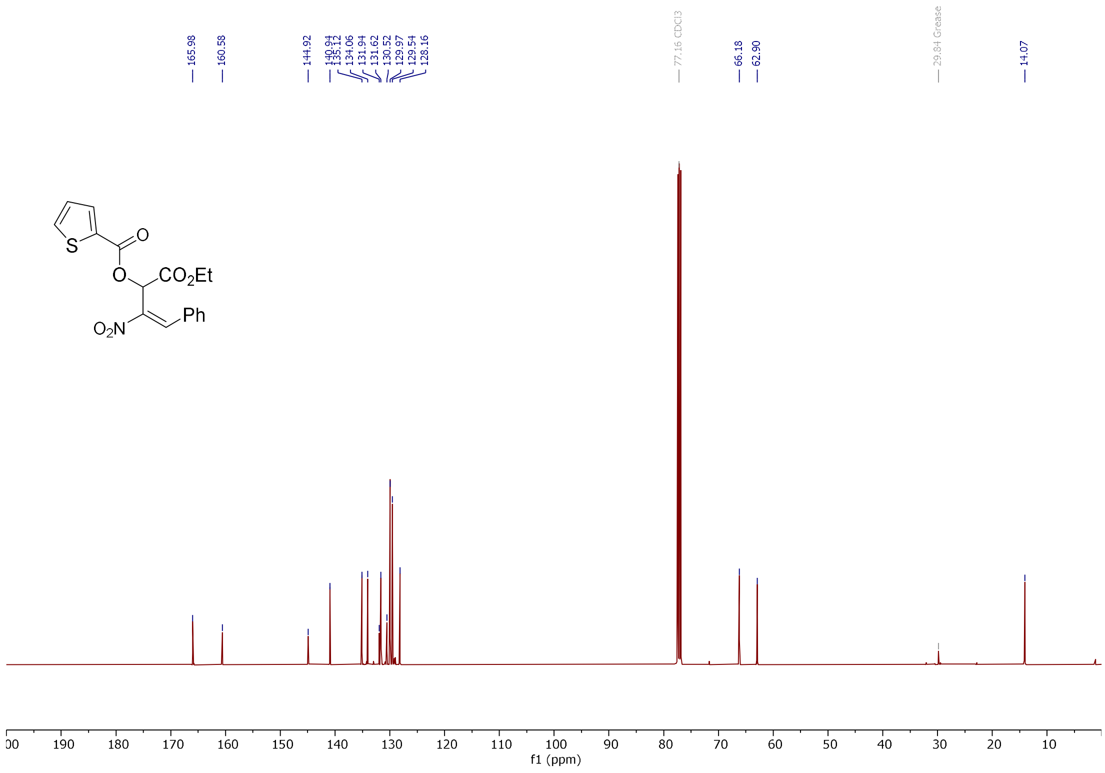

6.4.19 HSQC Spectrum of Compound S14h

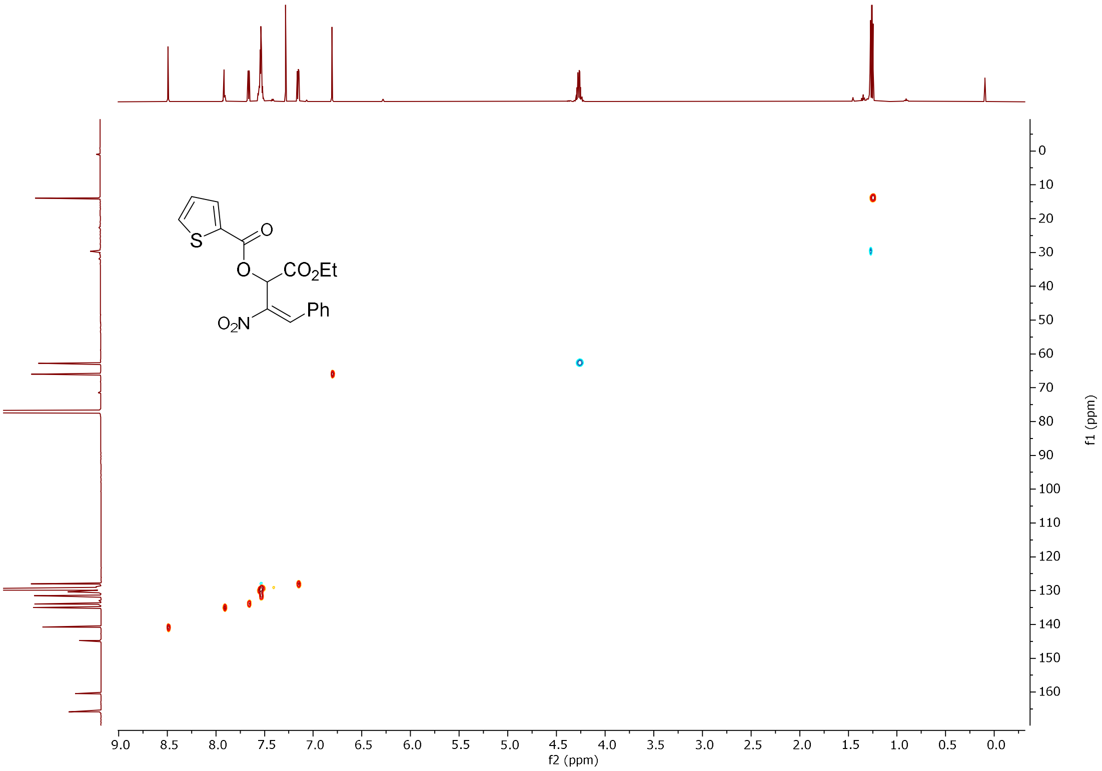

6.5. Organocatalytic Stereoretentive Diastereoconvergent Reaction

6.5.1 ^{1}H NMR Spectrum of Compound ((*R,S,S*)-25) (500 MHz, $CDCl_3$)

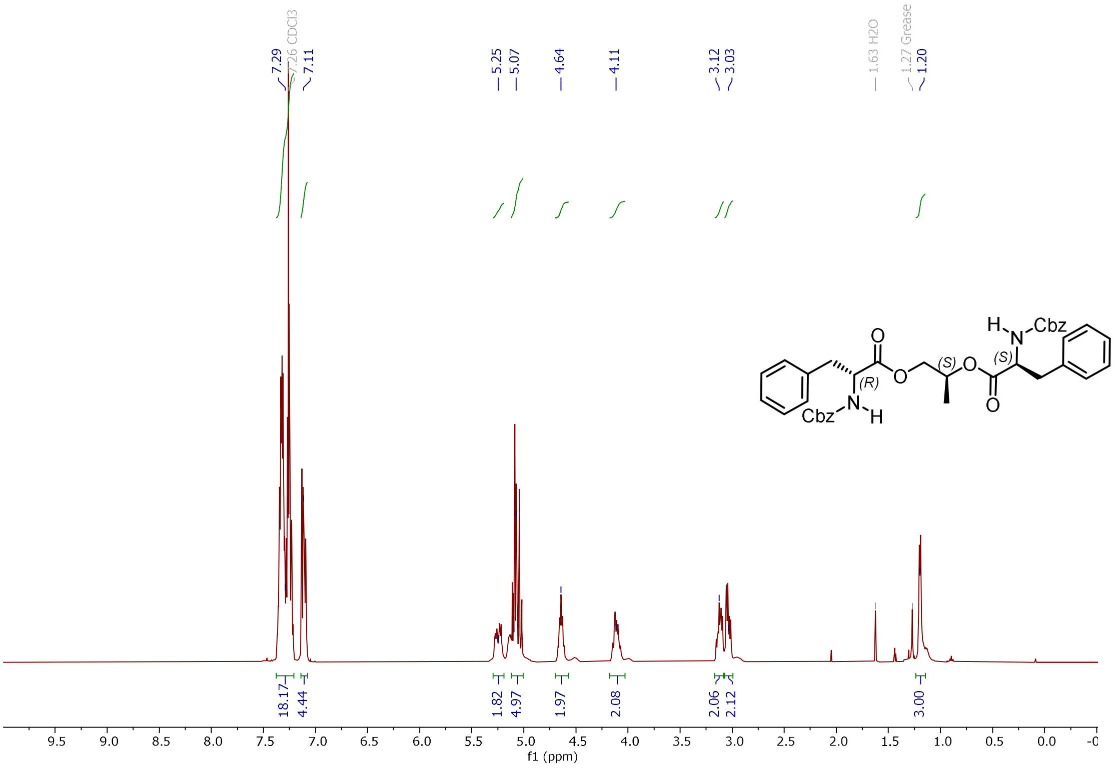

6.5.2 ^{13}C NMR Spectrum of Compound ((*R,S,S*)-25) (125 MHz, $CDCl_3$)

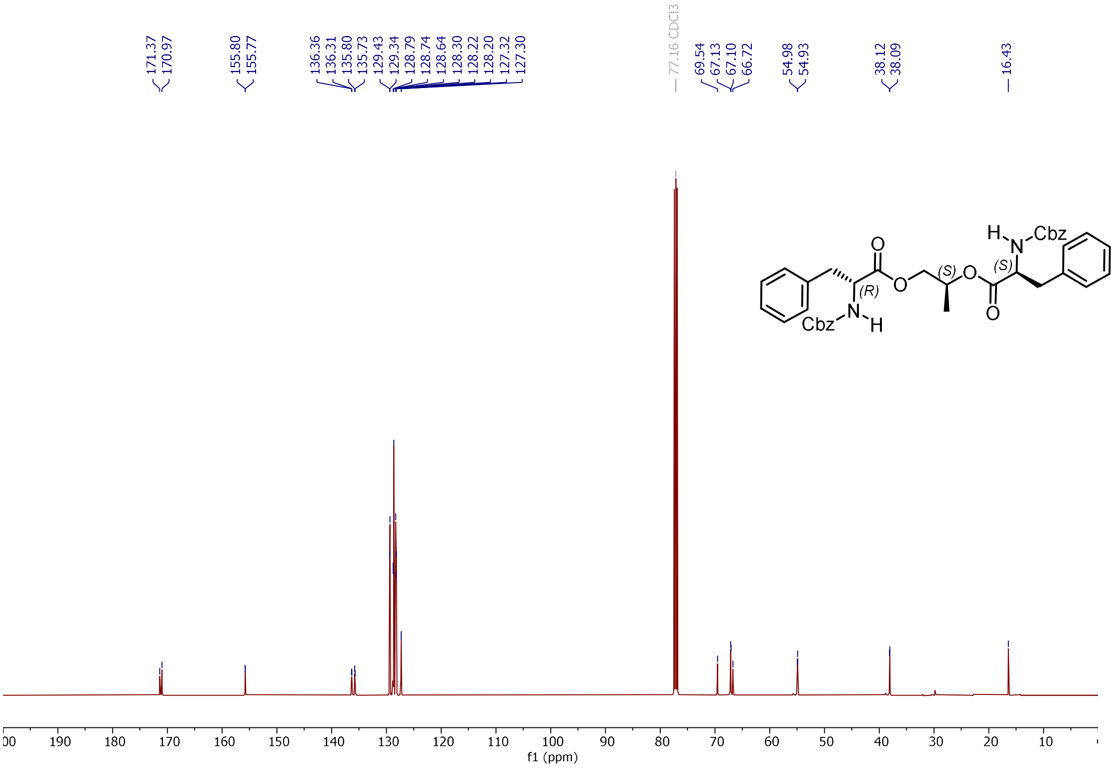

6.5.3 ^{1}H NMR Spectrum of Compound ((*S*,*S*,*R*)-25) (500 MHz, $CDCl_3$)

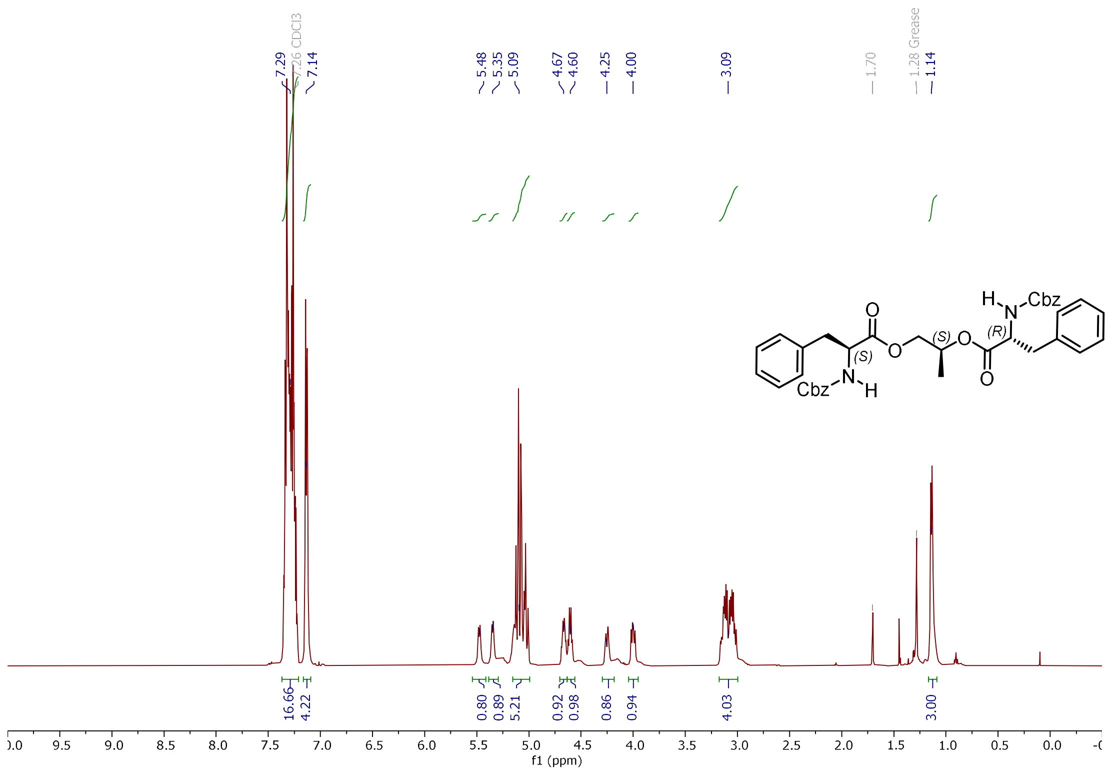

6.5.4 ^{13}C NMR Spectrum of Compound ((*S*,*S*,*R*)-25) (125 MHz, $CDCl_3$)

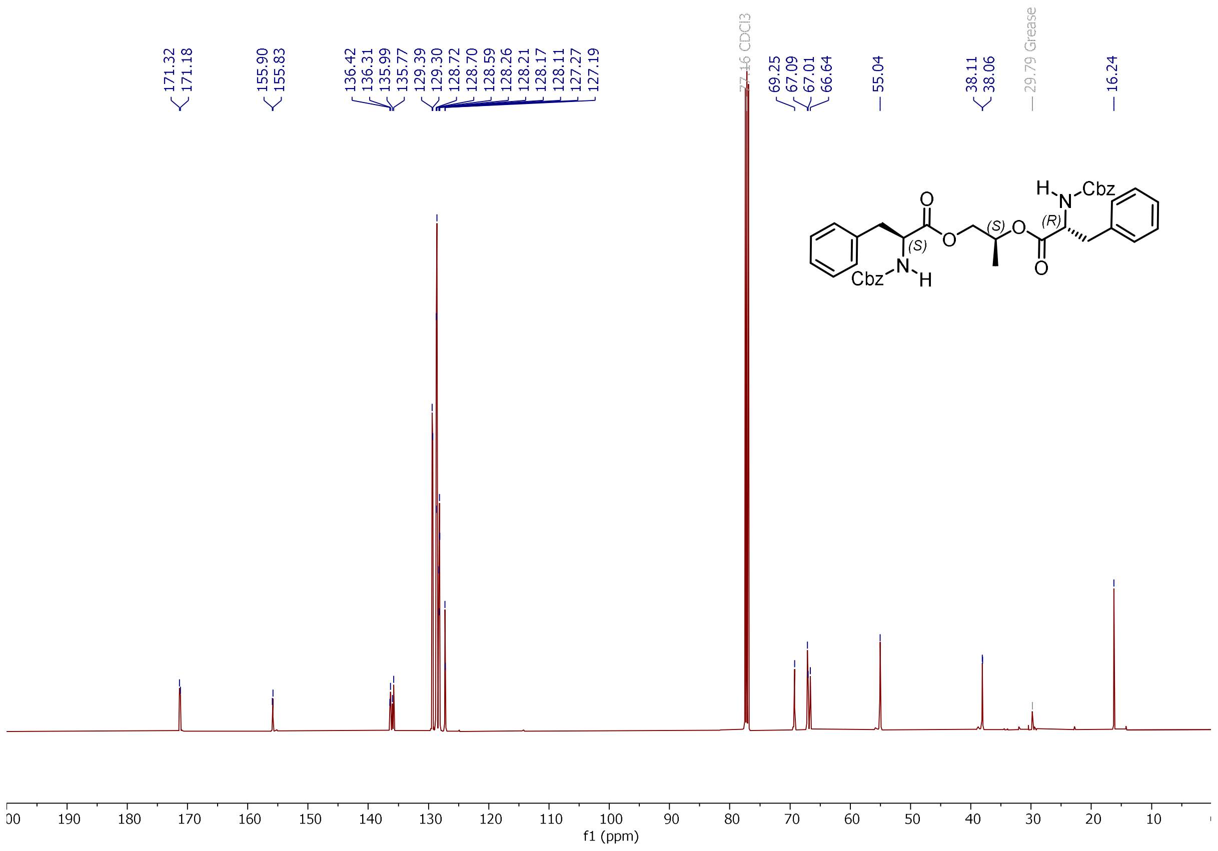

6.5.5 ^{1}H NMR Spectrum of Compound ((*S*,*S*,*S*)-25) (500 MHz, $CDCl_3$)

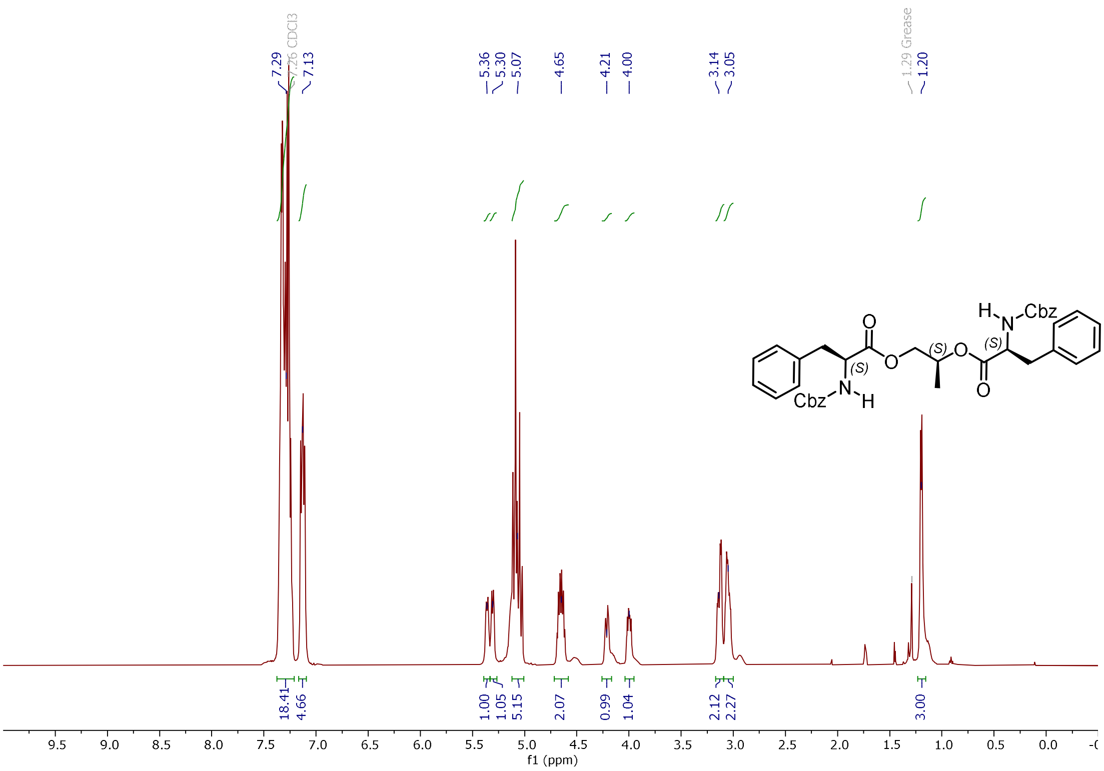

6.5.6 ^{13}C NMR Spectrum of Compound ((*S*,*S*,*R*)-25) (125 MHz, $CDCl_3$)

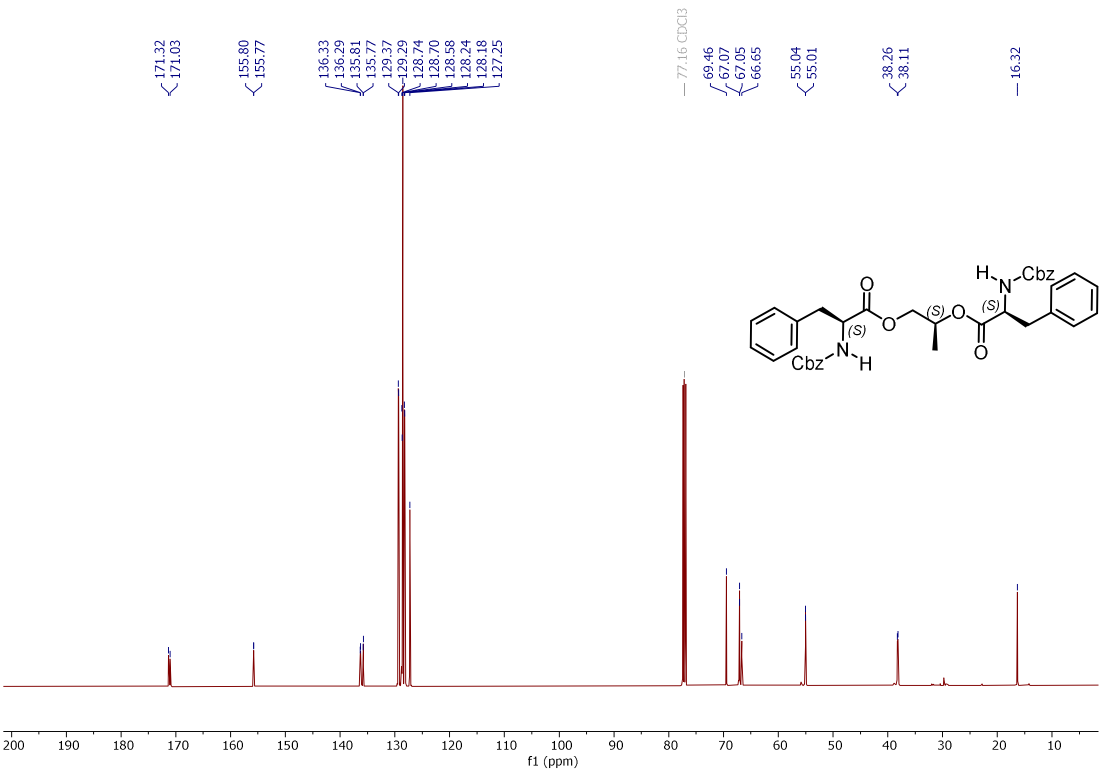

6.5.7 ^{1}H NMR Spectrum of Compound ((*R*,*S*,*R*)-25) (500 MHz, $CDCl_3$)

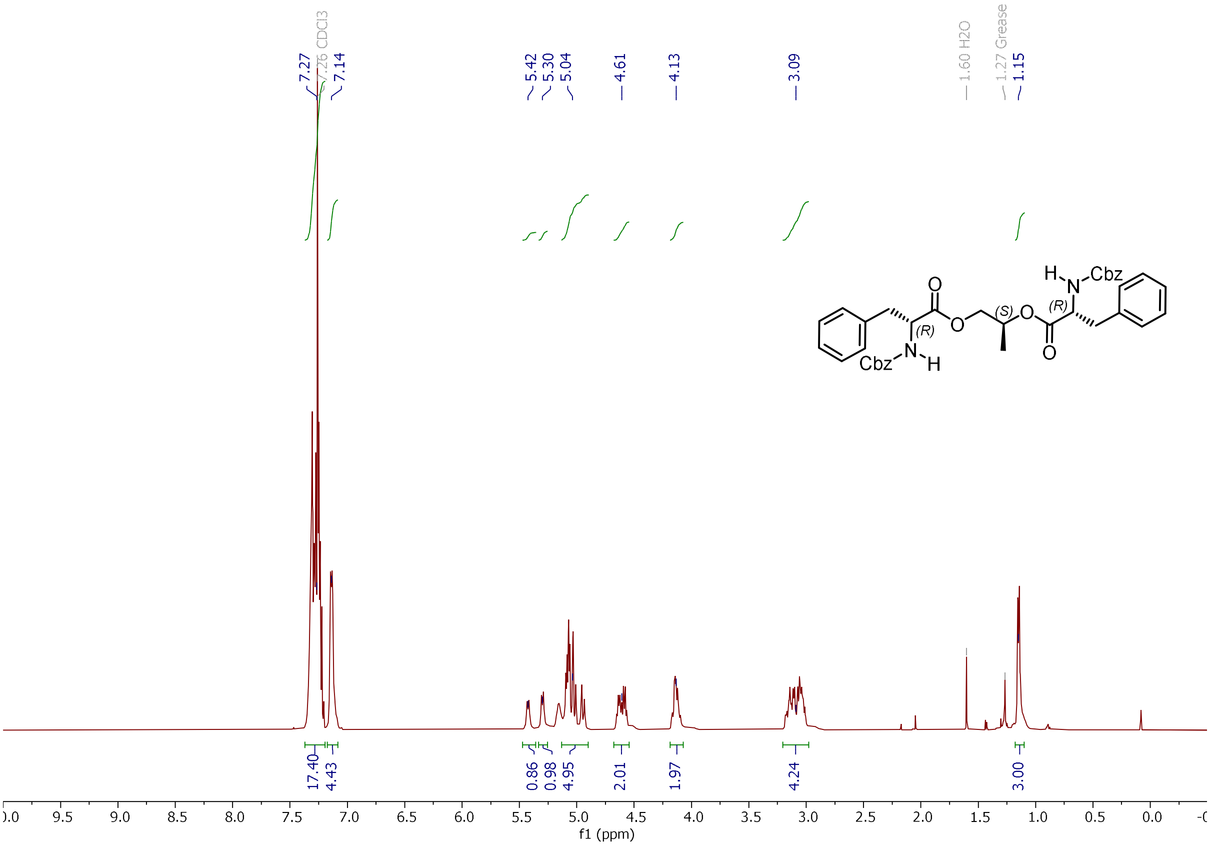

6.5.8 ^{13}C NMR Spectrum of Compound ((*R*,*S*,*R*)-25) (125 MHz, $CDCl_3$)

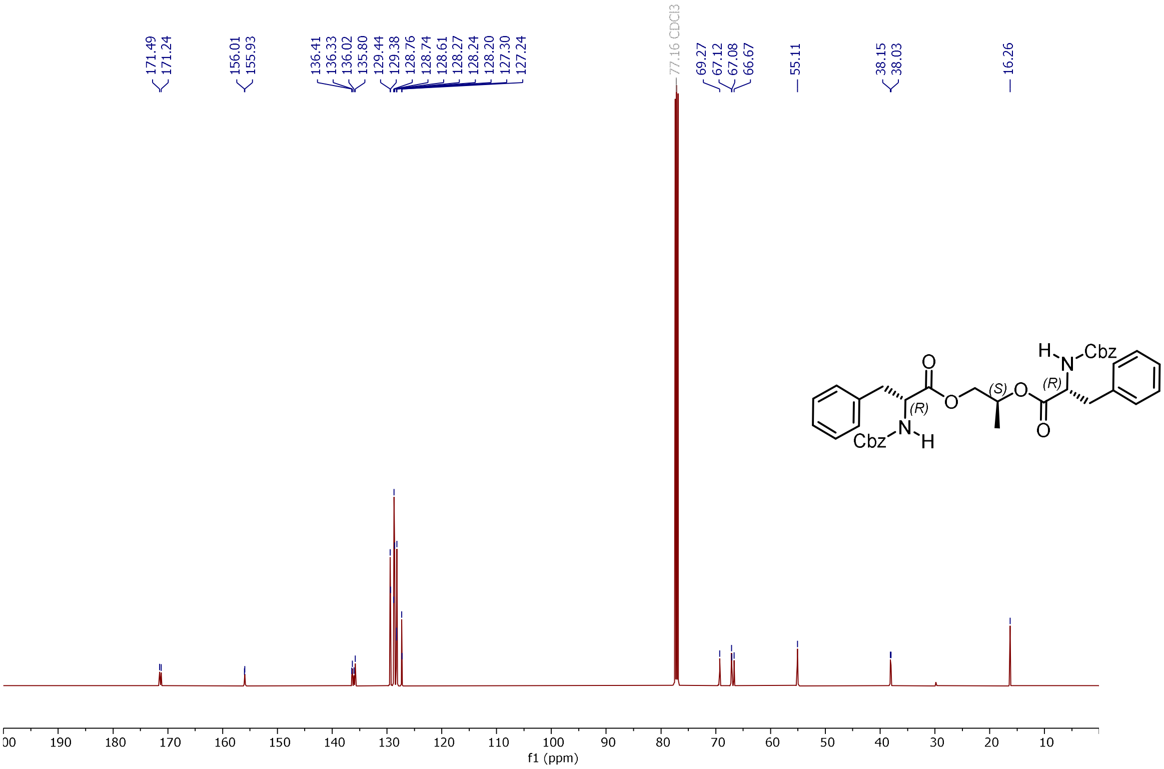

6.5.9 ^{1}H NMR Spectrum of Compound ((*S*,*S*)-S21) (500 MHz, $CDCl_3$)

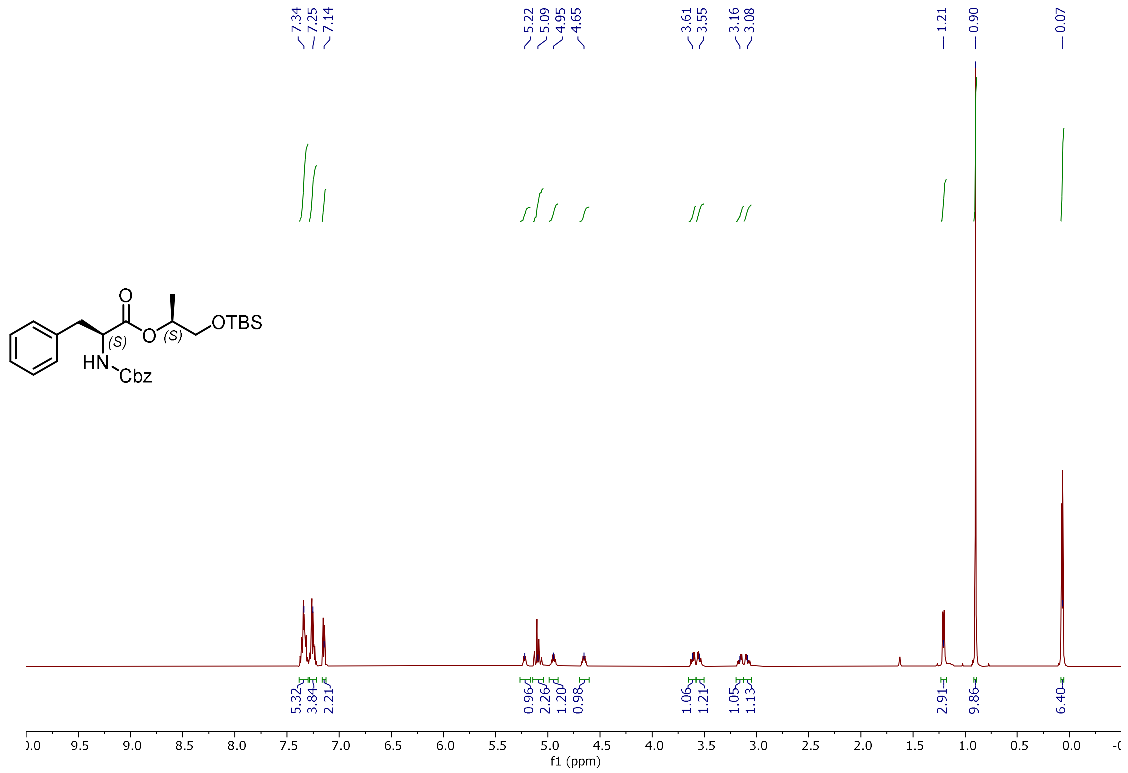

6.5.10 ^{13}C NMR Spectrum of Compound ((*S*,*S*)-S21) (125 MHz, $CDCl_3$)

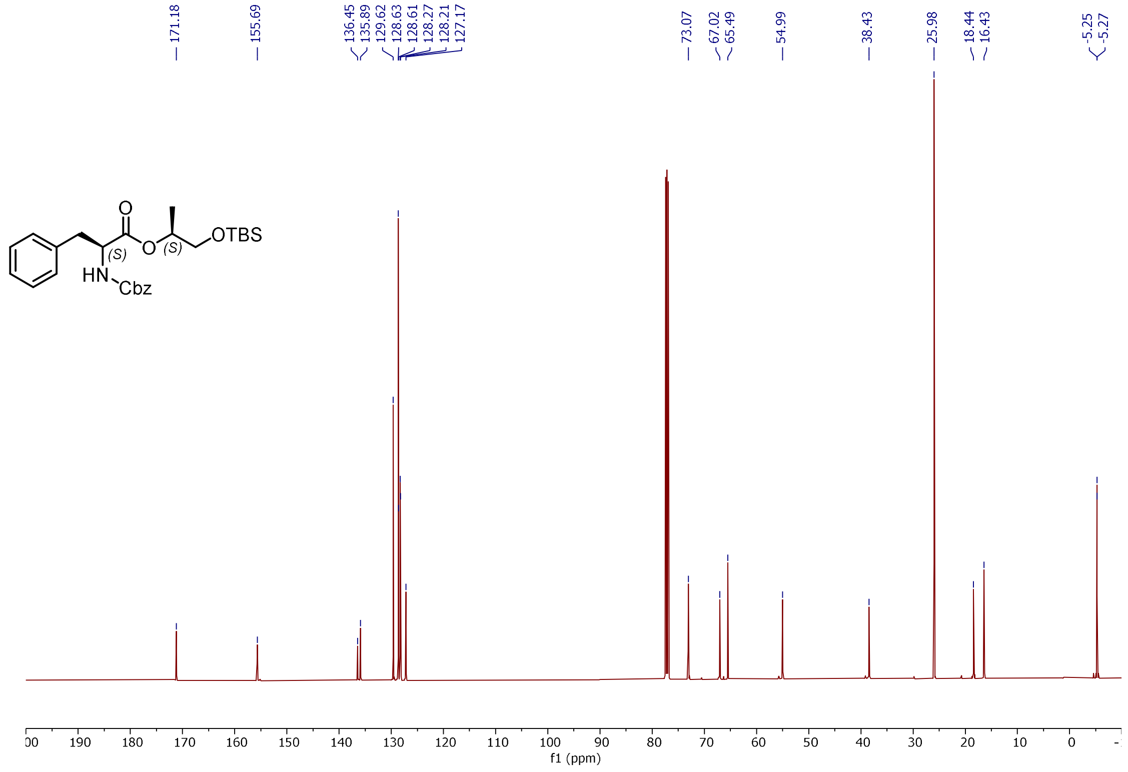

6.5.11 ^{1}H NMR Spectrum of Compound ((*R*,*S*)-S21) (500 MHz, CDCl$_3$)

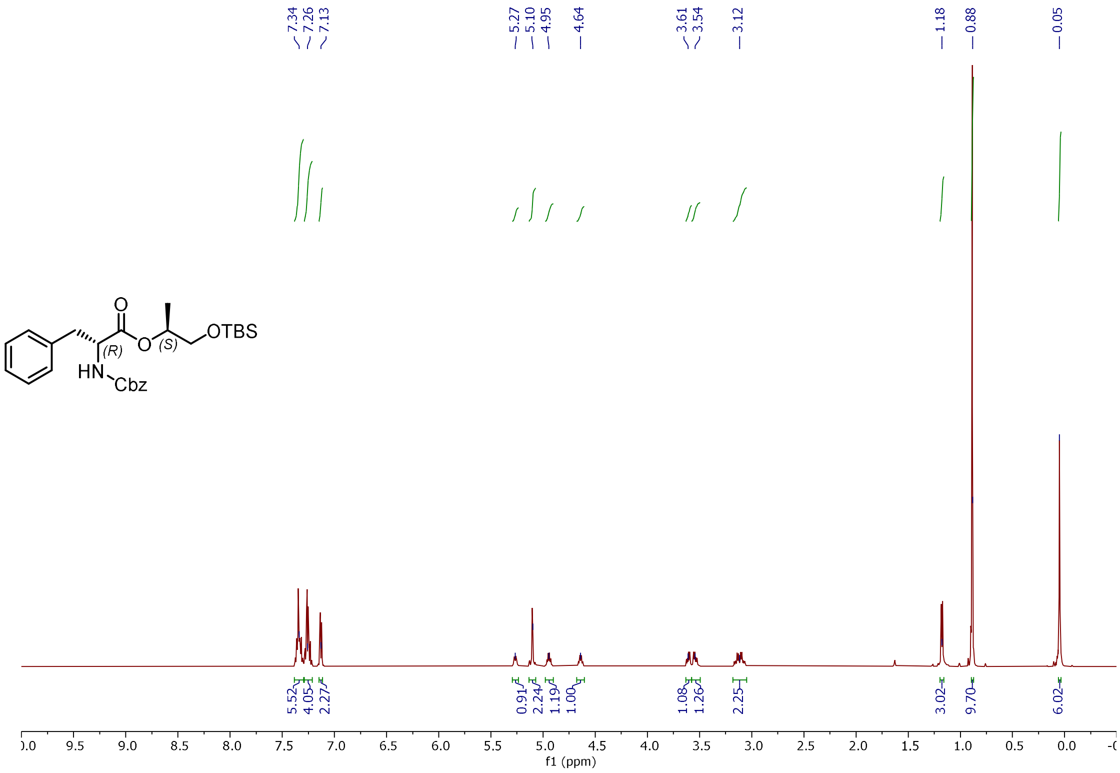

6.5.12 ^{13}C NMR Spectrum of Compound ((*R*,*S*)-S21) (125 MHz, CDCl$_3$)

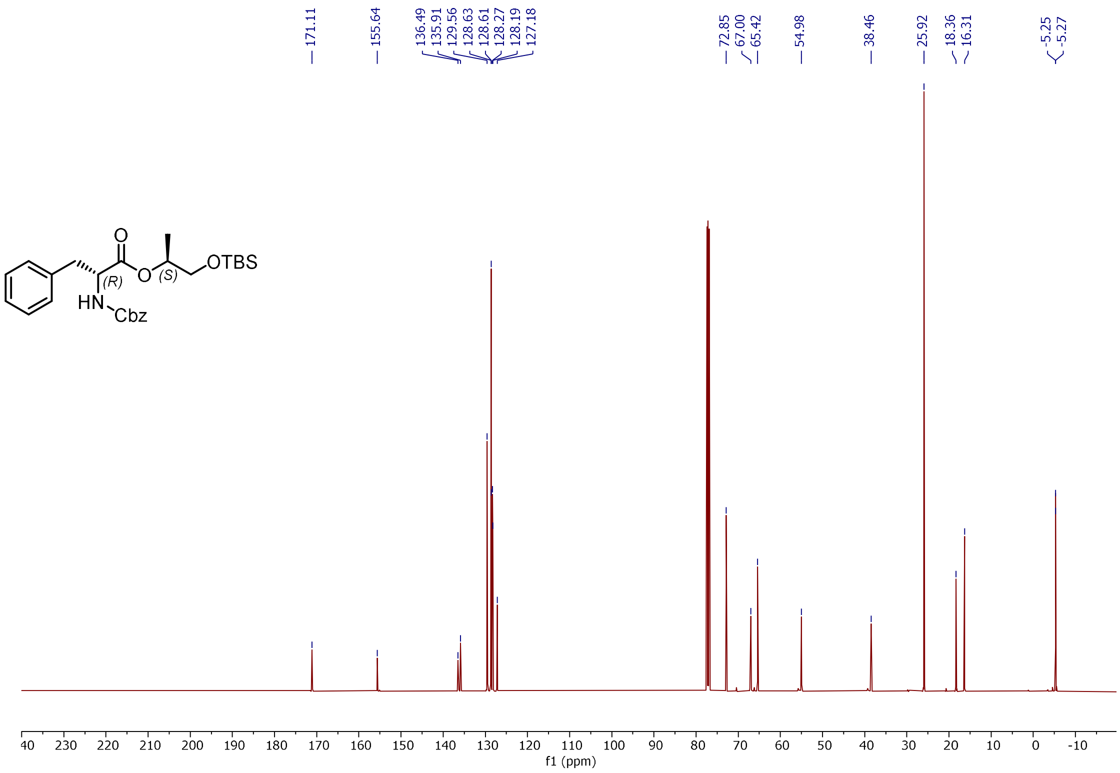

6.5.13 ^{1}H NMR Spectrum of Compound ((*R*,*S*)-S17) (500 MHz, $CDCl_3$) (*crude*):

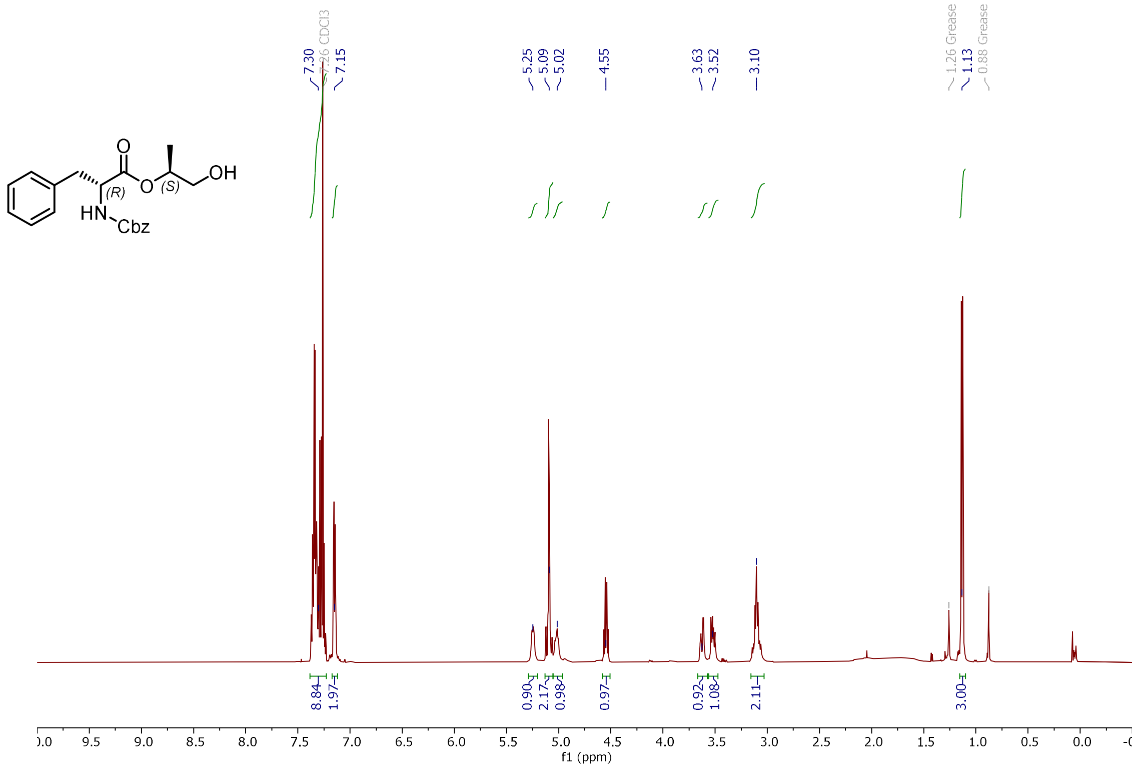

6.5.14 ^{13}C NMR Spectrum of Compound ((*R*,*S*)-S17) (125 MHz, $CDCl_3$)

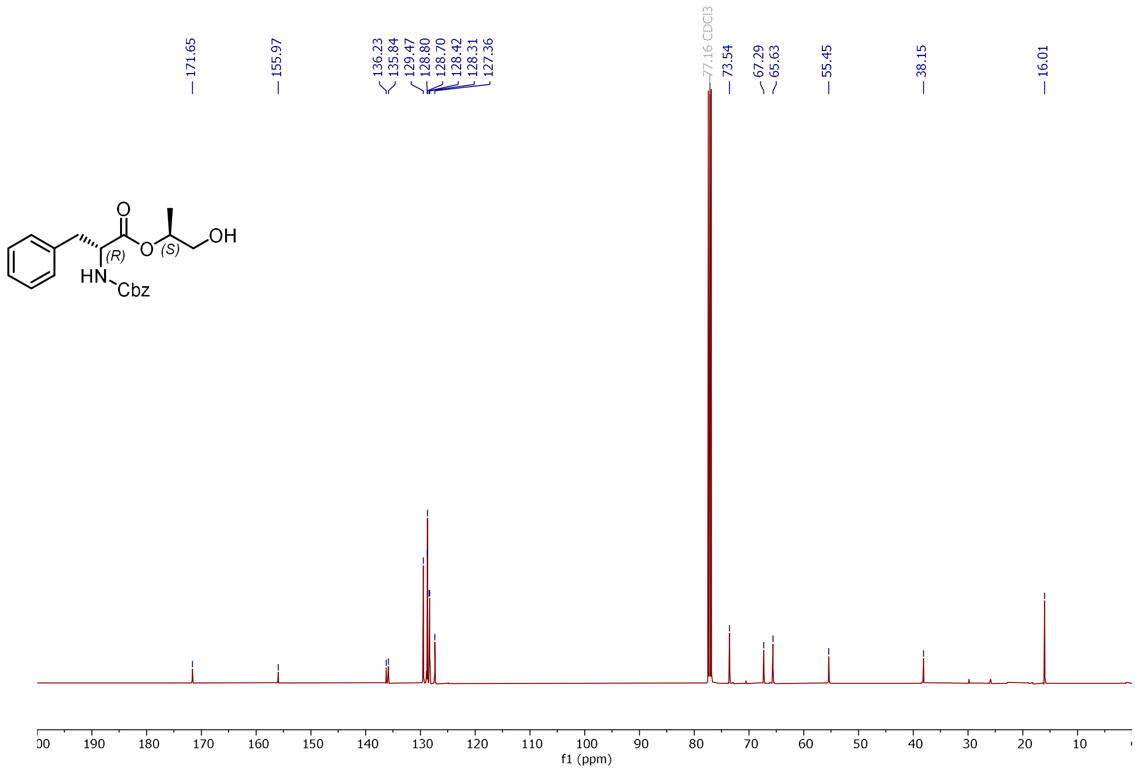

6.5.15 ^{1}H NMR Spectrum of Compound ((*S,S*)-S17) (500 MHz, $CDCl_3$) (*crude*)

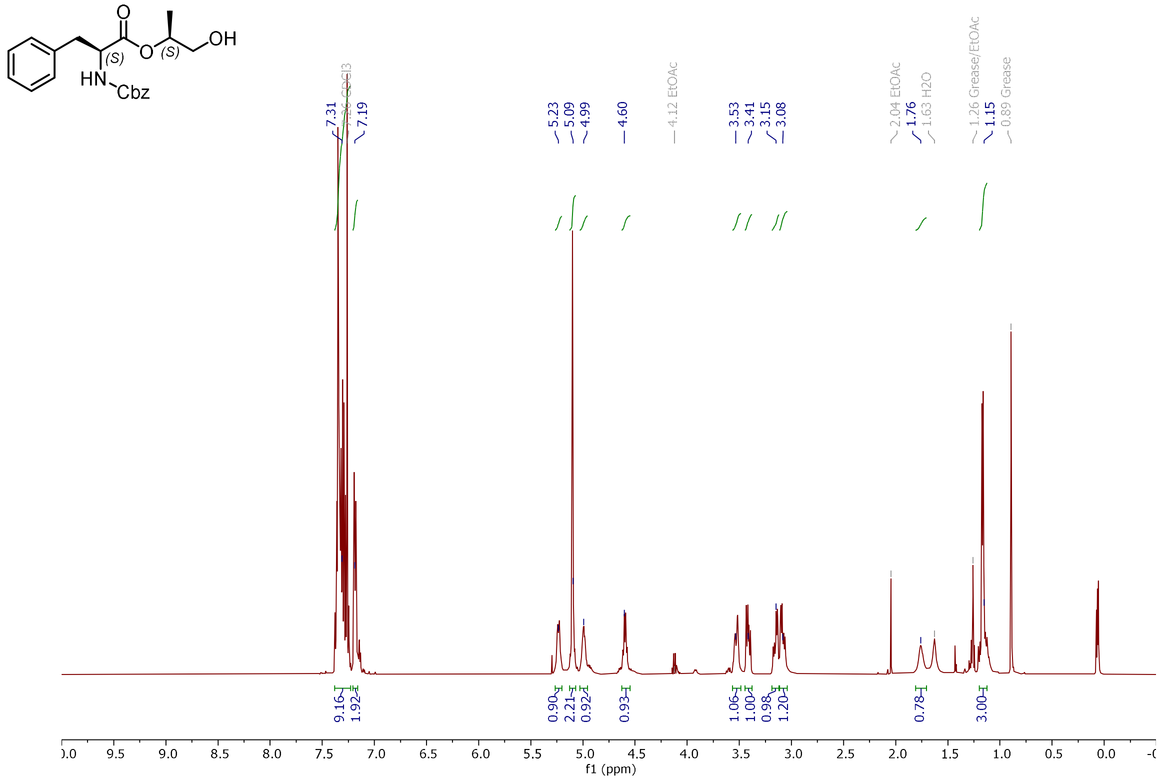

6.5.16 ^{13}C NMR Spectrum of Compound ((*S,S*)-S17) (125 MHz, $CDCl_3$)

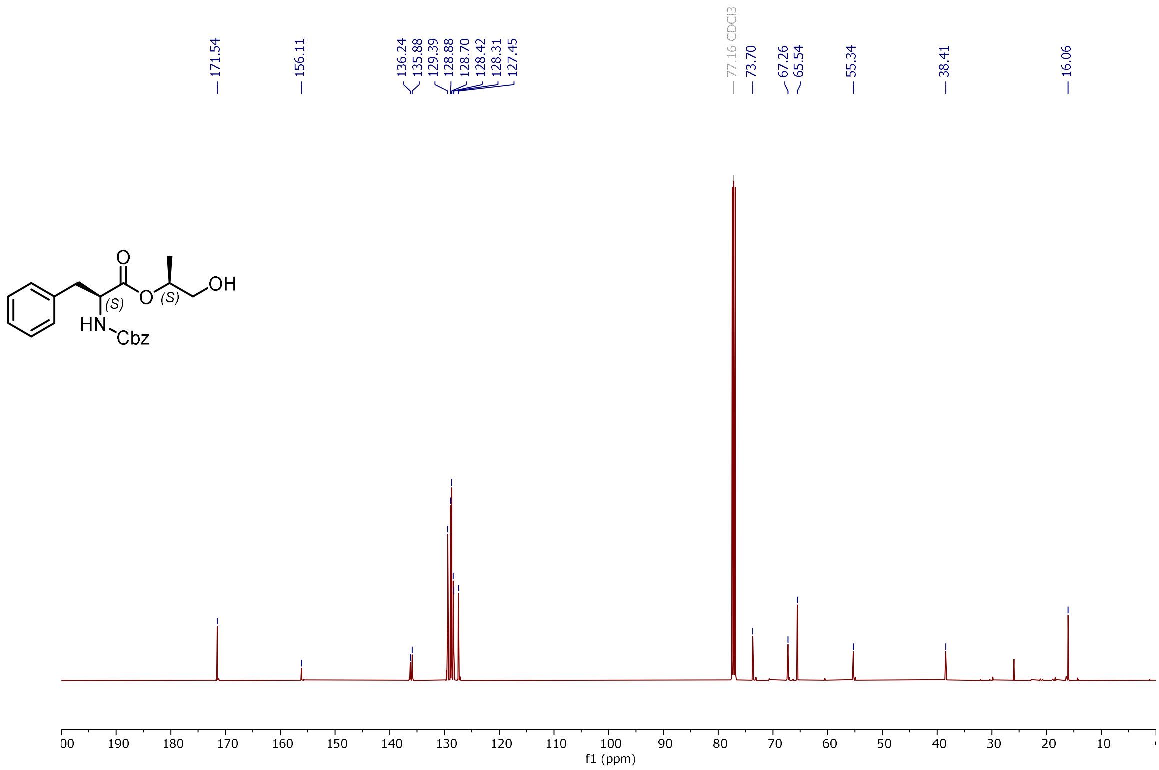

6.5.17 ^{1}H NMR Spectrum of Compound ((*R*,*R*)-S22) (500 MHz, CDCl$_3$)

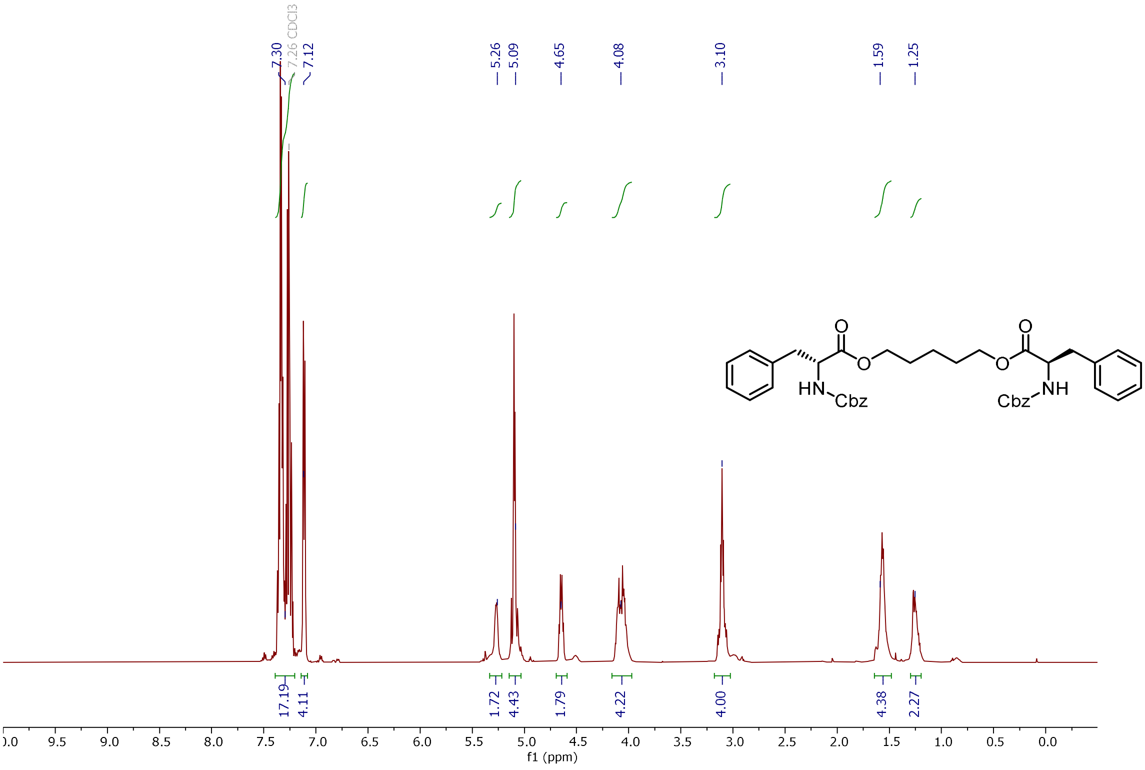

6.5.18 ^{13}C NMR Spectrum of Compound ((*R*,*R*)-S22) (125 MHz, CDCl$_3$)

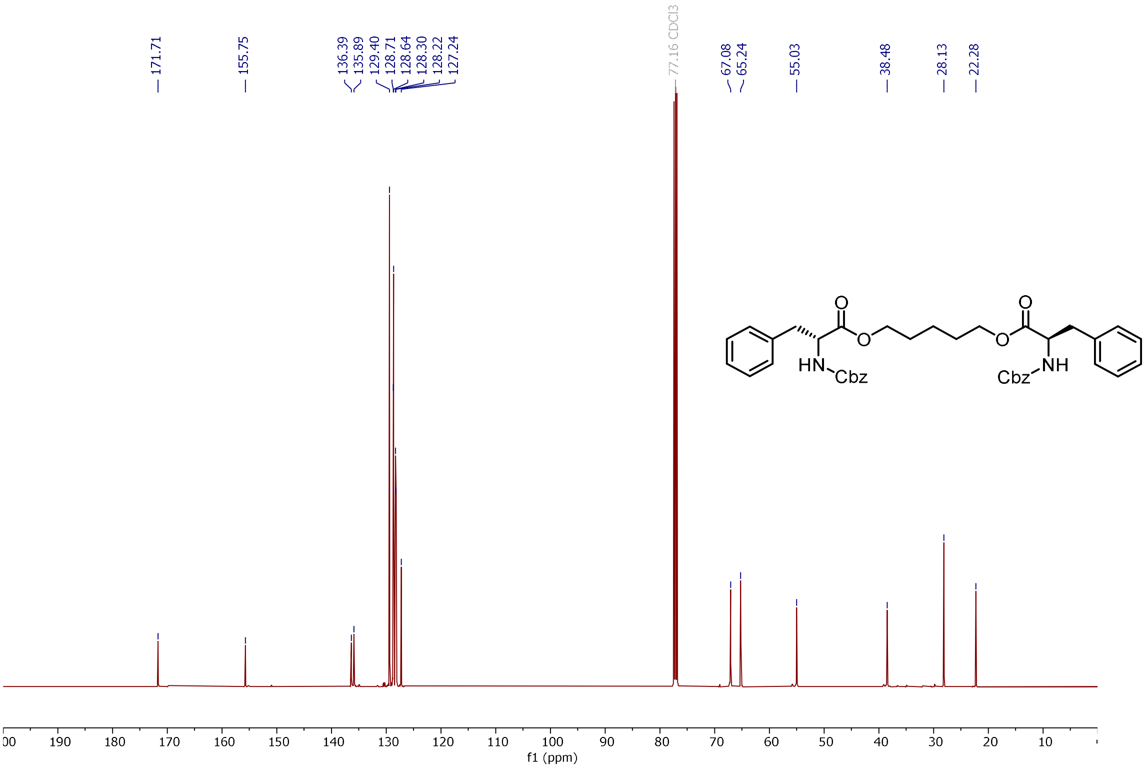

6.5.19 ^{1}H NMR Spectrum of Compound ((*R*,*S*)-S16) (500 MHz, $CDCl_3$)

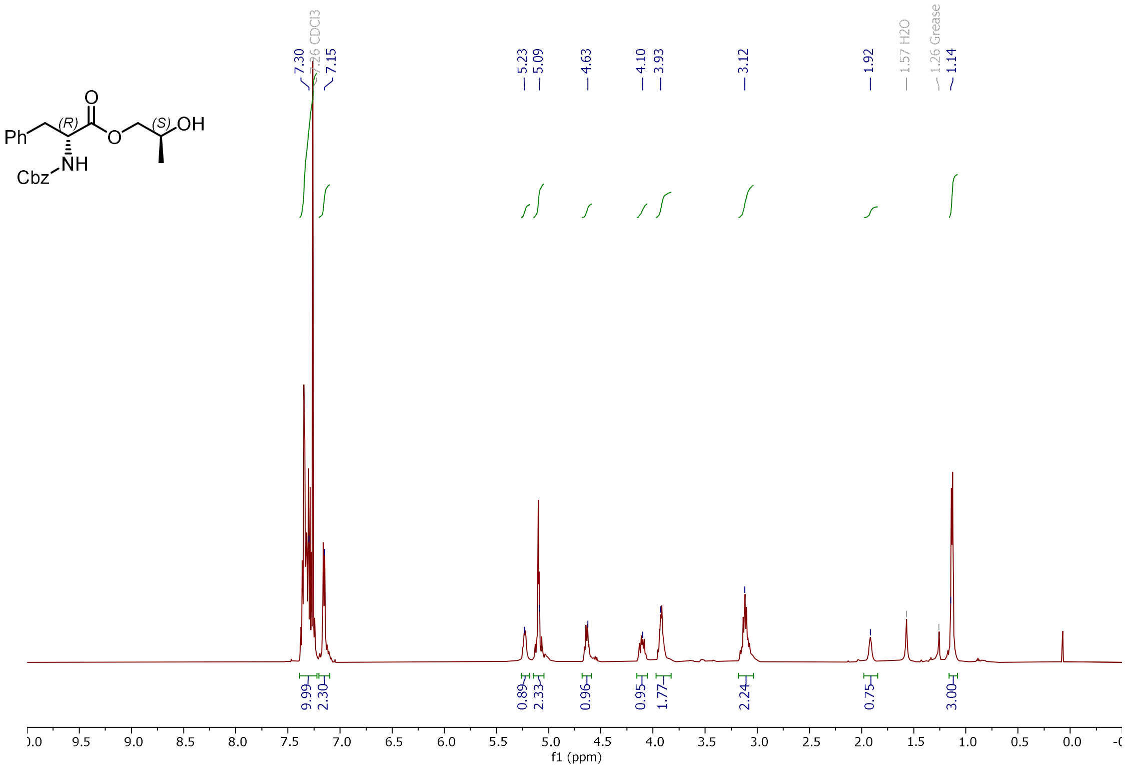

6.5.20 ^{13}C NMR Spectrum of Compound ((*R*,*S*)-S16) (125 MHz, $CDCl_3$)

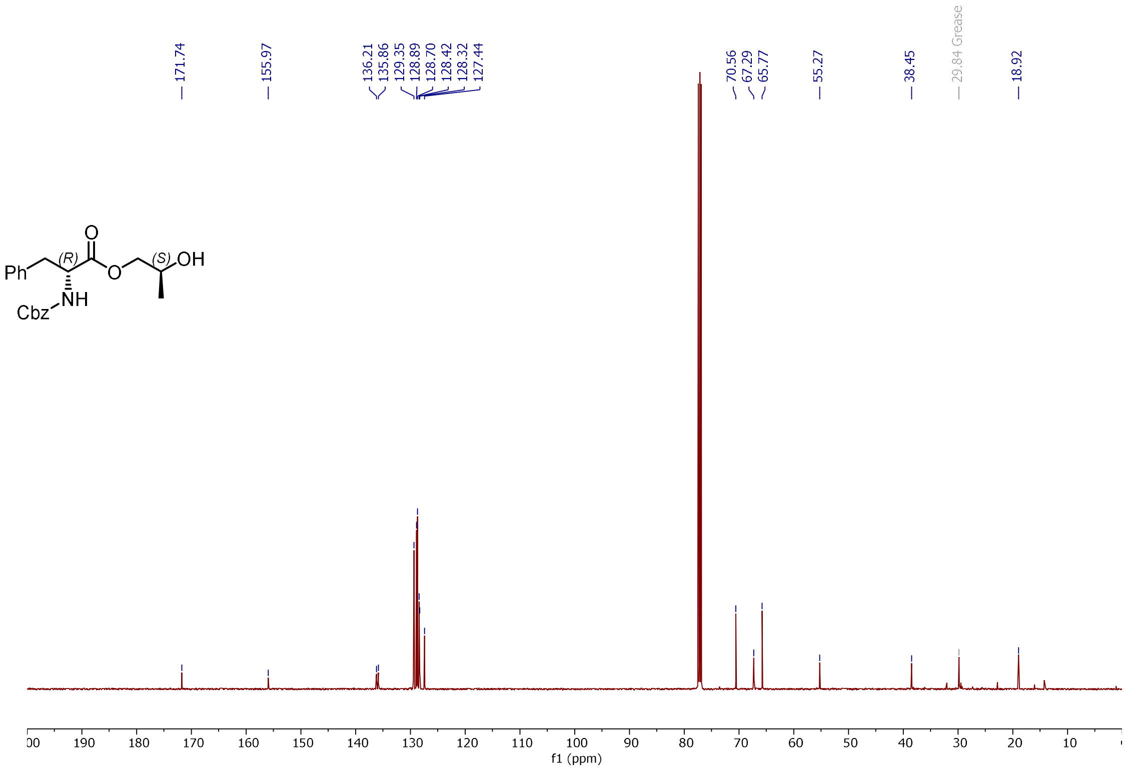

6.5.21 ^{1}H NMR Spectrum of Compound ((*R*,*R*)-S16) (500 MHz, $CDCl_3$)

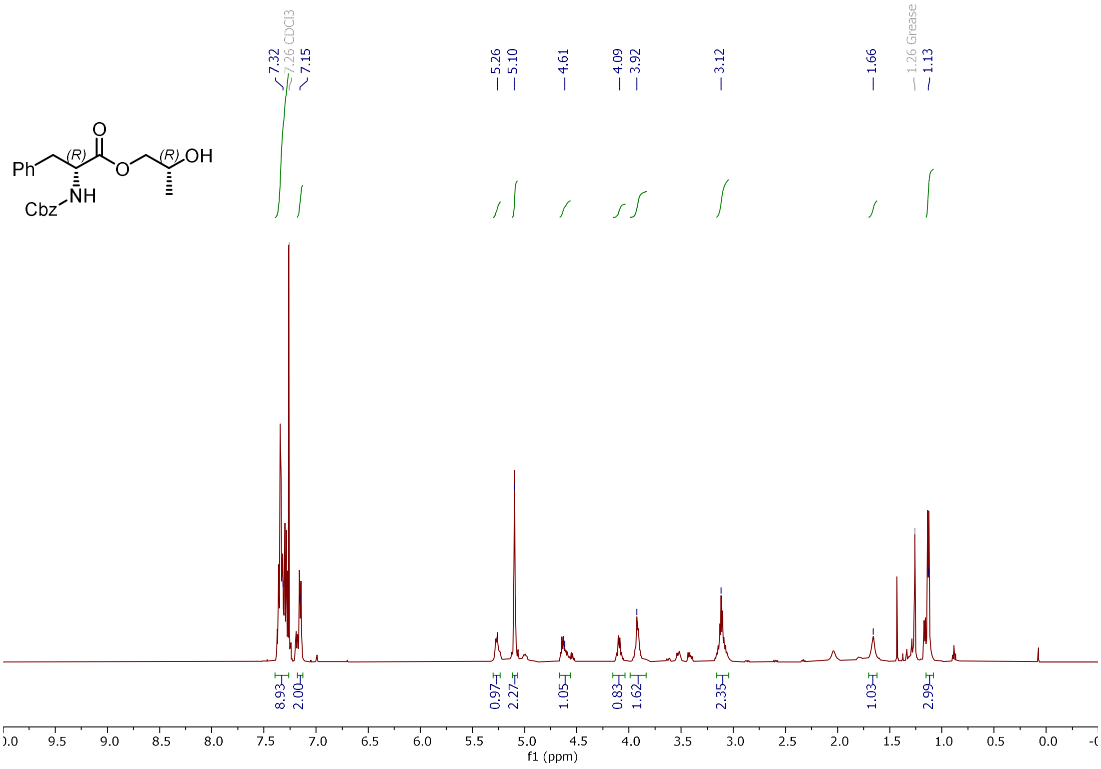

6.5.22 ^{13}C NMR Spectrum of Compound ((*R*,*R*)-S16) (125 MHz, $CDCl_3$)

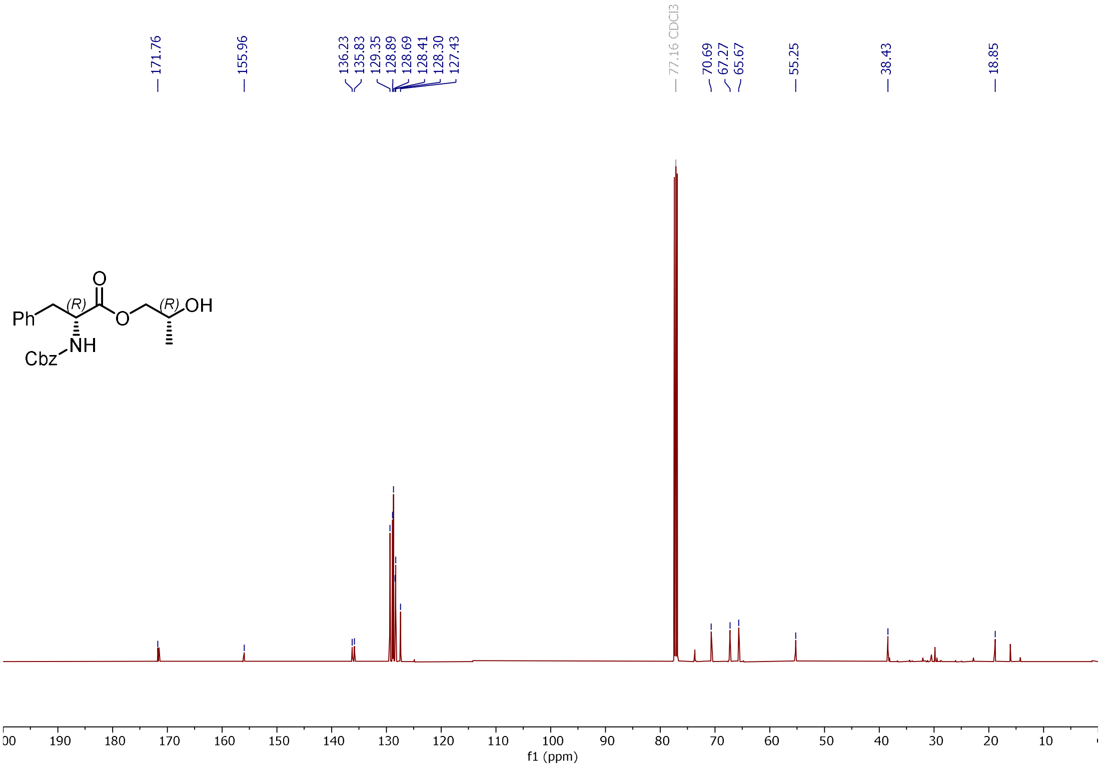

7. Kinetic Modelling

Mathematical modelling of the kinetics for the multi-component stereoretentive-enantioconvergent reactions was carried out using the Berkeley Madonna software package, version 10.2.8 (https://berkeley-madonna.myshopify.com/).[25]

Schematic of the multi-component stereoretentive-enantioconvergent reaction:

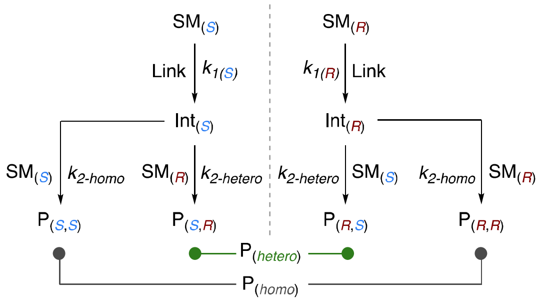

The ordinary differential equations used for the modelling:

$d[SM_{(S)}]/dt = -k_{1(S)}[SM_{(S)}][Link] - k_{2\text{-}homo}[Int_{(S)}][SM_{(S)}] - k_{2\text{-}hetero}[Int_{(R)}][SM_{(S)}]$

$d[SM_{(R)}]/dt = -k_{1(R)}[SM_{(R)}][Link] - k_{2\text{-}homo}[Int_{(R)}][SM_{(R)}] - k_{2\text{-}hetero}[Int_{(S)}][SM_{(R)}]$

$d[Link]/dt = -k_{1(S)}[SM_{(S)}][Link] - k_{1(R)}[SM_{(R)}][Link]$

$d[Int_{(S)}]/dt = k_{1(S)}[SM_{(S)}][Link] - k_{2\text{-}homo}[Int_{(S)}][SM_{(S)}] - k_{2\text{-}hetero}[Int_{(S)}][SM_{(R)}]$

$d[Int_{(R)}]/dt = k_{1(R)}[SM_{(R)}][Link] - k_{2\text{-}hetero}[Int_{(R)}][SM_{(S)}] - k_{2\text{-}homo}[Int_{(R)}][SM_{(R)}]$

$d[P_{(S,R)}]/dt = k_{2\text{-}hetero}[Int_{(S)}][SM_{(R)}]$

$d[P_{(R,S)}]/dt = k_{2\text{-}hetero}[Int_{(R)}][SM_{(S)}]$

$d[P_{(S,S)}]/dt = k_{2\text{-}homo}[Int_{(S)}][SM_{(S)}]$

$d[P_{(R,R)}]/dt = k_{2\text{-}homo}[Int_{(R)}][SM_{(R)}]$

The kinetic constraints and other parameters used for this modelling:

$[SM_{(S)}]_0 = [SM_{(R)}]_0 = [Link]_0 = 100$

$[Int_{(S)}]_0 = [Int_{(R)}]_0 = [P_{(S,R)}]_0 = [P_{(R,S)}]_0 = [P_{(S,S)}]_0 = [P_{(R,R)}]_0 = 0$

STARTTIME = 0

STOPTIME = 100000

$k_{1(S)} = 0.1$

$k_{1(R)} < k_{1(S)}$

$k_{2\text{-}hetero} = k_{2\text{-}homo} = 0.0001$ (multi-component stereoretentive-enantioconvergent reaction)

$k_{2\text{-}hetero} = k_{2\text{-}homo} = 0$ (kinetic resolution)

To obtain plots the following parameter plots were conducted:

1. Parameter plot of final $[P_{(S,R)}]$ and final $[P_{(R,S)}]$ for 100 values of $k_{1(R)}$ ($0.1 < k_{1(R)} < 0.001$), where $k_{2\text{-}hetero} = k_{2\text{-}homo} = 0.0001$
2. Parameter plot of final $[Int_{(S)}]$ and final $[Int_{(R)}]$ for 100 values of $k_{1(R)}$ ($0.1 < k_{1(R)} < 0.001$), where $k_{2\text{-}hetero} = k_{2\text{-}homo} = 0$

Data of $P_{(hetero)}$ ee (at 100% conversion) & Int ee (at 50% conversion) against *s*-factor:

$k_{1(R)}$	*s* factor	$[P_{(S,R)}]$	$[P_{(R,S)}]$	$P_{(hetero)}$ ee	$[Int_{(S)}]$	$[Int_{(R)}]$	Int ee
0.001	100	93.2846749	0.11089698	0.99762522	96.6584064	3.34159363	0.93316813
0.00104762	95.4544587	93.0529971	0.11909061	0.997443642	96.5379246	3.46207539	0.93075849
0.0010975	91.1161731	92.8139373	0.12786005	0.9972486	96.4134631	3.58653687	0.92826926
0.00114976	86.974673	92.5673108	0.13724331	0.997039125	96.2849104	3.71508962	0.92569821
0.0012045	83.0220008	92.3129313	0.14728065	0.996814183	96.1521528	3.84784719	0.92304306
0.00126186	79.2480941	92.0506107	0.15801477	0.996572668	96.0150749	3.98492513	0.9203015
0.00132194	75.6463985	91.7801596	0.16949086	0.996313398	95.8735591	4.12644093	0.91747118
0.00138489	72.207901	91.5013873	0.18175678	0.99603511	95.727486	4.272514	0.91454972
0.00145083	68.926063	91.2141022	0.19486318	0.995736454	95.5767344	4.42326562	0.91153469
0.00151991	65.7933693	90.9181121	0.20886361	0.995415987	95.4211811	4.57881889	0.90842362
0.00159228	62.8030246	90.6132241	0.22381467	0.995072172	95.2607013	4.73929867	0.90521403
0.0016681	59.9484443	90.2992452	0.23977617	0.994703363	95.0951685	4.9048315	0.90190337
0.00174753	57.2236242	89.9759825	0.25681127	0.994307806	94.9244544	5.07554558	0.89848909
0.00183074	54.6227209	89.6432433	0.2749866	0.99388363	94.7484294	5.25157063	0.89496859
0.00191791	52.14009	89.3008359	0.29437246	0.993428835	94.5669622	5.43303785	0.89133924
0.00200923	49.77031	88.9485693	0.31504293	0.992941291	94.3799202	5.62007982	0.8875984
0.0021049	47.5081952	88.5862543	0.33707607	0.992418726	94.1871696	5.81283039	0.88374339
0.00220513	45.3488003	88.2137031	0.36055406	0.991858717	93.9885754	6.01142458	0.87977151
0.00231013	43.2876072	87.8307302	0.38556335	0.991258682	93.7840015	6.21599846	0.87568003
0.00242013	41.3200944	87.4371528	0.41219487	0.99061587	93.5733109	6.42668906	0.87146622
0.00253536	39.4421305	87.0327909	0.44054416	0.98992735	93.3563658	6.64363418	0.86712732
0.00265609	37.6493266	86.6174682	0.47071154	0.989190002	93.1330277	6.86697229	0.86266055
0.00278256	35.9381289	86.1910121	0.50280228	0.988400504	92.9031576	7.09684239	0.85806315
0.00291505	34.3047289	85.7532545	0.53692682	0.987555321	92.6666162	7.33338383	0.85333232
0.00305386	32.7454435	85.3040322	0.57320082	0.986650692	92.4232639	7.57673613	0.84846528
0.00319927	31.2571305	84.8431874	0.61174547	0.985682618	92.1729612	7.82703885	0.84345922
0.0033516	29.836496	84.3705684	0.65268751	0.984646847	91.9155686	8.08443136	0.83831137
0.00351119	28.4803728	83.8860297	0.69615951	0.983538863	91.6509473	8.34905267	0.83301895
0.00367838	27.1858807	83.3894332	0.74229994	0.982353865	91.3789588	8.62104122	0.82757918
0.00385353	25.9502326	82.8806483	0.79125336	0.98108676	91.0994653	8.90053467	0.82198931
0.00403702	24.7707467	82.3595525	0.84317054	0.979732141	90.8123303	9.18766967	0.81624661
0.00422924	23.6449102	81.8260324	0.89820863	0.97828427	90.5174184	9.48258162	0.81034837
0.00443062	22.5702046	81.2799838	0.95653123	0.976737068	90.2145956	9.78540445	0.80429191
0.00464159	21.5443415	80.7213126	1.01830857	0.975084089	89.9037296	10.0962704	0.79807459
0.0048626	20.5651298	80.1499356	1.08371755	0.973318508	89.5846905	10.4153096	0.79169381
0.00509414	19.6303989	79.5657807	1.15294189	0.971433099	89.25735	10.74265	0.785147
0.0053367	18.7381715	78.9687877	1.22617216	0.969420219	88.921583	11.078417	0.77843166
0.00559081	17.8864959	78.3589092	1.30360588	0.967271787	88.5772667	11.4227333	0.77154533
0.00585702	17.0735289	77.7361106	1.38544752	0.964979266	88.2242818	11.7757182	0.76448564
0.00613591	16.2975011	77.1003717	1.47190857	0.962533643	87.8625121	12.1374879	0.75725024
0.00642807	15.556769	76.4516863	1.56320752	0.959925408	87.4918454	12.5081546	0.74983691
0.00673415	14.8496841	75.7900634	1.65956985	0.957144539	87.1121733	12.8878267	0.74224347
0.0070548	14.1747463	75.1155276	1.76122799	0.954180481	86.723392	13.276608	0.73446784
0.00739072	13.5304815	74.42812	1.86842125	0.951022124	86.3254022	13.6745978	0.72650804
0.00774264	12.9154914	73.7278982	1.98139573	0.947657794	85.9181096	14.0818904	0.71836219
0.00811131	12.3284648	73.0149372	2.10040418	0.944075228	85.5014254	14.4985746	0.71002851
0.00849753	11.7681256	72.2893302	2.22570589	0.940261563	85.0752665	14.9247335	0.70150533
0.00890215	11.2332414	71.5511883	2.35756647	0.936203323	84.6395555	15.3604445	0.69279111
0.00932603	10.7226762	70.8006419	2.49625764	0.931886406	84.1942219	15.8057782	0.68388444
0.0097701	10.2353098	70.0378403	2.64205699	0.927296073	83.7392013	16.2607987	0.67478403
0.01023531	9.77009978	69.2629526	2.79524766	0.922416945	83.2744369	16.7255631	0.66548874
0.01072267	9.3260354	68.4761681	2.95611807	0.917232998	82.7998789	17.2001211	0.65599758
0.01123324	8.90215112	67.6776961	3.12496151	0.911727565	82.3154855	17.6845145	0.64630971
0.01176812	8.49753402	66.8677666	3.30207572	0.905883336	81.8212227	18.1787773	0.63642445
0.01232847	8.11130659	66.0466306	3.48776252	0.899682377	81.3170652	18.6829349	0.6263413
0.0129155	7.74263482	65.2145597	3.68232725	0.893106135	80.802996	19.197004	0.61605992

0.01353048	7.3907208	64.3718468	3.88607826	0.88613547	80.2790074	19.7209926	0.60558015
0.01417474	7.05480312	63.5188057	4.09932638	0.878750677	79.745101	20.254899	0.59490202
0.01484968	6.73415185	62.6557714	4.32238423	0.870931524	79.201288	20.798712	0.58402576
0.01555676	6.42807371	61.7830999	4.55556566	0.862657302	78.6475894	21.3524106	0.57295179
0.01629751	6.13590665	60.9011677	4.79918497	0.853906873	78.0840364	21.9159636	0.56168073
0.01707353	5.85701961	60.0103723	5.05355622	0.844658743	77.5106708	22.4893292	0.55021342
0.0178865	5.59080871	59.1111311	5.31899243	0.834891131	76.9275447	23.0724553	0.53855089
0.01873817	5.33670044	58.2038817	5.59580478	0.82458206	76.3347216	23.6652785	0.52669443
0.01963041	5.09413711	57.2890809	5.88430175	0.813709461	75.7322755	24.2677246	0.51464551
0.02056512	4.86260231	56.3672045	6.18478822	0.802251281	75.120292	24.879708	0.50240584
0.02154435	4.64158817	55.4387467	6.49756455	0.790185614	74.4988682	25.5011318	0.48997736
0.0225702	4.43062091	54.5042194	6.82292562	0.777490845	73.8681125	26.1318875	0.47736225
0.02364489	4.22924361	53.5641512	7.16115989	0.764145798	73.2281453	26.7718547	0.46456291
0.02477076	4.03701784	52.6190868	7.51254832	0.750129917	72.5790984	27.4209016	0.45158197
0.02595024	3.85352891	51.6695863	7.8773634	0.735423445	71.9211157	28.0788843	0.43842231
0.02718588	3.6783801	50.7162237	8.25586807	0.720007623	71.254353	28.745647	0.42508706
0.02848036	3.51119157	49.7595863	8.64831469	0.703864904	70.5789778	29.4210222	0.41157956
0.02983647	3.35160292	48.8002735	9.05494392	0.686979176	69.8951696	30.1048304	0.39790339
0.03125716	3.19926698	47.8388953	9.47598371	0.669335995	69.2031196	30.7968804	0.38406239
0.03274549	3.05385566	46.8760717	9.91164816	0.650922834	68.5030309	31.4969691	0.37006062
0.03430469	2.91505331	45.9124307	10.3621365	0.631729322	67.795118	32.204882	0.35590236
0.03593814	2.78255914	44.9486075	10.827632	0.611747507	67.0796071	32.9203929	0.34159214
0.03764936	2.65608765	43.9852428	11.308301	0.590972101	66.3567356	33.6432644	0.32713471
0.03944206	2.53536453	43.0229815	11.8042918	0.569400736	65.626752	34.373248	0.31253504
0.04132012	2.4201285	42.0624712	12.3157336	0.547034198	64.8899156	35.1100844	0.29779831
0.04328761	2.31012985	41.1043607	12.8427357	0.523876666	64.1464963	35.8535037	0.28292993
0.04534879	2.2051305	40.1492984	13.3853867	0.499935913	63.3967744	36.6032256	0.26793549
0.0475081	2.10490422	39.1979309	13.9437533	0.47522351	62.6410401	37.3589599	0.2528208
0.04977024	2.00923283	38.2509013	14.5178795	0.449754978	61.8795929	38.1204071	0.23759186
0.05214008	1.91791037	37.3088478	15.1077862	0.423549929	61.1127419	38.8872581	0.22225484
0.05462277	1.83073835	36.3724017	15.7134702	0.396632154	60.3408047	39.6591953	0.20681609
0.05722368	1.7475283	35.4421867	16.3349036	0.369029682	59.5641071	40.4358929	0.19128214
0.05994843	1.6681004	34.5188165	16.9720333	0.340774784	58.782983	41.217017	0.17565966
0.06280291	1.59228291	33.6028936	17.6247805	0.311903935	57.9977731	42.0022269	0.15995546
0.06579332	1.51991114	32.6950082	18.2930404	0.282457717	57.2088254	42.7911747	0.14417651
0.06892612	1.4508288	31.7957359	18.9766817	0.252480675	56.4164935	43.5835065	0.12832987
0.07220809	1.38488637	30.9056373	19.6755466	0.222021113	55.6211368	44.3788632	0.11242274
0.07564633	1.3219412	30.0252559	20.3894506	0.191130841	54.8231198	45.1768802	0.0964624
0.07924829	1.26185688	29.1551169	21.1181825	0.159864868	54.0228109	45.9771891	0.08045622
0.08302176	1.20450349	28.2957264	21.8615046	0.128281041	53.2205823	46.7794177	0.06441165
0.0869749	1.149757	27.4475697	22.6191527	0.096439647	52.4168091	47.5831909	0.04833618
0.09111628	1.09749871	26.6111106	23.3908367	0.064402969	51.6118685	48.3881315	0.03223737
0.09545485	1.04761571	25.7867899	24.176241	0.032234812	50.806139	49.193861	0.01612278
0.1	1	24.975025	24.975025	0	50	50	0

Plot of $P_{(hetero)}$ ee (at 100% conversion) & Int ee (at 50% conversion) against *s*-factor:

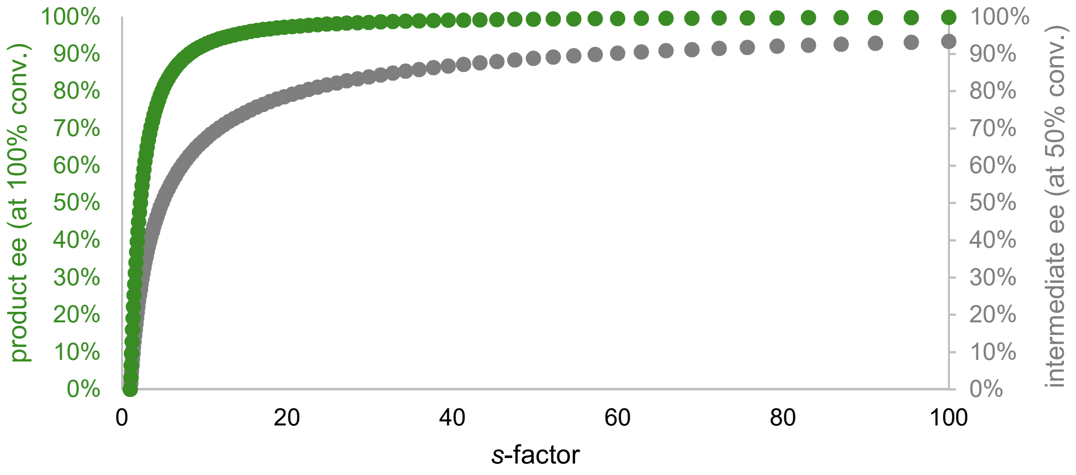

8. References

1. W. H. Brooks, W. C. Guida, K. G. Daniel, The significance of chirality in drug design and development. *Curr. Top. Med. Chem.* **11**, 760–770 (2011).

2. J. R. Brandt, F. Salerno, M. J. Fuchter, The added value of small-molecule chirality in technological applications. *Nat. Rev. Chem.* **1**, 45 (2017).

3. V. Bhat, E. R. Welin, X. Guo, Stoltz, B. M. Advances in Stereoconvergent Catalysis from 2005 to 2015: Transition-Metal-Mediated Stereoablative Reactions, Dynamic Kinetic Resolutions, and Dynamic Kinetic Asymmetric Transformations. *Chem. Rev.* **117**, 4528–4561 (2017).

4. J. T. Mohr, J. T. Moore, B. M. Stoltz, Enantioconvergent catalysis. *Beilstein J. Org. Chem.* **12***,* 2038–2045 (2016).

5. J. Steinreiber, K. Faber, H. Griengl, De-racemization of Enantiomers versus De-epimerization of Diastereomers—Classification of Dynamic Kinetic Asymmetric Transformations (DYKAT). *Chem. Eur. J.* **14**, 8060–8072 (2008).

6. A. M. Harned, From determination of enantiopurity to the construction of complex molecules: The Horeau principle and its application in synthesis. *Tetrahedron* **74**, 3797–3841 (2018).

7. H. D. Flack, Louis Pasteur's discovery of molecular chirality and spontaneous resolution in 1848, together with a complete review of his crystallographic and chemical work. *Acta Cryst.* **A65**, 371–389 (2009).

8. J. M. Keith, J. F. Larrow, E. N. Jacobsen, Practical Considerations in Kinetic Resolution Reactions. *Adv. Synth. Catal.* **343**, 5−26 (2001).

9. H. B. Kagan, J. C. Fiaud, Kinetic resolution. *Top. Stereochem.* **18**, 249–330 (1988).

10. L.-C. Yang, H. Deng, H. Renata, Recent Progress and Developments in Chemoenzymatic and Biocatalytic Dynamic Kinetic Resolution. *Org. Process Res. Dev.* **26**, 1925–1943 (2022).

11. O. Pàmies, J.-E. Bäckvall, Combination of Enzymes and Metal Catalysts. A Powerful Approach in Asymmetric Catalysis. *Chem. Rev.* **103**, 3247–3262 (2003).

12. H. Pellissier, Dynamic kinetic resolution. *Tetrahedron* **59**, 8291–8327 (2003).

13. M. Kitamura, T. Ohkuma, M. Tokunaga, R. Noyori, Dynamic Kinetic Resolution in BINAP–Ruthenium(II) Catalyzed Hydrogenation of 2-Substituted 3-Oxo Carboxylic Esters. *Tetrahedron: Asymmetry* **1**, 1–4 (1990).

14. J. T. Mohr, D. C. Ebner, B. M. Stoltz, Catalytic enantioselective stereoablative reactions: an unexploited approach to enantioselective catalysis. *Org. Biomol. Chem.* **5**, 3571–3576 (2007).

15. B. M. Trost, D. E. Patterson, E. J. Hembre, Dynamic Kinetic Asymmetric Transformations of Conduritol B Tetracarboxylates: An Asymmetric Synthesis of D-*myo*-Inositol 1,4,5-Trisphosphate. *J. Am. Chem. Soc.* **121**, 10834–10835 (1999).

16. W. Kroutil, M. Mischitz, K. Faber, Deracemization of (±)-2,3-disubstituted oxiranes *via* biocatalytic hydrolysis using bacterial epoxide hydrolases: kinetics of an enantioconvergent process. *J. Chem. Soc., Perkin Trans. 1* **1997**, 3629–3636 (1997).

17. H. Ito, S. Kunii, M. Sawamura, Direct enantio-convergent transformation of racemic substrates without racemization or symmetrization. *Nat. Chem.* **2**, 972–976 (2010).

18. F. W. Goetzke, M. Mortimore, S. P. Fletcher, Enantio- and Diastereoselective Suzuki-Miyaura Coupling with Racemic Bicycles. *Angew. Chem. Int. Ed.* **58**, 12128–12132 (2019).

19. J. Sweeney, Aziridine Synthesis via Nucleophilic Attack of Carbene Equivalents on Imines: the Aza-Darzens Reaction. *Eur. J. Org. Chem.* **2009**, 4911–4919 (2009).

20. D. Alickmann, R. Fröhlich, E.-U. Würthwein, Base-Induced Heterochiral Dimerization of an Oxiranyl Carbaldimine: Stereoselective Synthesis of a Highly Functionalized Aziridine. *Org. Lett.* **3**, 1527–1530 (2001).

21. P. J. Cox, N. S. Simpkins, Asymmetric synthesis using homochiral lithium amide bases. *Tetrahedron: Asymmetry* **2**, 1–26 (1991).

22. J. Merad, P. Borkar, F. Caijo, J.-M. Pons, J.-L. Parrain, O. Chuzel, C. Bressy, Double Catalytic Kinetic Resolution (DoCKR) of Acyclic *anti*-1,3-Diols: The Additive Horeau Amplification. *Angew. Chem. Int. Ed.* **56**, 16052–16056 (2017).

23. M. Burns, S. Essafi, J. Bame, S. P. Bull, M. P. Webster, S. Balieu, J. W. Dale, C. P. Butts, J. N. Harvey, V. K. Aggarwal, Assembly-line synthesis of organic molecules with tailored shapes. *Nature* **513**, 183–188 (2014).

24. N. J. Green, A. L. Lawrence, G. Bojase, A. C. Willis, M. N. Paddon-Row, M. S. Sherburn, Domino Cycloaddition Organocascades of Dendralenes. *Angew. Chem. Int. Ed.* **52**, 8333–8336 (2013).

25. F. V. Marcoline, J. Furth, S. Nayak, M. Grabe, R. I. Macey, Berkeley Madonna Version 10–A simulation package for solving mathematical models. *CPT: Pharmacomet. Syst. Pharmacol.* ***11***, 290–301 (2022).

26. M. D. Greenhalgh, J. E. Taylor, A. D. Smith, Best practice considerations for using the selectivity factor, *s*, as a metric for the efficiency of kinetic resolutions. *Tetrahedron* **74**, 5554–5560 (2018).

27. A. F. Zahrt, S. V. Athavale, S. E. Denmark, Quantitative Structure–Selectivity Relationships in Enantioselective Catalysis: Past, Present, and Future. *Chem. Rev.* **120**, 1620–1689 (2020).

28. K. C. Harper, M. S. Sigman, Predicting and optimizing asymmetric catalyst performance using the principles of experimental design and steric parameters. *Proc. Natl. Acad. Sci. U.S.A.* **108**, 2179–2183 (2011).

29. C. Schmidt-Dannert, F. H. Arnold, Directed evolution of industrial enzymes. *Trends Biotechnol.* **17**, 135–136 (1999).

30. A. C. Spivey, A. Maddaford, A. J. Redgrave, Asymmetric Catalysis of Acyl Transfer by Lewis Acids and Nucleophiles. A Review. *Org. Prep. Proced. Int.* **32**, 331−365 (2000).

31. M. Binanzer, S.-Y. Hsieh, J. W. Bode, Catalytic Kinetic Resolution of Cyclic Secondary Amines. *J. Am. Chem. Soc.* **133**, 19698–19701 (2011).

32. I. Kreituss, J. W. Bode, Catalytic Kinetic Resolution of Saturated N-Heterocycles by Enantioselective Amidation with Chiral Hydroxamic Acids. *Acc. Chem. Res.* **49**, 2807−2821 (2016).

33. D. A. Sánchez, G. M. Tonetto, M. L. Ferreira, *Burkholderia cepacia* lipase: A versatile catalyst in synthesis reactions. *Biotechnol. Bioeng.* **115**, 6–24 (2017).

34. A. Hietanen, T. Saloranta, R. Leino, L. T. Kanerva, Lipase catalysis in the preparation of 3-(1-amino-3-butenyl)pyridine enantiomers. *Tetrahedron: Asymmetry* **23**, 1629–1632 (2012).

35. S. Roy, K-F. Chen, R. Gurubrahamam, K. Chen, Organocatalytic Kinetic Resolution of Racemic Secondary Nitroallylic Alcohols Combined with Simultaneous Desymmetrization of Prochiral Cyclic Anhydrides. *J. Org. Chem.* **79**, 8955–8959 (2014).

36. K. Ishihara, M. Kubota, H. Kurihara, H. Yamamoto, Scandium Trifluoromethanesulfonate as an Extremely Active Lewis Acid Catalyst in Acylation of Alcohols with Acid Anhydrides and Mixed Anhydrides. *J. Org. Chem.* **61**, 4560–4567 (1996).

37. S. Dong, X. Liu, Y. Zhu, P. He, L. Lin, X. Feng, Organocatalytic Oxyamination of Azlactones: Kinetic Resolution of Oxaziridines and Asymmetric Synthesis of Oxazolin-4-ones. *J. Am. Chem. Soc.* **135**, 10026−10029 (2013).

38. J. Hang, S.-K. Tian, L. Tang, L. Deng, Asymmetric Synthesis of α-Amino Acids via Cinchona Alkaloid-Catalyzed Kinetic Resolution of Urethane-Protected α-Amino Acid *N*-Carboxyanhydrides. *J. Am. Chem. Soc.* **123**, 12696–12697 (2001).

39. V. Rodeschini, N. S. Simpkins, F. Zhang, Chiral Lithium Amide Base Desymmetrization Of A Ring Fused Imide: Formation Of (3as,7as)-2-[2-(3,4-Dimethoxyphenyl)-Ethyl]-1,3-Dioxo-Octahydro-Isoindole-3a-Carboxylic Acid Methyl Ester. *Org. Synth.* **84**, 306–316 (2007).

40. K. Aoki, K. Koga, Stereoselective Reactions. XXXII. Enantioselective Deprotonation of 4-*tert*-Butylcyclohexanone by Fluorine-containing Chiral Lithium Amides Derived from 1-Phenylethylamine and 1-(1-Naphthyl)ethylamine. *Chem. Pharm. Bull.* **48**, 571–574 (2000).

41. E. Curthbertson, P. O'Brien, T. D. Towers, Practical One-Step Synthesis of Koga's Chiral Bases. *Synthesis* **5**, 693–695 (2001).

42. T. Ogiyama, K. Yonezawa, M. Inoue, T. Watanabe, Y. Sugano, T. Gotoh, T. Kiso, A. Koakutsu, S. Kakimoto, J. Shishikura, Discovery of a 1-isopropyltetrahydroisoquinoline derivative as an orally active N-type calcium channel blocker for neuropathic pain. *Bioorg. Med. Chem.* **23**, 4624–4637 (2015).

43. S. Kobayashi, K. Hirano, M. Sugiura, α-Aminoallylation of aldehydes in aqueous ammonia. *Chem. Commun.* **2005**, 104–106 (2005).

44. S. Hinkes, C. Klein, Virtues of Volatility: A Facile Transesterification Approach to Boronic Acids. *Org. Lett.* **21**, 3048–3052 (2019).

45. M. Sugiura, K. Hirano, S. Kobayashi, α-Aminoallylation of Aldehydes with Ammonia: Stereoselective Synthesis of Homoallylic Primary Amines. *J. Am. Chem. Soc.* **126**, 7182–7183 (2004).

46. Y. Jiang, S. Schaus, Asymmetric Petasis Borono-Mannich Allylation Reactions Catalyzed by Chiral Biphenols. *Angew. Chem. Int. Ed.* **56**, 1544–1548 (2017).

47. H. Ren, W. Wullf. Direct Catalytic Asymmetric Aminoallylation of Aldehydes: Synergism of Chiral and Nonchiral Brønsted Acids. *J. Am. Chem. Soc.* **133**, 5656–5659 (2011).

48. B. Vakulya, S. Varga, A. Csámpai, T. Soós, Highly Enantioselective Conjugate Addition of Nitromethane to Chalcones Using Bifunctional Cinchona Organocatalysts. *Org. Lett.* **7**, 1967–1969 (2005).

49. A. Burke, P. M. Barrulas, Novel Picolinamide-Cinchona Organocatalysts and Derivatives. World Intellectual Property Organization, WO 2015/052656 A1 (2015).

50. J. Xu, Y. Hu, D. Huang, K-H. Wang, C. Xu, T. Niu, Thiourea-Catalyzed Enantioselective Fluorination of β-Keto Esters. *Adv. Synth. Catal.* **354**, 515–526 (2012).

51. I. Deb, M. Dadwal, S. M. Mobin, I. N. N. Namboothiri, Hydroxyalkylation of Conjugated Nitroalkenes with Activated Non-enolizable Carbonyl Compounds. *Org. Lett.* **8**, 1201–1204 (2006).

52. O. Eisenstein, M. M. Kayser, Theoretical study of regioselectivity in nucleophilic addition to unsymmetrical cyclic anhydrides. Intrinsic reactivity and influence of the cation. *Can. J. Chem.* **59**, 2457–2462 (1981).

53. J. Inanaga, K. Hirata, H. Saeki, T. Katsuki, M. Yamaguchi, A Rapid Esterification by Means of Mixed Anhydride and Its Application to Large-ring Lactonization. *Bull. Chem. Soc. Jpn.* **52**, 1989–1993 (1979).

54. I. Shiina, R. Ibuka. M. Kubota, A New Condensation Reaction for the Synthesis of Carboxylic Esters from Nearly Equimolar Amounts of Carboxylic Acids and Alcohols Using 2-Methyl-6-nitrobenzoic Anhydride. *Chem. Lett.* **31**, 286–287 (2002).

55. S. Mayr, M. Marin-Luna, H. Zipse, Size-Driven Inversion of Selectivity in Esterification Reactions: Secondary Beat Primary Alcohols. *J. Org. Chem.* **86**, 3456–3489 (2021).

56. G. Laconde, M. Amblard, J. Martinez, Synthesis of α-Amino Acid *N*-Carboxyanhydrides. *Org. Lett.* **23**, 6412–6416 (2021).

57. G. Schäfer, J. Bode, Synthesis of sterically hindered *N*-acylated amino acids from *N*-carboxyanhydrides. *Org. Lett.* **16**, 1526–1529 (2014).

58. W. Fuller, M. Cohen, M. Shabankareh, R. Blair, M. Goodman, F. Naider, Urethane protected amino acid *N*-carboxyanhydrides and their use in peptide synthesis. *J. Am. Chem. Soc.* **112**, 7414–7416 (1990).

59. R. Okabe, N. Sugisawa, S. Fuse, A micro-flow rapid dual activation approach for urethane-protected α-amino acid *N*-carboxyanhydride synthesis. *Org. Biomol. Chem.* **20**, 3303–3310 (2022).

60. H. Stewart, A. Hanby, T. King, A. Bond, T. Moss, H. Sore, D. Spring, An efficient, stereocontrolled and versatile synthetic route to bicyclic partially saturated privileged scaffolds. *Chem. Commun.* **56**, 6818–6821 (2020).

61. F. Manoni, C. Rumo, L. Li, P. Harran, Unconventional Fragment Usage Enables a Concise Total Synthesis of (−)-Callyspongiolide. *J. Am. Chem. Soc.* **140**(4), 1280–1284 (2018).

62. G. Kundu, T. Sperger, K. Rissanen, F. Schoenebeck, A Next-Generation Air-Stable Palladium(I) Dimer Enables Olefin Migration and Selective C-C Coupling in Air. *Angew. Chem. Int. Ed.* **59**, 21930–21934 (2020).

63. N. Li, Y. Gui, M. Chu, M. You, X. Qiu, H. Liu, S. Wang, M. Deng, B. Ji, Cobalt-Catalyzed Deprotection of Allyl Carboxylic Esters Induced by Hydrogen Atom Transfer. *Org. Lett.* **23**, 8460–8464 (2021).
